# Novel Homo-Bivalent and Polyvalent Compounds Based on Ligustrazine and Heterocyclic Ring as Anticancer Agents

**DOI:** 10.3390/molecules24244505

**Published:** 2019-12-09

**Authors:** Jiawen Wang, Ge Hong, Guoliang Li, Wenzhi Wang, Tianjun Liu

**Affiliations:** 1 Graduate Institute, Tianjin University of Traditional Chinese Medicine, Tianjin 301617, China; m13260192393@163.com; 2 Tianjin Key Laboratory of Biomedical Materials, Institute of Biomedical Engineering, Chinese Academy of Medical Sciences & Peking Union Medical College, Tianjin 300192, China; hongge6688@aliyun.com (G.H.); liguoliangsx@163.com (G.L.); wxwnx123@163.com (W.W.); 3 State Key Laboratory of Bioactive Substances and Functions of Natural Medicines, Institute of Materia Medica, Chinese Academy of Medical Sciences and Peking Union Medical College, Beijing 100050, China

**Keywords:** bivalency, polyvalency, antitumor, apoptosis, cell cycle

## Abstract

Bivalent and polyvalent inhibitors can be used as antitumor agents. In this experiment, eight ligustrazine dimers and seven ligustrazine tetramers linked by alkane diamine with different lengths of carbon chain lengths were synthesized. After screening their antiproliferation activities against five cancer cell lines, most ligustrazine derivatives showed better cytotoxicity than the ligustrazine monomer. In particular, ligustrazine dimer **8e** linked with decane-1,10-diamine exhibited the highest cytotoxicity in FaDu cells with an IC_50_ (50% inhibiting concentration) value of 1.36 nM. Further mechanism studies suggested that **8e** could induce apoptosis of FaDu cells through the depolarization of mitochondrial membrane potential and S-phase cell cycle arrest. Inspired by these results, twenty-seven additional small molecule heterocyclic dimers linked with decane-1,10-diamine and nine cinnamic acid dimers bearing ether chain were synthesized and screened. Most monocyclic and bicyclic aromatic systems showed highly selective anti-proliferation activity to FaDu cells and low toxicity to normal MCF 10A cells. The structure-activity relationship revealed that the two terminal amide bonds and the alkyl linker with a chain length of 8–12 carbon were two important factors to maintain its antitumor activity. In addition, the ADMET calculation predicted that most of the potent compounds had good oral bioavailability.

## 1. Introduction

Natural products play a vital role in the development of the drug, especially anticancer drugs [1]. Ligustrazine (2,3,5,6-tetramethylpyrazine, TMP), an important component of the Chinese traditional medicinal herb Chuanxiong (*Ligusticum chuanxiong Hort)*, has been of wide clinical use for cardiovascular and cerebrovascular diseases [2]. Recently, TMP has been reported to possess anticancer activity in vitro and in vivo, inducing cancer cell apoptosis and cell cycle modulation [3,4]. More importantly, the introduction of different substituted groups into ligustrazine has helped to improve the antitumor activity. Many ligustrazine derivatives with potent antitumor activity have been developed in the past few years, like monocarbonyl ligustrazine-curcumin hybrids [5], ligustrazine-betulinic acid hybrids [6], and ligustrazine-rhein derivatives [7]. This advancement stimulated our interest in using TMP as the scaffold to synthesize new antitumor agents.

In the interaction between host and guest molecules, both bivalency and polyvalency could enhance the affinity and activity of monovalent ligand [8,9]. This phenomenon has attracted attention in drug design. Many effective bivalent anti-cancer drugs have been found, such as artemisinin-derivative dimers [10], jesterone dimer [11], indole-3-carbinol dimer [12], bis-daunorubicin [13]. Previous work in this field has designed and synthesized several ligustrazine dimers linked by cyclohexanone and oxime [14], curcumin [15], or triterpenes [16], which had a good cytotoxic effect on human cancer cells. The result showed that the dimerization of ligustrazine could obviously improve the antitumor potency of its monomer. 

With an effort to developing new antitumor agents, we designed and synthesized a series of novel TMP dimers and tetramers linked with different alkyl diamines and screened their anti-proliferation potential on a panel of human cancer cell lines, including HeLa, Hep G2, MCF-7, FaDu, and A549. Based on these experimental results, according to the importance of small molecular heterocyclic rings in drug discovery [17], additional dimers series of aromatic rings instead of ligustrazine, a total of 51 new compounds were synthesized, screened, and their structure-activity relationship was briefly discussed. Meanwhile, through morphological observation and flow cytometric analysis, the antitumor mechanisms of the most potent one were preliminarily discussed in this study. ADMET properties of all compounds were also evaluated to explore the drug-likeness.

## 2. Results and Discussion

### 2.1. Chemistry

**Scheme 1:**
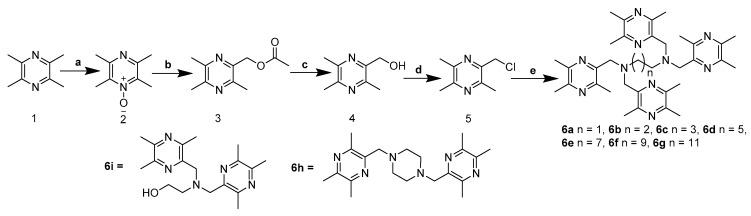
Synthesis of the TMP complexes **6a**–**6i**. *Reagents and conditions***:** (a) 30% H_2_O_2_, CH_3_COOH, 95 °C, 2 h; (b) (CH_3_CO)_2_O, 140 °C, 2.5 h; (c) CH_3_ONa, CH_3_OH, r.t., 0.5 h; (d) SOCl_2_, CH_2_Cl_2_, r.t., 2.5 h; (e) n-diaminoalkane (piperazine and monoethanolamine), K_2_CO_3_, CH_3_CN, 95 °C, 2 h.

**Scheme 2:**
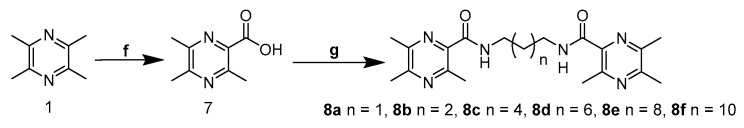
Synthesis of the Bis-TMP hybrids **8a**–**8f**. *Reagents and conditions***:** (f) KMnO_4_, H_2_O, 37 °C, 24 h; (g) n-diamino-alkane, EDCI/DMAP, r.t., 12 h.

In this study, 51 novel designed compounds were synthesized. Series **6a**–**g** and **8a**–**f** were obtained according to the synthetic method described in Scheme 1 and Scheme 2. The important intermediate 2-chloromethyl-3,5,6-trimethyl-pyrazine **5** was prepared from ligustrazine by the tandem reaction: Boekelheide reaction [18] and deprotection and the chlorination reaction [19]. The key intermediate 2-carboxylic acid-3,5,6-trimethyl-pyrazine **7** was synthesized by the one-pot reaction, as previously reported [20]. To explore the length effect, the TMP moieties, with 2–12 methylene alkyl chain as a linker, were synthesized.

**Scheme 3:**
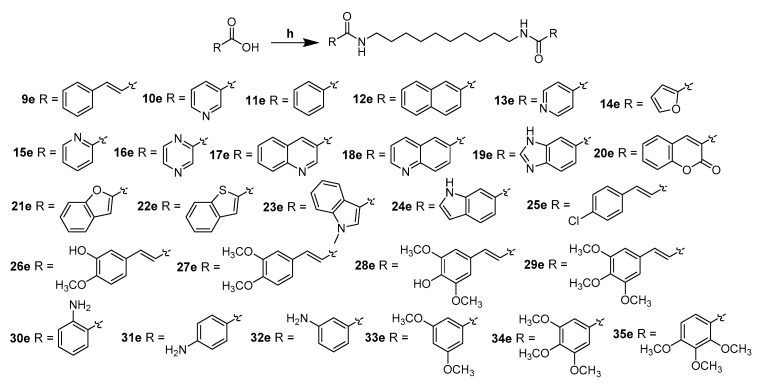
Synthesis of compounds **9e–35e**. *Reagents and conditions***:** (h) decane-1,10-diamine, EDCI/DMAP, r.t., 12 h.

**Scheme 4:**
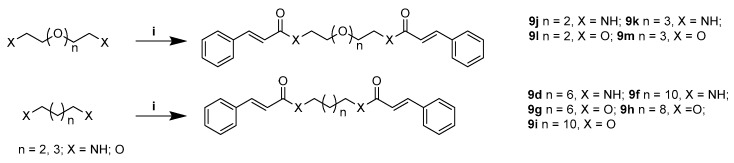
Synthesis of compounds **9d**, **9f**–**9m**. *Reagents and conditions***:** (i) cinnamic acid, EDCI/DMAP, r.t., 12 h.

**Figure 1:**
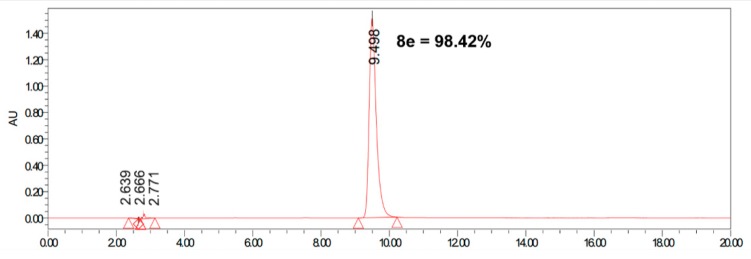
The purity of compound **8e** as a representative analyzed by HPLC (Kromasil C18 column, eluted by methanol/water (80/20) containing 0.1% trifluoroacetic acid at a flow rate of 1 mL/min).

Using heteroaromatic acids with decane-1,10-diamine as raw materials, the target compounds **9e**–**35e** (outlined in Scheme 3) linked by amide chain were synthesized with EDCI (1-(3-Dimethylaminopropyl)-3-ethylcarbodiimide hydrochloride) and DMAP (4-Dimethylaminopyridine) in anhydrous CH_2_Cl_2_ at room temperature. Series **9d**, **9f**–**l** (shown in Scheme 4) linked by ether chain or amide chain were synthesized by the above method. All the newly synthesized compounds were characterized by ^1^H-NMR, ^13^C-NMR, and high-resolution mass spectra (HRMS). Details were provided in the experimental part. The purity of the compounds was over 95% measured by HPLC (Figure 1).

### 2.2. Anti-Proliferative Activity In Vitro

For the newly synthesized compounds, their anti-proliferative activity on human cancer cell lines, HeLa (cervical carcinoma), Hep G2 (hepatoma carcinoma), MCF-7 (breast carcinoma), FaDu (head and neck carcinoma), A549 (lung carcinoma), and normal mammary epithelial cell line MCF 10A, was screened by MTT assay [21] with doxorubicin (DOX) as the positive control. The IC_50_ values of these compounds are summarized in Table 1. Among the ligustrazine derivatives, **6a**–**g** and **8a**–**f**, the tetramers **6f** and **6g** had broad-spectrum cytotoxic activities in all cell lines (IC_50_ 6.57–20.83 µM), and the cytotoxicity activities increased with the increase of carbon chain length. The dimeric ligustrazine **8d**, **8e**, **8f** showed the most promising anticancer activity in most tested tumor cell lines with IC_50_ values between 0.00136 and 6.35 µM, a much better result than DOX and TMP. In particular, compound **8e**, two ligustrazine rings linked with decane-1,10-diamine, exhibited the best anti-proliferative ability in cells (except Hep G2), and compared with other linkers, its IC_50_ values were 1.42 ± 0.71 µM, 0.037 ± 0.001 µM, 0.00136 ± 0.00035 µM, 1.05 ± 0.05 µM, and 0.047 ± 0.008 µM against HeLa, MCF-7, FaDu, A549, and MCF 10A, respectively. In light of these results, we replaced TMP with aromatic heterocycles similar to ligustrazine in electronic space. The novel small aromatic molecule and heterocyclic dimers **9e**–**35e** were synthesized, and their anti-proliferative ability was evaluated. Compounds **9e**, **10e**, **11e**, **17e**, **18e**, **21e**, **22e, 23e,** and **25e,** aromatic acid dimers (cinnamoyl, nicotinoyl, benzoyl, quinoline-3-carboxyl, quinoline-6-carboxyl, benzofuran-2-carboxyl, benzothiophene-2-carboxyl, indol-3-carboxyl, chlorinated cinnamoyl), were linked by decane-1,10-diamine, which had potent anti-proliferative ability in FaDu or A549 cell lines. Their IC_50_ in FaDu cell lines were less than 1 µM, in the range of 20 nM (benzothiophene-2-carboxyl) and 697 nM (benzofuran-2-carboxyl). Also, they had nearly no toxicity in normal cells like MCF 10A. In series **9d**–**n**, compounds **9d**–**f** (IC_50_ < 1 µM), two cinnamic acids linked by C8, C10, C12 chain with two terminal amide bonds, were more effective than compounds **9g**–**i** (IC_50_ > 20 µM), two cinnamic acids linked by the same alkyl chain with two terminal ester bonds instead of amide bonds. This demonstrated the vital role of two-terminal amide bonds in antitumor activity. Compared with compounds **9d**–**f**, the inhibition of compounds **9j**–**m**, two cinnamic acids linked by the similar length ether chain instead of alky chain, was weaker in tumor cells (IC_50_ > 20 µM), which confirmed that the alkyl chain played an important role in the structure. Then, we evaluated the position effect of the substitution at the ortho, meta, or para positions of the same aromatic ring. Compounds **25**–**35e** and the unsubstituted compounds **9e** and **11e** were all derivatives of cinnamic acid or benzoic acid with different substituents and electronic effects. The results of the compounds **26**–**27e** or **28**–**29e** confirmed that the substituents had a general effect on the activity, -OH, -OCH_3_, -NH_2_, and other electron donor groups could significantly reduce the activity, with the influence of -OH greater than that of -OCH_3_. However, the electron attraction group like -Cl (**25e**) had no obvious effect. The compounds with para-substituted analogs (**31e**, para position, IC_50_ = 5.594 μM) exhibited better cell growth inhibitory activities in FaDu cells than ortho- and meta- substituted analogs (**30e**, **32e**, ortho and meta position, IC_50_ > 20 μM). It is worth noting that para -OCH_3_ substitution could enhance anticancer activity. The IC_50_ value of compound **34e** was 5.853 ± 0.408 μM, the IC_50_ value of para- OCH_3_ was half that of the parent compound **33e**, and the IC_50_ value of compound **33e** was 10.393 ± 0.949 μM. Generally, there were three crucial elements in the structure-activity relationship of these systems: alkyl chain linker, two-terminal amide bonds, and heteroaromatic substituents. This SAR (structure-activity relationship) study would help to discover more effective compounds in the future.

To study the selective antiproliferative activities of bivalent and polyvalent inhibitors in normal cells and cancer cell lines, the cytotoxicity of compounds in FaDu cells and normal mammary epithelial MCF 10A cells was measured. The results listed in Table 1 showed that although the ligustrazine dimers **8d** and **8e** had high cytotoxicity in MCF 10A, their IC_50_ values were 2.69 ± 0.46 and 0.047 ± 0.008 μM, while the IC_50_ values in FaDu cells were 110 ± 30 and 1.36 ± 0.035 nM, respectively, which were far lower than that in MCF 10A. The selective index (SI) between MCF10A and Fadu (IC_50_^MCF 10A^/IC_50_^FaDu^) was 24.45 and 34.56, respectively. In addition, the heteroaromatic acid dimers **9e**–**12e**, **21e**–**23e,** and **25e** had the similar activity, which was stronger than doxorubicin in FaDu cells, and had high selectivity in FaDu cells and MCF 10A cells with SI (IC_50_^MCF 10A^/IC_50_^FaDu^) values over 28.69. In contrast, the SI (IC_50_^MCF 10A^/IC_50_^FaDu^) value of doxorubicin was 0.43. These results encouraged us to further investigate the possible cellular mechanisms.

### 2.3. Colony Formation Assay 

**Figure 2:**
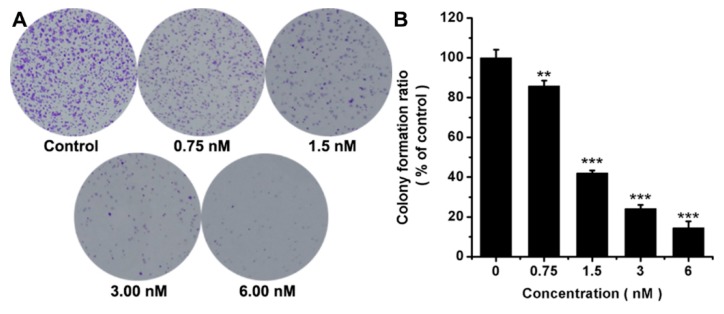
Effect of **8e** on the colony formation in FaDu cells. (**A**) Representative images of FaDu colonies after treatment at the indicated concentrations for 10 days; (**B**) The percentage of colonies compared with control group, *n* = 3. **: *p* < 0.01; ***: *p* < 0.001 versus control.

As ligustrazine dimer **8e** had the most potent antiproliferative activity in all synthesized compounds, the colony formation assay in FaDu cells was conducted. As shown in Figure 2, FaDu cells treated with compound **8e** at concentrations 0.75 nM, 1.50 nM, 3 nM, 6 nM for 10 days formed smaller and fewer colonies compared to untreated control group, indicating that compound **8e** had a dose-dependent inhibition on the proliferation of FaDu cells, and the colony inhibition rate reached around 85.4% at 6 nM.

### 2.4. Live/Dead Staining 

**Figure 3:**
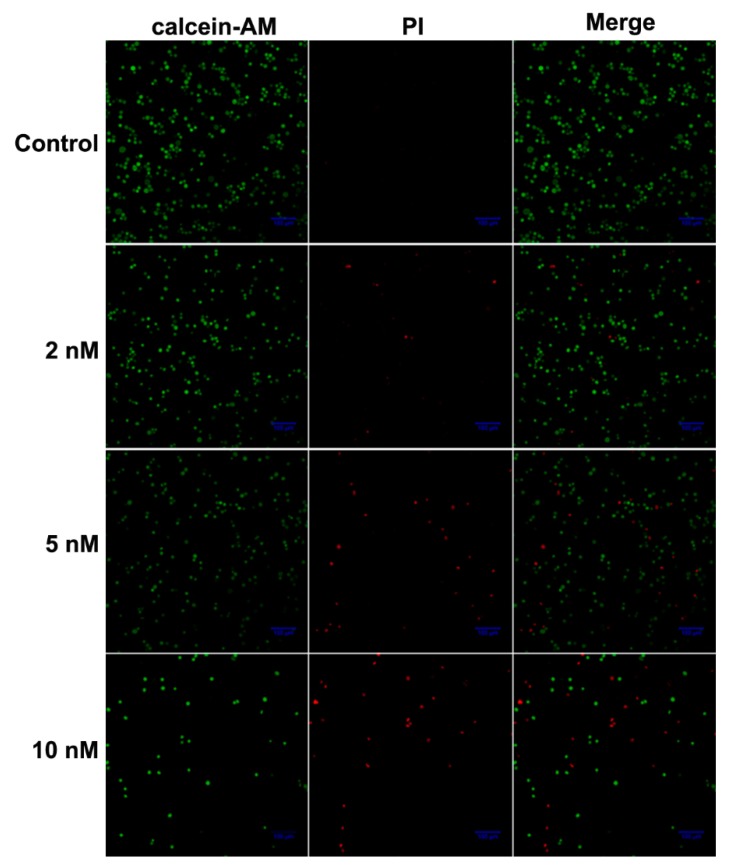
The confocal images of FaDu cells after incubation with compound **8e** at concentrations of 0, 2, 5, and 10 nM for 24 h, respectively. Viable cells were stained in green with Calcein-AM, and dead cells were stained in red with PI. The scale bar was 100 μm.

Live-dead double staining experiment was conducted to visually evaluate the cell toxicity of compound **8e**, the most potent one obtained by MTT assay. Propidium iodide (PI)-stained dead cells in red and calcein AM-stained living cells in green. The number of dead cells increased with the dose of **8e** while living cells decreased. Simultaneously, the densities of **8e**-treated cells were much lower than that of the control group. The concentration-dependent loss of FaDu cell viability was consistent with the MTT results (Figure 3). 

### 2.5. Morphological Observation by Hoechst 33,342 Staining 

**Figure 4:**
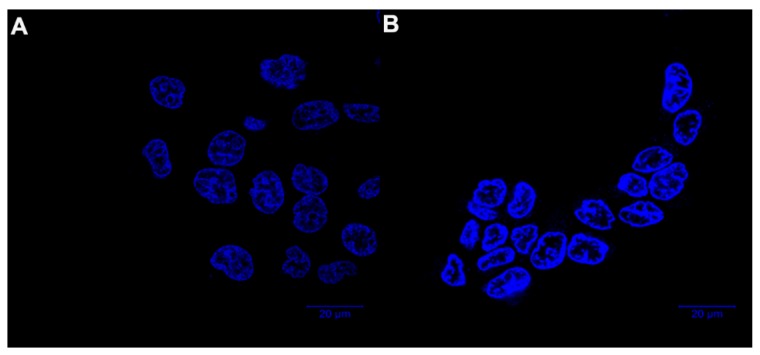
Hoechst staining of FaDu cells treated with compound **8e,** (**A**) Control group; (**B**) 20 nM group.

To investigate whether the anti-proliferation behavior of **8e** was related to the apoptosis pathway, the interaction between **8e**- and FaDu cells was analyzed through Hoechst 33,342 staining with a laser scanning confocal microscope. As shown in Figure 4, cells without treatment as the control group exhibited almost negligible apoptotic characteristics with homogenous and round blue-stained nuclei. On the contrary, the typical apoptotic nuclei with irregular nuclear morphology and condensation of chromatin (much brighter stained cells) were observed, and most FaDu cells were shrunken and rounded up from the culture dish after exposure to **8e**. The result demonstrated that inducing apoptosis in FaDu cells might be one cause of cell growth inhibition by compound **8e**.

### 2.6. Apoptosis Analysis by Annexin V-FITC/PI Staining 

**Figure 5:**
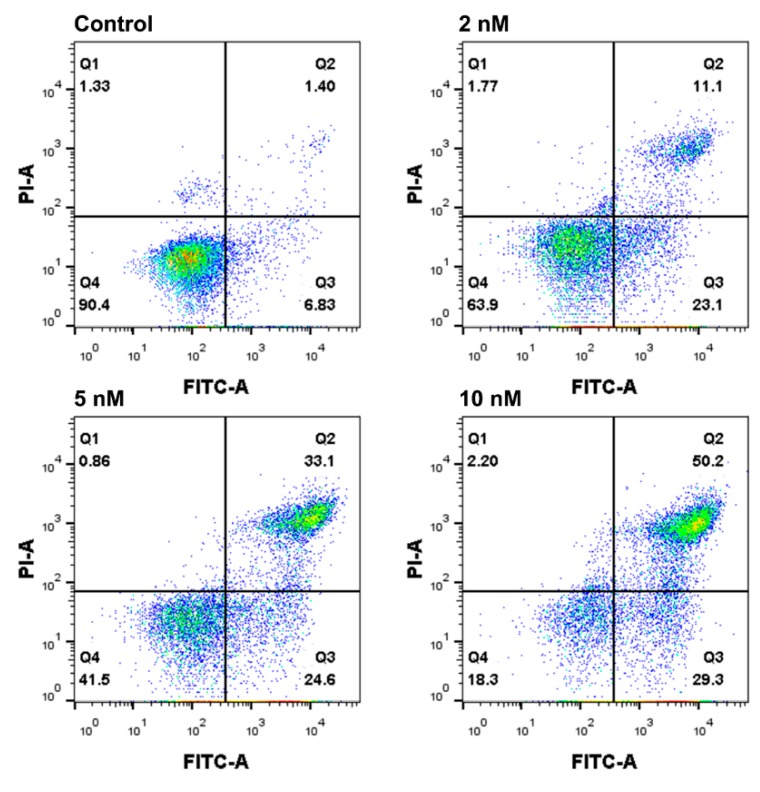
Flow cytometry analysis of apoptosis using annexin V-FITC/PI double staining in FaDu cells treated with compound **8e** at the indicated concentrations for 24 h. The percentage of cells was given in the respective quadrants.

To substantiate whether the cell death induced by the ligustrazine dimer **8e** was associated with apoptosis, the interaction of FaDu cells with **8e** was further analyzed by an annexin V-FITC/PI staining, and the apoptosis ratios were quantitated by flow cytometry. The cells were divided into four quadrants: Q1 represented necrotic cells (annexin V^−^/PI^+^), Q2 represented late apoptotic cells (annexin V^+^/PI^+^), Q3 represented early apoptotic cells (annexin V^+^/PI^−^), Q4 represented living cells (annexin V^−^/PI^−^). As shown in Figure 5, after being exposed to different concentrations of **8e** (2, 5, 10 nM) for 24 h in FaDu cells, the apoptotic cells (including the early and late apoptosis ratios) increased gradually from 8.23% of the control to 34.2%, 57.7%, 79.5%, respectively. The results revealed that **8e** could induce FaDu cells’ apoptosis in a dose-dependent manner. This was consistent with other reports showing TMP and its derivatives could induce apoptosis of cancer cells [22,23,24].

### 2.7. Mitochondrial Membrane Potential (ΔΨm) Analysis

**Figure 6:**
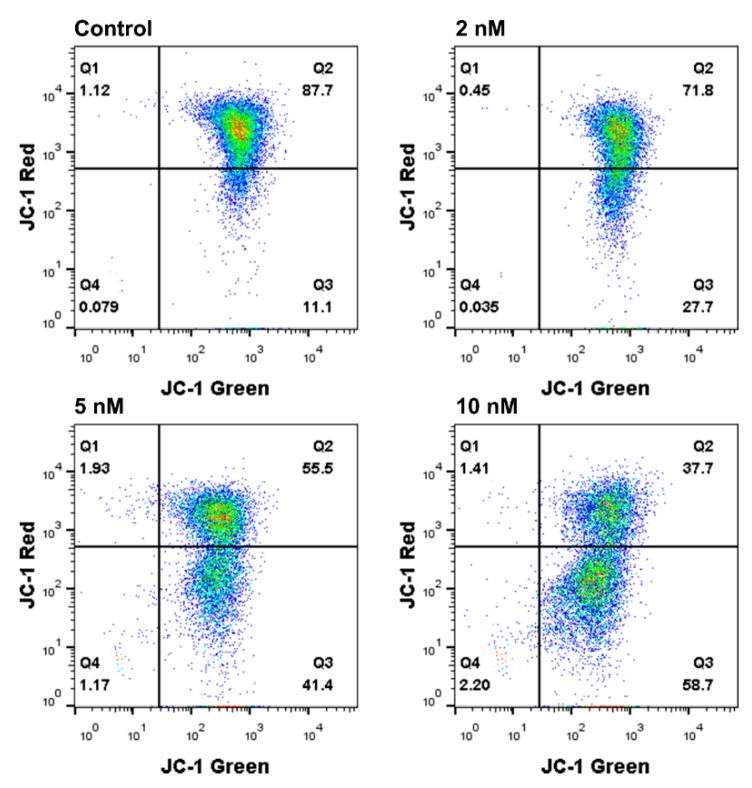
Flow cytometry analysis of the mitochondrial membrane potential using the JC-1 (tetraethylbenzimidazolylcarbocyanine iodide) dye in FaDu cells treated with compound **8e** at the indicated concentrations for 24 h. The percentage of cells was given in the respective quadrants.

Mitochondria play an essential role in cell apoptotic progression by regulating mitochondrial membrane potential (*ΔΨ*m). Depolarization of *ΔΨ*m is considered an indicator of cell apoptosis [25]. To better understand the mechanism of **8e**-induced FaDu cells apoptosis, the depolarization of *ΔΨ*m was quantitated by JC-1 (tetraethylbenzimidazolylcarbocyanine iodide) dye. As a mitochondrial-specific dual-fluorescence probe, JC-1 selectively entered the mitochondria. In healthy cells with high *ΔΨ*m, it accumulated in the mitochondrial matrix as J-aggregates with red fluorescence, while in the reduced *ΔΨ*m cells, it remained in the cytoplasm as a monomer with green fluorescence. The change of *ΔΨ*m could be easily detected by the change of fluorescence color. Flow cytometric analysis of FaDu cells treated with different concentrations of **8e** (2, 5, 10 nM) for 24 h revealed that the ratio of green to red fluorescence was significantly higher than that of the control group in a concentration-dependent manner, indicating that the number of lower *ΔΨ*m cells increased (Figure 6). These results suggested that **8e** could induce apoptosis of FaDu cells by decreasing *ΔΨ*m. 

### 2.8. Cell Cycle Analysis

**Figure 7:**
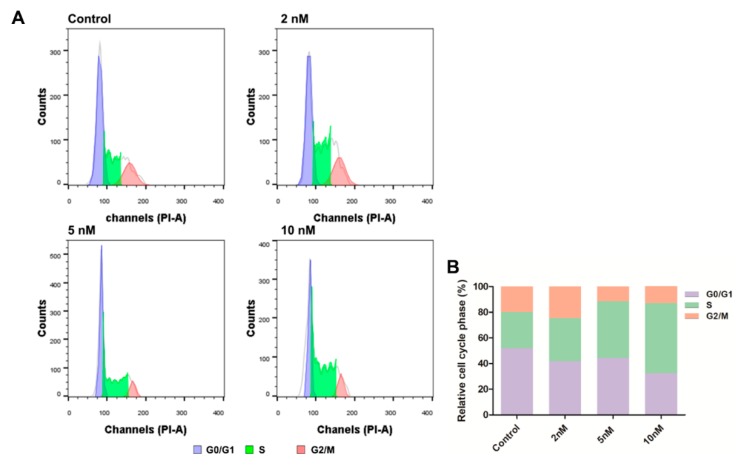
Flow cytometry analysis for cell cycle distribution of FaDu cells. (**A**) Representative cell cycle distribution of FaDu cells with compound 8e treatment (0, 2, 5, and 10 nM) for 24 h. The tall peak (**left**) represents cells in the G0/G1 phase, the small peak (**right**) represents cells in the G2/M phase, and the middle area represents cells in the S phase. (**B**) Quantification of the percentage of cell cycle distribution.

In order to investigate whether **8e** induced cell cycle disturbances in FaDu cells, the flow cytometric analysis was performed with PI staining. The fluorescence intensity was proportional to the content of double-stranded DNA. The separation of cells in G0/G1, S, and G2/M was based upon the distribution of DNA content. Cytometric profiles of the PI-stained DNA showed that after being treated with **8e** at different concentrations (2, 5, 10 nM) for 24 h, the cell cycle of FaDu cells was arrested. As shown in Figure 7, the percentage of cells in the S phase (33.7%, 44.3%, and 54.7%) was markedly improved compared with the control group (28.1%), and this effect was concentration-dependent in FaDu cells. The population of the G0/G1 phase decreased from 51.9% to 41.8%, 44.2%, and 32.4%, respectively. These data indicated that **8e** significantly arrested cell cycle at S phase. This was different from the previously reported cell cycle arrest in the G0/G1 phase induced by TMP [4], which revealed that the dimerization of TMP could change the modulation of the cell cycle of its monomer.

### 2.9. In Silico ADMET Prediction 

**Figure 8:**
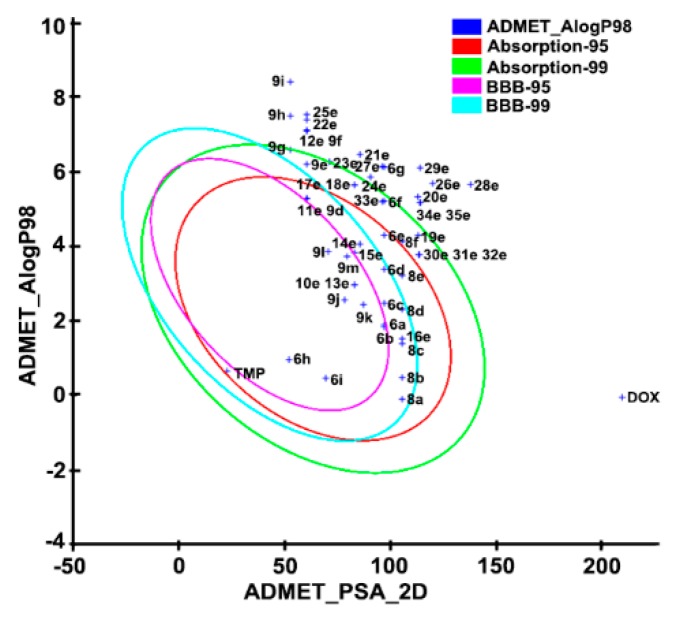
The plot of PSA (polar surface area) versus AlogP98 for the newly synthesized compounds showing the 95% and 99% confidence limit ellipses corresponding to the intestinal absorption and blood-brain barrier models.

ADMET prediction is becoming increasingly crucial as most drugs fail in clinical trials owing to poor pharmacokinetic parameters [26,27]. Table 2 shows the ADMET calculated descriptors for our synthesized compounds and two biplots shown in Figure 8. All compounds had 0–4 HBD (hydrogen bond donor) and 4–10 HBA (hydrogen bond acceptor), which were under Lipinski’s rule of five (HBD < 5, HBA < 10). PPB (plasma protein binding) was one of the key properties related to drug efficacy. Except for compounds **10**–**15e**, **17**–**24e,** and **9d,** most of the compounds had weak (<90%) binding with plasma proteins in the blood, which were predicted to be non-inhibitors of cytochrome P450 2D6, and participated in the good- metabolism of phase I metabolism. T_1/2_ of the target molecules was in the range of 1.63–2.29 h, showing the characteristic of rapid metabolism. The HT (hepatotoxicity) values estimated the hepatoxicity of the chemical compounds. HT values for series **6a**–**i**, **13**–**14e**, **20e**, **29e**, **31**–**35e**, **9f**, **9i**, **9k,** and **9m** were zero, and there was no hepatoxicity, while other compounds might be hepatotoxic. Moreover, the LD_50_ (median lethal dose) predicted that series **8a**–**f**, **9**–**35e,** and **9d**–**m** were over 500 mg/kg, implying they were less toxic. In addition, an ADMET model formed with descriptors 2D polar surface area (PSA) and AlogP98 could predict human intestinal absorption and the blood-brain barrier penetration (BBB) at 95% and 99% confidence level. In the absorption plot, compounds **6a**–**f**, **6h**–**i**, **8a**–**f**, **9**–**11e**, **13**–**19e**, **23e**, **30**–**33e**, **9d**, **9g,** and **9j**–**m** were predicted to be easily absorbed by the intestine at 99% confidence level. While in the BBB plot, the most potent compounds **8e** and **8f** were fallen out of the 99% ellipse (undefined), suggesting its poor blood-brain barrier penetration ability. Therefore, these molecules might possess low or no side effects of the central nervous system (CNS). In conclusion, ADMET prediction of the compounds might be helpful for further designing new drugs with favorable oral bioavailability.

## 3. Conclusions

Using ligustrazine, aromatic, or heteroaromatic acids as the main raw materials and linked by alkyl or ether chain, bivalent antitumor agents were synthesized and screened from five different human cancer cell lines and one normal mammary epithelial cell line. SAR revealed that the two terminal amide bonds and the alky chain were the dominant factors with 10 C atom chain showing the most potent antitumor effect. Among different aromatic moieties, the ligustrazine dimer **8e** was the best candidate; its IC_50_ was 1.36 nM in FaDu cell, it showed a broad-spectrum antitumor activity with IC_50_ in the range of 0.00136–1.42 μM, and the selective ratio MCF 10A/FaDu was 34.56. The subsequent fluorescence staining and flow cytometry analysis indicated that **8e** could induce apoptosis through depolarization of the mitochondrial membrane potential in FaDu cells. Further mechanism investigation showed that **8e** could arrest cell cycle at the S phase in FaDu cells. Furthermore, ADMET predicted that most of the potent compounds followed Lipinski’s law and became ‘drug-like’ molecules, further confirming that the dimerization of ligustrazine could improve the antitumor activity of its monomer. The highly selective inhibition of these dimers on the proliferation of FaDu cells might be related to the effect on a specific target in FaDu cells. Based on the characteristics of FaDu cells, further targeted screening would give insight into the selectivity of such potent compounds.

## 4. Materials and Methods

### 4.1. Chemistry

^1^H-NMR and ^13^C-NMR spectra were recorded on a Mercury Vx-300 (300 MHz) or AVANCE III (400 MHz). Chemical shifts were reported in ppm (*δ*) using the residue solvent line as the internal standard (for TMS: 0 ppm, ^1^H and ^13^C; for CHCl_3_-*d*: 7.26 ppm, ^1^H and 77.16 ppm, ^13^C). Coupling constants were given in Hertz (*J*). High-resolution MS (HRMS) were recorded on an Agilent 6520 Q-TOF LC/MS. Melting points were uncorrected and were determined on a digital melting point apparatus (Shenguang WRS-1B, shanghai, China). Flash column chromatography was carried out by using silica gel (200–300 mesh). Reactions were monitored by thin-layer chromatography (TLC) on silica gel GF254 plates (Qingdao Haiyang Chemical Plant, Qingdao, China). Reagents and solvents were used as purchased from commercial sources without further purification. The purity of the synthesized compounds was over 95% analyzed by HPLC (Waters 2695 Alliance system, Waters Corp., Milford, MA, USA), with the Kromasil C18 column eluted by methanol/water (80/20) containing 0.1% trifluoroacetic acid at a flow rate of 1 mL/min. Compounds **4** and **5** were prepared, as reported previously [19]. ^1^H-NMR, ^13^C-NMR and HR-MS spectra of compounds **6a**–**9m** can be seen in supplementary materials.

### 4.2. General Synthetic Procedure for ***6a**–**g***

A mixture of alkane-diamine (10 mmol), TMP-Cl (50 mmol), KOH (60 mmol), and dimethylformamide (35 mL) was heated to reflux. The reaction progress was monitored via TLC. After 2 h, the reaction mixture was cooled to room temperature. Subsequently, the solid was filtered off, and the filtrate was added with H_2_O (100 mL) and extracted with ethyl acetic acid three times. The organic layers were combined and washed with H_2_O and brine, and then separated and dried over anhydrous Na_2_SO_4_ for 8 h. The solvent was removed under reduced pressure; the resulting residue was separated on flash column chromatography eluted by a mixture of petroleum ether/acetone in a volume ratio of 7:1–3:1, and further recrystallized from acetone to give pale white solid.

#### 4.2.1. N^1^,N^1^,N^2^,N^2^-Tetrakis((3,5,6-Trimethylpyrazin-2-yl)Methyl)Ethane-1,2-Diamine (**6a**)

Pale white solid, yield 28%, m.p. 207.6–208.3 °C; ^1^H-NMR (300 MHz, CDCl_3_) *δ*: 3.60 (s, 8H), 2.67 (s, 4H), 2.43 (s, 12H), 2.41 (s, 12H), 2.23 (s, 12H); ^13^C-NMR (101 MHz, DMSO) *δ*: 149.91, 149.63, 148.37, 147.85, 58.75, 52.14, 50.83, 21.61, 21.42, 20.65; HRMS (ESI) *m/z*: 597.4134 [M + H]^+^, calcd. for [C_34_H_49_N_10_]^+^ 597.4142.

#### 4.2.2. N^1^,N^1^,N^3^,N^3^-Tetrakis((3,5,6-Trimethylpyrazin-2-yl)Methyl)Propane-1,3-Diamine (**6b**)

Pale white solid, yield 27%, m.p. 113–114.3 °C; ^1^H-NMR (300 MHz, CDCl_3_) *δ*: 3.65 (s, 8H), 2.46 (s, 12H), 2.43 (s, 12H), 2.41–2.38 (m, 4H), 2.32 (s, 12H), 1.81–1.71 (m, 2H). ^13^C-NMR (101 MHz, DMSO) *δ*: 149.93, 149.53, 148.51, 147.82, 58.63, 53.36, 21.61, 21.44, 20.70; HRMS (ESI) *m/z*: 611.4286 [M + H]^+^, calcd. for [C_35_H_51_N_10_]^+^ 611.4298.

#### 4.2.3. N^1^,N^1^,N^4^,N^4^-Tetrakis((3,5,6-Trimethylpyrazin-2-yl)Methyl)Butane-1,4-Diamine (**6c**)

Pale white solid, yield 37%, m.p. 129.2–129.5 °C; ^1^H-NMR (300 MHz, CDCl_3_) *δ*: 3.62 (s, 8H), 2.44 (s, 24H), 2.40–2.37 (m, 4H), 2.30 (s, 12H), 1.37 (s, 4H); ^13^C-NMR (101 MHz, DMSO) *δ*: 150, 149.53, 148.60, 147.79, 58.54, 54.85, 24.39, 21.64, 21.47, 20.71; HRMS (ESI) *m/z*: 625.4451 [M + H]^+^, calcd. for [C_36_H_53_N_10_]^+^ 625.4455.

#### 4.2.4. N^1^,N^1^,N^6^,N^6^-Tetrakis((3,5,6-Trimethylpyrazin-2-yl)Methyl)Hexane-1,6-Diamine (**6d**)

Pale white solid, yield 43%, m.p. 146.7–147.9 °C; ^1^H-NMR (300 MHz, CDCl_3_) *δ*: 3.67 (s, 8H), 2.46–2.45 (m, 24H), 2.43–2.40 (m, 4H), 2.34 (s, 12H), 1.43 (br, 4H), 1.04 (br, 4H); ^13^C-NMR (101 MHz, DMSO) *δ*: 150.05, 149.48, 148.71, 147.77, 58.64, 54.93, 27.34, 26.56, 21.65, 21.48, 20.74; HRMS (ESI) *m/z*: 653.4765 [M + H]^+^, calcd. for [C_38_H_57_N_10_]^+^ 653.4768.

#### 4.2.5. N^1^,N^1^,N^8^,N^8^-Tetrakis((3,5,6-Trimethylpyrazin-2-yl)Methyl)Octane-1,8-Diamine (**6e**)

Pale white solid, yield 48%, m.p. 107.8–108.6 °C; ^1^H-NMR (300 MHz, CDCl_3_) *δ*: 3.67 (s, 8H), 2.45 (br, 24H), 2.42–2.38 (m, 4H), 2.34 (s, 12H), 1.48–1.40 (m, 4H), 1.05 (br, 8H); ^13^C-NMR (101 MHz, CDCl_3_) *δ*: 150.09, 149.47, 148.75, 147.76, 58.69, 54.90, 29.52, 27.46, 26.55, 21.65, 21.47, 20.73; HRMS (ESI) *m/z*: 681.5076 [M + H]^+^, calcd. for [C_40_H_61_N_10_]^+^ 681.5081.

#### 4.2.6. N^1^,N^1^,N^10^,N^10^-Tetrakis((3,5,6-Trimethylpyrazin-2-yl)Methyl)Decane-1,10-Diamine (**6f**)

Pale white solid, yield 76%, m.p. 67.4–68 °C; ^1^H-NMR (300 MHz, CDCl_3_) *δ*: 3.69 (s, 8H), 2.46 (br, 24H), 2.41–2.39 (m, 4H), 2.35 (s, 12H), 1.47 (br, 4H), 1.11 (br, 12H); ^13^C-NMR (101 MHz, DMSO) *δ*: 150.10, 149.45, 148.78, 147.76, 58.71, 54.92, 29.73, 29.52, 27.48, 26.57, 21.64, 21.47, 20.74; HRMS (ESI) *m/z*: 709.5378 [M + H]^+^, calcd. for [C_42_H_65_N_10_]^+^ 709.5394.

#### 4.2.7. N^1^,N^1^,N^12^,N^12^-Tetrakis((3,5,6-Trimethylpyrazin-2-yl)Methyl)Dodecane-1,12-Diamine (**6g**)

Pale white solid, yield 76%, m.p. 84.7–85.3 °C; ^1^H-NMR (300 MHz, CDCl_3_) *δ*: 3.69 (s, 8H), 2.46 (br, 24H), 2.44 (br, 4H), 2.35 (s, 12H), 1.47 (br, 4H), 1.15–1.12 (br, 16H); ^13^C-NMR (101 MHz, DMSO) *δ*: 150.10, 149.44, 148.78, 147.74, 58.72, 54.89, 29.75, 29.53, 27.46, 26.56, 21.64, 21.47, 20.73; HRMS (ESI) *m/z*: 737.5689 [M + H]^+^, calcd. for [C_44_H_69_N_10_]^+^ 737.5707.

### 4.3. 1,4-Bis((3,5,6-Trimethylpyrazin-2-yl)Methyl)Piperazine (***6h***)

A mixture of piperazine (0.800 g, 9.29 mmol), TMP-Cl (3.80 g, 22.29 mmol), KOH (1.56 g, 27.66 mmol), and dimethylformamide (20 mL) was heated under reflux for 2 h. The reaction was monitored by TLC. The reaction was quenched, filtered, and the filtrate was added with 100 mL H_2_O and extracted with ethyl acetate three times. The organic layers were combined, washed with H_2_O and brine, and then dried over anhydrous Na_2_SO_4_ for 8 h. The solvent was evaporated in vacuo. The resulting residue was separated on flash column chromatography with a mixture of petroleum ether/acetone 7:1–3:1(volume ratio) as eluent, and then further recrystallized from acetone to afford compound **6h**. 

Pale white solid, yield 62%, m.p. 155.8–156.3 °C; ^1^H-NMR (300 MHz, CDCl_3_) *δ*: 3.58 (s, 4H), 2.55 (s, 8H), 2.47 (br, 18H); ^13^C-NMR (101 MHz, DMSO) *δ*: 150.01, 149.57, 148.10, 147.98, 61.88, 53.26, 50.70, 21.61, 21.51, 21.04; HRMS (ESI) *m/z*: 355.2610 [M + H]^+^, calcd. for [C_20_H_31_N_6_]^+^ 355.2610.

### 4.4. 2-(Bis((3,5,6-Trimethylpyrazin-2-yl)Methyl)Amino)Ethanol (***6i***)

A mixture of ethanolamine (0.500 g, 8.19 mmol), TMP-Cl (3.35 g, 19.65 mmol), K_2_CO_3_ (3.39 g, 24.56 mmol), and acetonitrile (20 mL) was heated under reflux for 3 h. The product formation was monitored via TLC. After cooling, the mixture was filtered, and the filtrate was evaporated in vacuo. The residue was purified by flash column chromatography with an eluent of petroleum ether/acetone = 10:1–3:1 to afford compound **6i**.

Light yellow oil, yield 72%; ^1^H-NMR (300 MHz, CDCl_3_) *δ*: 3.82 (13s, 4H), 3.66 (t, *J* = 5 Hz, 2H), 2.87 (t, *J* = 5.1 Hz, 2H), 2.41 (s, 6H), 2.37 (s, 6H), 2.35 (s, 6H); ^13^C-NMR (101 MHz, DMSO) *δ*: 149.54, 148.94, 148.27, 147.87, 59.67, 58.15, 57.36, 21.41, 21.21, 20.52; HRMS (ESI) *m/z*: 330.2290 [M + H]^+^, calcd. for [C_18_H_28_N_5_O]^+^ 330.2294.

### 4.5. General Synthetic Procedure for ***8a***–***f***

Compound **7** was gained according to the method described by Wu [20]. A mixture of tetramethylpyrazine acid (4.40 mmol), EDCI (4.72 mmol), triethylamine (12 mmol), DMAP (2.40 mmol), and anhydrous dichloromethane (10 mL) was stirred to dissolve, then diamino-alkane (2 mmol) was added and stirred at room temperature until the reaction was finished (monitored by TLC). The reaction mixture was washed with brine and water, extracted with dichloromethane (20 mL), dried over anhydrous sodium sulfate for 8 h, filtered, and the solvent was evaporated. The resulting residue was purified by flash column chromatography with an eluent of petroleum ether/acetone = 15:1–3:1(volume ratio) to give pale white solid. 

#### 4.5.1. N,N′-(Propane-1,3-Diyl)Bis(3,5,6-Trimethylpyrazine-2-Carboxamide) (**8a**)

Pale white solid, yield 16%, m.p. 125.4–126.4 °C; ^1^H-NMR (300 MHz, CDCl_3_) *δ*: 8.30 (t, *J* = 5.9 Hz, 2H), 3.58–3.52 (m, 4H), 2.92 (s, 6H), 2.56 (s, 6H), 2.50 (s, 6H), 1.99–1.90 (m, 2H); ^13^C-NMR (101 MHz, CDCl3) *δ*: 165.57, 154.09, 151.29, 147.80, 139.09, 36.89, 29.87, 22.94, 22.07, 21.47; HRMS (ESI) *m/z*: 371.2189 [M + H]^+^, calcd. for [C_19_H_27_N_6_O_2_]^+^ 371.2195.

#### 4.5.2. N,N′-(Butane-1,4-Diyl)Bis(3,5,6-Trimethylpyrazine-2-Carboxamide) (**8b**)

Pale white solid, yield 30%, m.p. 168.4–168.6 °C; ^1^H-NMR (300 MHz, CDCl_3_) *δ*: 8.07 (t, *J* = 5.5 Hz, 2H), 3.50–3.48 (m, 4H), 2.91 (s, 6H), 2.56 (s, 6H), 2.52 (s, 6H), 1.75–1.73 (m, 4H); ^13^C-NMR (101 MHz, CDCl_3_) *δ*: 165.25, 154.15, 151.38, 147.75, 139.07, 50.90, 39.13, 27.49, 22.96, 22.06, 21.48; HRMS (ESI) *m/z*: 385.2352 [M + H]^+^, calcd. for [C_20_H_29_N_6_O_2_]^+^ 385.2352.

#### 4.5.3. N,N′-(Hexane-1,6-Diyl)Bis(3,5,6-Trimethylpyrazine-2-Carboxamide) (**8c**)

Pale white solid, yield 47%, m.p. 114.2–114.7 °C; ^1^H-NMR (300 MHz, CDCl_3_) *δ*: 8.02 (t, *J* = 5.4 Hz, 2H), 3.45–3.38 (m, 4H), 2.90 (s, 6H), 2.55 (s, 6H), 2.51 (s, 6H), 1.67–1.63 (m, 4H), 1.47–1.43 (m, 4H); ^13^C-NMR (101 MHz, CDCl_3_) *δ*: 165.12, 154.03, 151.35, 147.63, 139.11, 39.33, 29.74, 26.82, 22.98, 22.06, 21.48; HRMS (ESI) *m/z*: 413.2662 [M + H]^+^, calcd. for [C_22_H_33_N_6_O_2_]^+^ 413.2665.

#### 4.5.4. N,N′-(Octane-1,8-Diyl)Bis(3,5,6-Trimethylpyrazine-2-Carboxamide) (**8d**)

Pale white solid, yield 68%, m.p. 113.6–113.8 °C; ^1^H-NMR (300 MHz, CDCl_3_) *δ*: 8 (t, *J* = 5.3 Hz, 2H), 3.45–3.38 (m, 4H), 2.91 (s, 6H), 2.56 (s, 6H), 2.52 (s, 6H), 1.65–1.60 (m, 4H), 1.37–1.37 (m, 8H); ^13^C-NMR (101 MHz, CDCl_3_) *δ*: 165.10, 154.02, 151.40, 147.65, 139.20, 39.45, 29.81, 29.34, 27.11, 23.01, 22.08, 21.53; HRMS (ESI) *m/z*: 441.2975 [M + H]^+^, calcd. for [C_24_H_37_N_6_O_2_]^+^ 441.2978.

#### 4.5.5. N,N′-(Decane-1,10-Diyl)Bis(3,5,6-Trimethylpyrazine-2-Carboxamide) (**8e**)

Pale white solid, yield 66%, m.p. 110.3–110.6 °C; ^1^H-NMR (300 MHz, CDCl_3_) *δ*: 8 (t, *J* = 5.2 Hz, 2H), 3.44–3.37 (m, 4H), 2.91 (s, 6H), 2.55 (s, 6H), 2.51 (s, 6H), 1.64–1.57 (m, 4H), 1.34–1.30 (m, 12H); ^13^C-NMR (101 MHz, CDCl_3_) *δ*: 165.07, 153.97, 151.33, 147.66, 139.21, 39.47, 29.80, 29.56, 29.39, 27.14, 22.94, 22.02, 21.49; HRMS (ESI) *m/z*: 469.3287 [M + H]^+^, calcd. for [C_26_H_41_N_6_O_2_]^+^ 469.3291.

#### 4.5.6. N,N′-(Dodecane-1,12-Diyl)Bis(3,5,6-Trimethylpyrazine-2-Carboxamide) (**8f**)

Pale white solid, yield 72%, m.p. 98.3–99.1 °C; ^1^H-NMR (300 MHz, CDCl_3_) *δ*: 8 (t, *J* = 4.9 Hz, 2H), 3.45–3.38 (m, 4H), 2.91 (s, 6H), 2.56 (s, 6H), 2.52 (s, 6H), 1.67–1.57 (m, 4H), 1.34–1.27 (m, 16H); ^13^C-NMR (101 MHz, CDCl_3_) *δ*: 165.07, 153.98, 151.37, 147.62, 139.22, 39.49, 29.83, 29.66, 29.44, 27.18, 22.99, 22.07, 21.51; HRMS (ESI) *m/z*: 497.3602 [M + H]^+^, calcd. for [C_28_H_45_N_6_O_2_]^+^ 497.3604.

### 4.6. General Synthetic Procedure for ***9e*–*35e***

Compounds **9e**–**35e** were obtained by using one-pot reaction. A mixture of aromatic acid (6.30 mmol), EDCI (7.50 mmol), DMAP (0.60 mmol), and anhydrous dichloromethane (20 mL) was stirred to dissolve, then decane-diamine (3 mmol) was added and stirred at room temperature for 12 h. The mixture solution was filtered under reduced pressure. After that, the residue was washed with little amount of CH_2_Cl_2_ and water successively, and dried to give the solid. Then, the residue was purified on preparative TLC eluted with chloroform/methanol = 40:1–7:1 to yield compounds **26e**, **28e, 30e,** and **31e**.

#### 4.6.1. (2E,2′E)-N,N′-(Decane-1,10-Diyl)Bis(3-Phenylacrylamide) (**9e**)

Pale white solid, yield 62%, m.p. 169.8–170.5 °C; ^1^H-NMR (300 MHz, CDCl_3_+CD_3_OD) *δ*: 7.54 (d, *J* = 15.7 Hz, 2H), 7.49–7.46 (m, 4H), 7.36–7.31 (m, 6H), 6.47 (d, *J* = 15.6 Hz, 2H), 3.33–3.28 (m, 4H), 1.57–1.54 (m, 4H), 1.52–1.28 (m, 12H); ^13^C-NMR (101 MHz, CDCl_3_+CD_3_OD) *δ*: 150.10, 149.45, 148.78, 147.76, 58.71, 54.92, 29.73, 29.52, 27.48, 26.57, 21.64, 21.47, 20.74; HRMS (ESI) *m/z*: 433.2851 [M + H]^+^, calcd. for [C_28_H_45_N_6_O_2_]^+^ 433.2855

#### 4.6.2. N,N′-(Decane-1,10-Diyl)Dinicotinamide (**10e**)

Pale white solid, yield 67%, m.p. 167.4–167.8 °C; ^1^H-NMR (300 MHz, CDCl_3_+CD_3_OD) *δ*: 8.84 (s, 2H), 8.55 (d, *J* = 3.9 Hz, 2H), 8.11 (dt, *J* = 8, 1.7 Hz, 2H), 7.35 (dd, *J* = 7.9, 4.9 Hz, 2H), 3.34 (t, *J* = 7.2 Hz, 4H), 1.58–1.51 (m, 4H), 1.27–1.24 (m, 12H); ^13^C-NMR (101 MHz, CDCl_3_+CD_3_OD) *δ*: 165.85, 151.29, 147.53, 135.93, 130.87, 123.79, 40.16, 29.28, 29.12, 26.86; HRMS (ESI) *m/z*: 383.2441 [M + H]^+^, calcd. for [C_28_H_45_N_6_O_2_]^+^ 383.2447.

#### 4.6.3. N,N′-(Decane-1,10-Diyl)Dibenzamide (**11e**)

Pale white solid, yield 70%, m.p. 154.3–154.6 °C; ^1^H-NMR (300 MHz, CDCl_3_+CD_3_OD) *δ*: 7.77–7.73 (m, 4H), 7.51–7.33 (m, 6H), 3.42–3.35 (m, 4H), 1.63–1.56 (m, 4H), 1.34–1.30 (m, 12H); ^13^C-NMR (101 MHz, CDCl_3_+CD_3_OD) *δ*: 168.39, 134.55, 131.36, 128.45, 126.86, 40.02, 29.36, 29.27, 29.13, 26.84. HRMS (ESI) *m/z*: 381.2702 [M + H]^+^, calcd. for [C_24_H_33_N_2_O_2_]^+^ 381.2542.

#### 4.6.4. N,N′-(Decane-1,10-Diyl)Bis(2-Naphthamide) (**12e**)

Pale white solid, yield 64%, m.p. 163.9–164 °C; ^1^H-NMR (300 MHz, CDCl_3_+CD_3_OD) *δ*: 8.22–8.17 (m, 2H), 7.89–7.82 (m, 4H), 7.54–7.39 (m, 8H), 3.49–3.44 (m, 4H), 1.67–1.60 (m, 4H), 1.38–1.34 (m, 12H); ^13^C-NMR (101 MHz, CDCl_3_ + CD_3_OD) *δ*: 170.38, 134.50, 133.56, 130.33, 129.96, 128.22, 126.89, 126.29, 125.10, 124.77, 124.65, 39.94, 29.36, 29.30, 29.11, 26.85. HRMS (ESI) *m/z*: 481.2853 [M + H]*+*, calcd. for [C_32_H_37_N_2_O_2_]^+^ 481.2855.

#### 4.6.5. N,N′-(Decane-1,10-Diyl)Diisonicotinamide (**13e**)

Pale white solid, yield 60%, m.p. 175.6–176.3 °C; ^1^H-NMR (300 MHz, CDCl_3_+CD_3_OD) *δ*: 8.62 (d, *J* = 5.6 Hz, 4H), 7.71 (dd, *J* = 6.1, 2.8 Hz, 4H), 3.41–3.33 (m, 4H), 1.64–1.57 (m, 4H), 1.33–1.31 (m, 12H); ^13^C-NMR (101 MHz, CDCl_3_+CD_3_OD) *δ*: 165.88, 149.48, 142.45, 121.46, 40.04, 29.16, 28.99, 26.71. HRMS (ESI) *m/z*: 383.2445 [M + H]^+^, calcd. for [C_22_H_31_N_4_O_2_]^+^ 383.2447.

#### 4.6.6. N,N′-(Decane-1,10-Diyl)Bis(Furan-2-Carboxamide) (**14e**)

Pale white solid, yield 62%, m.p. 132.6–133.3 °C; ^1^H-NMR (300 MHz, CDCl_3_) *δ*: 7.41 (dd, *J* = 1.7, 0.8 Hz, 2H), 7.09 (dd, *J* = 3.5, 0.7 Hz, 2H), 6.48 (dd, *J* = 3.5, 1.7 Hz, 2H), 6.37 (br, 2H), 3.45–3.38 (m, 4H), 1.65–1.55 (m, 4H), 1.36–1.30 (m, 12H); ^13^C-NMR (101 MHz, CDCl_3_) *δ*: 158.53, 148.38, 143.77, 114.02, 112.22, 39.29, 29.80, 29.48, 29.33, 26.98. HRMS (ESI) *m/z*: 383.1944 [M + Na]^+^, calcd. for [C_20_H_28_N_2_NaO_4_]^+^ 383.1947.

#### 4.6.7. N,N′-(Decane-1,10-Diyl)Dipicolinamide (**15e**)

Pale white solid, yield 68%, m.p. 156.6–156.9 °C; ^1^H-NMR (300 MHz, CDCl_3_) *δ*: 9.35 (d, *J* = 1.4 Hz, 2H), 8.69 (d, *J* = 2.5 Hz, 2H),.8.47–8.46 (m, 2H), 7.77 (br, 2H), 3.47–3.40 (m, 4H), 1.65–1.55 (m, 4H), 1.34–1.26 (m, 12H); ^13^C-NMR (101 MHz, CDCl_3_) *δ*: 163.01, 147.26, 144.76, 144.55, 142.58, 39.61, 29.68, 29.51, 29.34, 27.03. HRMS (ESI) *m/z*: 383.2441 [M + H]^+^, calcd. for [C_22_H_31_N_4_O_2_]^+^ 383.2447

#### 4.6.8. N,N′-(Decane-1,10-Diyl)Bis(Pyrazine-2-Carboxamide) (**16e**)

Pale white solid, yield 70%, m.p. 77.6–80.1 °C; ^1^H-NMR (300 MHz, CDCl_3_) *δ*: 8.54–8.51 (m, 2H), 8.20–8.17 (m, 2H), 8.05 (br, 2H), 7.85–7.80 (m, 2H), 7.42–7.38 (m, 2H), 3.49–3.43 (m, 4H), 1.68–1.58 (m, 4H), 1.38–1.26 (m, 12H); ^13^C-NMR (101 MHz, CDCl_3_) *δ*: 164.31, 150.25, 148.09, 137.49, 126.13, 122.34, 39.61, 29.77, 29.56, 29.41, 27.11. HRMS (ESI) *m/z*: 385.2350 [M + H]^+^, calcd. for [C_20_H_29_N_6_O_2_]^+^ 385.2352.

#### 4.6.9. N,N′-(Decane-1,10-Diyl)Bis(Quinoline-3-Carboxamide) (**17e**)

Pale white solid, yield 59%, m.p. 206.7–207.1 °C; ^1^H-NMR (300 MHz, DMSO-*d*_6_ + CD_3_OD) *δ*: 8.57 (d, *J* = 1.8 Hz, 2H), 8.09 (s, 2H), 7.39 (dd, *J* = 8.3, 3.1 Hz, 4H), 7.17 (t, *J* = 7.6 Hz, 2H), 7 (t, *J* = 7.4 Hz, 2H), 2.68 (t, *J* = 7 Hz, 4H), 0.99–0.88 (m, 4H), 0.76–0.62 (m, 12H). ^13^C-NMR (101 MHz, CDCl_3_) *δ*: 165.92, 148.01, 136.52, 131.47, 128.81, 127.93, 127.56, 127.35, 127.06, 40.11, 29.20, 29.15, 29.04, 26.78. HRMS (ESI) *m/z*: 483.2756 [M + H]^+^, calcd. for [C_30_H_35_N_4_O_2_]^+^ 483.2760. 

#### 4.6.10. N,N′-(Decane-1,10-Diyl)Bis(Quinoline-6-Carboxamide) (**18e**)

Pale yellow solid, yield 73%, m.p. 197.7–198.3 °C; ^1^H-NMR (300 MHz, CDCl_3_ + CD_3_OD) *δ*: 8.71 (dd, *J* = 4.3, 1.6 Hz, 2H), 8.19 (d, *J* = 1.6 Hz, 2H), 8.16 (dd, *J* = 8.3, 1.3 Hz, 2H), 7.95 (dd, *J* = 8.8, 1.9 Hz, 2H), 7.91 (s, 2H), 7.35 (dd, *J* = 8.3, 4.3 Hz, 2H), 3.28 (t, *J* = 7.2 Hz, 4H), 1.53–1.45 (m, 5H), 1.22–1.13 (m, 12H). ^13^C-NMR (101 MHz, CDCl_3_ + CD_3_OD) *δ*: 167.43, 151.15, 148.21, 137.99, 132.82, 128.38, 127.79, 127.71, 127.64, 121.81, 40.14, 29.19, 29.04, 26.78. HRMS (ESI) *m/z*: 483.2758 [M + H]^+^, calcd. for [C_30_H_35_N_4_O_2_]^+^ 483.2760. 

#### 4.6.11. N,N′-(Decane-1,10-Diyl)Bis(1H-Benzo[d]Imidazole-6-Carboxamide) (**19e**)

Brick red solid, yield 75%, m.p. 229.7–230.3 °C; ^1^H-NMR (300 MHz, CDCl_3_+ CD_3_OD) *δ*: 8.11 (s, 2H), 8.07 (s, 2H), 7.73 (dd, *J* = 8.5, 1.6 Hz, 2H), 7.61 (d, *J* = 8.5 Hz, 2H), 3.42 (t, *J* = 7.1 Hz, 4H), 1.70–1.56 (m, 4H), 1.42–1.25 (m, 12H). ^13^C-NMR (101 MHz, CDCl_3_+ CD_3_OD) *δ*: 168.61, 142.46, 128.70, 121.15, 39.32, 28.66, 28.62, 28.50, 26.19. HRMS (ESI) *m/z*: 461.2662 [M + H]^+^, calcd. for [C_26_H_33_N_6_O_2_]^+^ 461.2665. 

#### 4.6.12. N,N′-(Decane-1,10-Diyl)Bis(2-Oxo-2H-Chromene-3-Carboxamide) (**20e**)

Pale white solid, yield 60%, m.p. 208.9–209.3 °C; ^1^H-NMR (300 MHz, CDCl_3_) *δ*: 8.90 (s, 2H), 8.80 (s, 2H), 7.71–7.61 (m, 4H), 7.43–7.32 (m, 4H), 3.45 (dd, *J* = 13, 7 Hz, 4H), 1.63 (dt, *J* = 14.4, 7.2 Hz, 4H), 1.44–1.26 (m, 12H). ^13^C-NMR (101 MHz, CDCl_3_ + CD_3_OD) *δ*: 161.74, 161.44, 154.23, 148.38, 134.16, 129.80, 125.30, 118.46, 117.97, 116.38, 39.69, 29.20, 29.02, 29.01, 26.77. HRMS (ESI) *m/z*: 517.2338 [M + H]^+^, calcd. for [C_30_H_33_N_2_O_6_]^+^ 517.2339.

#### 4.6.13. N,N′-(Decane-1,10-Diyl)Bis(Benzofuran-2-Carboxamide) (**21e**)

Pale white solid, yield 43%, m.p. 135.8–136.3 °C; ^1^H-NMR (300 MHz, DMSO-*d*_6_) *δ*: 8.64 (s, 2H), 7.74 (d, *J* = 7.7 Hz, 2H), 7.62 (d, *J* = 8.2 Hz, 2H), 7.49 (s, 2H), 7.43 (t, *J* = 7.5 Hz, 2H), 7.30 (t, *J* = 7.4 Hz, 2H), 3.25 (dd, *J* = 13.1, 6.6 Hz, 4H), 1.58–1.43 (m, 4H), 1.37–1.14 (m, 12H). ^13^C-NMR (101 MHz, DMSO-*d*_6_) *δ*: 157.94, 154.13, 149.37, 127.17, 126.61, 123.59, 122.63, 111.70, 109.02, 38.68, 29.01, 28.91, 28.71, 26.39. HRMS (ESI) *m/z*: 461.2438 [M + H]^+^, calcd. for [C_28_H_33_N_2_O_4_]^+^ 461.2430.

#### 4.6.14. N,N′-(Decane-1,10-Diyl)Bis(Benzo[b]Thiophene-2-Carboxamide) (**22e**)

Pale yellow solid, yield 46%, m.p. 175–175.7 °C; ^1^H-NMR (300 MHz, DMSO-*d*_6_) *δ*: 8.68 (s, 2H), 8.04 (s, 2H), 8.01–7.95 (m, 2H), 7.93–7.87 (m, 2H), 7.41 (p, *J* = 7 Hz, 4H), 3.25 (dd, *J* = 12.8, 6.6 Hz, 4H), 1.58–1.44 (m, 4H), 1.37–1.21 (m, 12H). ^13^C-NMR (101 MHz, DMSO-*d*_6_) *δ*: 161.28, 140.28, 140.07, 139.16, 126, 125.02, 124.80, 124.33, 122.72, 28.99, 28.92, 28.72, 26.43. HRMS (ESI) *m/z*: 493.1976 [M + H]^+^, calcd. for [C_28_H_33_N_2_O_2_S_2_]^+^ 493.1983.

#### 4.6.15. N,N′-(Decane-1,10-Diyl)Bis(1-Methyl-1H-Indole-3-Carboxamide) (**23e**)

Pale white solid, yield 43%, m.p. 192.8–193.2 °C; ^1^H-NMR (300 MHz, DMSO-*d*_6_ + CD_3_OD) *δ*: 8.08 (d, *J* = 7.7 Hz, 2H), 7.86 (s, 2H), 7.41 (d, *J* = 8 Hz, 2H), 7.20–7.13 (m, 2H), 7.13–7.06 (m, 2H), 3.78 (s, 6H), 3.22 (t, *J* = 7.1 Hz, 4H), 1.55–1.43 (m, 4H), 1.32–1.22 (m, 12H). ^13^C-NMR (101 MHz, CDCl_3_ + CD_3_OD) *δ*: 166.26, 137.01, 131.75, 125.56, 122.27, 121.03, 120.13, 109.63, 39.26, 32.62, 29.33, 29.07, 28.94, 26.66. HRMS (ESI) *m/z:* 487.3064 [M + H]^+^, calcd. for [C_30_H_39_N_4_O_2_]^+^ 487.3073. 

#### 4.6.16. N,N′-(Decane-1,10-Diyl)Bis(1H-Indole-6-Carboxamide) (**24e**)

Pale white solid, yield 47%, m.p. 168.4–169.2 °C; ^1^H-NMR (300 MHz, DMSO-*d*_6_) *δ*: 11.32 (s, 2H), 8.30 (s, 2H), 7.92 (s, 2H), 7.59–7.41 (m, 6H), 6.46 (s, 2H), 3.25 (dd, *J* = 12.7, 6.4 Hz, 4H), 1.61–1.43 (m, 4H), 1.38–1.21 (m, 12H). ^13^C-NMR (101 MHz, DMSO) *δ*: 167.04, 135.24, 129.61, 127.74, 127.66, 119.27, 117.90, 111.07, 101.14, 39.21, 29.27, 29, 28.83, 26.55. HRMS (ESI) *m/z*: 459.2760 [M + H]^+^, calcd. for [C_28_H_35_N_4_O_2_]^+^ 459.2760.

#### 4.6.17. (2E,2′E)-N,N′-(Decane-1,10-Diyl)Bis(3-(4-Chlorophenyl)Acrylamide) (**25e**)

Pale white solid, yield 64%, m.p. 230.3–230.9 °C; ^1^H-NMR (300 MHz, CDCl_3_ +CF_3_COOD) *δ*: 7.64 (d, *J* = 13 Hz, 2H), 7.54–7.38 (m, 8H), 6.51 (d, *J* = 11.9 Hz, 2H), 3.62–3.37 (m, 4H), 1.73–1.54 (m, 4H), 1.46–1.18 (m, 12H). ^13^C-NMR (101 MHz, CDCl_3_ + CF_3_COOD) *δ*: 129.71, 118.78, 115.96, 113.13, 110.30, 29.22, 28.99, 26.64. HRMS (ESI) *m/z*: 501.2075 [M + H]^+^, calcd. for [C_28_H_35_Cl_2_N_2_O_2_]^+^ 501.2076.

#### 4.6.18. (2E,2′E)-N,N′-(Decane-1,10-Diyl)Bis(3-(3-Hydroxy-4-Methoxyphenyl)Acrylamide) (**26e**)

Pale yellow solid, yield 35%, m.p. 192.9–195.3 °C; ^1^H-NMR (300 MHz, CDCl_3_ + CD_3_OD) *δ*: 7.42 (d, *J* = 15.3 Hz, 2H), 7.05 (s, 2H), 6.98 (d, *J* = 8.1 Hz, 2H), 6.83 (d, *J* = 8.3 Hz, 2H), 6.33 (d, *J* = 15.6 Hz, 2H), 3.89 (s, 6H), 3.29 (t, *J* = 7 Hz, 4H), 1.55–1.50 (m, 4H), 1.31–1.27 (m, 12H). ^13^C-NMR (101 MHz, CDCl_3_ + CD_3_OD) *δ*: 167.37, 148.94, 146.05, 140.42, 128.11, 121.06, 118.25, 113.07, 111, 55.57, 39.50, 29.13, 28.98, 26.69. HRMS (ESI) *m/z*: 525.2951 [M + H]^+^, calcd. for [C_30_H_41_N_2_O_6_]^+^ 525.2965.

#### 4.6.19. (2E,2′E)-N,N′-(Decane-1,10-Diyl)Bis(3-(3,4-Dimethoxyphenyl)Acrylamide) (**27e**)

Pale white solid, yield 59%, m.p. 168.9–169.9 °C; ^1^H-NMR (300 MHz, DMSO-*d*_6_) *δ*: 7.95 (br, 2H), 7.30 (br, 2H), 7.11 (br, 4H), 6.97 (br, 2H), 6.50 (br, 2H), 3.78 (br, 12H), 3.14 (br, 4H), 1.42 (br, 4H), 1.27 (br, 12H). ^13^C-NMR (101 MHz, CDCl_3_+ CD_3_OD) *δ*: 167.15, 150.31, 148.90, 140.25, 127.89, 121.77, 118.56, 111.04, 109.74, 55.66, 55.58, 39.47, 29.16, 29.14, 28.99, 26.69. HRMS (ESI) *m/z:* 553.3272 [M + H]^+^, calcd. for [C_32_H_45_N_2_O_6_]^+^ 553.3278.

#### 4.6.20. (2E,2′E)-N,N′-(Decane-1,10-Diyl)Bis(3-(4-Hydroxy-3,5-Dimethoxyphenyl)Acrylamide) (**28e**)

Pale yellow solid, yield 33%, m.p. 179.8–180.5 °C; ^1^H-NMR (300 MHz, CDCl_3_ + CD_3_OD) *δ*: 7.44 (d, *J* = 15.5 Hz, 3H), 6.76 (s, 4H), 6.37 (d, *J* = 15.6 Hz, 2H), 3.88 (s, 12H), 3.30 (t, *J* = 7.1 Hz, 4H), 1.61–1.49 (m, 4H), 1.38–1.24 (m, 12H). ^13^C-NMR (101 MHz, CDCl_3_ + CD_3_OD) *δ*: 166.94, 147.48, 141.03, 136.87, 126.16, 118.46, 104.99, 56.23, 39.63, 29.39, 29.21, 29.08, 26.79. HRMS (ESI) *m/z*: 585.3164 [M+H]^+^, calcd. for [C_32_H_45_N_2_O_8_]^+^ 585.3176

#### 4.6.21. (2E,2′E)-N,N′-(Decane-1,10-Diyl)Bis(3-(3,4,5-Trimethoxyphenyl)Acrylamide) (**29e**)

Pale white solid, yield 67%, m.p. 193.4–194.3 °C; ^1^H-NMR (300 MHz, CDCl_3_ + CD_3_OD) *δ*: 7.45 (d, *J* = 15.4 Hz, 2H), 6.76 (s, 4H), 6.44 (d, *J* = 15.6 Hz, 2H), 3.88 (s, 12H), 3.85 (s, 6H), 3.31 (t, *J* = 7.1 Hz, 4H), 1.63–1.47 (m, 4H), 1.40–1.23 (m, 12H). ^13^C-NMR (101 MHz, CDCl_3_ + CD_3_OD) *δ*: 166.73, 153.26, 140.53, 139.33, 130.65, 120.18, 104.99, 60.81, 56, 39.63, 29.28, 29.21, 29.05, 26.76. HRMS (ESI) *m/z*:613.3472 [M + H]^+^, calcd. for [C_34_H_49_N_2_O_8_]^+^ 613.3489.

#### 4.6.22. N,N′-(Decane-1,10-Diyl)Bis(2-Aminobenzamide) (**30e**)

Pale white solid, yield 67%, m.p. 90.2–91 °C; ^1^H-NMR (300 MHz, CDCl_3_ + CD_3_OD) *δ*: 7.36 (dd, *J* = 7.8, 1.2 Hz, 2H), 7.22–7.15 (m, 2H), 6.79–6.60 (m, 4H), 3.35 (dt, *J* = 9.7, 5.5 Hz, 4H), 1.66–1.49 (m, 4H), 1.41–1.24 (m, 12H). ^13^C-NMR (101 MHz, CDCl_3_ + CD_3_OD) *δ*: 173.92, 151.77, 135.96, 131.40, 121.48, 121.28, 120.96, 43.54, 33.31, 33.26, 33.11, 30.83. HRMS (ESI) *m/z*:411.2759 [M + H]^+^, calcd. for [C_24_H_35_N_4_O_2_]^+^ 411.2760.

#### 4.6.23. N,N′-(Decane-1,10-Diyl)Bis(4-Aminobenzamide) (**31e**)

Pale yellow solid, yield 31%, m.p. 186.7–187.7 °C; ^1^H-NMR (300 MHz, CDCl_3_ + CD_3_OD) *δ*: 7.57 (d, *J* = 8.6 Hz, 4H), 6.66 (d, *J* = 8.6 Hz, 4H), 3.34 (t, *J* = 7.2 Hz, 4H), 1.63–1.53 (m, 4H), 1.31–1.26 (m, 12H). ^13^C-NMR (101 MHz, CDCl_3_ + CD_3_OD) *δ*: 168.44, 150.01, 128.49, 123.39, 114.04, 39.77, 29.33, 29.18, 29.06, 26.76. HRMS (ESI) *m/z*: 411.2755 [M + H]^+^, calcd. for [C_24_H_35_N_4_O_2_]^+^ 411.2760.

#### 4.6.24. N,N′-(Decane-1,10-Diyl)bis(3-Aminobenzamide) (**32e**)

Pale white solid, yield 57%, m.p. 135.7–136.3 °C; ^1^H-NMR (300 MHz, CDCl_3_ + CD_3_OD) *δ*: 7.30–6.97 (m, 6H), 6.83 (dd, *J* = 20.8, 7.2 Hz, 2H), 3.36 (t, *J* = 6.8 Hz, 4H), 1.67–1.47 (m, 4H), 1.46–1.06 (m, 12H). ^13^C-NMR (101 MHz, CDCl_3_ + CD_3_OD) *δ*: 168.75, 146.84, 135.56, 129.27, 118.09, 116.55, 113.73, 77.48, 77.16, 76.84, 49.31, 49.09, 48.88, 48.67, 48.45, 39.93, 29.29, 29.22, 29.09, 26.79. HRMS (ESI) *m/z*: 411.2758 [M + H]^+^, calcd. for [C_24_H_35_N_4_O_2_]^+^ 411.2760

#### 4.6.25. N,N′-(Decane-1,10-Diyl)Bis(3,5-Dimethoxybenzamide) (**33e**)

Pale white solid, yield 56%, m.p. 143.4–144.2 °C; ^1^H-NMR (300 MHz, CDCl_3_ + CD_3_OD) *δ*: 6.79 (d, *J* = 2.1 Hz, 4H), 6.43 (t, *J* = 1.9 Hz, 2H), 3.69 (s, 12H), 3.25 (t, *J* = 7.2 Hz, 4H), 1.50–1.45 (m, 4H), 1.25–1.15 (m, 12H). ^13^C-NMR (101 MHz, CDCl_3_ + CD_3_OD) *δ*: 168.01, 160.73, 136.74, 104.94, 103.34, 55.40, 40.03, 29.28, 29.26, 29.11, 26.82. HRMS (ESI) *m/z:* 501.2959 [M + H]^+^, calcd. for [C_28_H_41_N_2_O_6_]^+^ 501.2965.

#### 4.6.26. N,N′-(Decane-1,10-Diyl)Bis(3,4,5-Trimethoxybenzamide) (**34e**)

Pale white solid, yield 63%, m.p. 194–194.9 °C; ^1^H-NMR (300 MHz, CDCl_3_ + CD_3_OD) *δ*: 6.91 (s, 4H), 3.72 (s, 12H), 3.67 (s, 6H), 3.22–3.16 (m, 4H), 1.50–1.36 (m, 4H), 1.22–1.08 (m, 12H). ^1^H-NMR (300 MHz, CDCl_3_ + CD_3_OD). *δ*: 6.91 (s, 4H), 3.72 (s, 12H), 3.67 (s, 6H), 3.22–3.16 (m, 4H), 1.50–1.36 (m, 4H), 1.22–1.08 (m, 12H). ^13^C-NMR (101 MHz, CDCl_3_ + CD_3_OD) *δ*: 167.81, 152.77, 129.84, 104.42, 60.53, 55.85, 40.01, 29.17, 29.03, 26.73. HRMS (ESI) *m/z:* 561.3169 [M + H]^+^, calcd. for [C_30_H_45_N_2_O_8_]^+^ 561.3176.

#### 4.6.27. N,N′-(Decane-1,10-Diyl)Bis(2,3,4-Trimethoxybenzamide) (**35e**)

Pale white solid, yield 26%, m.p. 112.3–112.5 °C; ^1^H-NMR (300 MHz, CDCl_3_) *δ*: 7.94 (s, 2H), 7.89 (d, *J* = 8.9 Hz, 2H), 6.76 (d, *J* = 9 Hz, 2H), 3.96 (s, 6H), 3.90 (s, 6H), 3.87 (s, 6H), 3.44 (dd, *J* = 12.7, 6.9 Hz, 4H), 1.62 (dt, *J* = 14.3, 7.1 Hz, 4H), 1.41–1.29 (m, 12H). ^13^C-NMR (101 MHz, CDCl_3_) *δ*: 164.90, 156.35, 152.42, 141.83, 126.76, 119.31, 107.67, 61.71, 61.08, 56.15, 39.71, 29.78, 29.65, 29.43, 27.24. HRMS (ESI) *m/z:* 561.3172 [M + H]^+^, calcd. for [C_30_H_45_N_2_O_8_]^+^ 561.3176.

### 4.7. General Synthetic Procedure for ***9d***, ***9f***

A mixture of cinnamic acid (6.30 mmol), EDCI (7.50 mmol), DMAP (0.60 mmol), and anhydrous dichloromethane (20 mL) was stirred to dissolve, then diamino-alkane (3 mmol) was added and stirred at room temperature for 12 h. The mixture solution was filtered under reduced pressure. After that, the residue was washed with dichloromethane and water successively, and then dried to give a pale white solid. TLC indicated that it was a single point.

#### 4.7.1. (2E,2′E)-N,N′-(Octane-1,8-Diyl)Bis(3-Phenylacrylamide) (**9d**)

Pale white solid, yield 63%, m.p. 186–186.4 °C; ^1^H-NMR (300 MHz, CDCl_3_ + CD_3_OD) *δ*: 7.58–7.29 (m, 12H), 6.53 (d, *J* = 15.7 Hz, 2H), 3.31 (t, *J* = 7.1 Hz, 4H), 1.59–1.55 (m, 4H), 1.35 (s, 8H). ^13^C-NMR (101 MHz, CDCl_3_ + CD_3_OD) *δ*: 166.96, 140.39, 134.78, 129.42, 128.59, 127.54, 120.60, 39.43, 29.04, 28.85, 26.57. HRMS (ESI) *m/z:* 405.2537 [M + H]^+^, calcd. for [C_26_H_33_N_2_O_2_]^+^ 405.2542.

#### 4.7.2. (2E,2′E)-N,N′-(Dodecane-1,12-Diyl)Bis(3-Phenylacrylamide) (**9f**)

Pale white solid, yield 49%, m.p. 164.7–165 °C; ^1^H-NMR (300 MHz, CDCl_3_ + CD_3_OD) *δ*: 7.56–7.49 (m, 6H), 7.40–7.28 (m, 6H), 6.53 (d, *J* = 15.7 Hz, 2H), 3.30 (t, *J* = 7.1 Hz, 4H), 1.62–1.51 (m, 4H), 1.38–1.23 (m, 16H). ^13^C-NMR (101 MHz, CDCl_3_ + CD_3_OD) *δ*: 166.94, 140.22, 134.73, 129.29, 128.47, 127.42, 120.54, 39.42, 29.19, 29.03, 28.99, 26.66. HRMS (ESI) *m/z:* 461.3166 [M + H]^+^, calcd. for [C_30_H_41_N_2_O_2_]^+^ 461.3168.

### 4.8. General Synthetic Procedure for ***9g*–*9i***

A mixture of cinnamic acid (6.30 mmol), EDCI (7.50 mmol), DMAP (0.60 mmol), and anhydrous dichloromethane (20 mL) was stirred to dissolve, then alkane-diol (3 mmol) was added and stirred at room temperature for 12 h. The mixture solution was filtered under reduced pressure. After that, the residue was washed with dichloromethane and water successively, subsequently, purified by preparative TLC eluted with petroleum ether/ethyl acetate = 5:1 to give pale white solid.

#### 4.8.1. Octane-1,8-Diyl (2E,2′E)-Bis(3-Phenylacrylate) (**9g**)

Pale white solid, yield 35%, m.p. 61.1–62.5 °C; ^1^H-NMR (300 MHz, CDCl_3_) *δ*: 7.68 (d, *J* = 16 Hz, 2H), 7.58–7.46 (m, 4H), 7.43–7.30 (m, 6H), 6.45 (d, *J* = 16 Hz, 2H), 4.21 (t, *J* = 6.7 Hz, 4H), 1.77–1.68 (m, 4H), 1.46–1.40 (m, 8H). ^13^C-NMR (101 MHz, CDCl_3_) *δ*: 167.22, 144.72, 134.62, 130.35, 129.01, 128.19, 118.43, 64.79, 29.31, 28.85, 26.05. HRMS (ESI) *m/z:* 407.2223 [M + H]^+^, calcd. for [C_26_H_31_O_4_]^+^ 407.2222.

#### 4.8.2. Decane-1,10-Diyl (2E,2′E)-Bis(3-Phenylacrylate) (**9h**)

Pale white solid, yield 33%, m.p. 118.6–119.2 °C; ^1^H-NMR (300 MHz, CDCl_3_) *δ*: 7.68 (d, *J* = 16 Hz, 2H), 7.57–7.48 (m, 4H), 7.38 (dd, *J* = 6.6, 3.6 Hz, 6H), 6.45 (d, *J* = 16 Hz, 2H), 4.21 (t, *J* = 6.7 Hz, 4H), 1.77–1.62 (m, 4H), 1.48–1.25 (m, 12H). ^13^C-NMR (101 MHz, CDCl_3_) *δ*: 167.23, 144.69, 134.64, 130.35, 129.01, 128.19, 118.46, 64.85, 29.58, 29.40, 28.88, 26.12. HRMS (ESI) *m/z:* 435.2534 [M + H]^+^, calcd. for [C_28_H_35_O_4_]^+^ 435.2535.

#### 4.8.3. Dodecane-1,12-Diyl (2E,2′E)-Bis(3-Phenylacrylate) (**9i**)

Pale white solid, yield 28%, m.p. 63.1–64 °C; ^1^H-NMR (300 MHz, CDCl_3_) *δ*: 7.68 (d, *J* = 16 Hz, 2H), 7.52 (dt, *J* = 4.6, 3.2 Hz, 4H), 7.42–7.33 (m, 6H), 6.45 (d, *J* = 16 Hz, 2H), 4.21 (t, *J* = 6.7 Hz, 4H), 1.77–1.65 (m, 4H), 1.43–1.25 (m, 16H). ^13^C-NMR (101 MHz, CDCl_3_) *δ*: 167.22, 144.68, 134.64, 130.34, 129.01, 128.18, 118.47, 64.87, 29.68, 29.66, 29.42, 28.87, 26.12. HRMS (ESI) *m/z:* 463.2851 [M + H]^+^, calcd. for [C_30_H_39_O_4_]^+^ 463.2828.

### 4.9. General Synthetic Procedure for ***9j*–*9m***

A mixture of cinnamic acid (6.30 mmol), EDCI (7.50 mmol), DMAP (0.60 mmol), and anhydrous dichloromethane (20 mL) was stirred to dissolve, then diamino-ether (3 mmol) was added and stirred at room temperature for 12 h. Then, the solid was filtered off, the filtrate was added with H_2_O (50 mL) and extracted by dichloromethane three times. The combined organic layers were washed with H_2_O and brine, dried with anhydrous sodium sulfate for 8 h, filtered, and evaporated. The resulting residue was purified by preparative TLC with petroleum ether/ethyl acetate = 2:1–1:1 to give pale white solid or light yellow oil.

#### 4.9.1. (2E,2′E)-N,N′-((Ethane-1,2-Diylbis(Oxy))Bis(Ethane-2,1-Diyl))Bis(3-Phenylacrylamide) (**9j**)

Pale white solid, yield 78%, m.p. 117.9–118.5 °C; ^1^H-NMR (300 MHz, CDCl_3_) *δ*: 7.62 (d, *J* = 15.6 Hz, 2H), 7.51–7.41 (m, 4H), 7.37–7.27 (m, 6H), 6.48 (d, *J* = 15.6 Hz, 2H), 3.65–3.60 (m, 12H). ^13^C-NMR (101 MHz, CDCl_3_) *δ*: 166.26, 141.19, 134.95, 129.77, 128.92, 127.92, 120.82, 70.50, 70.05, 39.63. HRMS (ESI) *m/z*: 409.2121 [M + H]^+^, calcd. for [C_24_H_29_N_2_O_4_]^+^ 409.2127.

#### 4.9.2. (2E,2′E)-N,N′-(((Oxybis(Ethane-2,1-Diyl))Bis(Oxy))Bis(Ethane-2,1-Diyl))Bis(3-Phenylacrylamide) (**9k**)

Light yellow oil, yield 28%; ^1^H-NMR (300 MHz, CDCl_3_) *δ*: 7.60 (d, *J* = 15.6 Hz, 2H), 7.45 (dd, *J* = 6.5, 3.1 Hz, 4H), 7.32–7.30 (m, 4H), 6.71 (t, *J* = 5.7 Hz, 2H), 6.47 (d, *J* = 15.6 Hz, 2H), 3.65 (s, 8H), 3.63–3.54 (m, 8H). ^13^C-NMR (101 MHz, CDCl_3_) *δ*: 166.17, 140.96, 134.98, 129.70, 128.89, 127.86, 120.93, 70.49, 70.27, 70, 39.59. HRMS (ESI) *m/z:* 453.2387 [M + H]^+^, calcd. for [C_26_H_33_N_2_O_5_]^+^ 453.2389.

#### 4.9.3. (Ethane-1,2-Diylbis(Oxy))Bis(Ethane-2,1-Diyl) (2E,2′E)-Bis(3-Phenylacrylate) (**9l**)

Light yellow oil, yield 65%; ^1^H-NMR (300 MHz, CDCl_3_) *δ*: 7.70 (d, *J* = 16 Hz, 2H), 7.54–7.48 (m, 4H), 7.40–7.34 (m, 6H), 6.48 (d, *J* = 16 Hz, 2H), 4.42–4.36 (m, 4H), 3.84–3.77 (m, 4H), 3.73 (s, 4H). ^13^C-NMR (101 MHz, CDCl_3_) *δ*: 167.03, 145.21, 134.50, 130.44, 129.01, 128.23, 118, 70.79, 69.48, 63.78. HRMS (ESI) *m/z:* 411.1805 [M + H]^+^, calcd. for [C_24_H_27_O_6_]^+^ 411.1808. 

#### 4.9.4. ((Oxybis(Ethane-2,1-Diyl))Bis(Oxy))Bis(Ethane-2,1-Diyl) (2E,2′E)-bis(3-Phenylacrylate) (**9m**)

Light yellow oil, yield 87%; ^1^H-NMR (300 MHz, CDCl_3_) *δ*: 7.70 (d, *J* = 16 Hz, 2H), 7.55–7.48 (m, 4H), 7.38 (dd, *J* = 3.8, 2.7 Hz, 6H), 6.48 (d, *J* = 16 Hz, 2H), 4.37 (dd, *J* = 5.5, 4.1 Hz, 4H), 3.81–3.74 (m, 4H), 3.70 (s, 8H). ^13^C-NMR (101 MHz, CDCl_3_) *δ*: 167.03, 145.19, 134.52, 130.45, 129.02, 128.23, 118.04, 77.48, 77.36, 77.16, 76.84, 70.82, 70.79, 69.42, 63.80. HRMS (ESI) *m/z*: 472.2337 [M + NH_4_]^+^, calcd. for [C_26_H_34_NO_7_]^+^ 472.2335.

### 4.10. Biological Assays

#### 4.10.1. Cell Culture

The tested cells were obtained from Shanghai Institute of Biochemistry and Cell Biology of the Chinese Academy of Sciences (Shanghai, China). HeLa and Hep G2 cell lines were cultured in Dulbecco’s modified Eagle’s medium (DMEM; gibco, Life Technologies, Carlsbad, CA, USA) supplemented with 10% fetal bovine serum (FBS; Biological Industries), 100 U/mL penicillin-streptomycin (PS; gibco, Life Technologies, Carlsbad, CA, USA). MCF-7 and A549 cell lines were maintained in RPMI-1640 media (gibco, Life Technologies, Carlsbad, CA, USA) supplemented with 10% FBS and 100 U/mL PS. FaDu cell lines were cultured in minimum essential medium (MEM; Gibco, Life Technologies, Carlsbad, CA, USA) supplemented with 10% FBS and 100 U/mL PS. MCF 10A cell lines were maintained in Mammary MEGM kit (Lonza/Clonetics) supplemented with 100 ng/mL cholera toxin (Sigma, Shanghai, China). Cell lines were grown at 37 °C in a humidified atmosphere of 5% CO_2_. 

#### 4.10.2. Cell Viability Assay

The growth-inhibitory effects against HeLa, Hep G2, MCF-7, FaDu, and A549 cancer cells and normal cell lines MCF 10A were determined by MTT assay [21]. All of the synthesized compounds and the positive control drug doxorubicin were dissolved in 0.1% DMSO. Cells were seeded in 96 well plates at a density of 1 × 10^4^ cells per well and incubated for 24 h at 37 °C in a humidified atmosphere of 5% CO_2_. The cells were then incubated with various concentrations of drugs for 48 h in a 5% CO_2_ incubator at 37 °C. After treatment with 1 mg/mL 3-(4,5-dimethylthiazol-2-yl)-2,5-diphenyltetrazolium bromide (MTT) solution for 4 h, the formazan crystals were dissolved in 100 µL DMSO in each well. Plates were read on a microplate reader (Thermo Varioskan Flash 3001) at 490 nm. The experiment was performed in triplicate. The inhibitory concentration at 50% (IC_50_) was calculated based on concentration-inhibition relationships via regression using SPSS software (Version 17.0).

#### 4.10.3. Colony Formation Assay 

FaDu cells were seeded in a 6-well plate with 500 cells per well and incubated for 24 h. Then, the cells were treated with compound **8e** at various final concentrations of 0, 0.75, 1.50, 3 nM. Following treatment for 10 days, the cells were washed with PBS twice and fixed with 4% paraformaldehyde for 10 min at room temperature. Then, the cell colonies were visualized by staining with 0.1% crystal violet for 10 min. The image was photographed with Handy camera, and the numbers of cell colonies were analyzed using the open Image J software (Developed by National Institutes of Health). A group of >50 cells was defined as one colony. Triplicate wells were set up for each concentration.

#### 4.10.4. Live/Dead Staining

The Calcein AM/propidium iodide (PI) double staining was performed following the manufacturer’s instructions (Dojindo Laboratory, Kumamoto, Japan). Briefly, FaDu cells were seeded in 6-well plates overnight. Then, the **8e** solutions at final concentrations of 2.5 nM, 5 nM, and 10 nM were added. After incubation for 24 h, the cells were collected, washed with PBS, and stained by a mixture of 2 µM Calcein-AM (live cells, green) and 4.5 µM PI (dead cells, red) solution in the dark for 30 min at 37 °C (excitation 490 nm). Then, the cells were rinsed with PBS twice and re-suspended in the cell medium. Confocal fluorescence images were acquired using a laser scanning confocal microscope (LSCM, Carl-Zeiss LSM 710). 

#### 4.10.5. Hoechst 33,342 Staining 

For Hoechst staining, FaDu cells were seeded onto a glass-bottom of cell culture dish at a density of 1 × 10^5^ cells. After incubation for 24 h, the media containing 20 nM of the compound **8e** was used to replace the culture medium. Then, the cells were stained with Hoechst 33,342 solution (10 µg/mL in the culture medium, Beyotime Institute of Biotechnology, Shanghai, China) at 37 °C in the dark for 20 min after treatment with **8e**. Then, the cells were washed with the serum-free medium to remove excess dye. LSCM (Carl-Zeiss LSM 710) was used to capture the images of nuclear morphological changes to find apoptotic cells.

#### 4.10.6. Flow Cytometric Analysis of Apoptosis by Annexin V-FITC/PI Staining

Apoptosis in FaDu cells was evaluated by an annexin V-FITC/PI apoptosis detection kit (Beyotime Institute of Biotechnology, Shanghai, China). Briefly, FaDu cells were treated with various concentrations of compound **8e** (2, 5, 10 nM) or DMSO as vehicle control for 24 h at 37 °C; then, the cells were washed twice with cold 1× PBS and centrifuged at 1000 rpm for 5 min. The harvested cells were resuspended gently in 400 µL binding buffer, containing 5 µL annexin V-FITC and 10 µL PI. After incubating for 15 min in the dark at room temperature, the cells were analyzed with flow cytometry (CyFlow^®^ Cube 6, Sysmex). Data were shown as pseudo color graphs and analyzed using FlowJo Software (Tree Star Inc, Ashland, OR). 

#### 4.10.7. Mitochondrial Membrane Potential (ΔΨm) Analysis

The mitochondrial membrane potential (*ΔΨ*m) was detected by a mitochondrial membrane potential assay kit with JC-1 (Beyotime Institute of Biotechnology, Shanghai, China). Briefly, after treatment with various concentrations of compound **8e** (2, 5, 10 nM) for 24 h, FaDu cells were harvested and stained with 500 µL 1× JC-1 dye solution at 37 °C for 20 min in the dark. Then, the treated cells were washed twice and resuspended by 1× JC-1 staining buffer. The fluorescence of approximately 1 × 10^4^ cells was analyzed using flow cytometry (CyFlow^®^ Cube 6, Sysmex).

#### 4.10.8. Cell Cycle Analysis

The cell cycle analysis was carried out by a cell cycle and apoptosis analysis kit with a propidium iodide (PI) staining method (Beyotime Institute of Biotechnology, Shanghai, China). Briefly, FaDu cells were incubated with the above-mentioned doses of compound **8e** for 24 h; all samples were collected, washed once with cold 1× PBS, and fixed by 70% ice-cold ethanol at 4 °C for 12 h. Then, the fixed cells were washed once with cold 1× PBS and stained with 500 µL 1× PI dye solution (containing 10 µL RNase A) at 37 °C for 30 min in the dark. The red fluorescence was detected using a flow cytometer (CyFlow^®^ Cube 6, Sysmex). The data were analyzed using FlowJo Software (Tree Star Inc, Ashland, OR, USA).

#### 4.10.9. In Silico ADMET Prediction

A computational study of all synthesized compounds was carried out for the prediction of ADMET (absorption, distribution, metabolism, excretion, and toxicity) properties. In this module, thirteen mathematical models, such as HBD (hydrogen bond donor), HBA (hydrogen bond acceptor), RBN (number of rotatable bonds), logP (log of the octanol/water partition coefficient), log S (log of the aqueous solubility), and PSA (polar surface area) were predicted via Discovery Studio 2.0 Software (Studio 2.5, Accelrys, Co. Ltd., San Diego, CA, USA) and HIA (human intestinal absorption), PPB (plasma protein binding), CYP3D4 (CYP3D4 inhibition), T_1/2_ (half lifetime), HT (human hepatotoxicity), and LD_50_ (median lethal dose) were calculated using web-based applications and then analyzed to predict the drug likeliness profile (Xiangya School of Pharmaceutical Sciences and Central South University, http://admet.scbdd.com/home/index/) [28]. We also screened our novel derivatives through the prediction model by Egan et al. [29,30]. Two molecular descriptors, AlogP98 and PSA, were computed and plotted in a 2D plane to comprehensively evaluate the absorption and blood-brain penetration of the compounds.

## Figures and Tables

**Table 1:** Inhibitory effects of synthetic compounds on the growth of different types of human cancer cells and normal mammary epithelial cells.

Comp.	Antiproliferative Activity IC_50_ ± SEM (µM)						
	HeLa[a]	Hep G2[b]	MCF-7[c]	FaDu[d]	A549[e]	MCF 10A[f]	SI[g]
**6a**	>100	>100	>100	>100	>100	90.29 ± 3.41	NC^h^
**6b**	>100	>100	>100	>100	>100	>100	NC^h^
**6c**	>100	>100	>100	>100	>100	>100	NC^h^
**6d**	>100	>100	>100	>100	>100	>100	NC^h^
**6e**	62.67 ± 4.19	50.77 ± 1.35	66.36 ± 0.05	49.50 ± 1.61	>100	85.44 ± 2.28	1.73
**6f**	10.16 ± 0.55	10.72 ± 0.22	20.83 ± 0.14	10.94 ± 0.12	20.05 ± 0.41	12.97 ± 0.55	1.19
**6g**	7.89 ± 0.86	9.62 ± 0.11	9.83 ± 0.17	6.80 ± 0.05	9.23 ± 0.16	6.57 ± 0.30	0.97
**6h**	>100	>100	>100	>100	>100	>100	NC^h^
**6i**	>100	>100	>100	>100	>100	>100	NC^h^
**8a**	>100	>100	>100	>100	>100	>100	NC^h^
**8b**	>100	>100	>100	>100	>100	>100	NC^h^
**8c**	>100	>100	>100	80.41 ± 1.36	>100	46.59 ± 0.65	NC^h^
**8d**	3.31 ± 0.14	>20	0.480 ± 0.003	0.11 ± 0.03	6.35 ± 0.05	2.69 ± 0.46	24.45
**8e**	1.42 ± 0.71	>20	0.037 ± 0.001	0.00136 ± 0.00035	1.05 ± 0.05	0.047 ± 0.008	34.56
**8f**	4.96 ± 1.62	>20	0.158 ± 0.009	0.174 ± 0.010	2.46 ± 0.05	0.051 ± 0.021	0.29
**9e**	>100	>100	>100	0.054 ± 0.002	11.27 ± 0.81	>100	>1851.85
**10e**	>100	>100	>100	0.25 ± 0.02	2.94 ± 0.25	38.60 ± 2.48	154.40
**11e**	>100	>100	>100	0.50 ± 0.06	64.30 ± 3.46	80.54 ± 7.60	161.08
**12e**	>100	>100	>100	1.33 ± 0.07	8.77 ± 0.47	>100	>75.19
**13e**	>100	>100	>100	48.31 ± 3.17	>100	>100	>2.07
**14e**	>100	97.75 ± 1.55	>100	6.33 ± 0.65	44.37 ± 0.29	19.82 ± 1.41	3.13
**15e**	37.74 ± 4.32	26.34 ± 5.36	21.48 ± 1.47	8.85 ± 0.45	23.08 ± 0.12	54.65 ± 5.92	6.18
**16e**	>100	>100	>100	88.11 ± 1.37	>100	>100	NC^h^
**17e**	>20	>20	>20	0.236 ± 0.005	>20	>20	>84.75
**18e**	>20	>20	>20	0.697 ± 0.021	>20	>20	>28.69
**19e**	>20	>20	>20	8.74 ± 0.44	>20	>20	>2.29
**20e**	>20	>20	>20	>20	>20	>20	NC^h^
**21e**	>20	>20	>20	0.665 ± 0.028	>20	>20	>30.08
**22e**	>20	>20	>20	0.020 ± 0.002	>20	>20	>1000
**23e**	>20	>20	>20	0.638 ± 0.089	>20	>20	>31.35
**24e**	>20	>20	>20	5.296 ± 0.366	>20	>20	>3.78
**25e**	>20	>20	>20	0.056 ± 0.016	>20	>20	>357.14
**26e**	>20	>20	>20	>20	>20	>20	NC^h^
**27e**	>20	>20	>20	4.621 ± 0.539	>20	>20	>4.33
**28e**	>20	>20	>20	>20	>20	>20	NC^h^
**29e**	>20	>20	>20	5.067 ± 0.461	>20	>20	>3.95
**30e**	>20	>20	>20	>20	>20	>20	NC^h^
**31e**	>20	>20	>20	5.594 ± 0.628	>20	>20	>3.58
**32e**	>20	>20	>20	>20	>20	>20	NC^h^
**33e**	>20	>20	>20	10.393 ± 0.949	>20	>20	>1.92
**34e**	>20	>20	>20	5.853 ± 0.408	>20	>20	>3.42
**35e**	>20	>20	>20	>20	>20	>20	NC^h^
**9d**	>20	>20	>20	1.054 ± 0.091	>20	>20	>18.98
**9f**	>20	>20	>20	0.027 ± 0.002	>20	>20	>740.74
**9g**	>20	>20	>20	>20	>20	>20	NC^h^
**9h**	>20	>20	>20	>20	>20	>20	NC^h^
**9i**	>20	>20	>20	>20	>20	>20	NC^h^
**9j**	>20	>20	>20	>20	>20	>20	NC^h^
**9k**	>20	>20	>20	4.447 ± 0.208	>20	>20	>4.50
**9l**	>20	>20	>20	>20	>20	>20	NC^h^
**9m**	>20	>20	>20	>20	>20	>20	NC^h^
**TMP**	>100	>100	>100	>100	>100	>100	NC^h^
**DOX**	11.39 ± 0.48	28.04 ± 1.09	6 ± 0.07	1.27 ± 0.13	>100	0.55 ± 0.03	0.43

[a] HeLa (cervical cancer cell line); [b] Hep G2 (hepatocarcinoma cell line); [c] MCF-7 (breast cancer cell line); [d] FaDu (head and neck squamous carcinoma cell line); [e] A549 (epithelial cancer cell line); [f] MCF 10A (normal mammary epithelial cell); [g] SI: selectivity index. It was calculated as: SI = IC_50 (MCF 10A)_/IC_50 (FaDu)_; [h] NC: not calculated.

**Table 2:** The ADMET properties of all synthetic compounds.

Comp.	HBD^a^	HBA^b^	RBN^c^	logP^d^	logS^e^	PSA^f^	HIA^g^	PPB^h^	BBB^i^	CYP3A4^j^	T_1/2_ (h)^k^	HT^l^	LD_50_(mg/kg)^m^
**6a**	0	10	11	1.84	−2.23	96.79	0.81	0.68	0.94	0	2.02	0	459.04
**6b**	0	10	12	1.90	−1.93	96.79	0.80	0.67	0.91	0	2.13	0	429.48
**6c**	0	10	13	2.48	−2.05	96.79	0.80	0.68	0.91	0	2.14	0	462.17
**6d**	0	10	15	3.39	−2.02	96.79	0.80	0.65	0.91	0	2.12	0	447.58
**6e**	0	10	17	4.31	−1.90	96.79	0.80	0.65	0.91	0	2.14	0	425.74
**6f**	0	10	19	5.22	−1.67	96.79	0.80	0.66	0.91	0	2.18	0	460.97
**6g**	0	10	21	6.13	−1.35	96.79	0.80	0.67	0.91	0	2.23	0	508.77
**6h**	0	6	4	0.97	−2.49	51.75	0.79	0.67	0.98	0	2.00	0	664.69
**6i**	1	6	6	0.47	−1.52	69.21	0.78	0.70	0.75	0	1.77	0	1011.08
**8a**	2	6	6	−0.09	−1.53	105.27	0.69	0.78	0.98	0	1.81	1	1027.40
**8b**	2	6	7	0.49	−1.87	105.27	0.72	0.79	0.98	0	1.88	1	988.28
**8c**	2	6	9	1.40	−2.30	105.27	0.72	0.80	0.98	0	1.89	1	1033.87
**8d**	2	6	11	2.31	−2.67	105.27	0.72	0.83	0.98	0	1.92	1	1099.10
**8e**	2	6	13	3.22	−2.97	105.27	0.72	0.85	0.98	0	2.03	1	1278.93
**8f**	2	6	15	4.14	−3.21	105.27	0.72	0.85	0.98	0	2.01	1	1318.54
**9e**	2	2	15	6.21	−4.45	60.22	0.69	0.89	0.99	0	1.93	1	961.82
**10e**	2	4	13	2.97	−2.60	82.74	0.70	0.91	0.99	0	1.85	1	1075.58
**11e**	2	2	13	5.27	−4.27	60.22	0.68	0.91	1.00	0	1.96	1	1653.45
**12e**	2	2	13	7.09	−6.11	60.22	0.69	0.92	0.99	0	2.12	1	1818.99
**13e**	2	4	13	2.97	−2.60	82.74	0.72	0.91	0.99	0	1.89	0	1070.63
**14e**	2	2	13	4.06	−3.66	85.33	0.41	0.90	1.00	0	1.78	0	1145.12
**15e**	2	4	13	3.83	−3.35	82.74	0.70	0.91	0.99	0	1.89	1	1128.86
**16e**	2	6	13	1.53	−1.68	105.27	0.68	0.85	0.99	0	1.82	1	1461.77
**17e**	2	4	13	5.65	−5.10	82.74	0.70	0.95	0.98	0	2.22	1	1217.90
**18e**	2	4	13	5.65	−5.10	82.74	0.72	0.96	0.95	0	1.99	1	1254.91
**19e**	4	4	13	4.31	−4.38	112.85	0.69	0.90	0.94	0	1.91	1	1167.64
**20e**	2	6	13	5.33	−4.57	112.68	0.43	0.93	0.98	0	2.29	0	973.08
**21e**	2	2	13	6.46	−6.02	85.33	0.47	0.94	0.99	0	2.00	1	966.72
**22e**	2	2	13	7.40	−6.99	60.22	0.69	0.91	0.99	0	2.07	1	904.88
**23e**	2	2	13	6.27	−5.55	70.92	0.75	0.95	0.99	0	2.27	1	995.92
**24e**	4	2	13	5.86	−5.15	90.33	0.72	0.95	0.96	0	2.01	1	1072.61
**25e**	2	2	15	7.54	−5.78	60.22	0.67	0.89	0.99	0	1.98	1	1322.11
**26e**	4	6	17	5.69	−3.28	119.71	0.45	0.87	0.85	0	1.93	1	1249.90
**27e**	2	6	19	6.14	−3.64	95.94	0.54	0.82	0.98	0	1.82	1	1217.58
**28e**	4	8	19	5.66	−3.13	137.57	0.46	0.77	0.94	0	2.02	1	1163.97
**29e**	2	8	21	6.11	−3.08	113.80	0.54	0.73	0.96	0	1.95	0	927.46
**30e**	4	4	13	3.78	−2.74	113.30	0.59	0.90	0.99	0	1.96	1	849.92
**31e**	4	4	13	3.78	−2.69	113.30	0.64	0.90	0.97	0	1.93	0	951.43
**32e**	4	4	13	3.78	−2.72	113.30	0.64	0.90	0.98	0	1.94	0	853.84
**33e**	2	6	17	5.21	−3.97	95.94	0.53	0.81	0.99	0	1.76	0	1184.47
**34e**	2	8	19	5.18	−3.62	113.80	0.54	0.76	0.98	0	1.63	0	1073.30
**35e**	2	8	19	5.18	−3.67	113.80	0.53	0.76	0.97	0	1.72	0	1061.01
**9d**	2	2	13	5.30	−4.11	60.22	0.69	0.91	0.99	0	1.92	1	1523.97
**9f**	2	2	17	7.12	−4.73	60.22	0.69	0.87	0.99	0	1.95	0	1003.19
**9g**	0	4	15	6.59	−5.34	52.46	0.55	0.88	0.98	0	1.96	1	6181.36
**9h**	0	4	17	7.50	−5.69	52.46	0.55	0.86	0.98	0	1.96	1	4624.51
**9i**	0	4	19	8.42	−5.96	52.46	0.55	0.85	0.98	0	1.96	0	3127.76
**9j**	2	4	13	2.57	−2.06	78.08	0.37	0.86	0.99	0	1.97	1	1574.67
**9k**	2	5	16	2.44	−1.41	87.01	0.37	0.85	0.99	0	1.90	0	1693.04
**9l**	0	6	15	3.86	−2.96	70.32	0.50	0.88	0.98	0	1.89	1	4511.05
**9m**	0	7	18	3.73	−2.14	79.25	0.50	0.86	0.98	0	1.83	0	4493.16
**DOX**	6	12	5	−0.04	−4.80	209.31	0.02	0.78	0.02	0	2.71	1	324.50
**TMP**	0	2	0	0.66	−1.51	22.52	0.93	0.49	0.99	0	1.77	0	1194.46

^a^ HBD (hydrogen bond donor), ^b^ HBA (hydrogen bond acceptor), ^c^ RBN (number of rotatable bonds), ^d^ logP (log of the octanol/water partition coefficient), ^e^ log S (log of the aqueous solubility), and ^f^ PSA (polar surface area) were predicted using Discovery Studio 2.0 software. ^g^ HIA (human intestinal absorption), ^h^ PPB (plasma protein binding), ^I^ CYP3D4 (CYP3D4 inhibition), ^j^ T1/2 (half lifetime), ^k^ HT (human hepatoxicity), and ^l^ LD50 (median lethal dose) were calculated via http://admet.scbdd.com/home/index/

## Supplementary Materials

The following are available online at https://www.mdpi.com/1420-3049/24/24/4505/s1, ^1^H-NMR, ^13^C-NMR and HR-MS spectra of compounds.
