# Supplementary material for: Novel Homo-Bivalent and Polyvalent Compounds Based on Ligustrazine and Heterocyclic Ring as Anticancer Agents
Source: Molecules. 2019 Dec 9;24(24):4505. doi: 10.3390/molecules24244505 (PMC6943434; doi:10.3390/molecules24244505)
Supplement: Supplementary file 1 [file molecules-24-04505-s001.pdf]

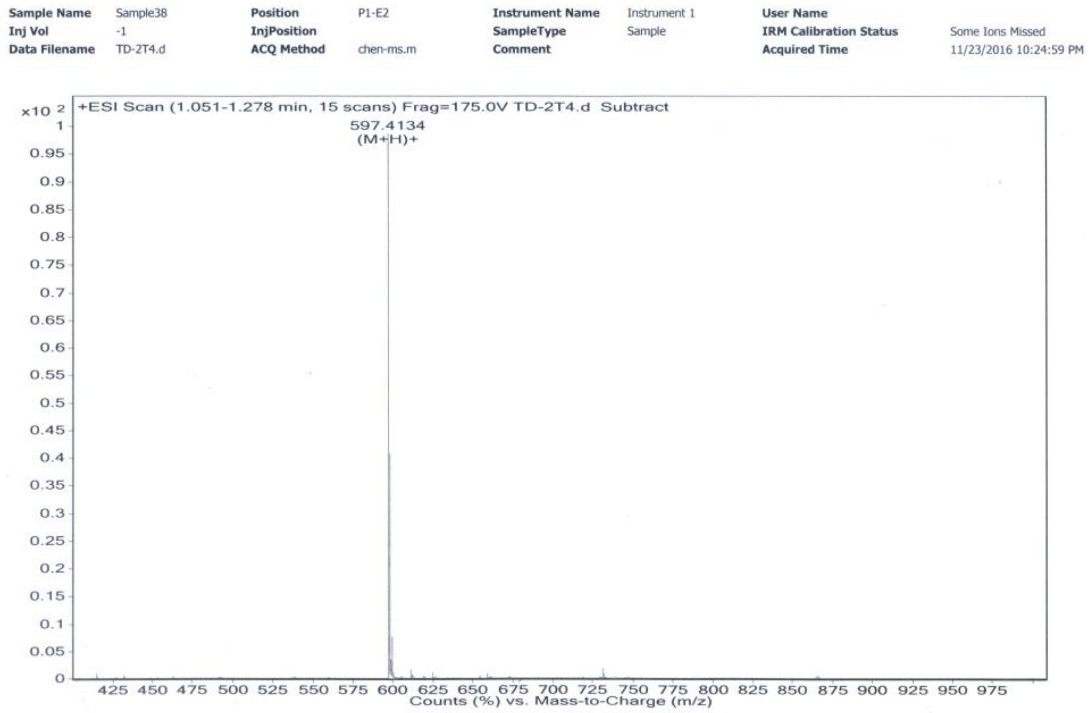

Figure S1. HRMS of compound **6a**

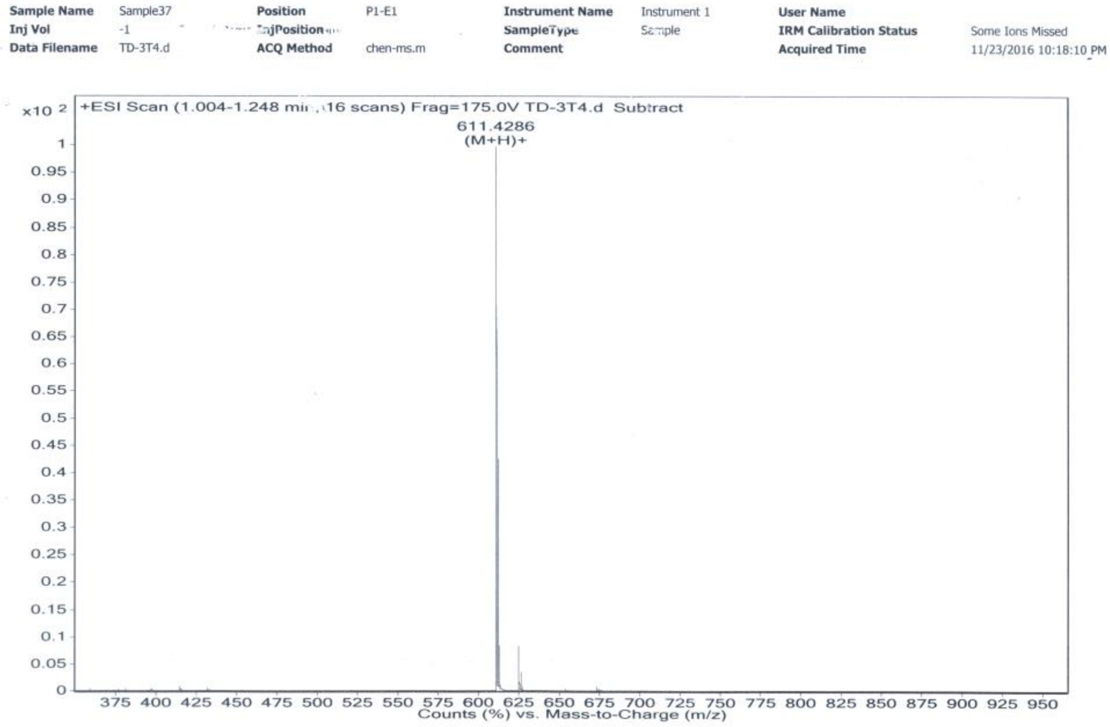

Figure S2. HRMS of compound **6b**

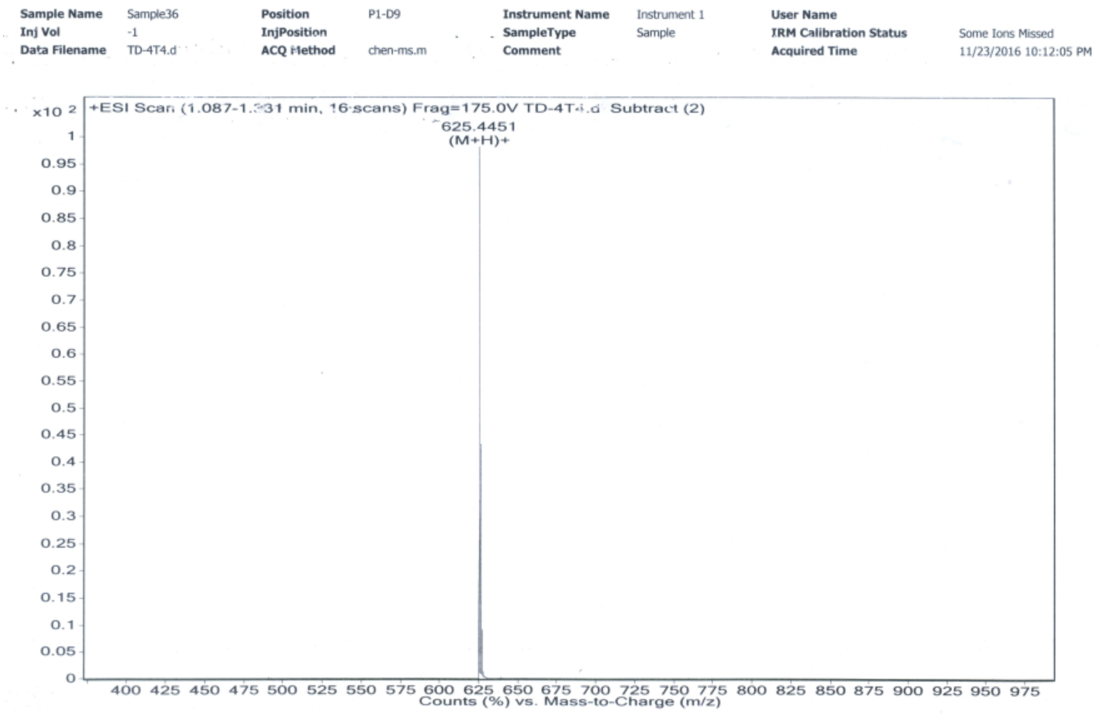

Figure S3. HRMS of compound **6c**

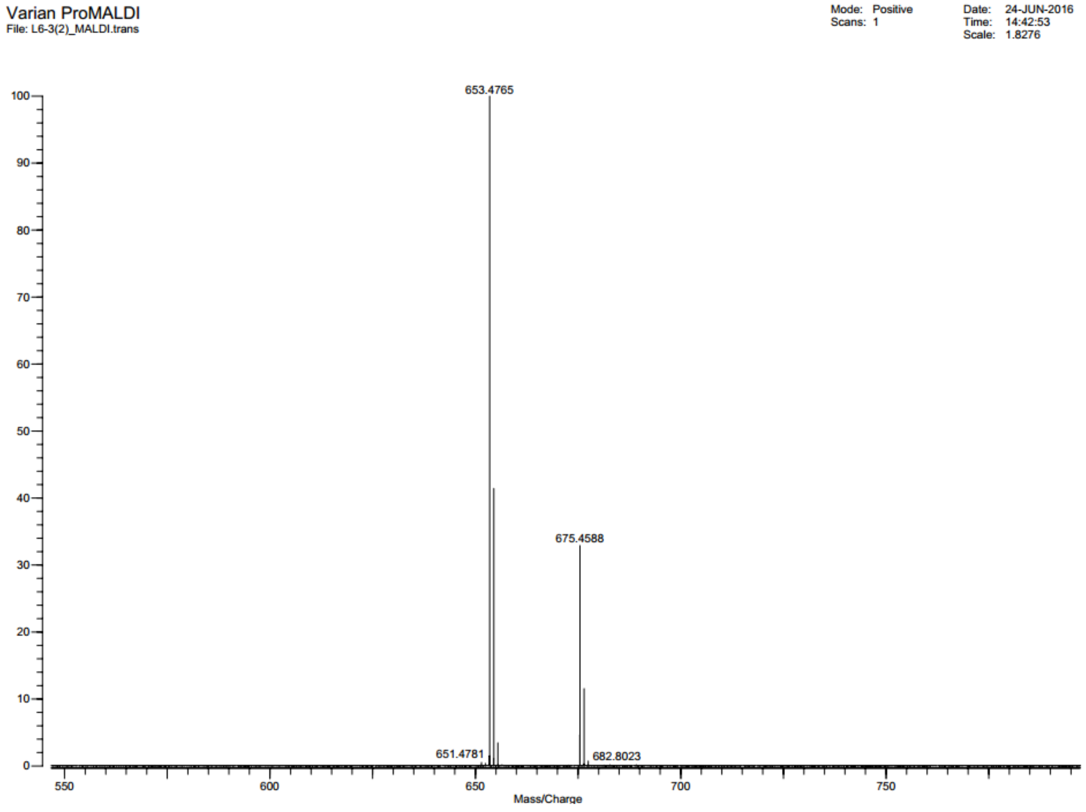

Figure S4. HRMS of compound **6d**

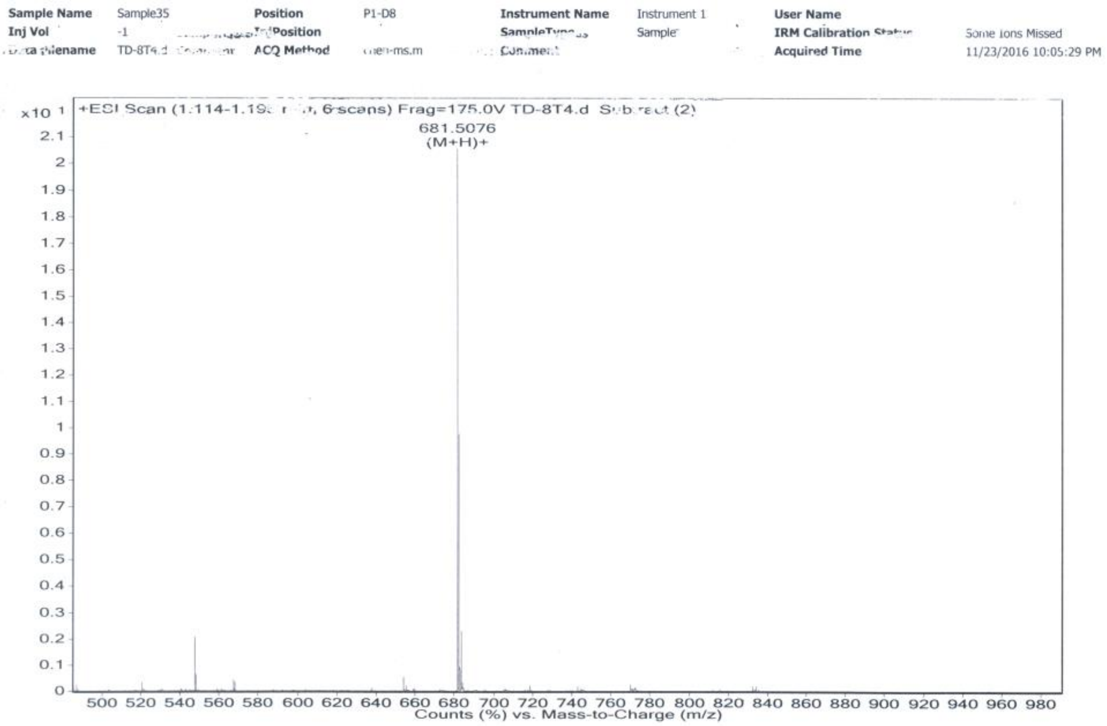

Figure S5. HRMS of compound **6e**

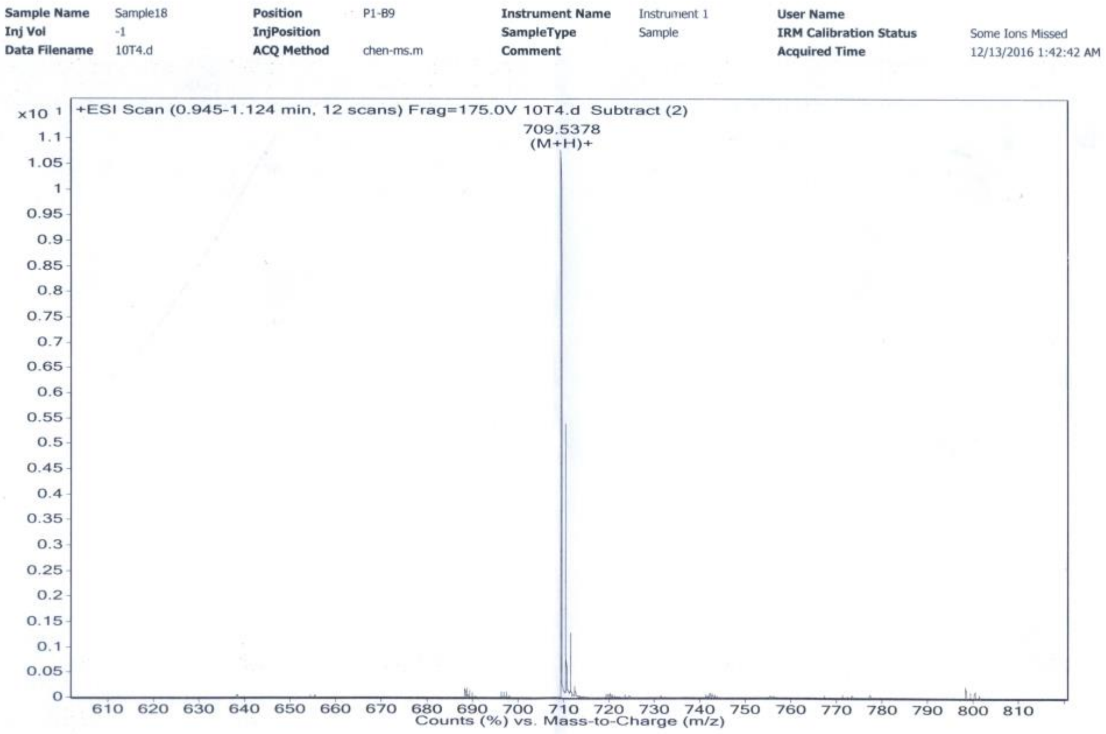

Figure S6. HRMS of compound **6f**

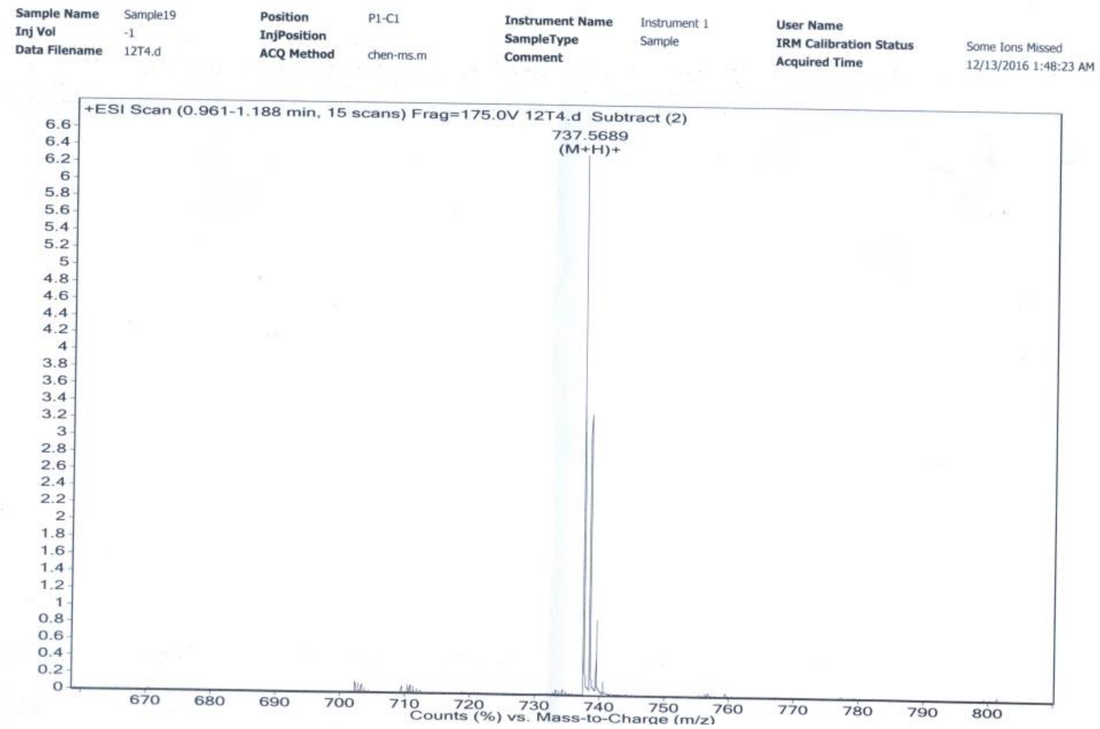

Figure S7. HRMS of compound **6g**

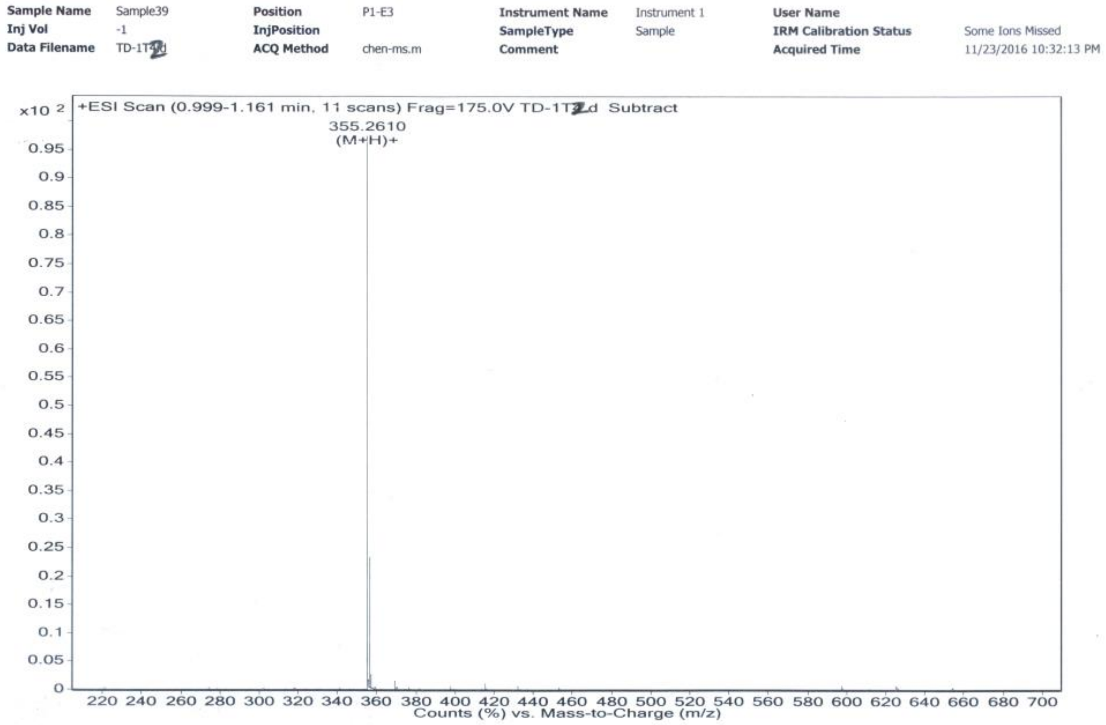

Figure S8. HRMS of compound **6h**

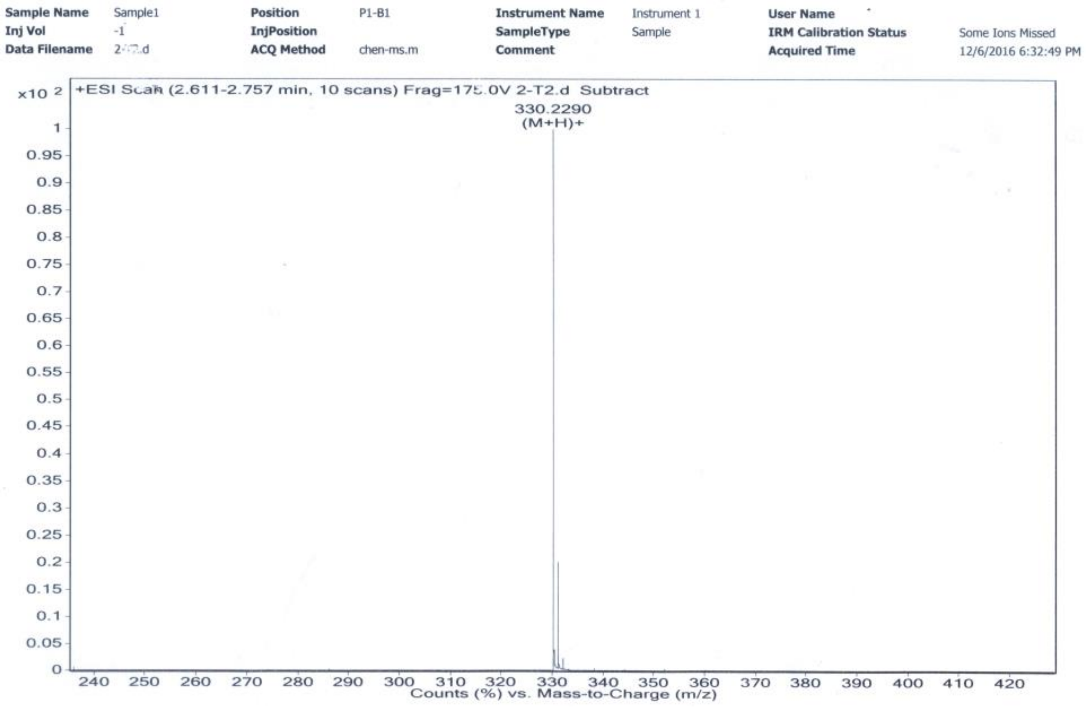

Figure S9. HRMS of compound **6i**

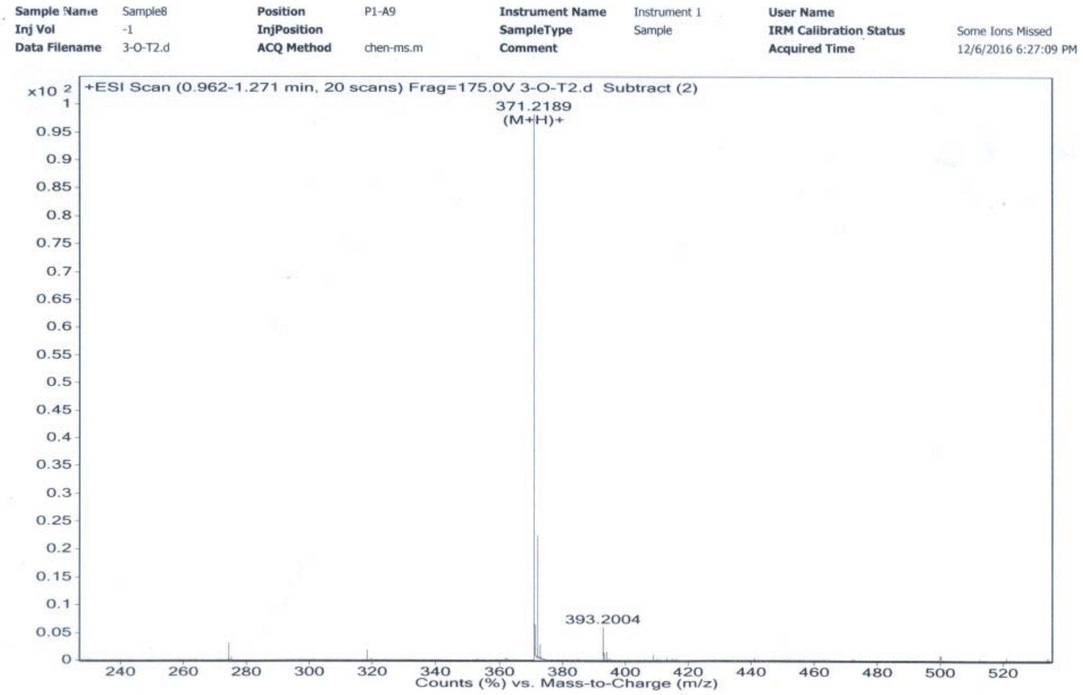

Figure S10. HRMS of compound **8a**

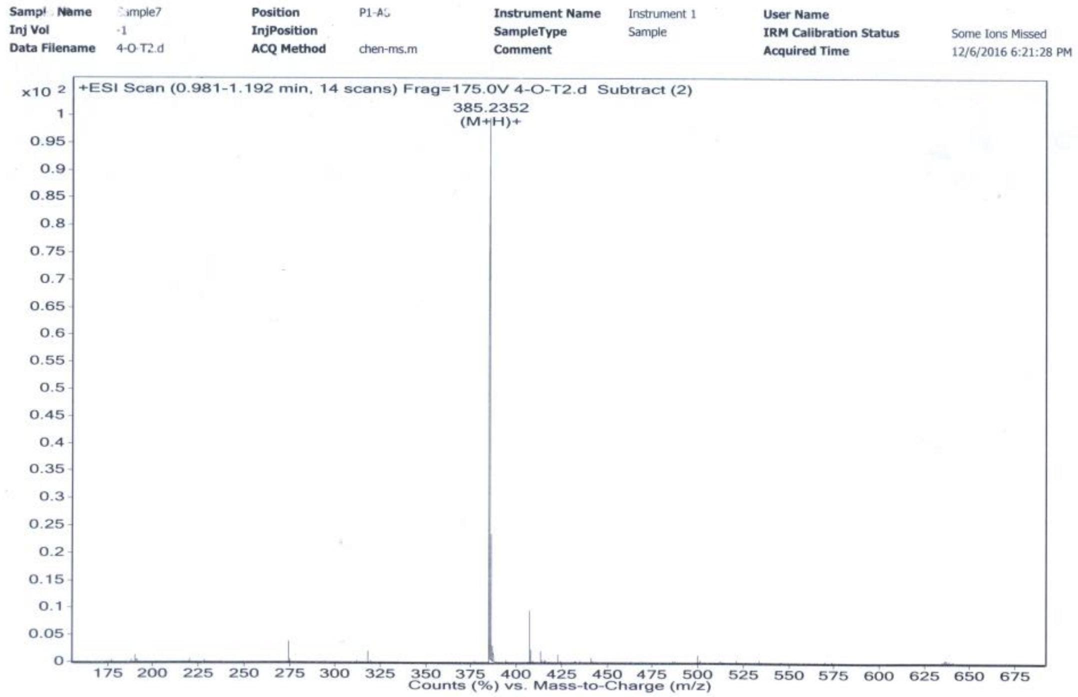

Figure S11. HRMS of compound **8b**

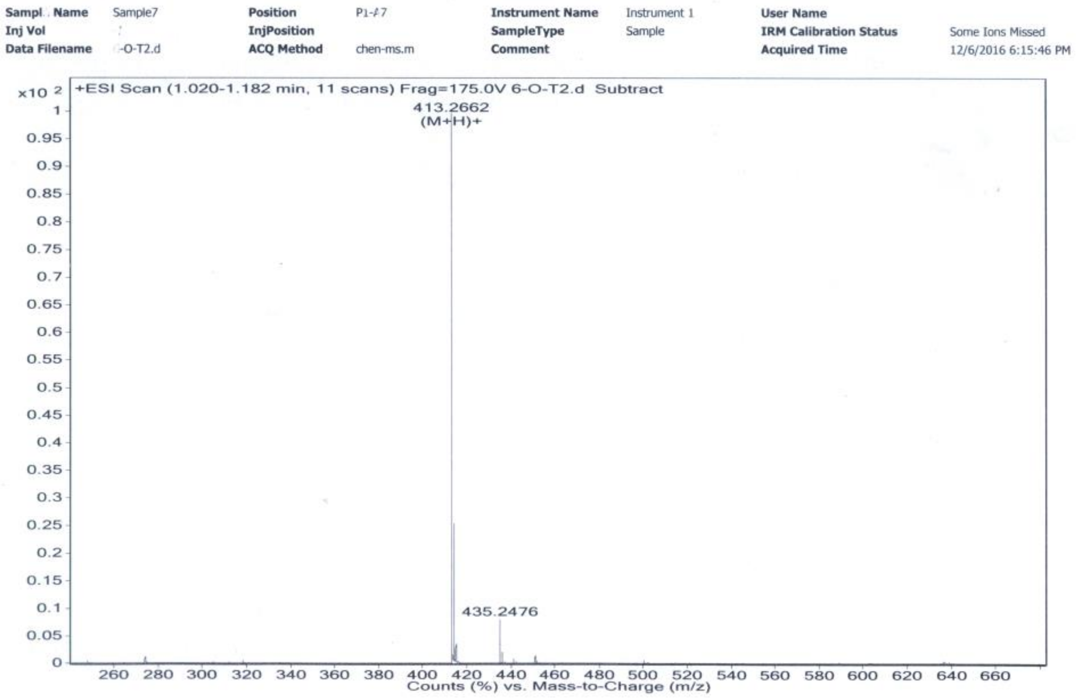

Figure S12. HRMS of compound **8c**

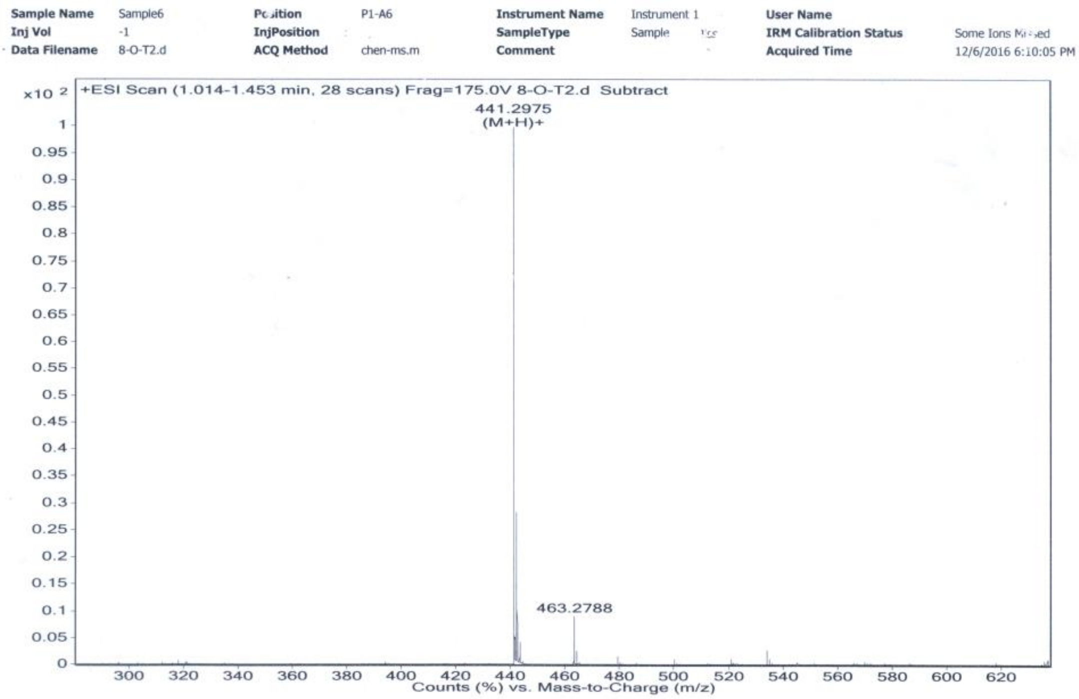

Figure S13. HRMS of compound **8d**

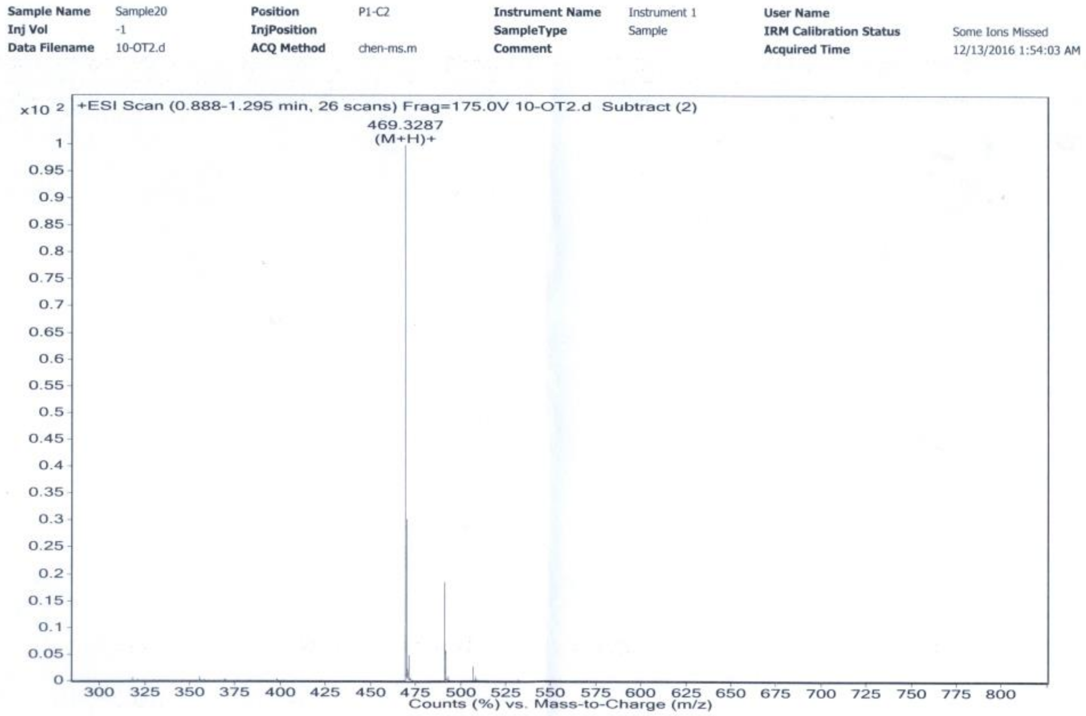

Figure S14. HRMS of compound **8e**

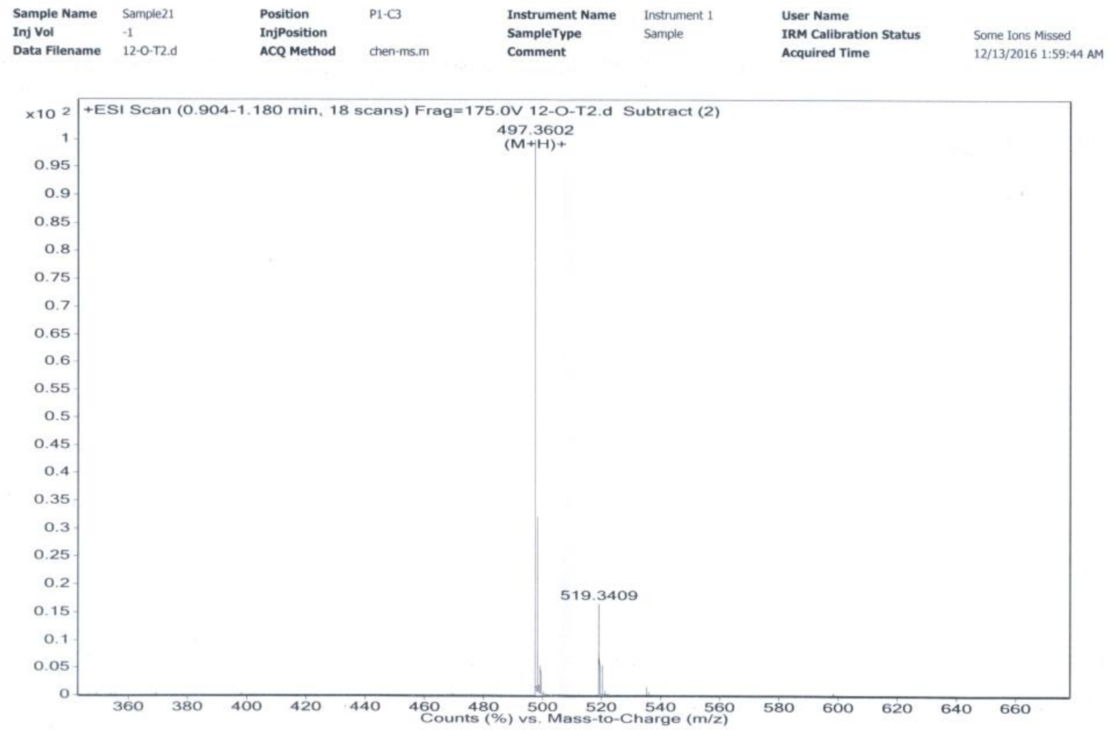

Figure S15. HRMS of compound **8f**

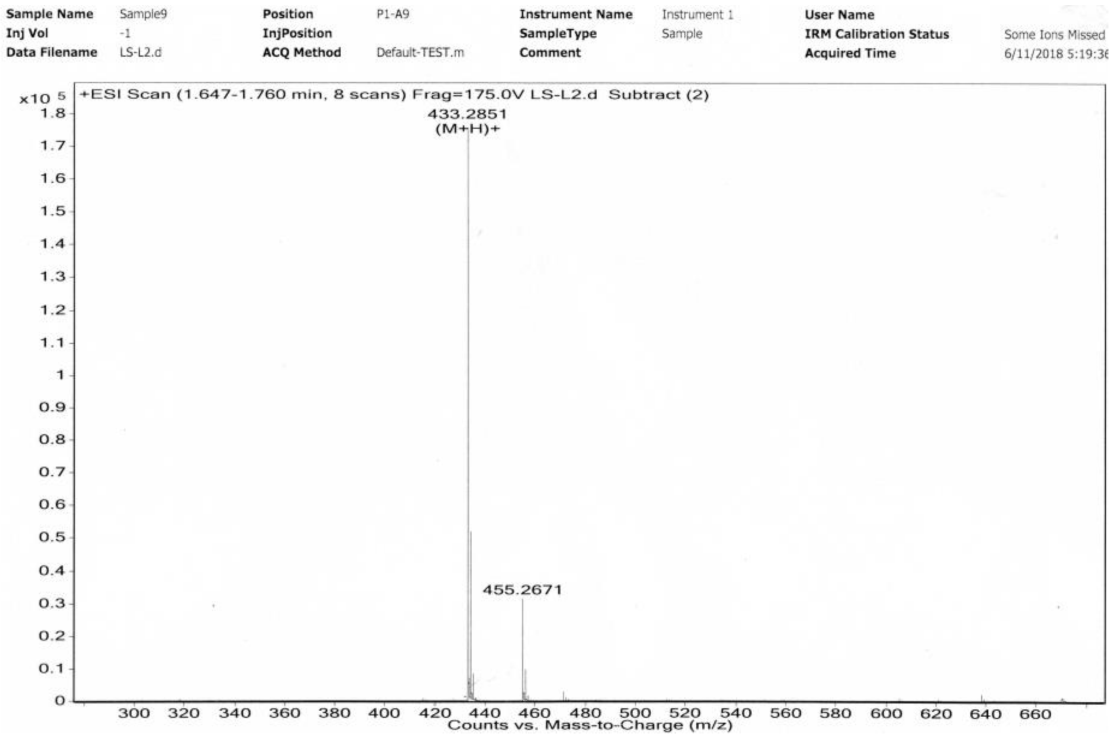

Figure S16. HRMS of compound **9e**

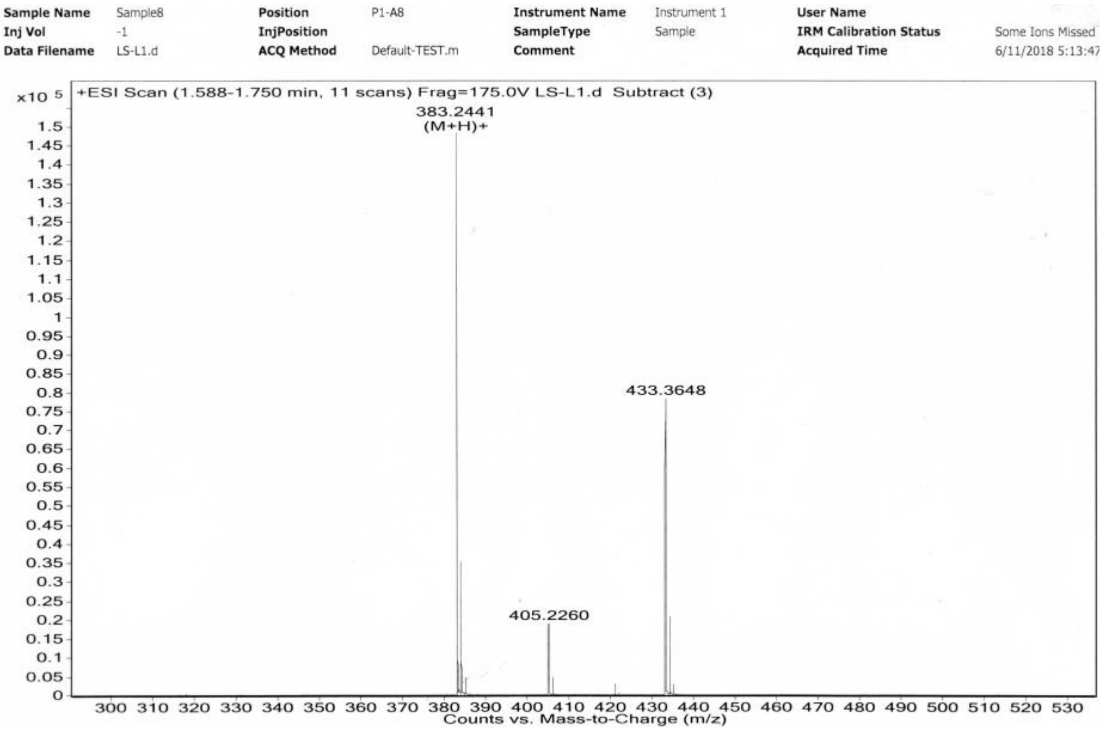

Figure S17. HRMS of compound **10e**

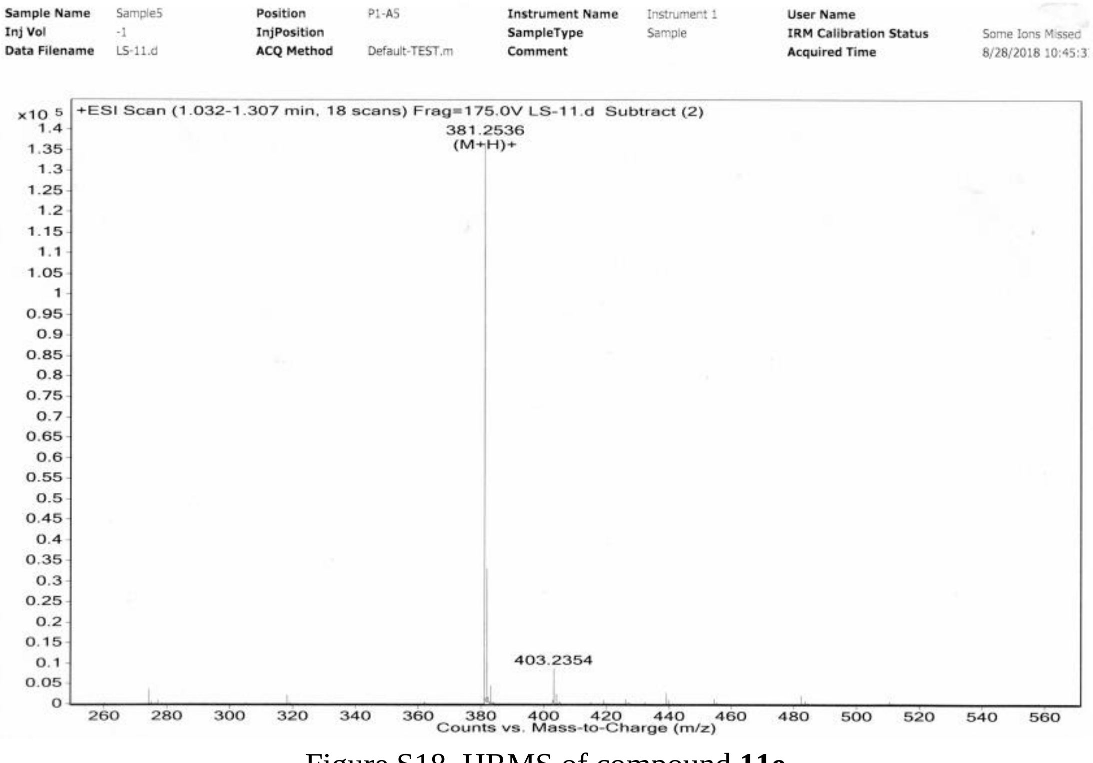

Figure S18. HRMS of compound **11e**

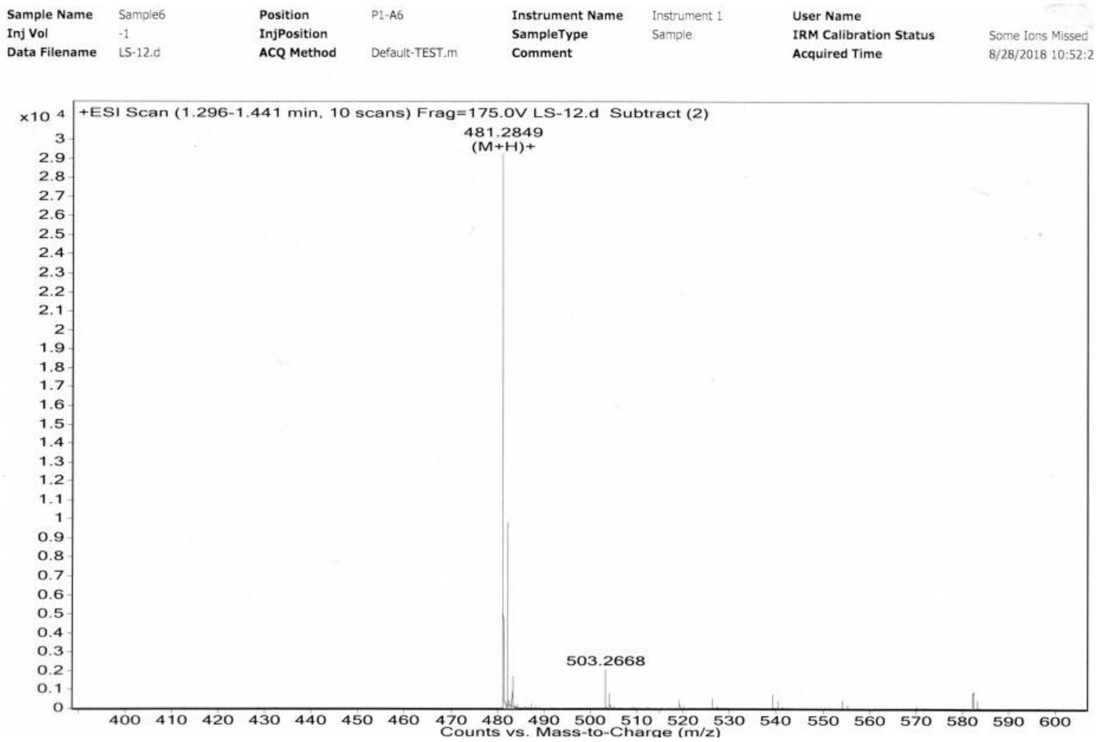

Figure S19. HRMS of compound **12e**

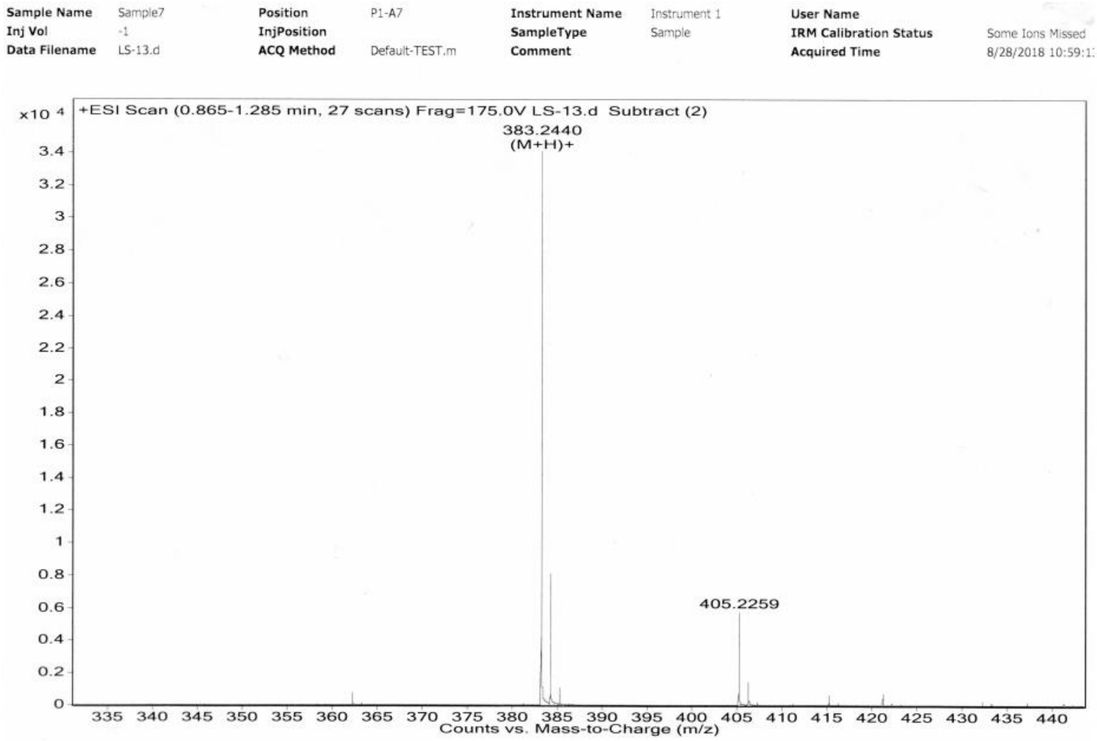

Figure S20. HRMS of compound **13e**

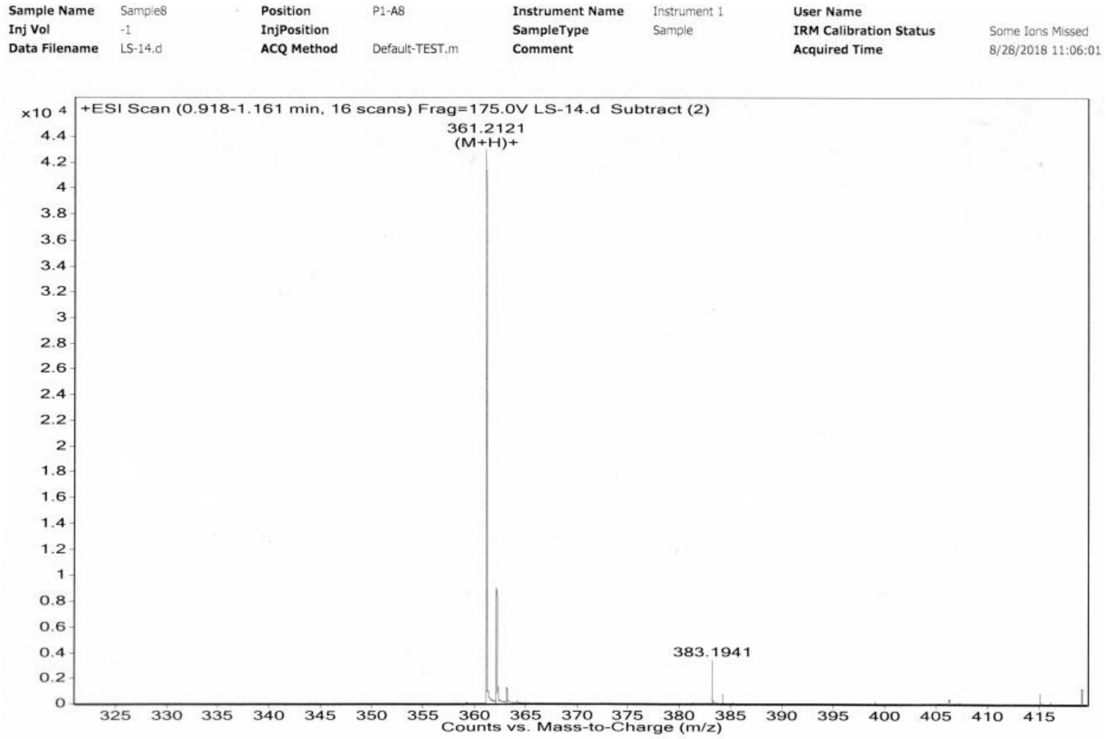

Figure S21. HRMS of compound **14e**

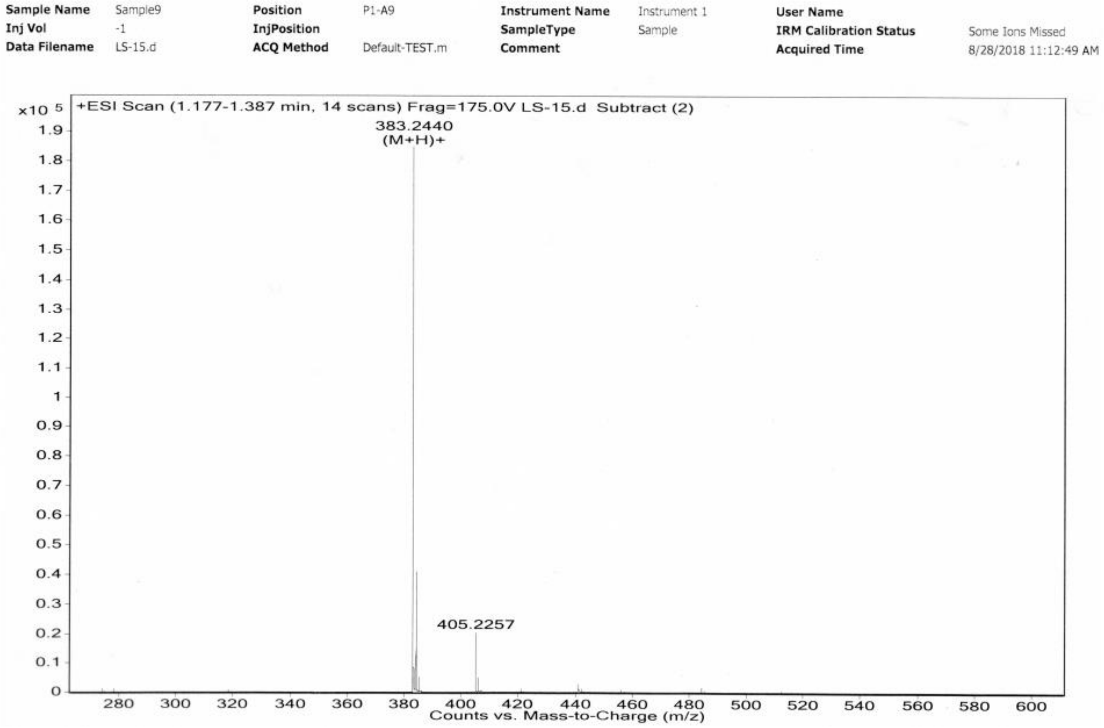

Figure S22. HRMS of compound **15e**

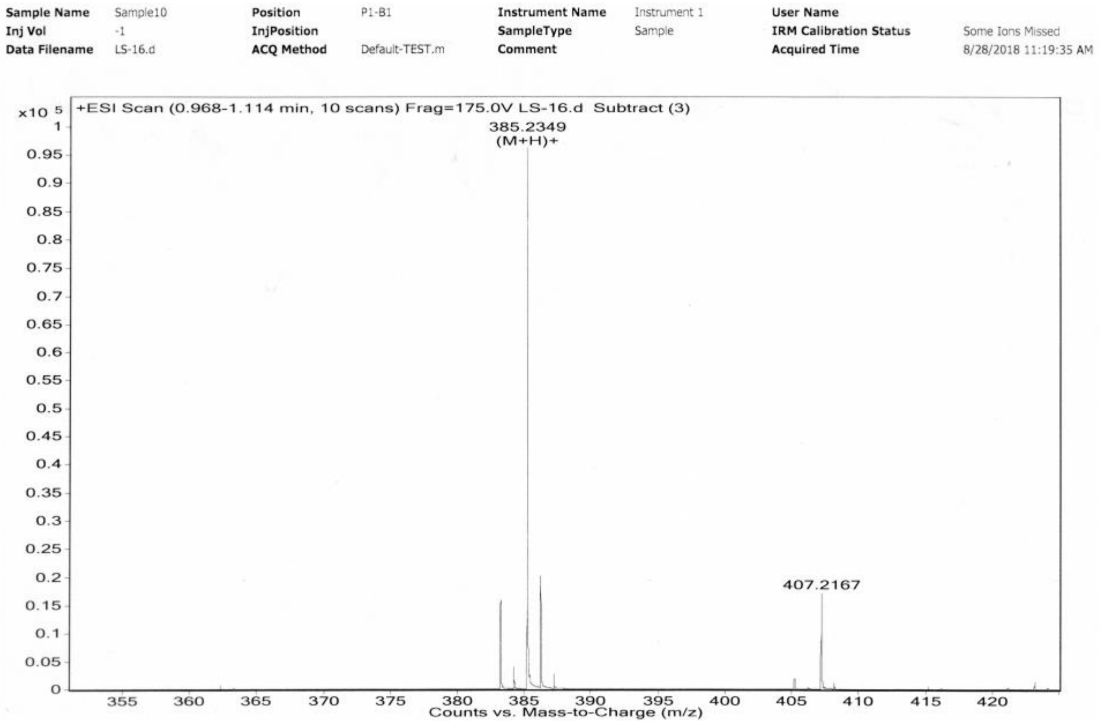

Figure S23. HRMS of compound **16e**

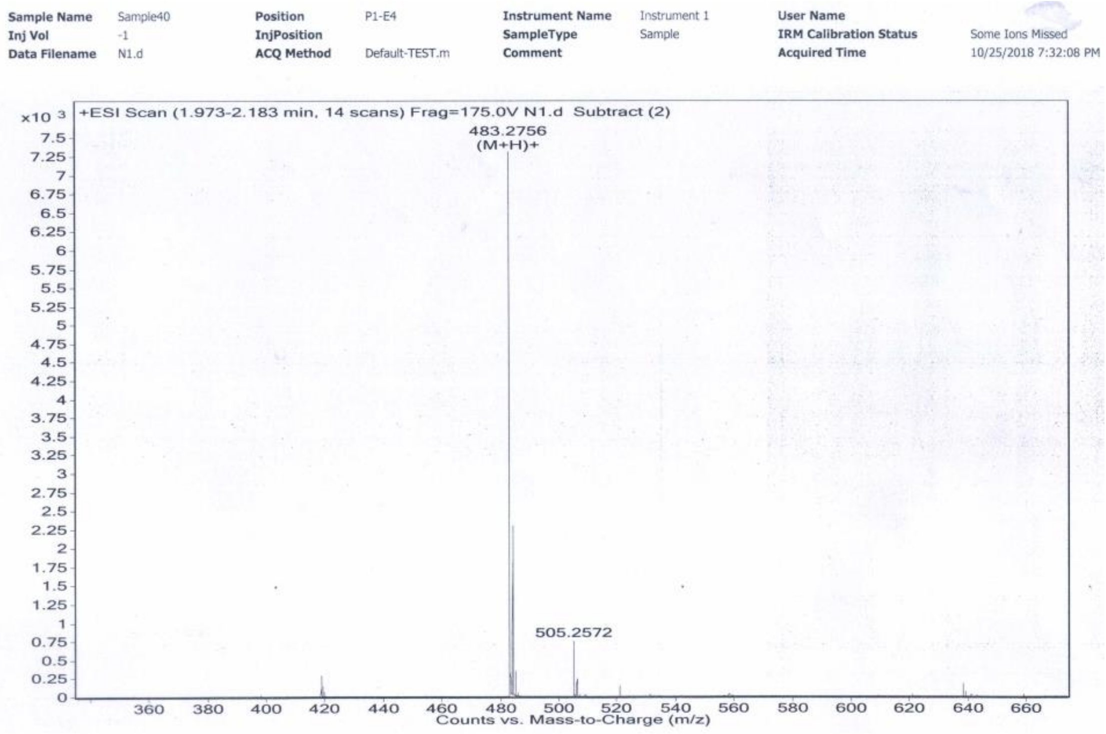

Figure S24. HRMS of compound **17e**

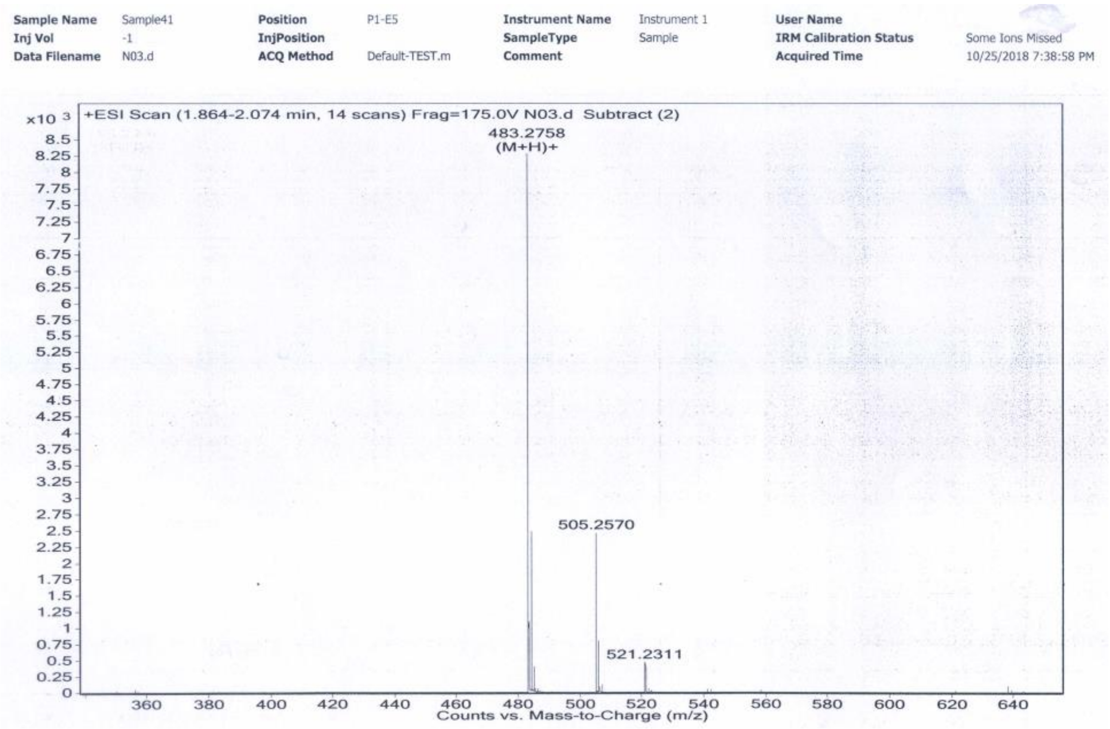

Figure S25. HRMS of compound **18e**

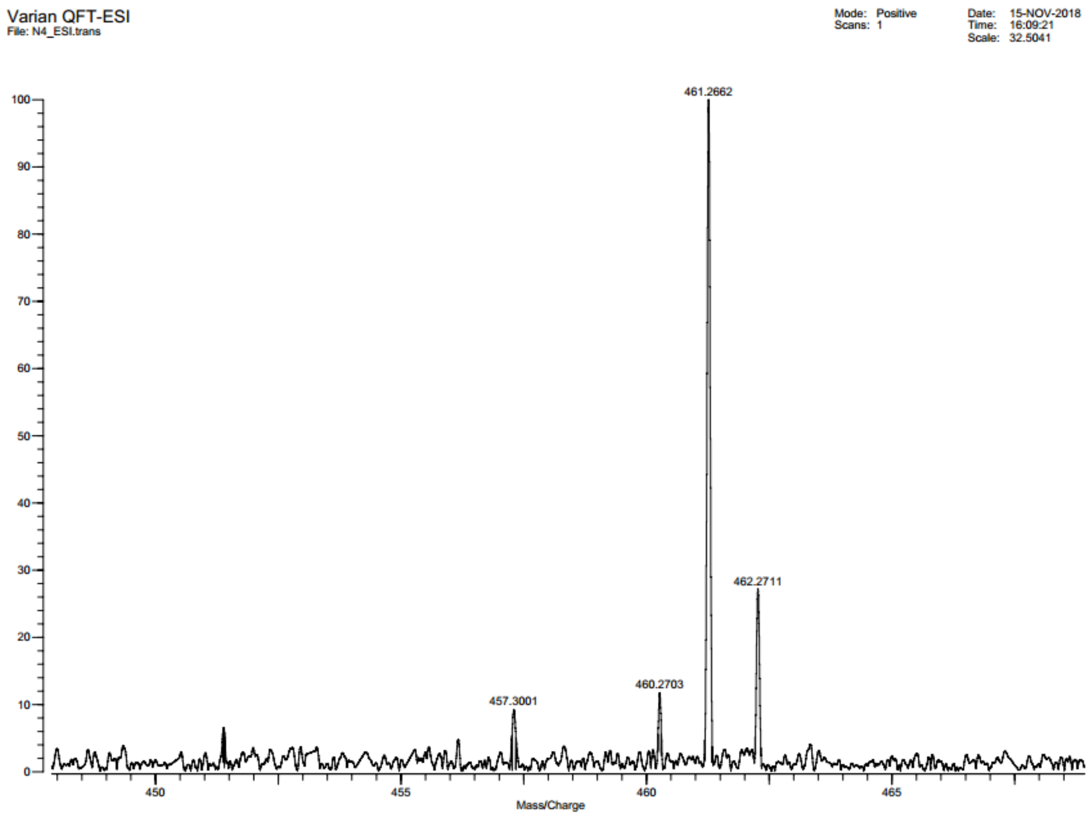

Figure S26. HRMS of compound **19e**

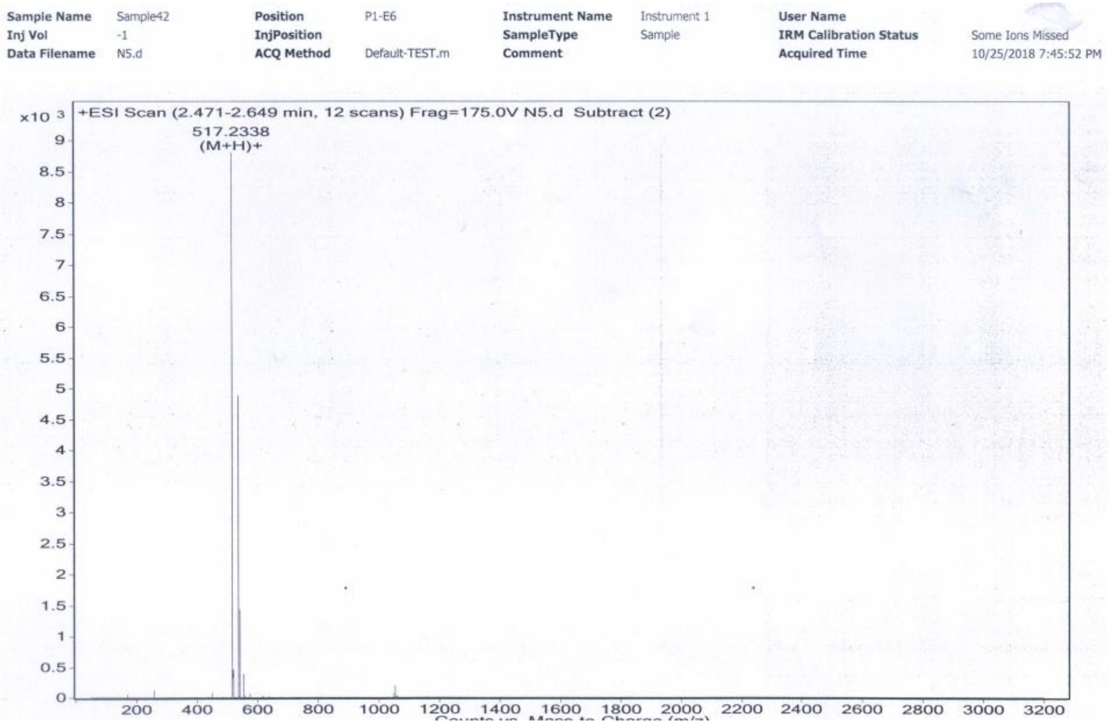

Figure S27. HRMS of compound **20e**

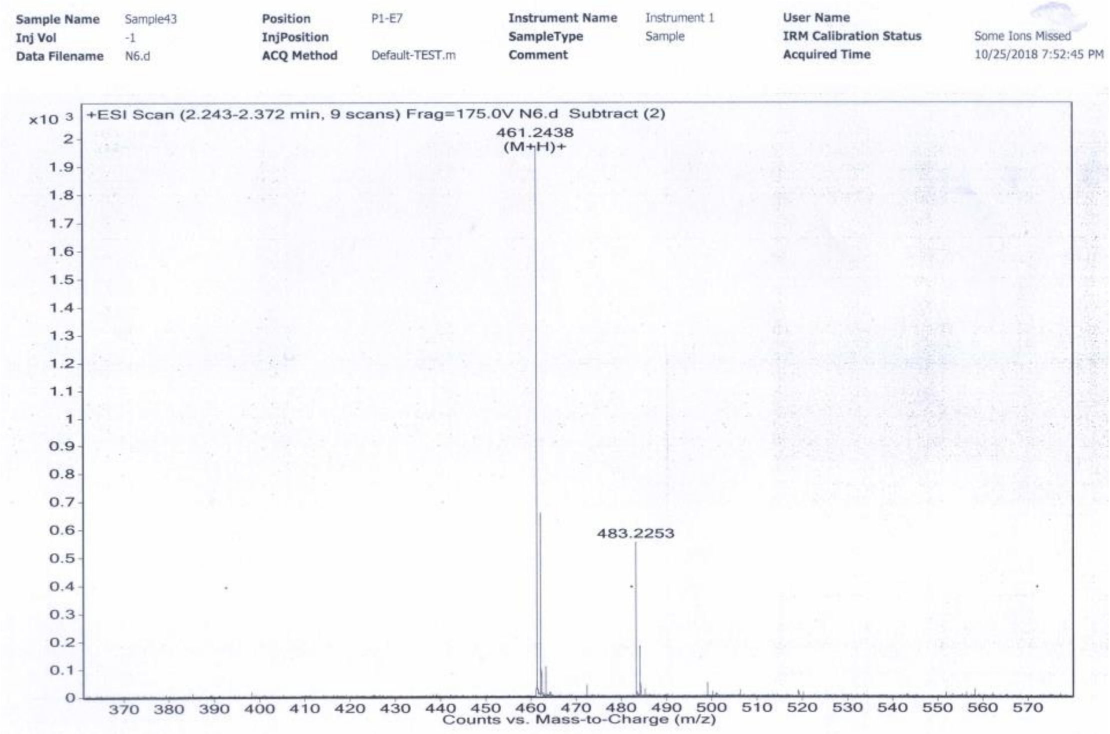

Figure S28. HRMS of compound **21e**

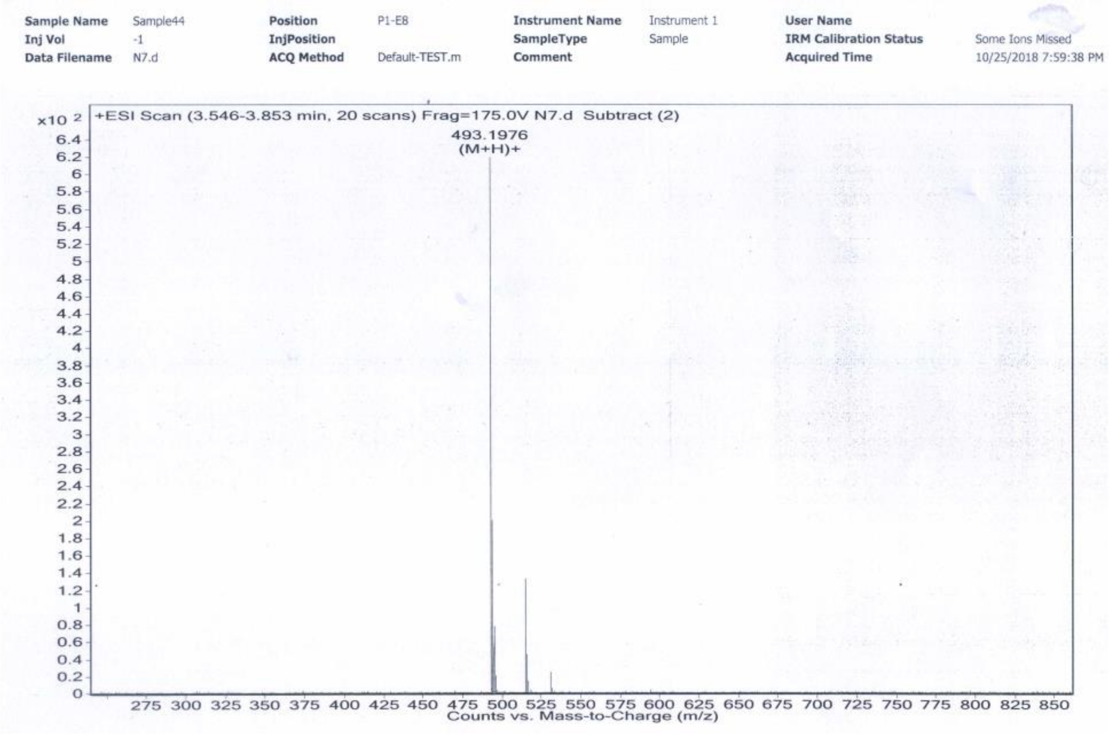

Figure S29. HRMS of compound **22e**

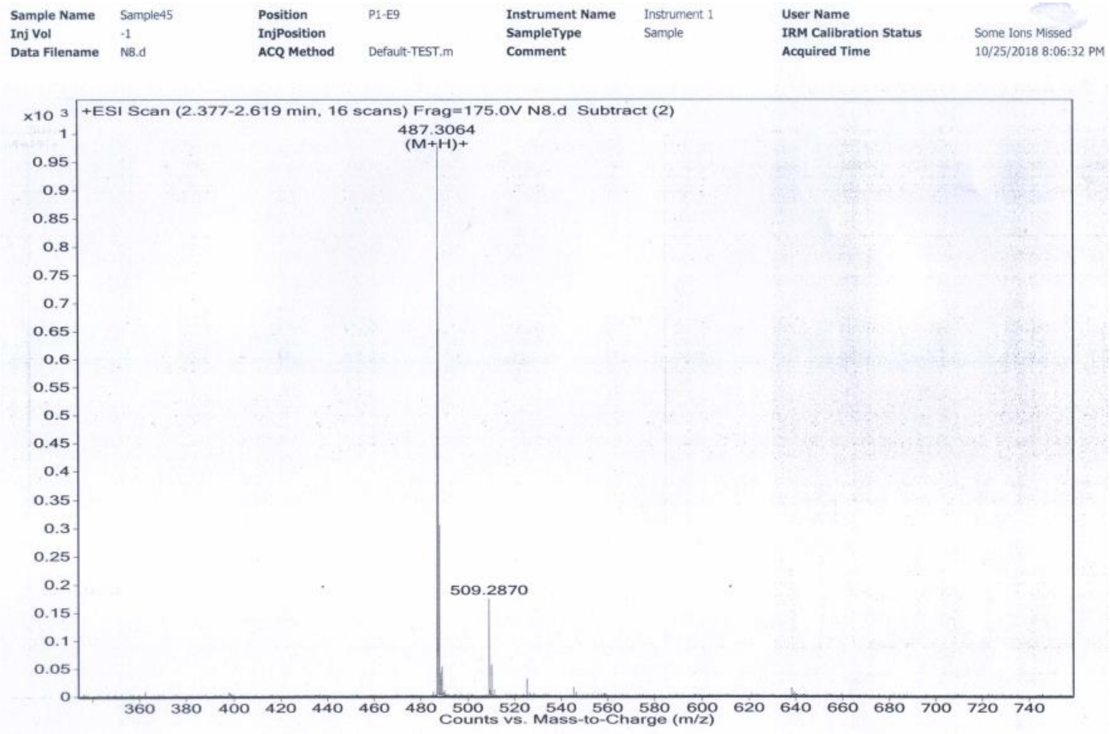

Figure S30. HRMS of compound **23e**

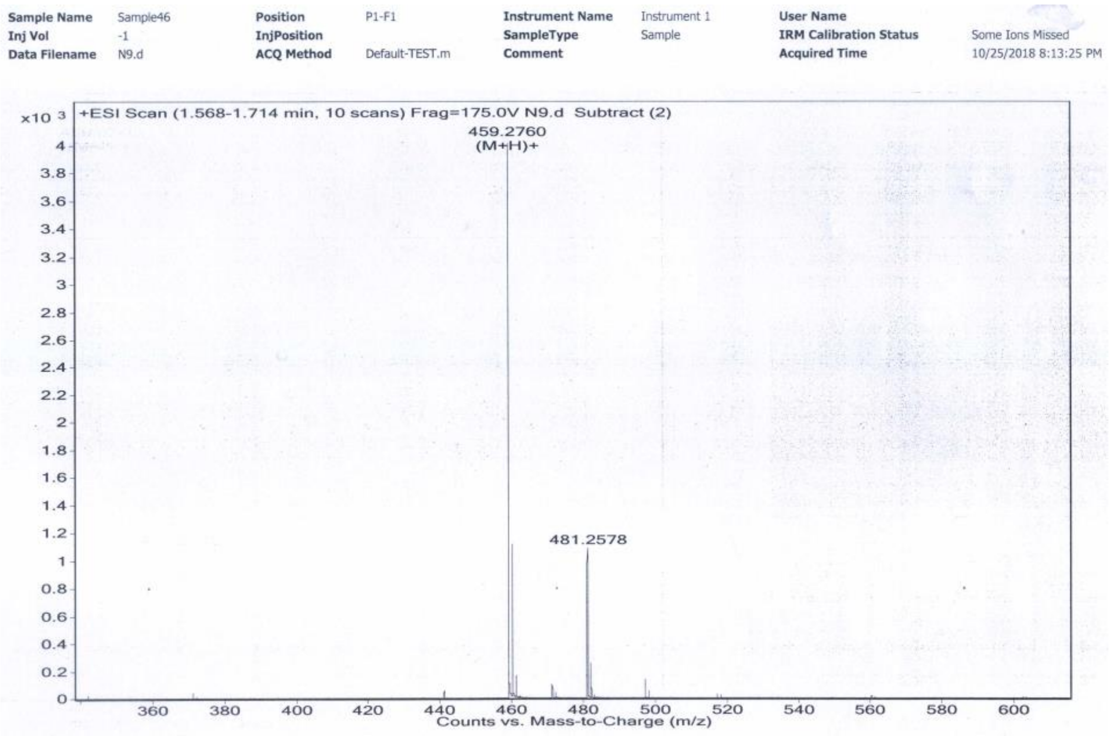

Figure S31. HRMS of compound **24e**

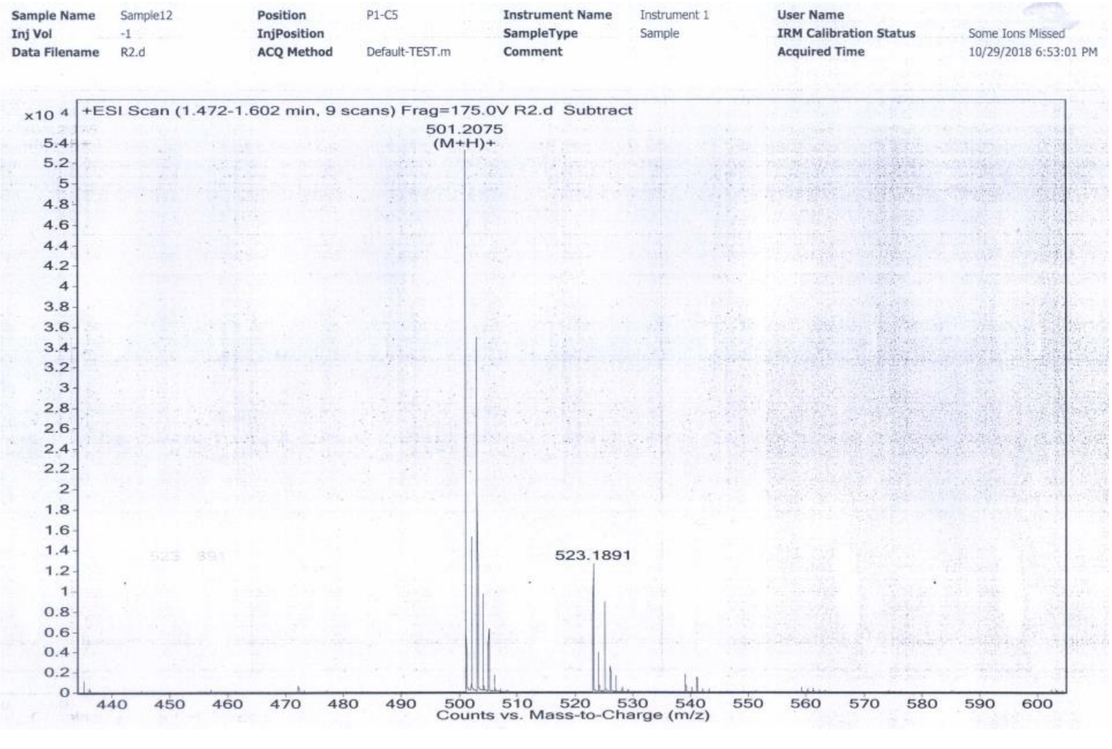

Figure S32. HRMS of compound **25e**

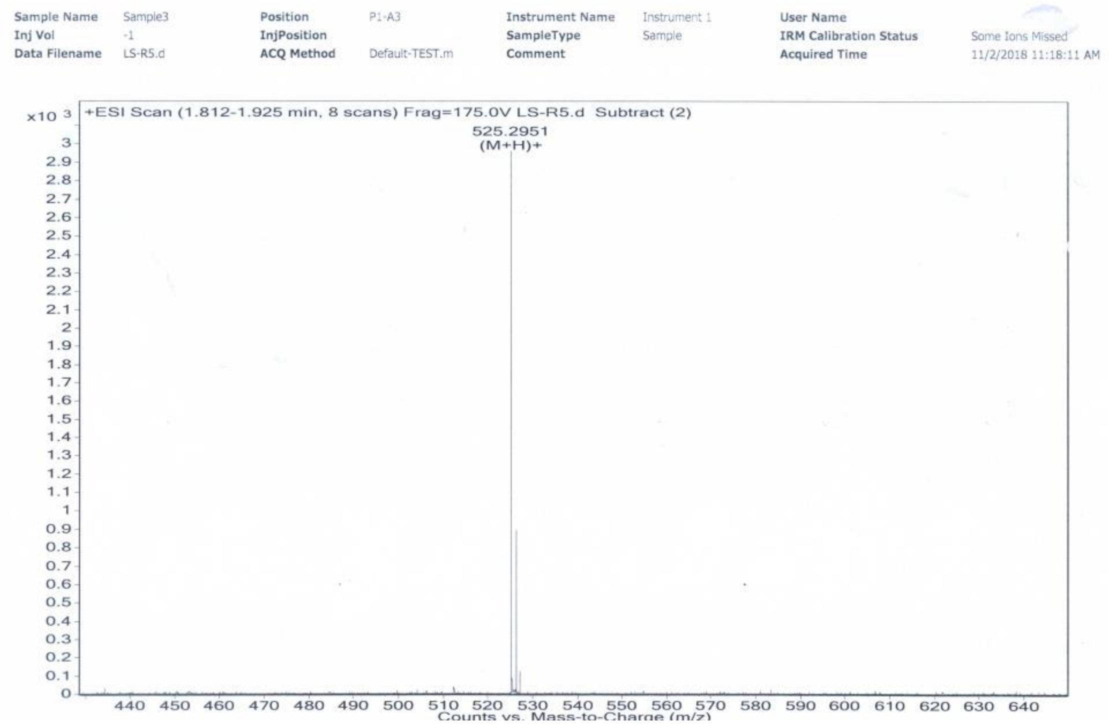

Figure S33. HRMS of compound **26e**

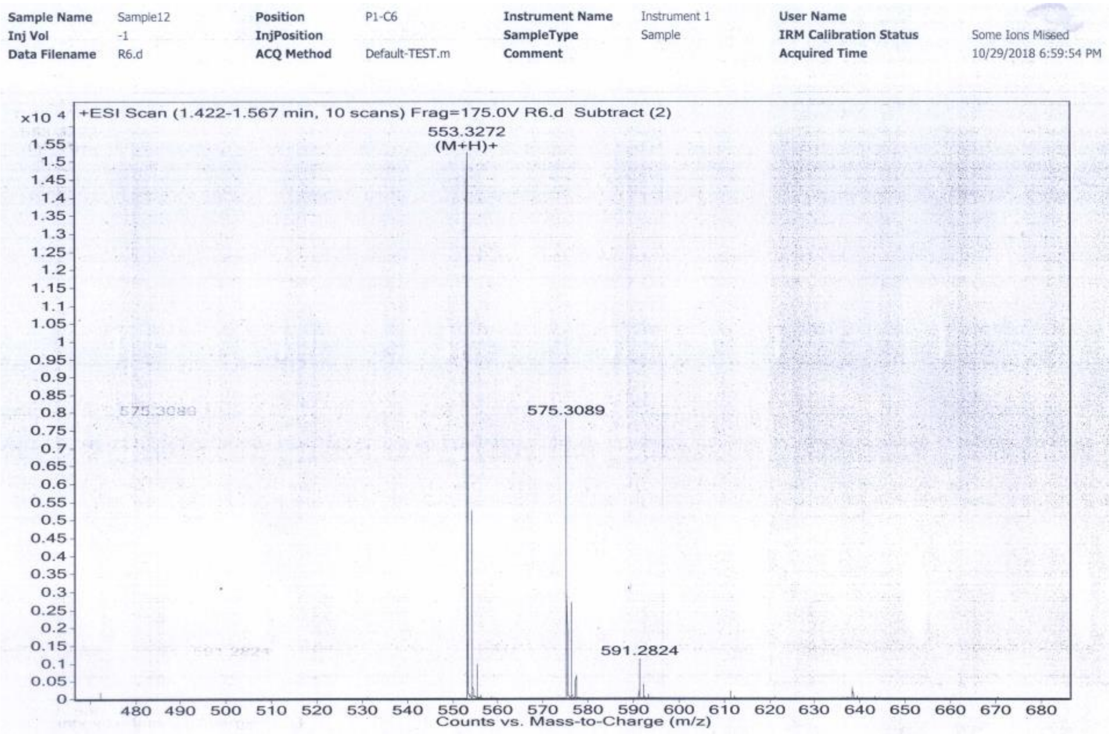

Figure S34. HRMS of compound **27e**

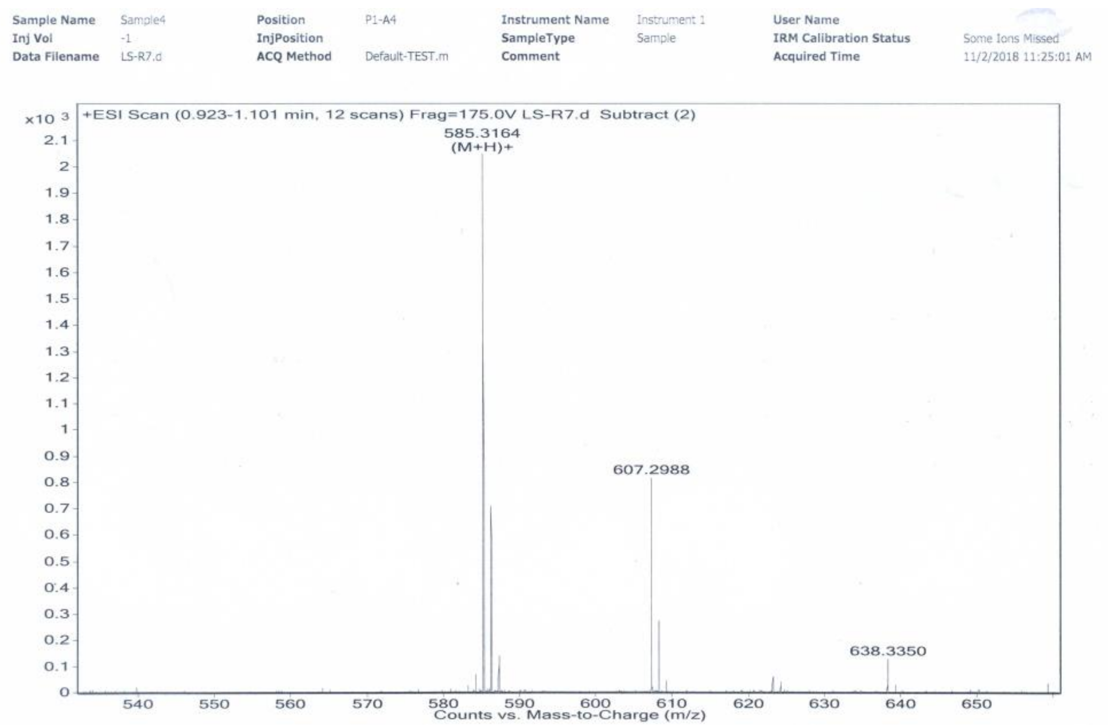

Figure S35. HRMS of compound **28e**

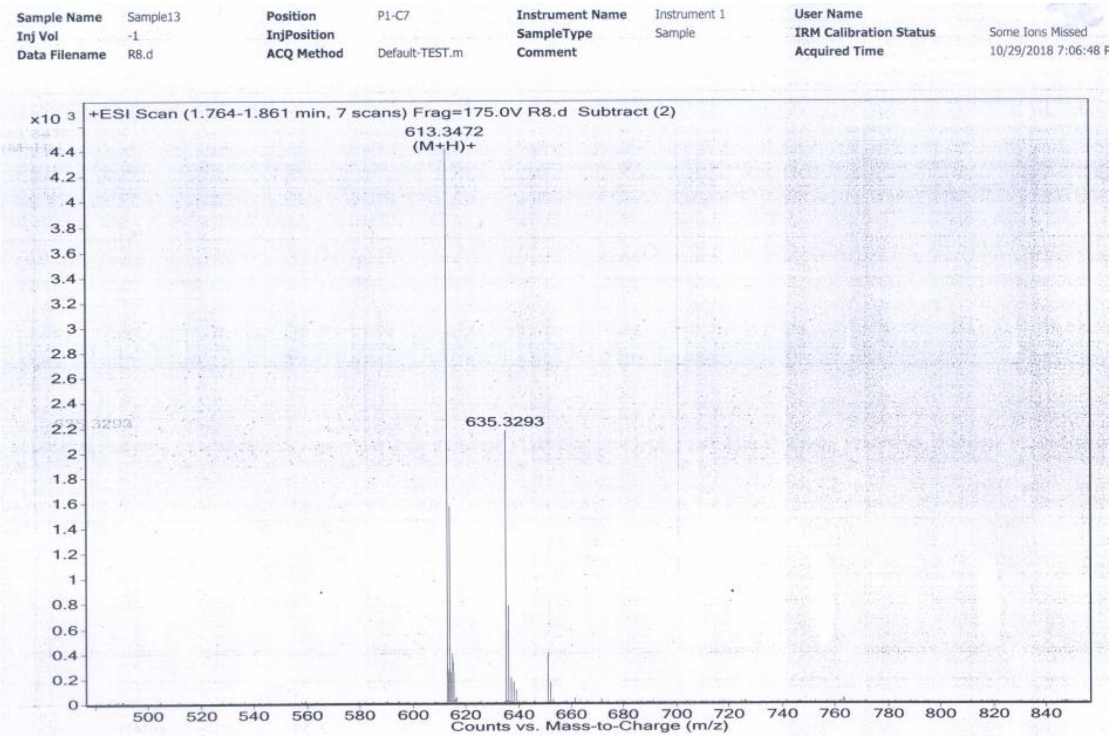

Figure S36. HRMS of compound **29e**

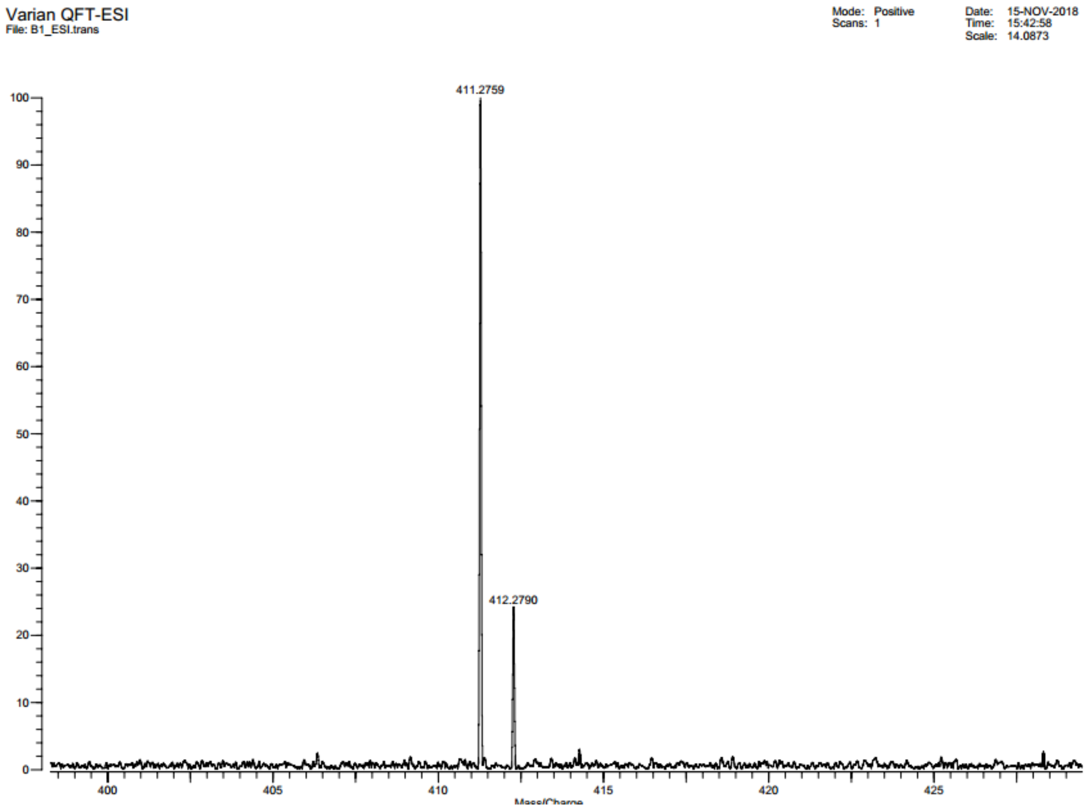

Figure S37. HRMS of compound **30e**

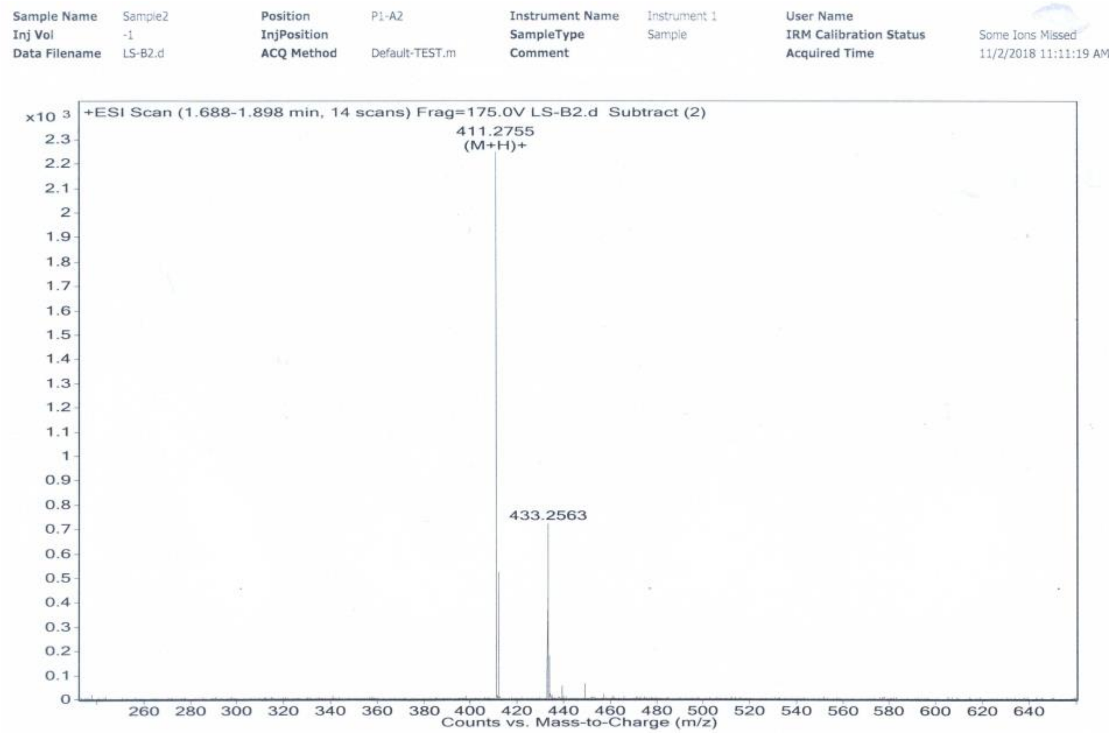

Figure S38. HRMS of compound **31e**

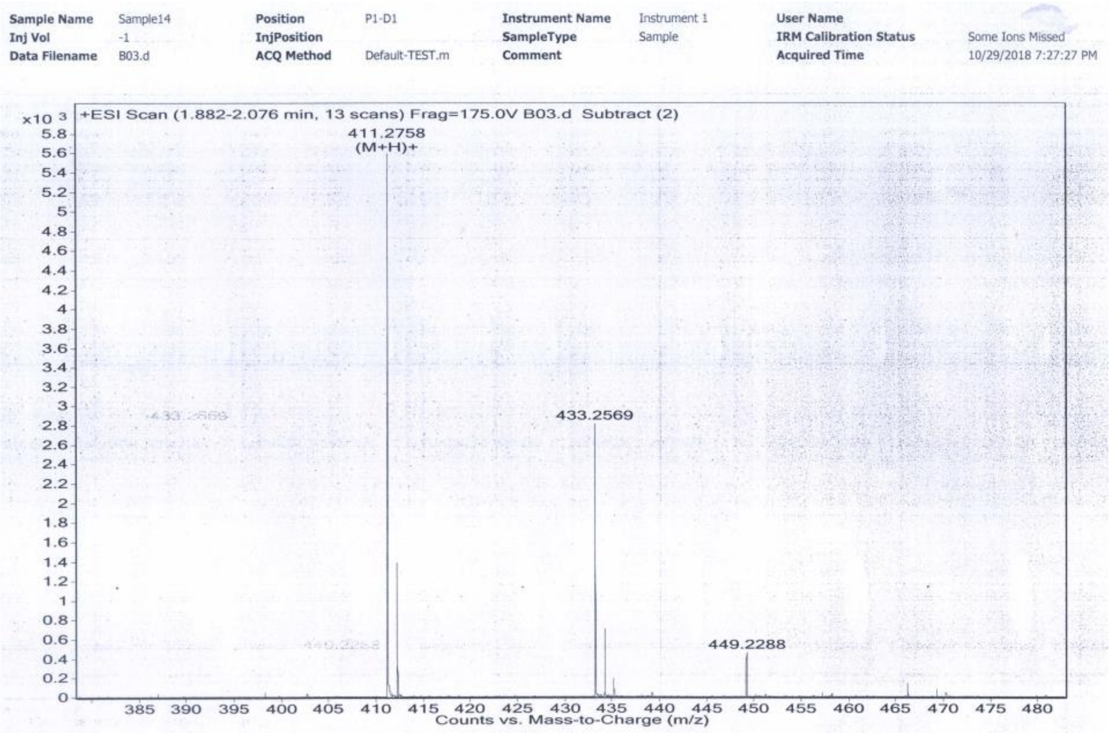

Figure S39. HRMS of compound **32e**

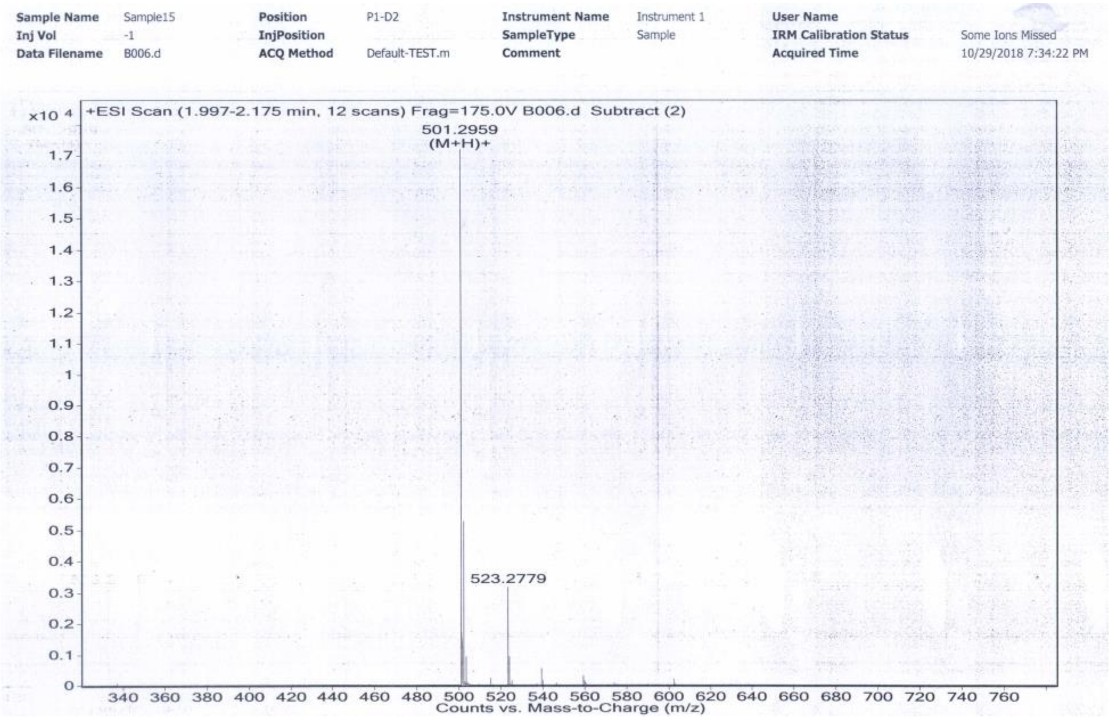

Figure S40. HRMS of compound **33e**

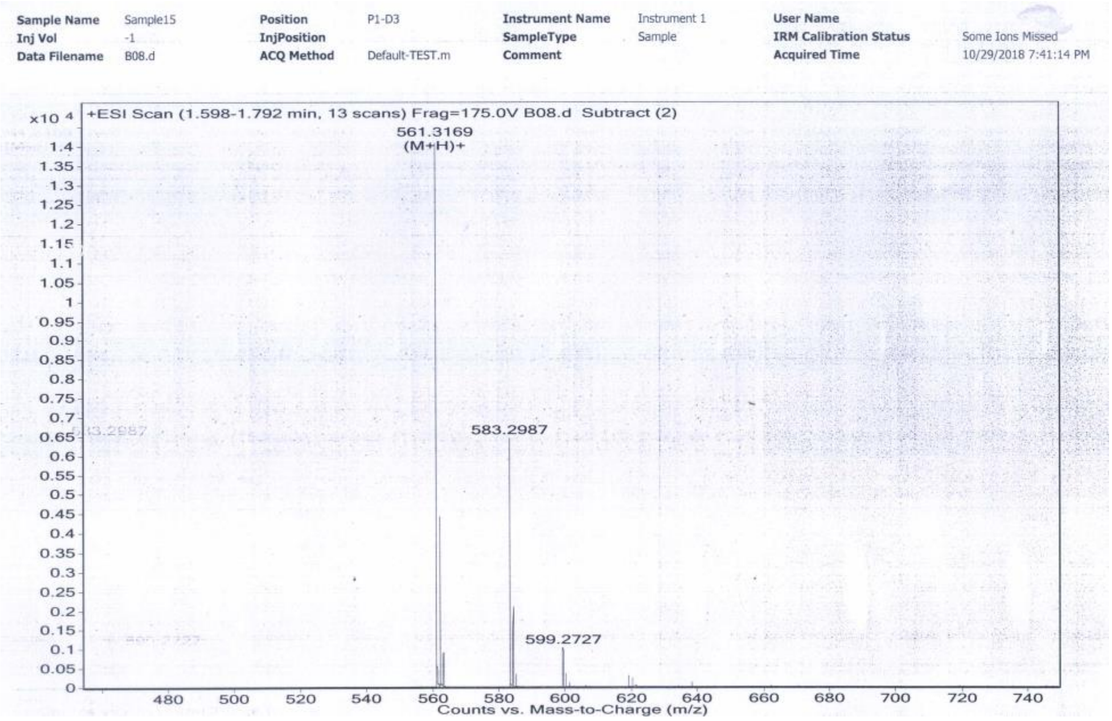

Figure S41. HRMS of compound **34e**

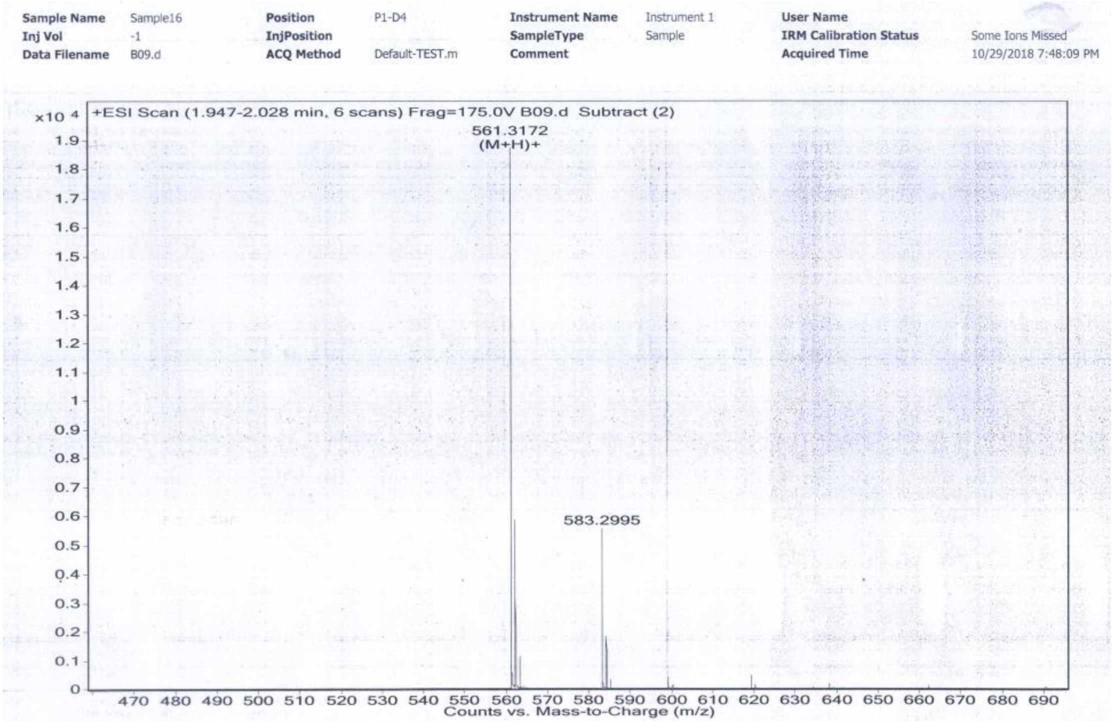

Figure S42. HRMS of compound **35e**

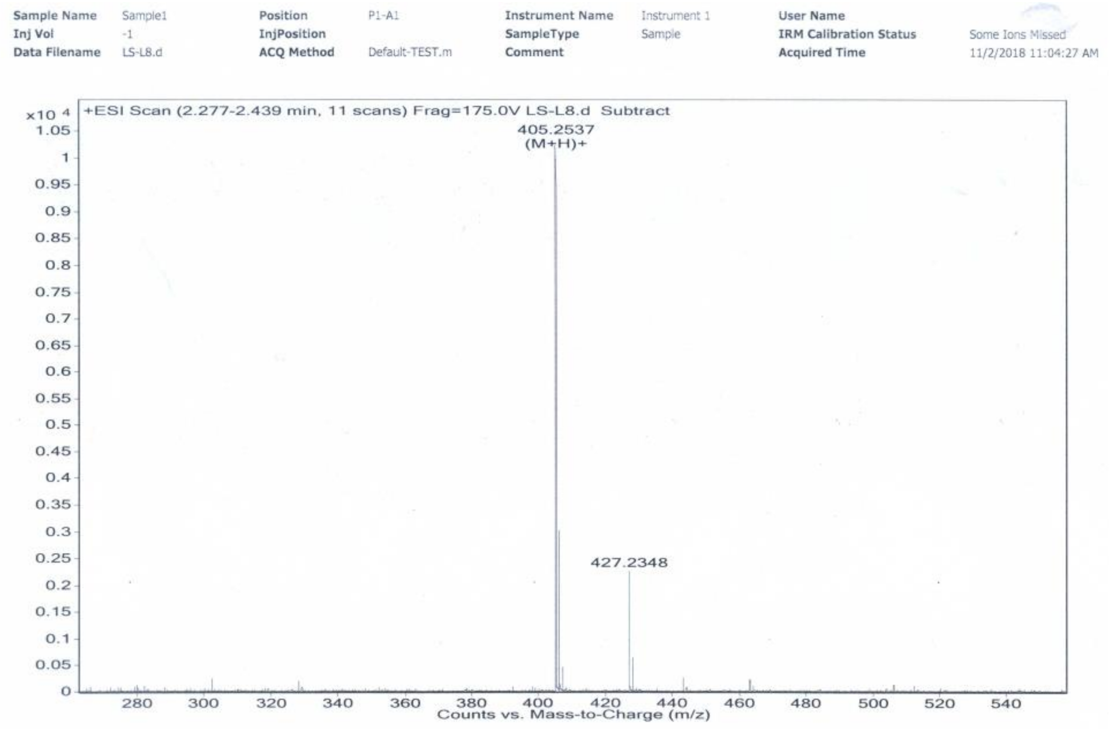

Figure S43. HRMS of compound **9d**

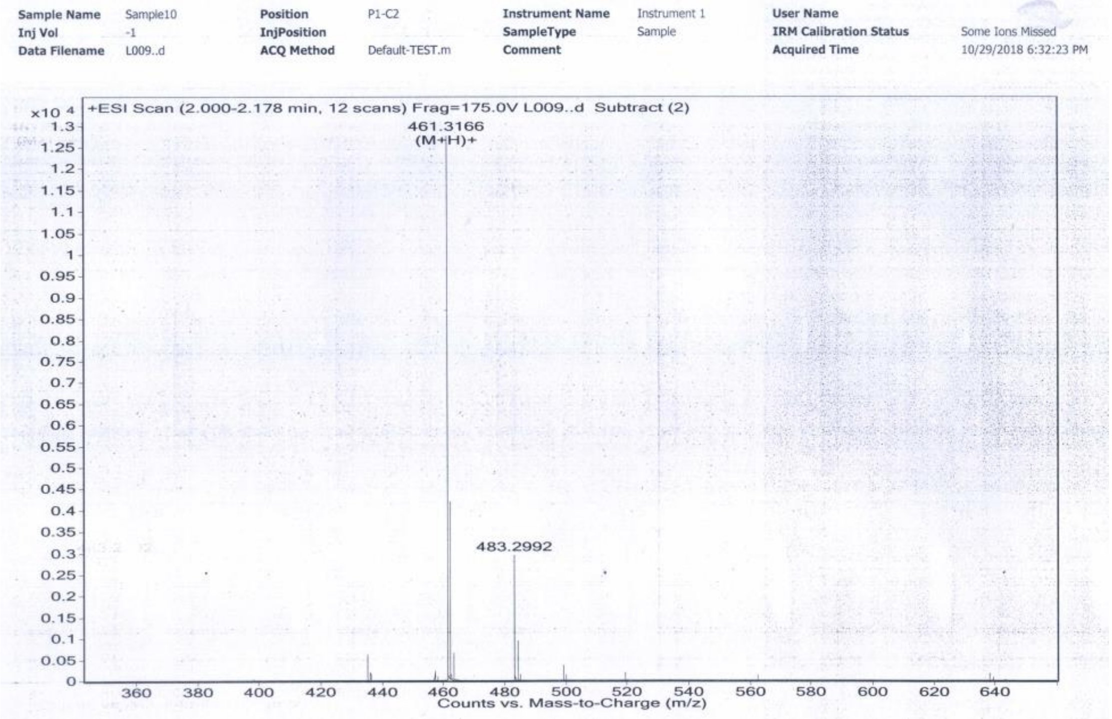

Figure S44. HRMS of compound **9f**

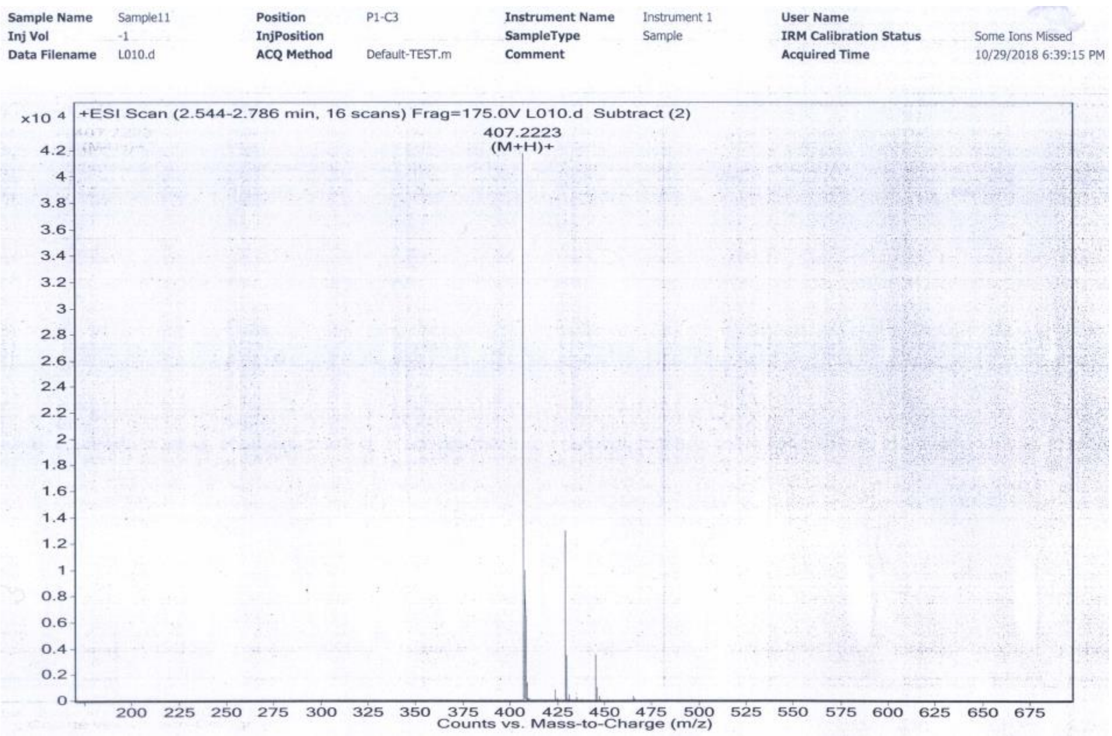

Figure S45. HRMS of compound **9g**

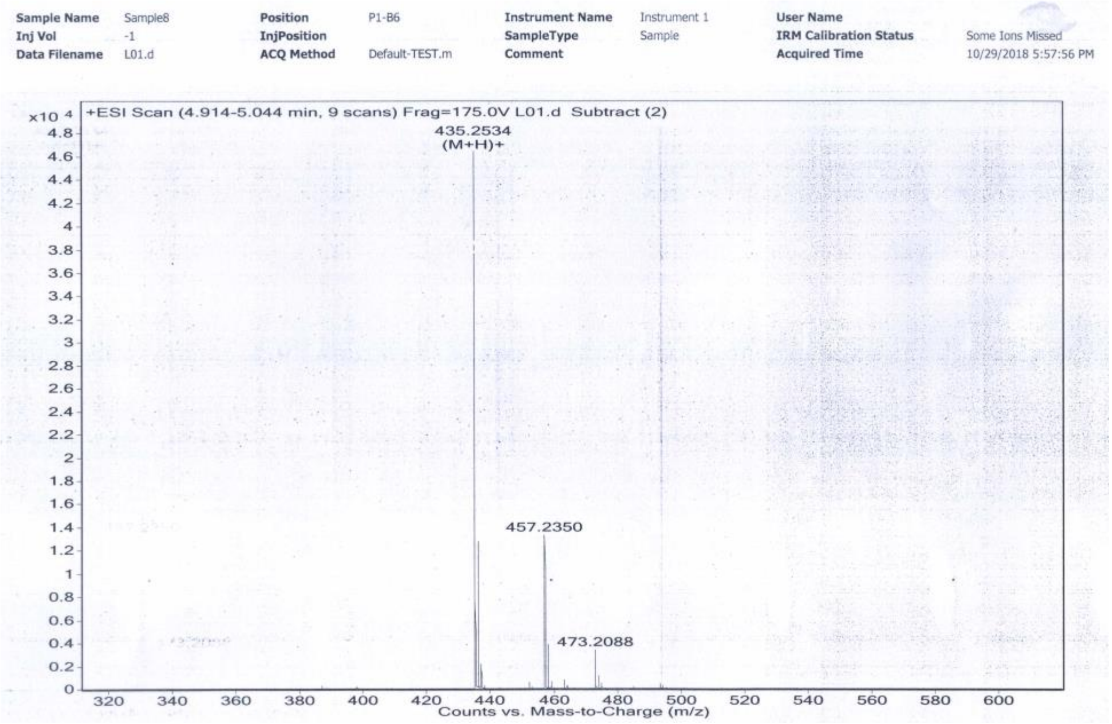

Figure S46. HRMS of compound **9h**

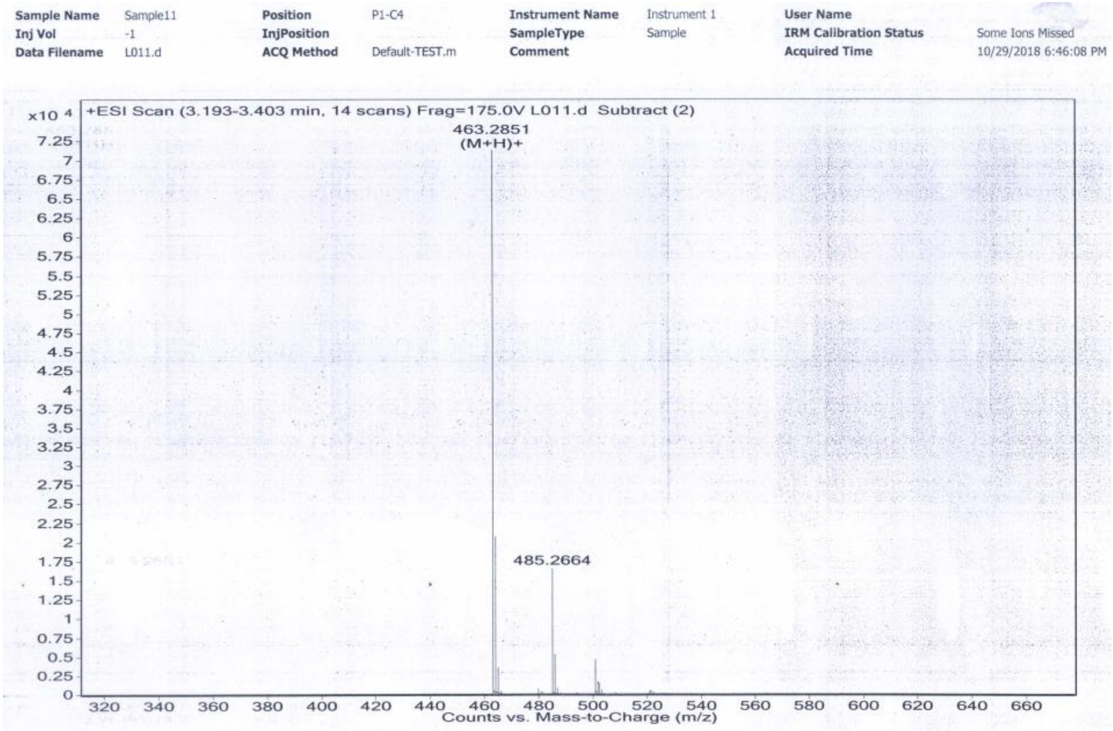

Figure S47. HRMS of compound **9i**

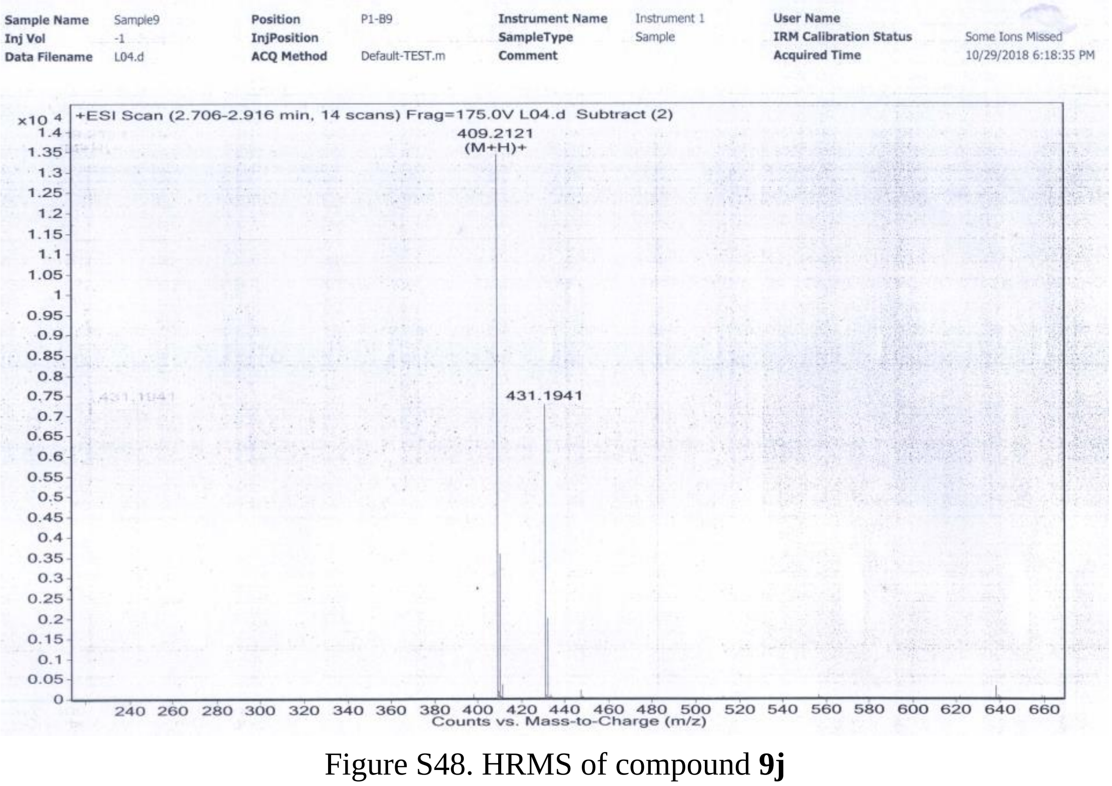

Figure S48. HRMS of compound **9j**

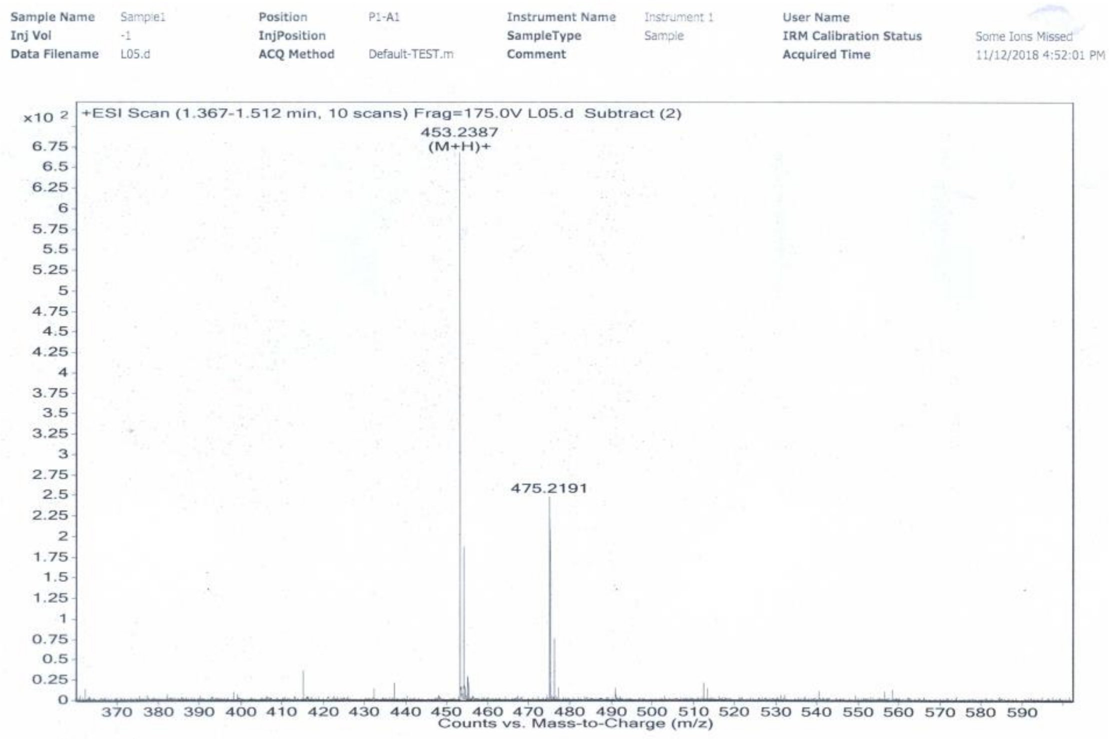

Figure S49. HRMS of compound **9k**

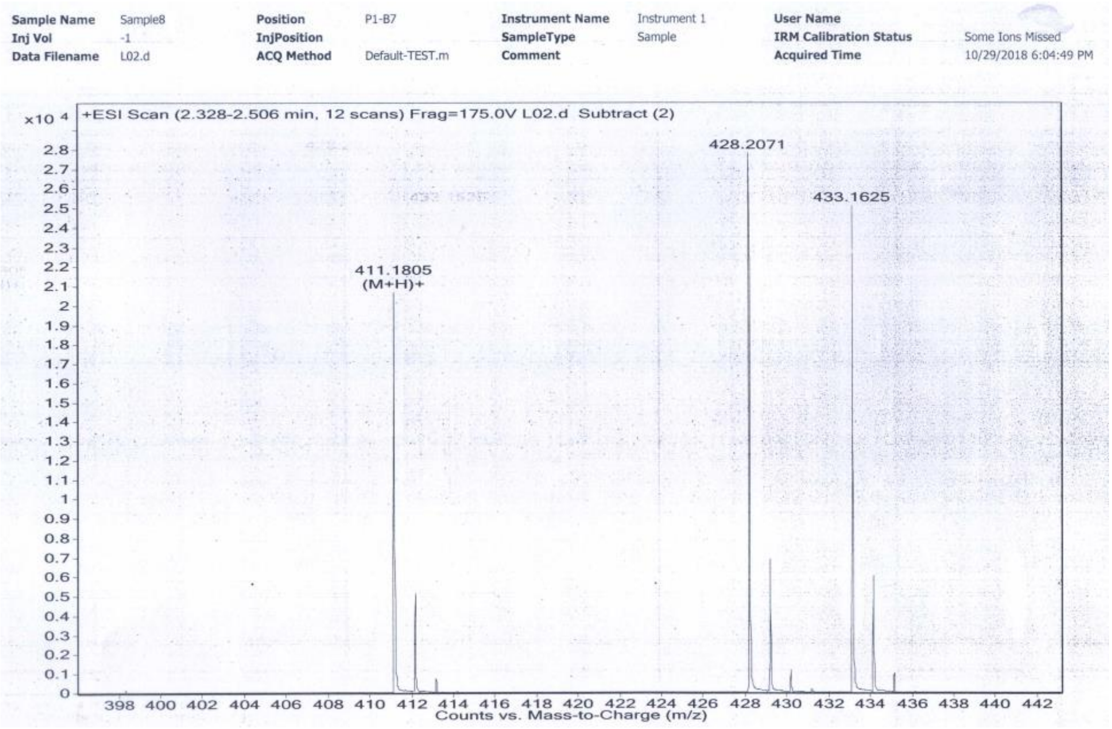

Figure S50. HRMS of compound **9l**

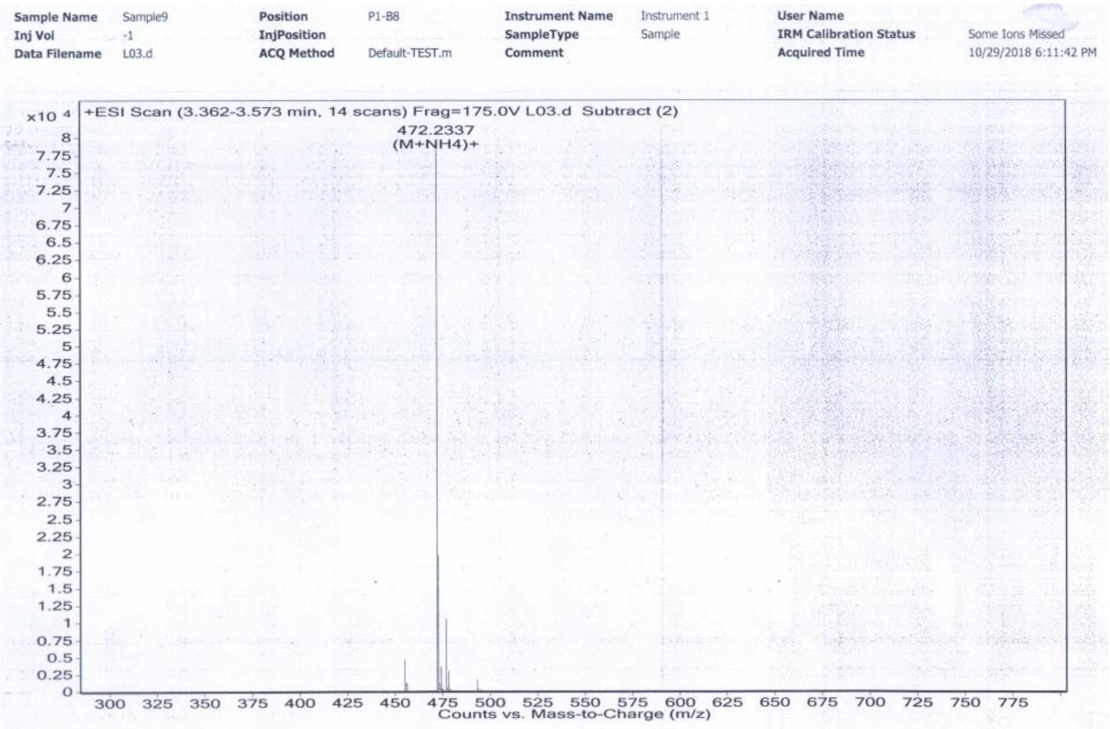

Figure S51. HRMS of compound **9m**

1H-NMR

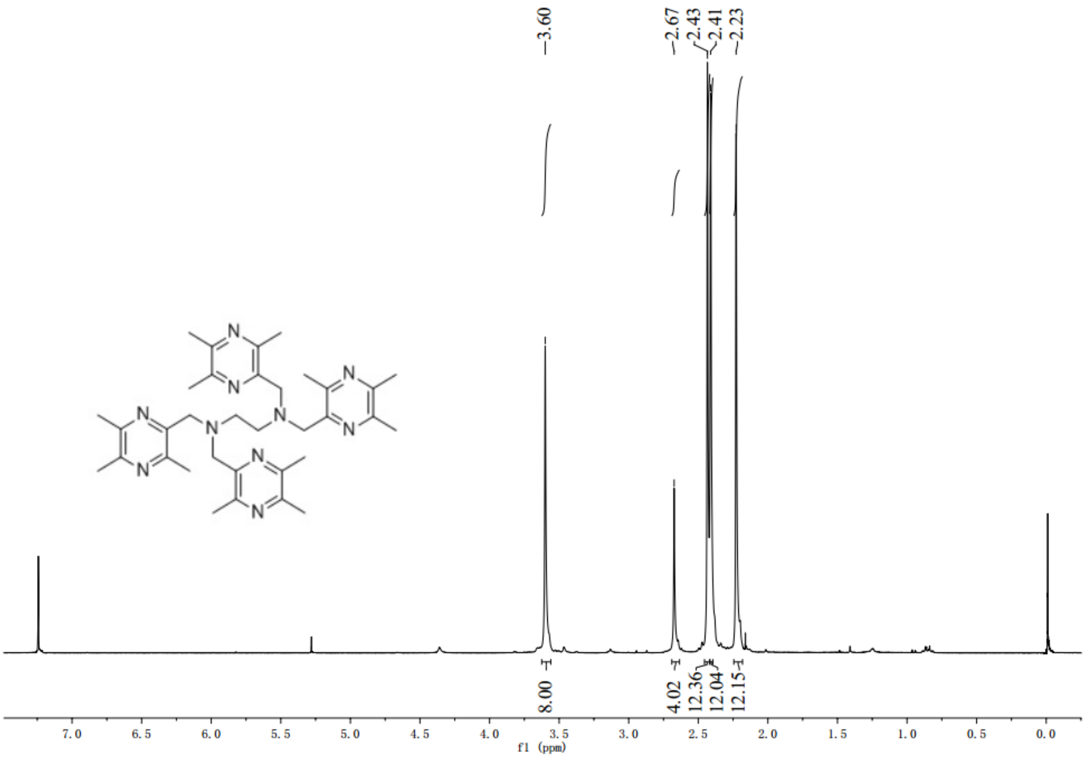

Figure S52 ^{1}H NMR spectrum of compound **6a.**

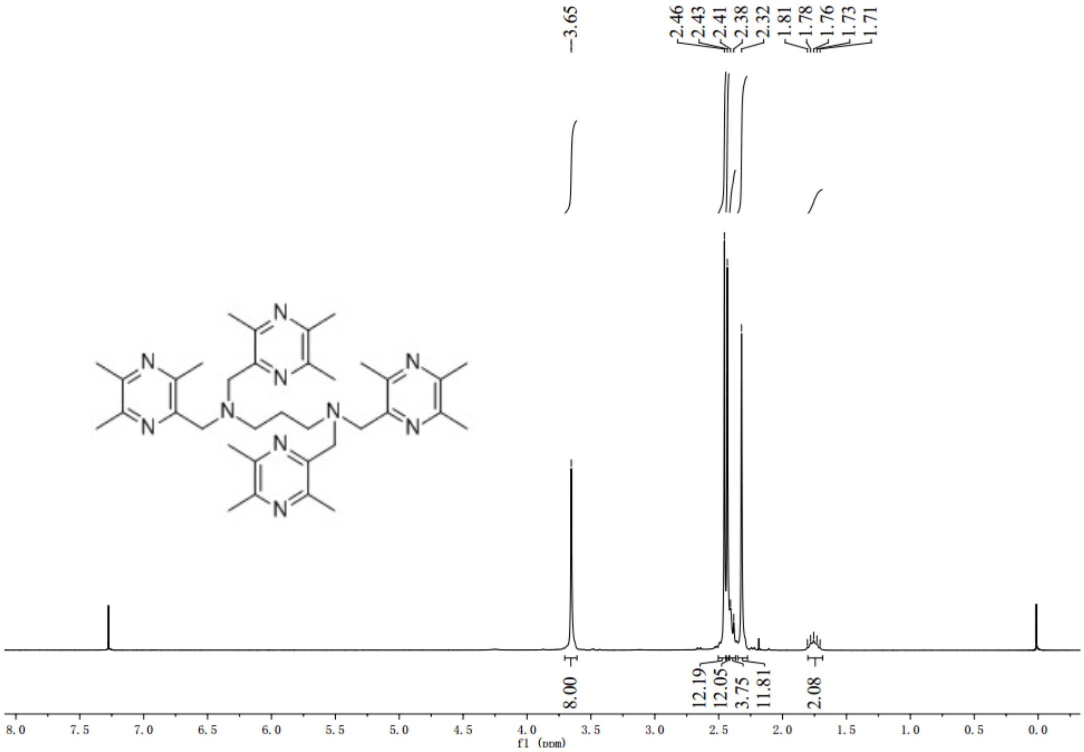

Figure S53 ^{1}H NMR spectrum of compound **6b.**

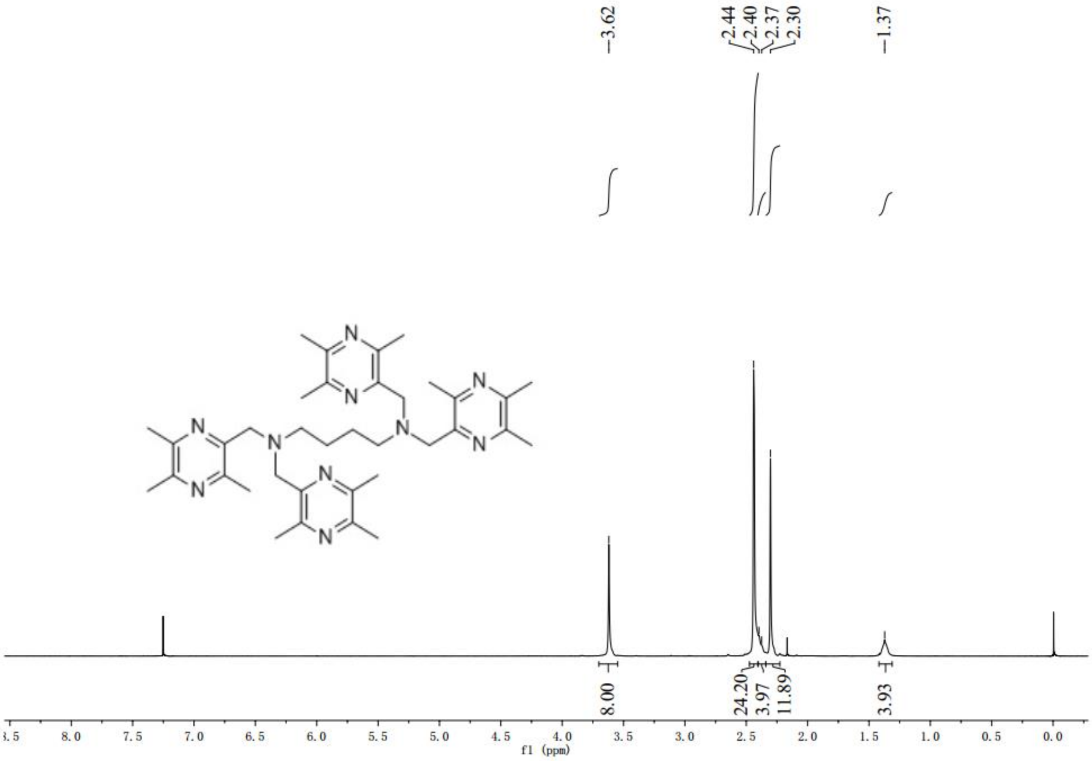

Figure S54 ^{1}H NMR spectrum of compound **6c.**

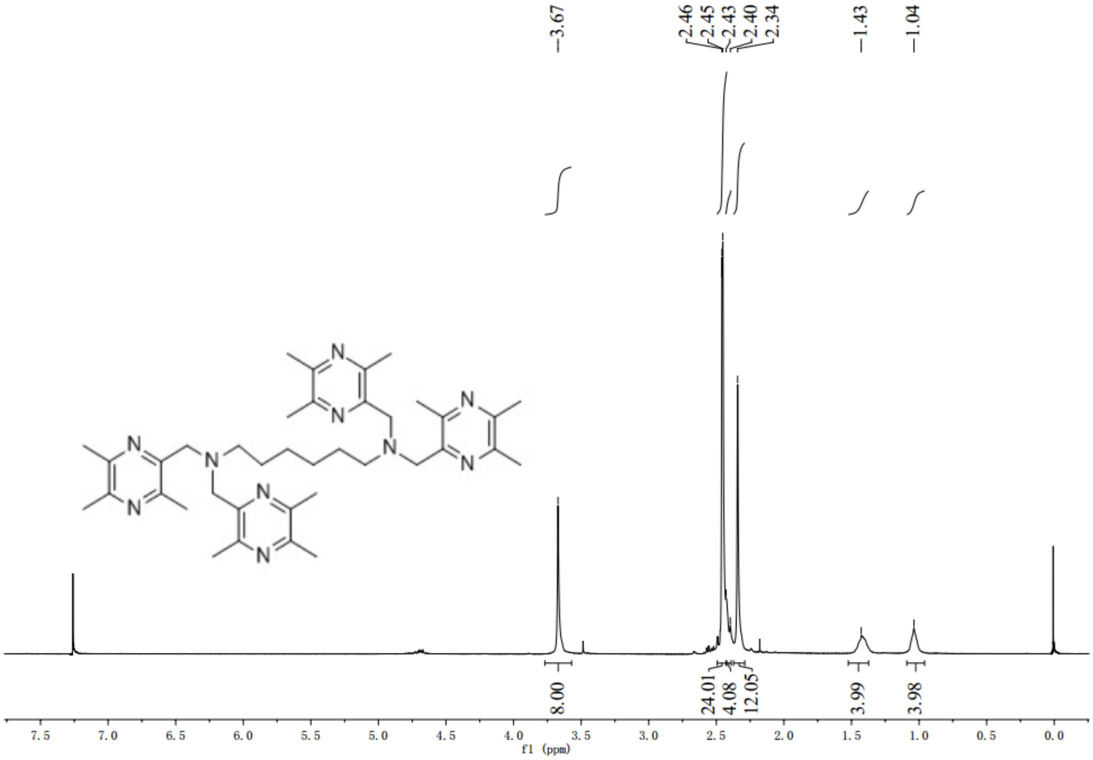

Figure S55 ^{1}H NMR spectrum of compound **6d.**

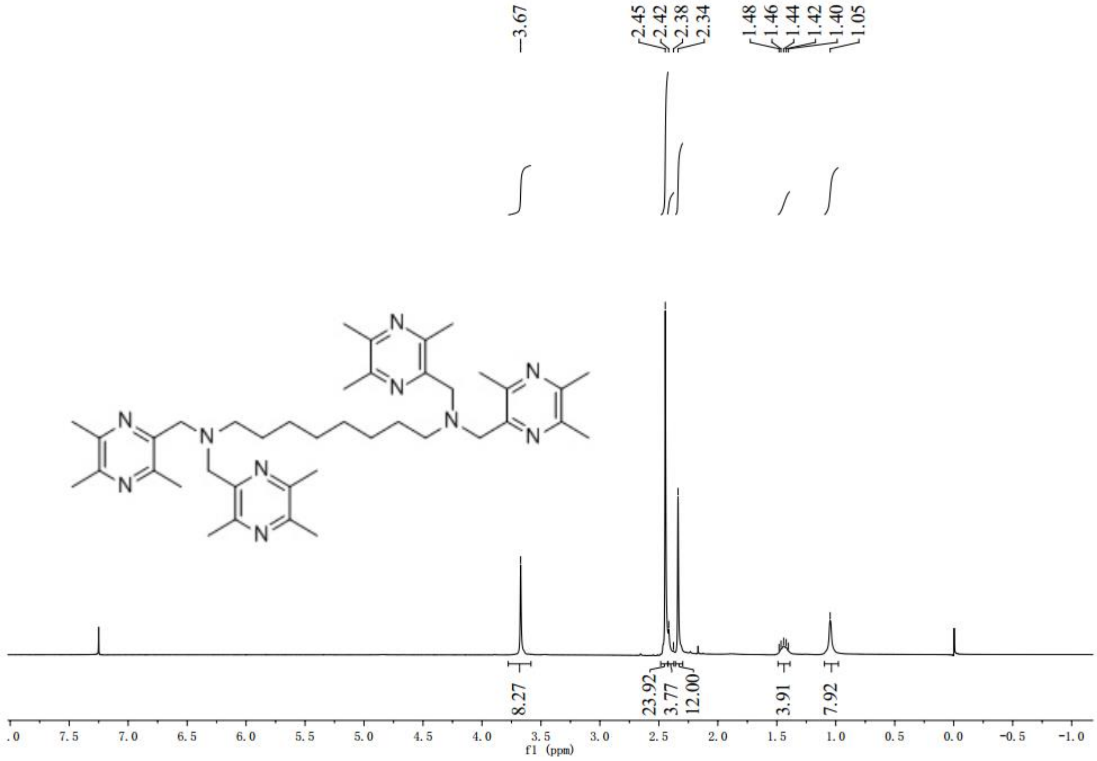

Figure S56 ^{1}H NMR spectrum of compound **6e.**

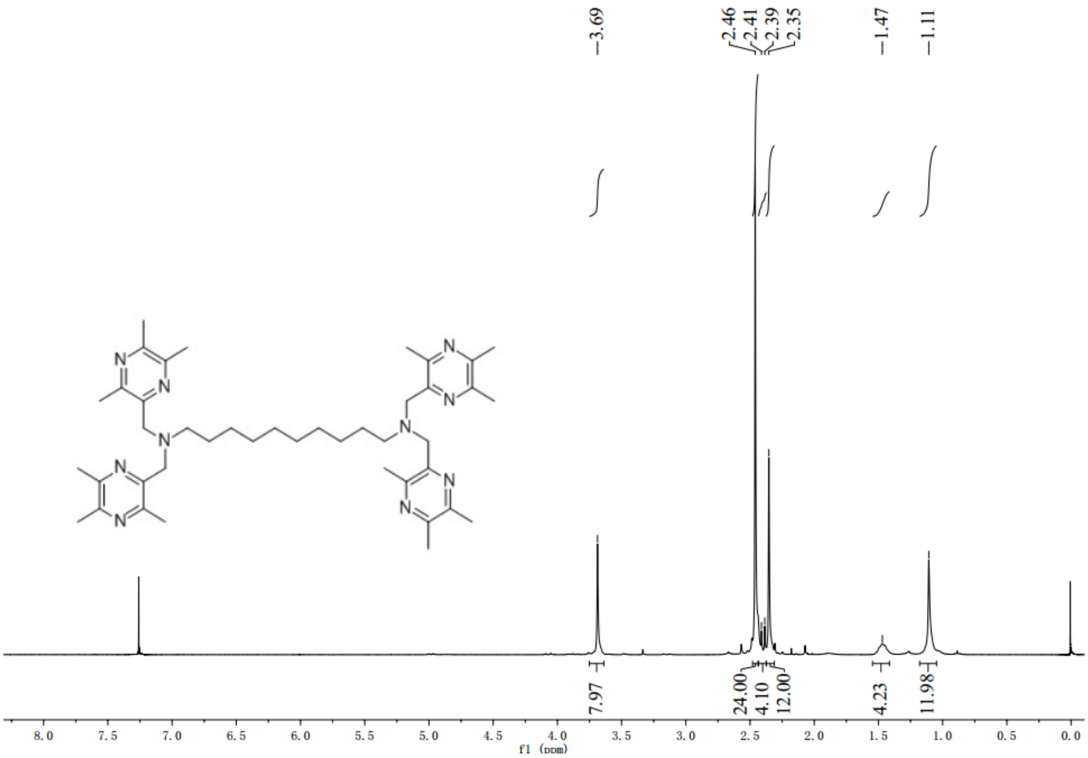

Figure S57 ¹H NMR spectrum of compound **6f.**

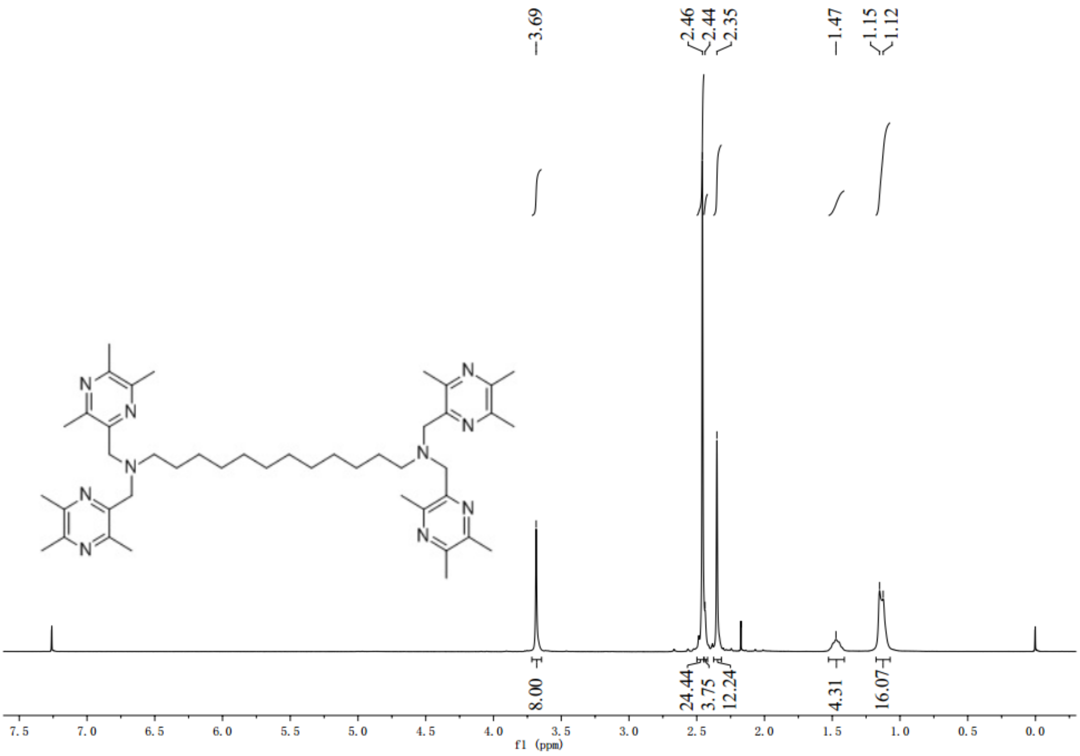

Figure S58 ¹H NMR spectrum of compound **6g.**

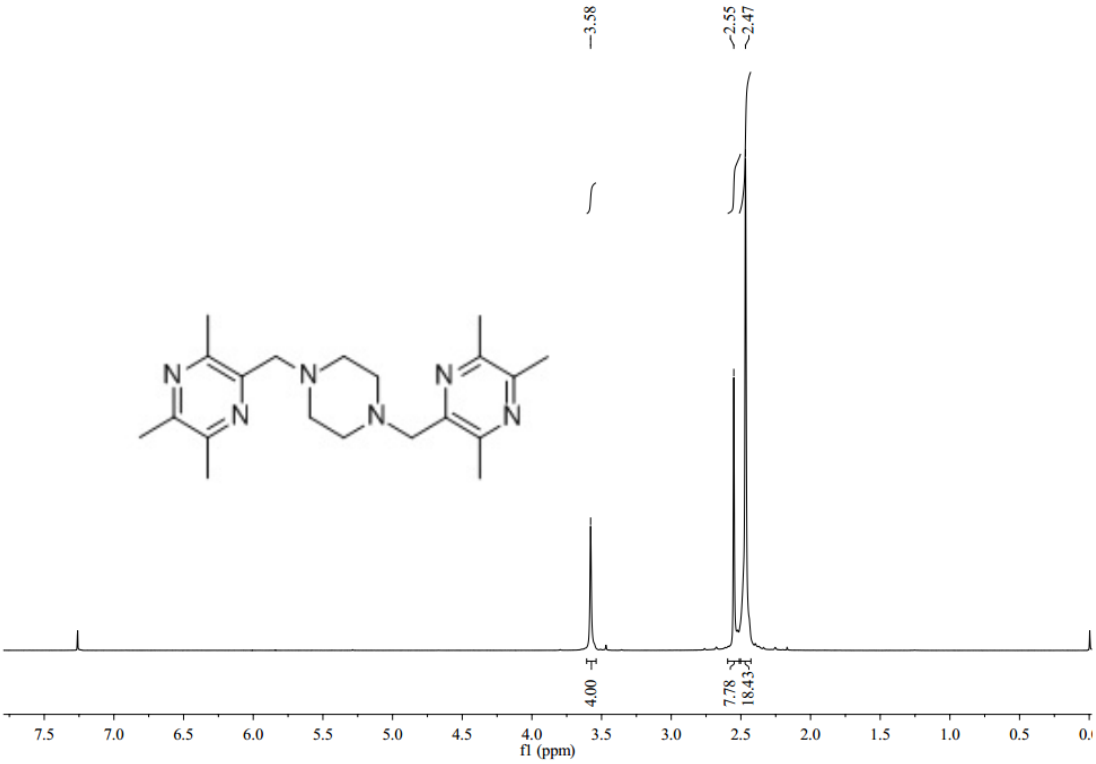

Figure S59 ^{1}H NMR spectrum of compound **6h.**

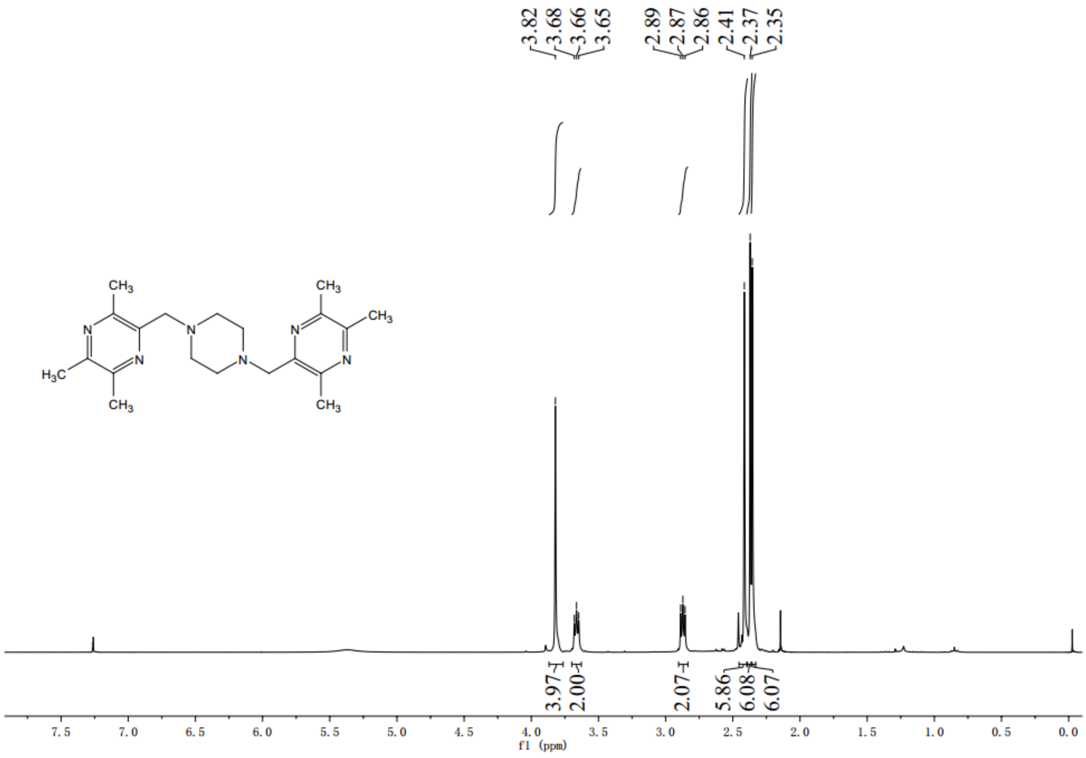

Figure S60 ^{1}H NMR spectrum of compound **6i.**

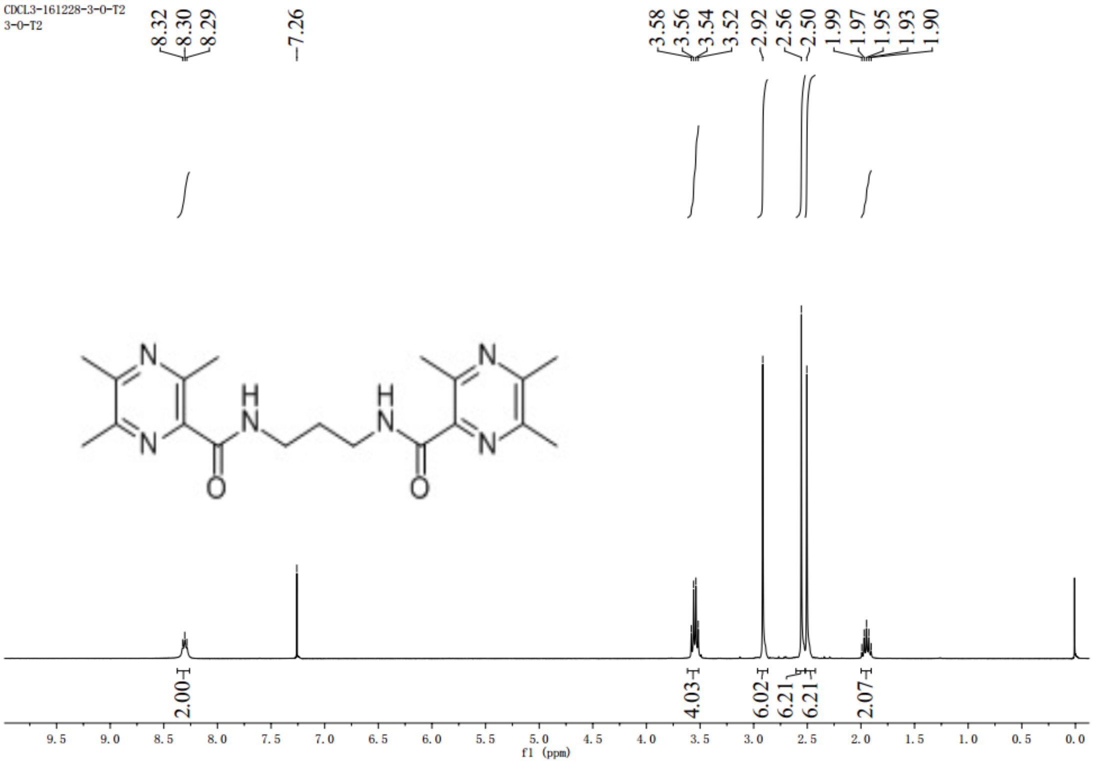

Figure S61 ^{1}H NMR spectrum of compound **8a.**

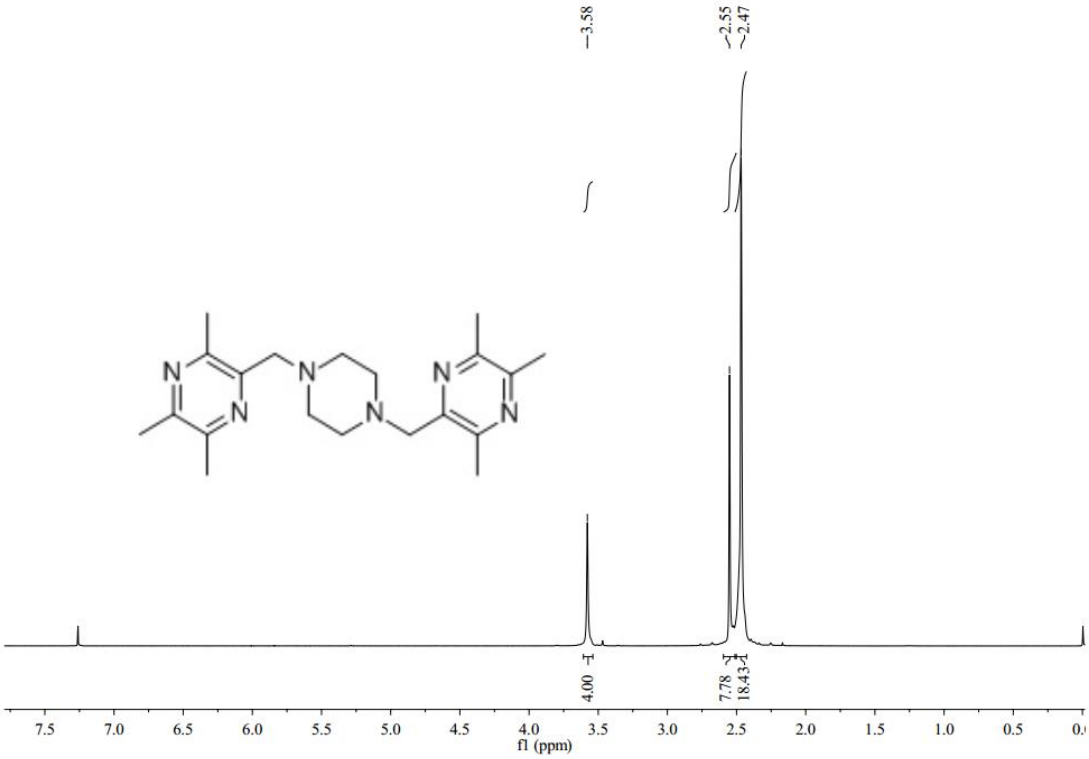

Figure S62 ^{1}H NMR spectrum of compound **8b.**

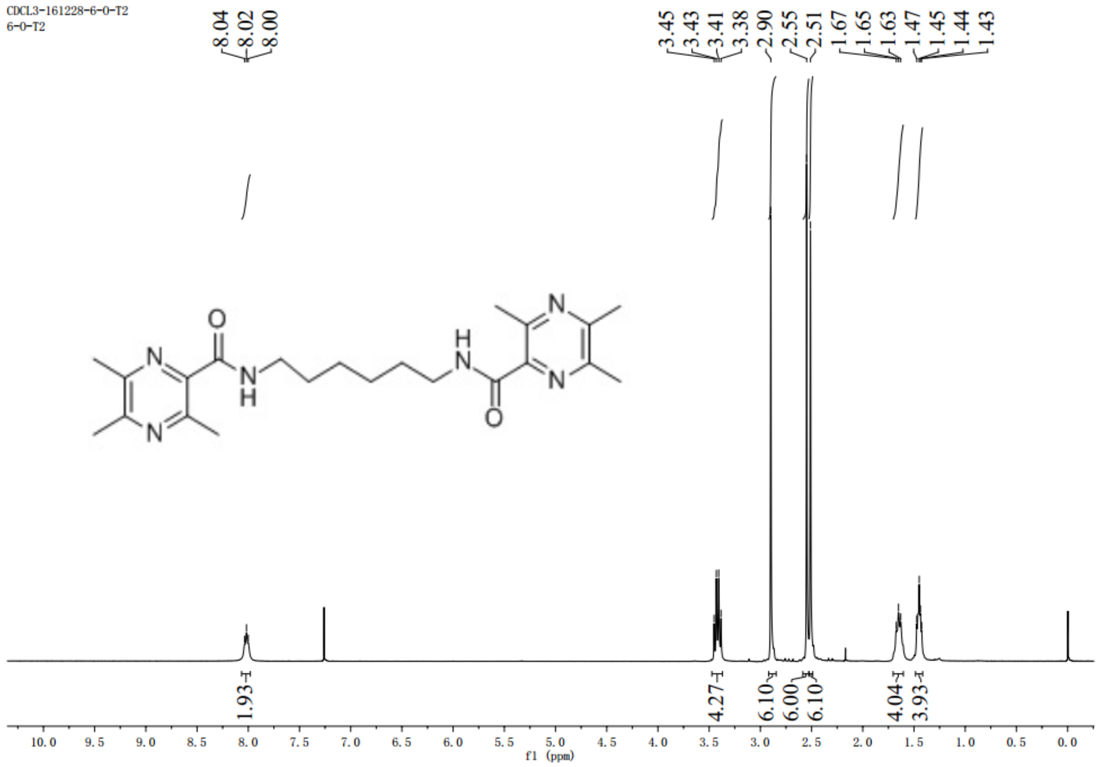

Figure S63 ^{1}H NMR spectrum of compound **8c.**

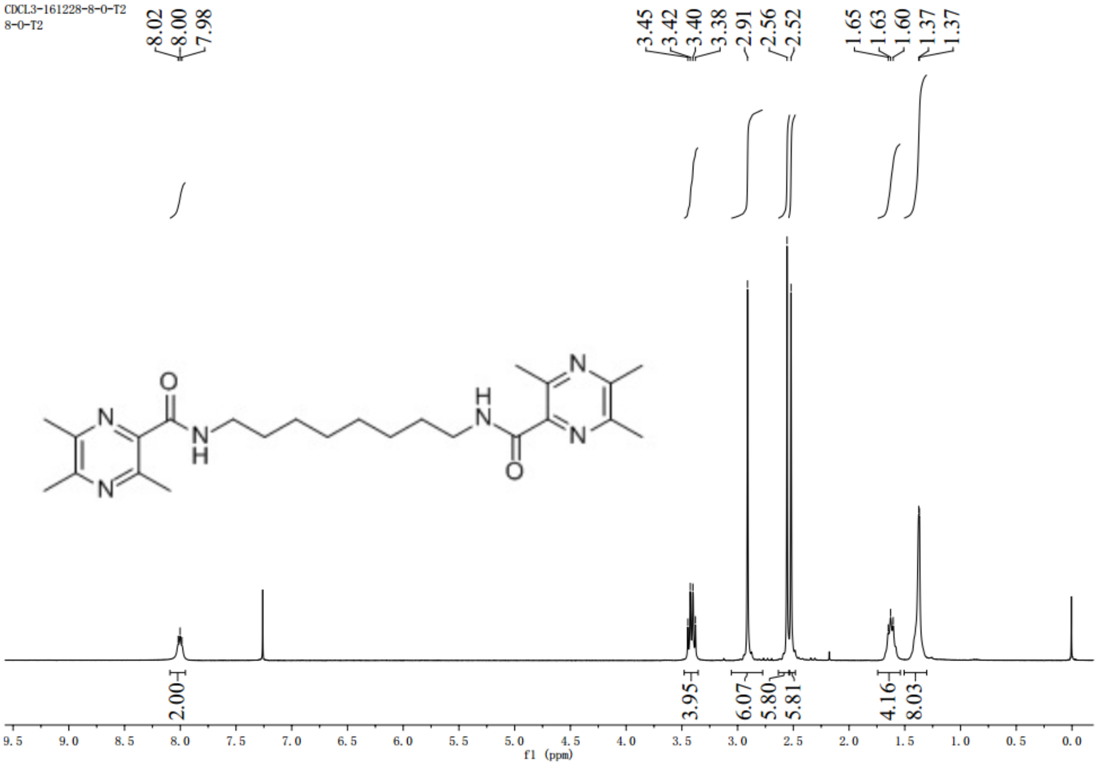

Figure S64 ^{1}H NMR spectrum of compound **8d.**

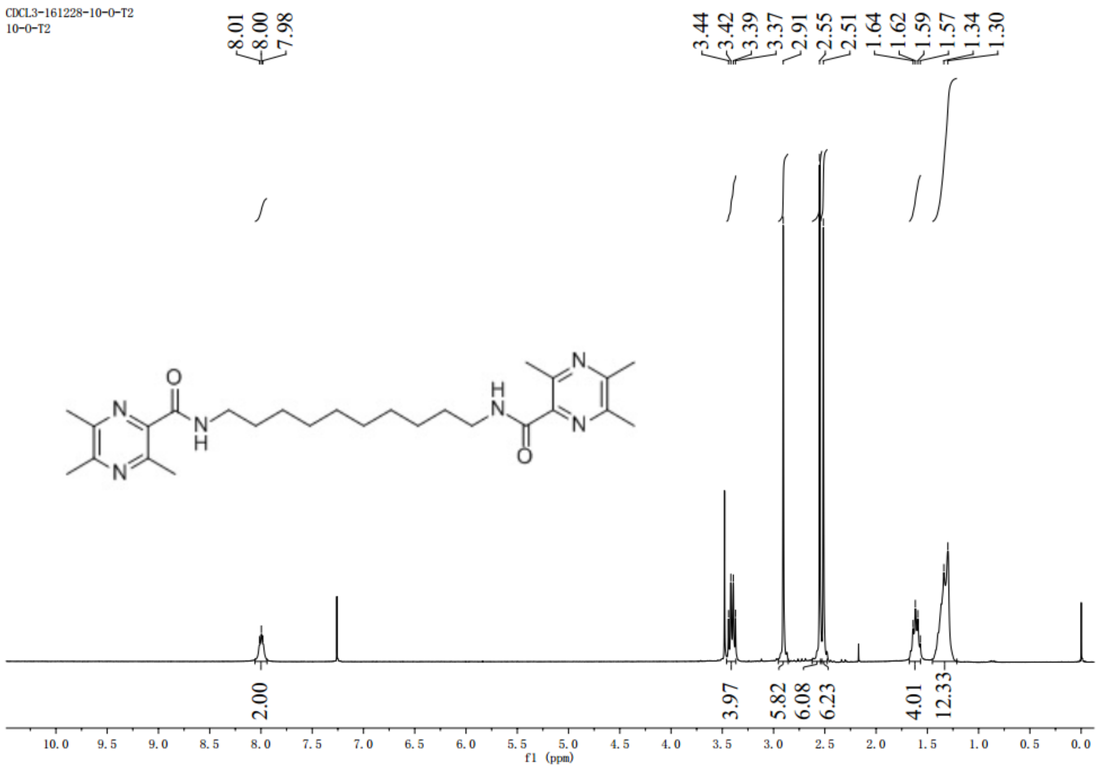

Figure S65 ^{1}H NMR spectrum of compound **8e.**

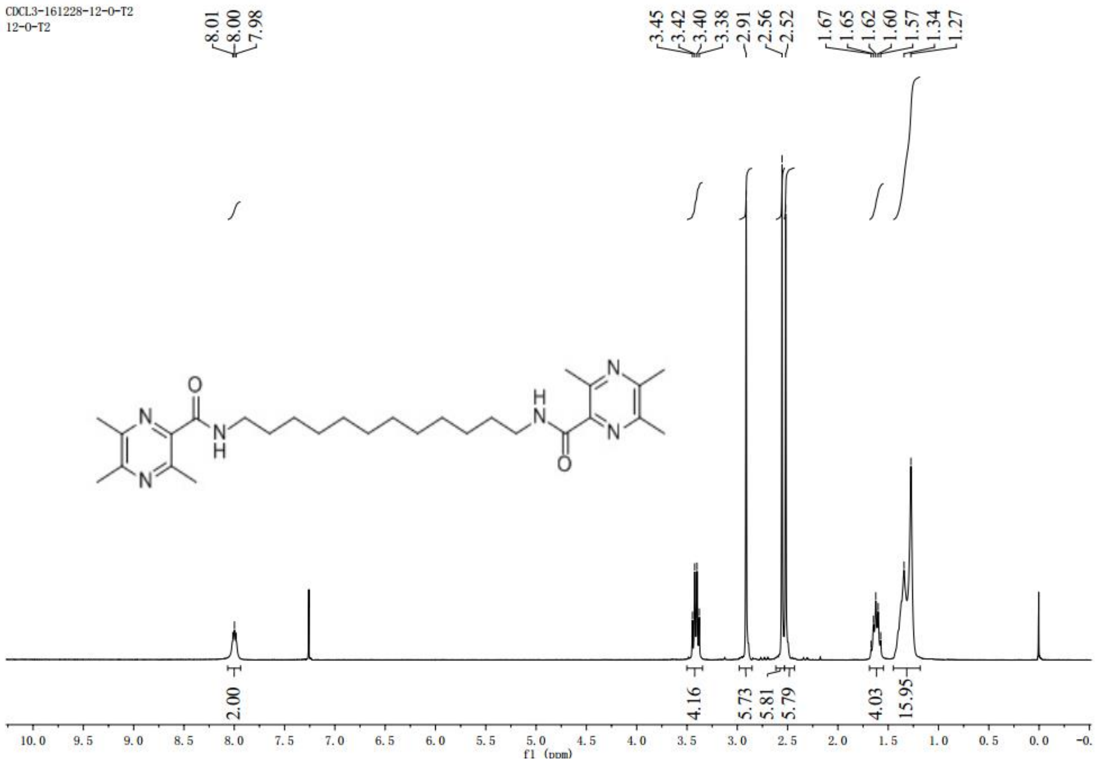

Figure S66 ^{1}H NMR spectrum of compound **8f.**

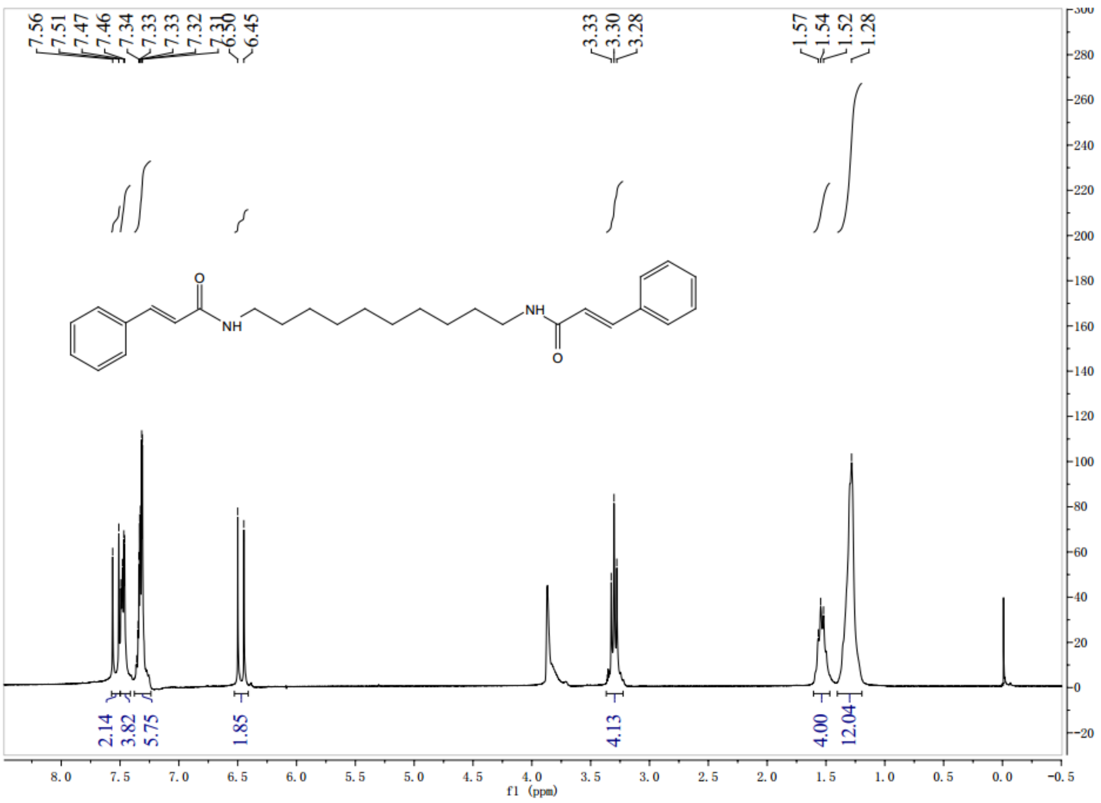

Figure S67 ^{1}H NMR spectrum of compound **9e.**

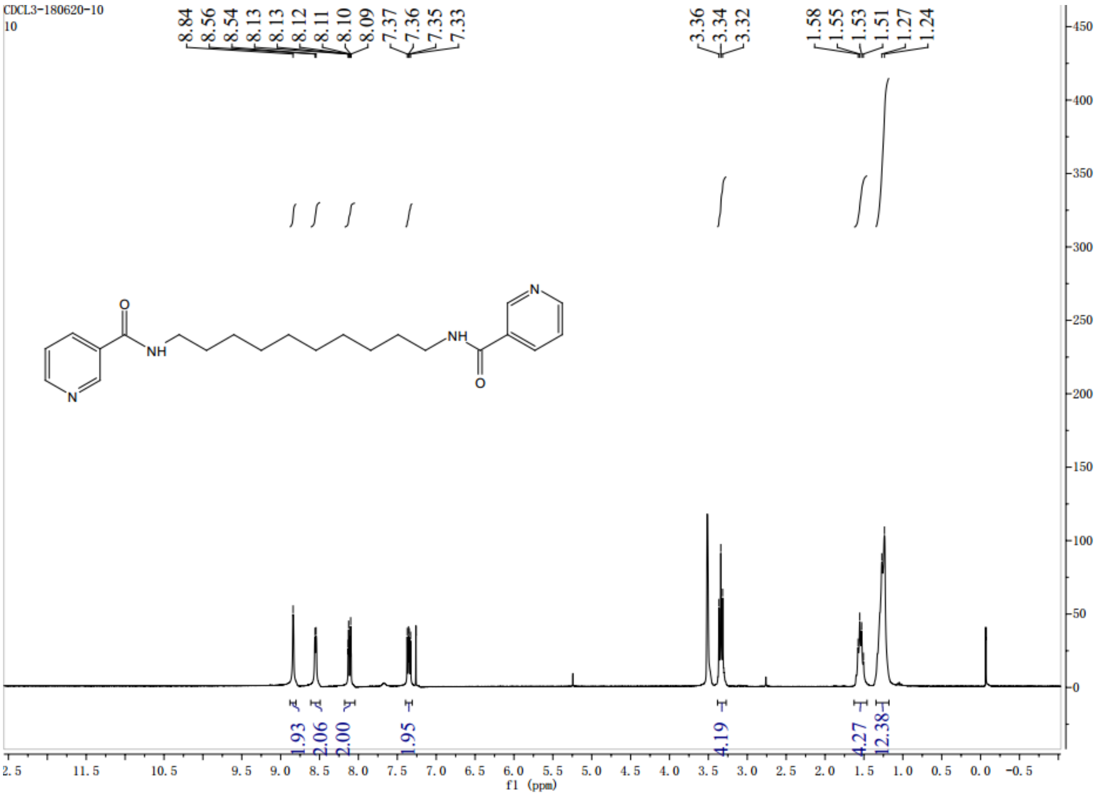

Figure S68 ^{1}H NMR spectrum of compound **10e.**

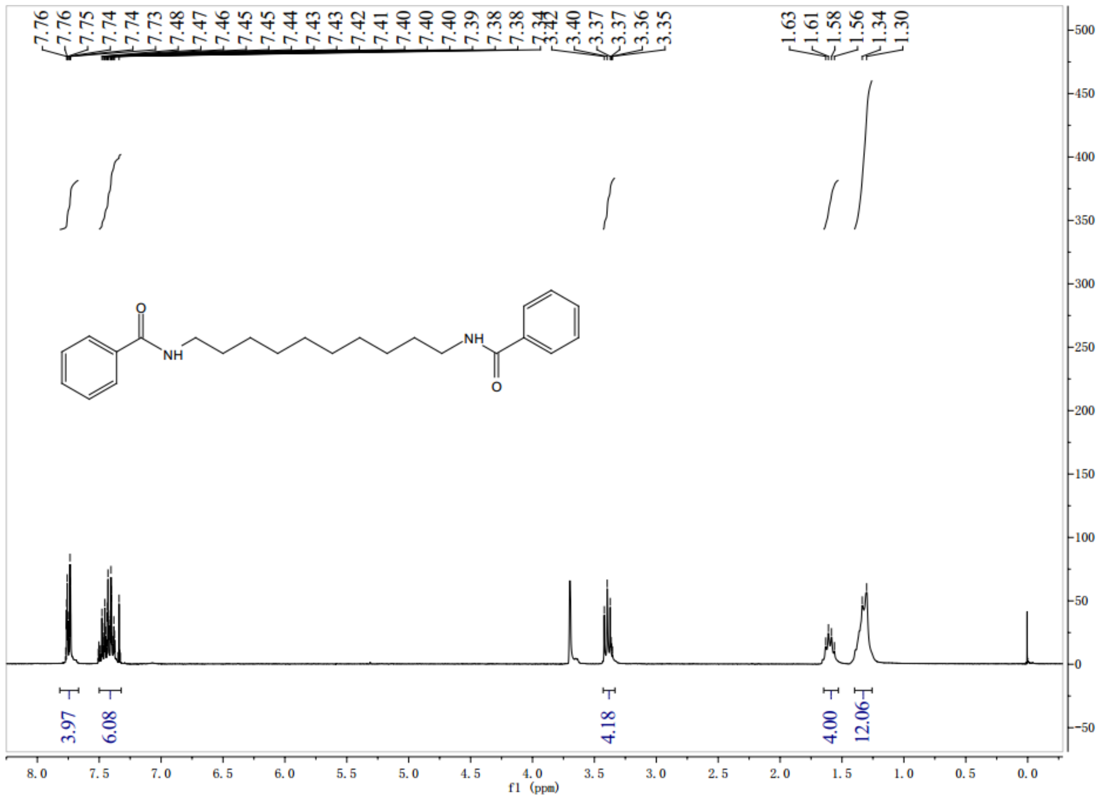

Figure S69 ^{1}H NMR spectrum of compound **11e.**

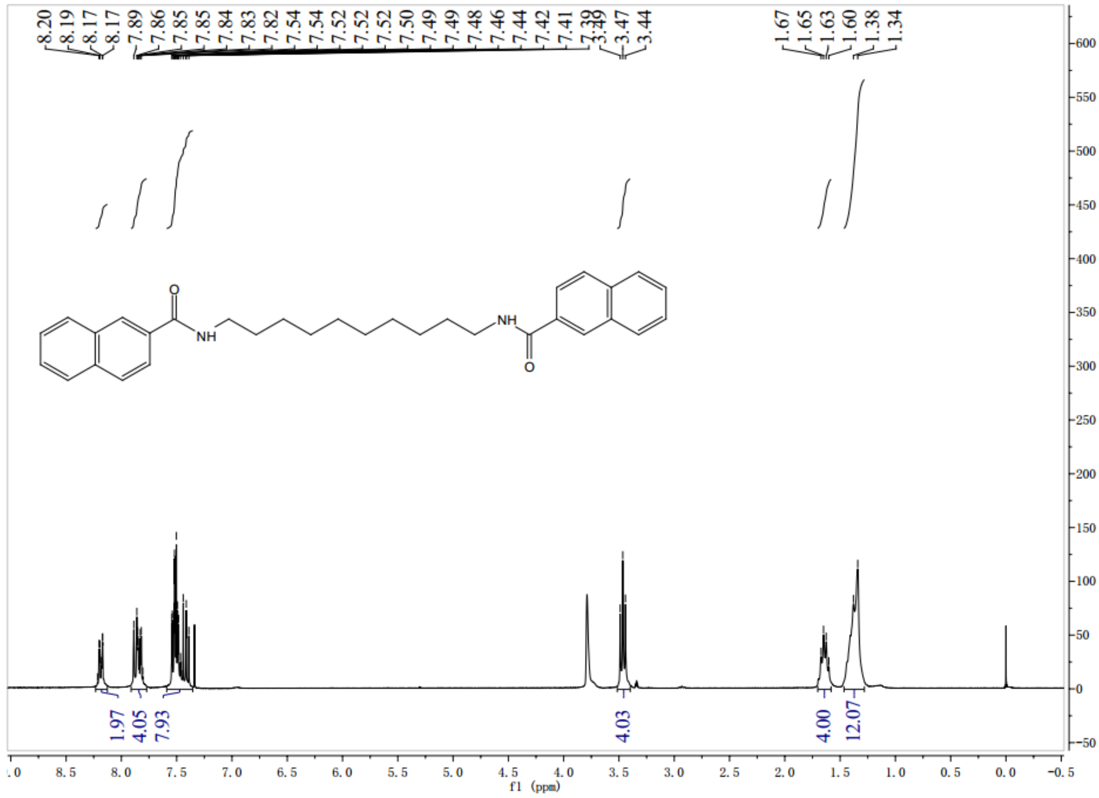

Figure S70 ^{1}H NMR spectrum of compound **12e.**

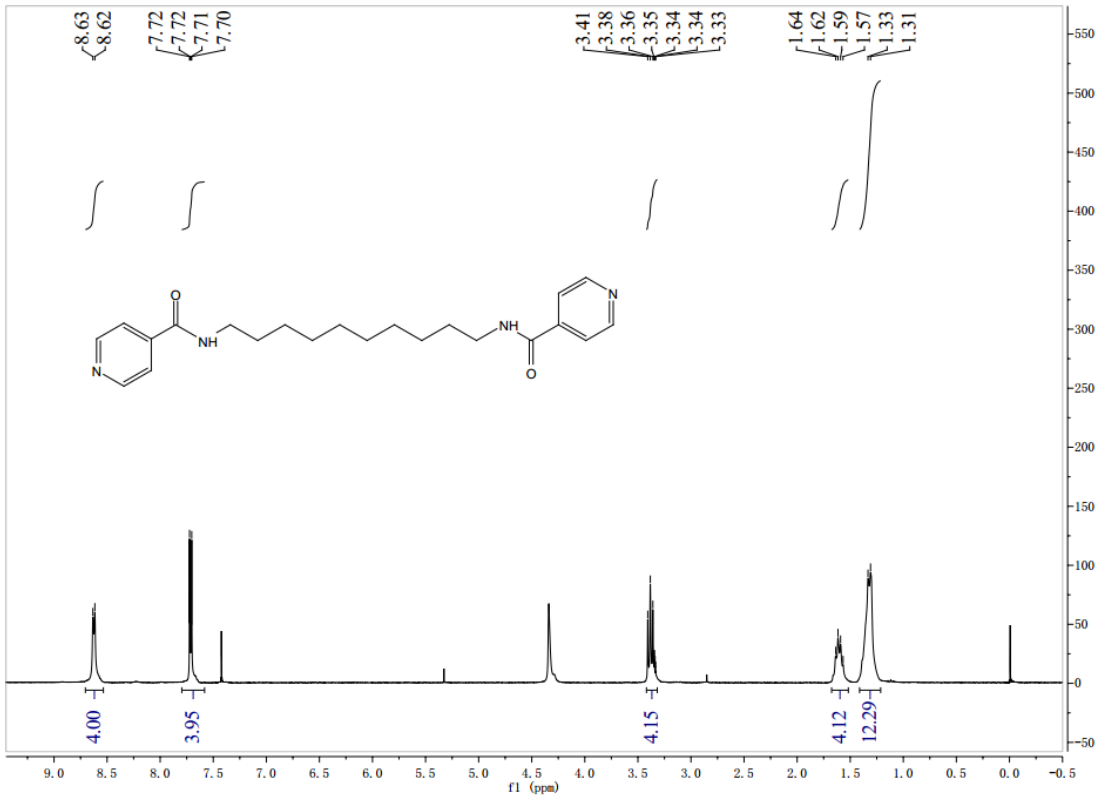

Figure S71 ^{1}H NMR spectrum of compound **13e.**

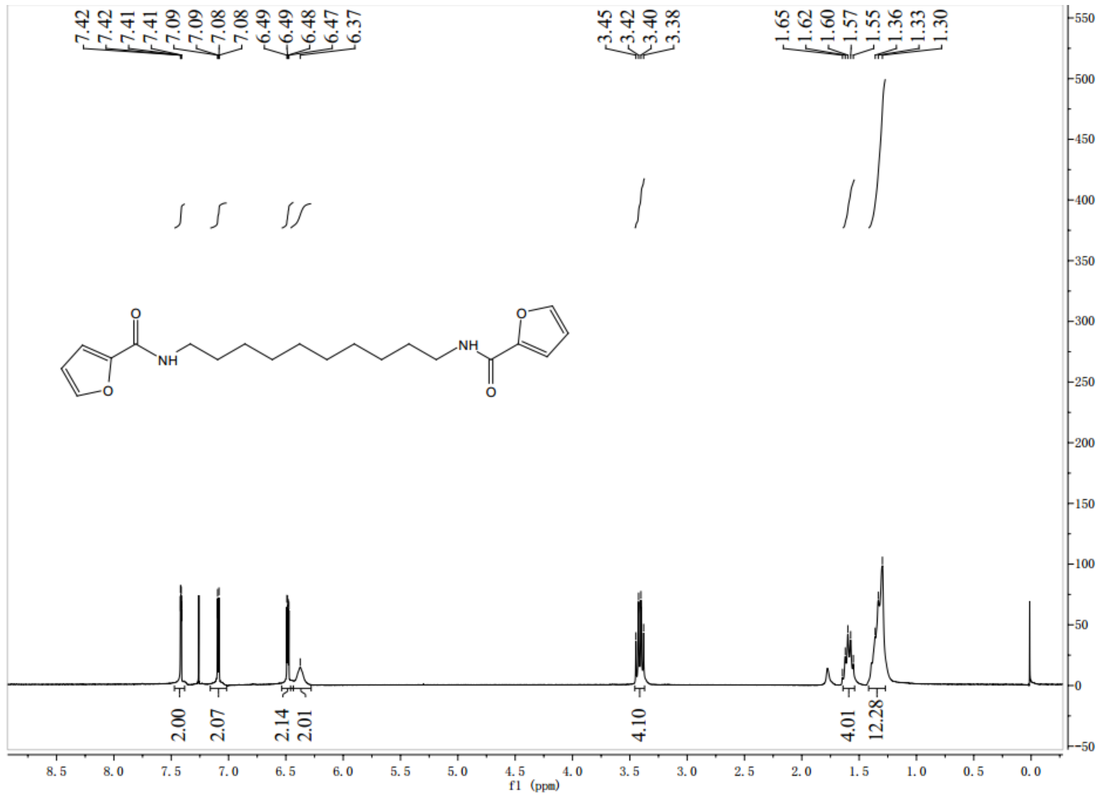

Figure S72 ^{1}H NMR spectrum of compound **14e.**

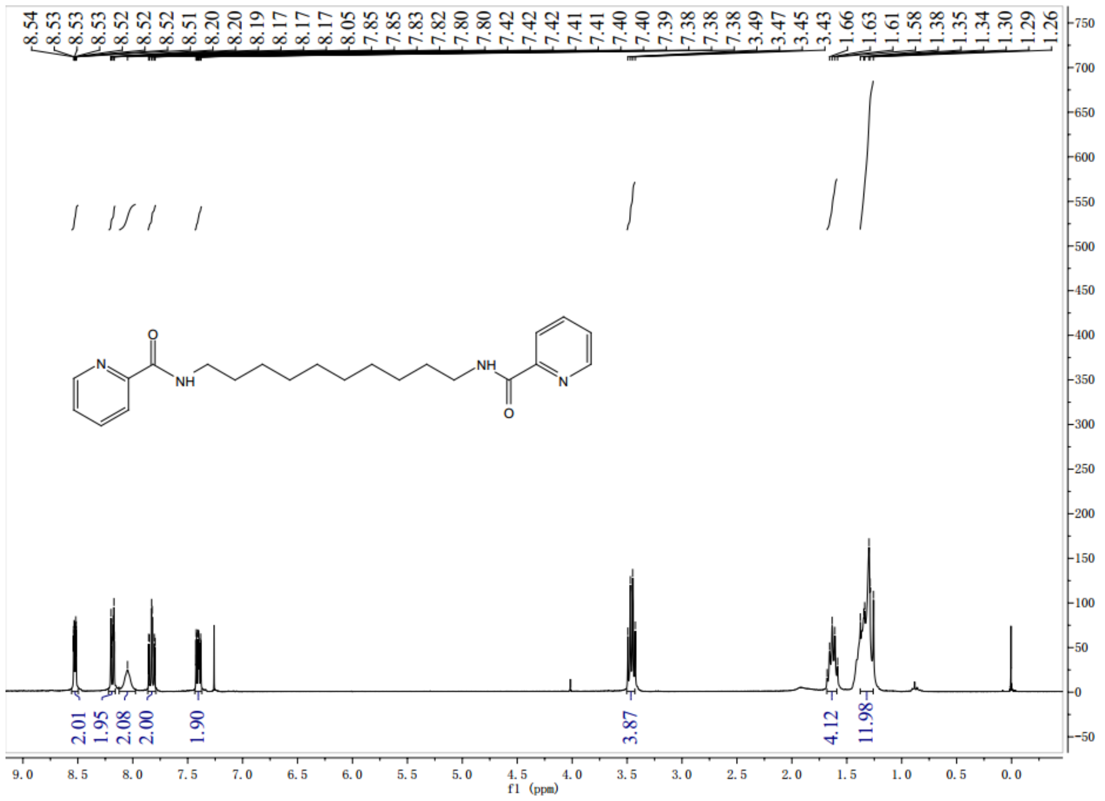

Figure S73 ^{1}H NMR spectrum of compound **15e.**

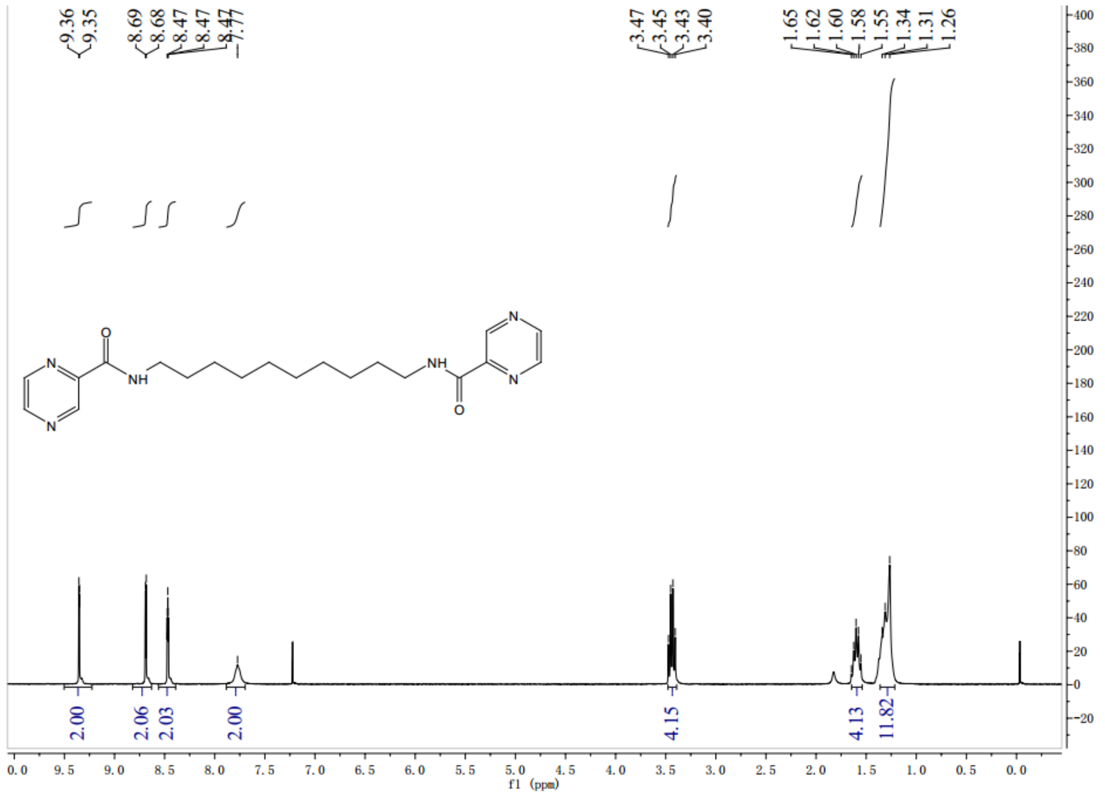

Figure S74 ^{1}H NMR spectrum of compound **16e.**

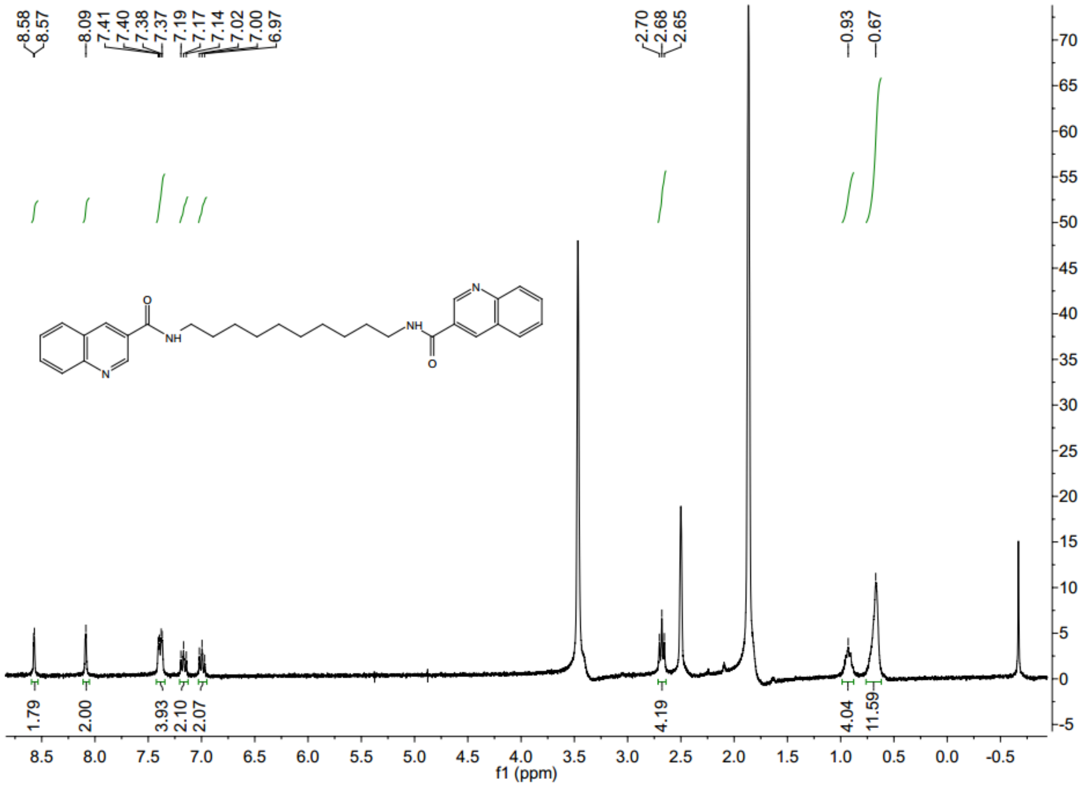

Figure S75 ^{1}H NMR spectrum of compound **17e.**

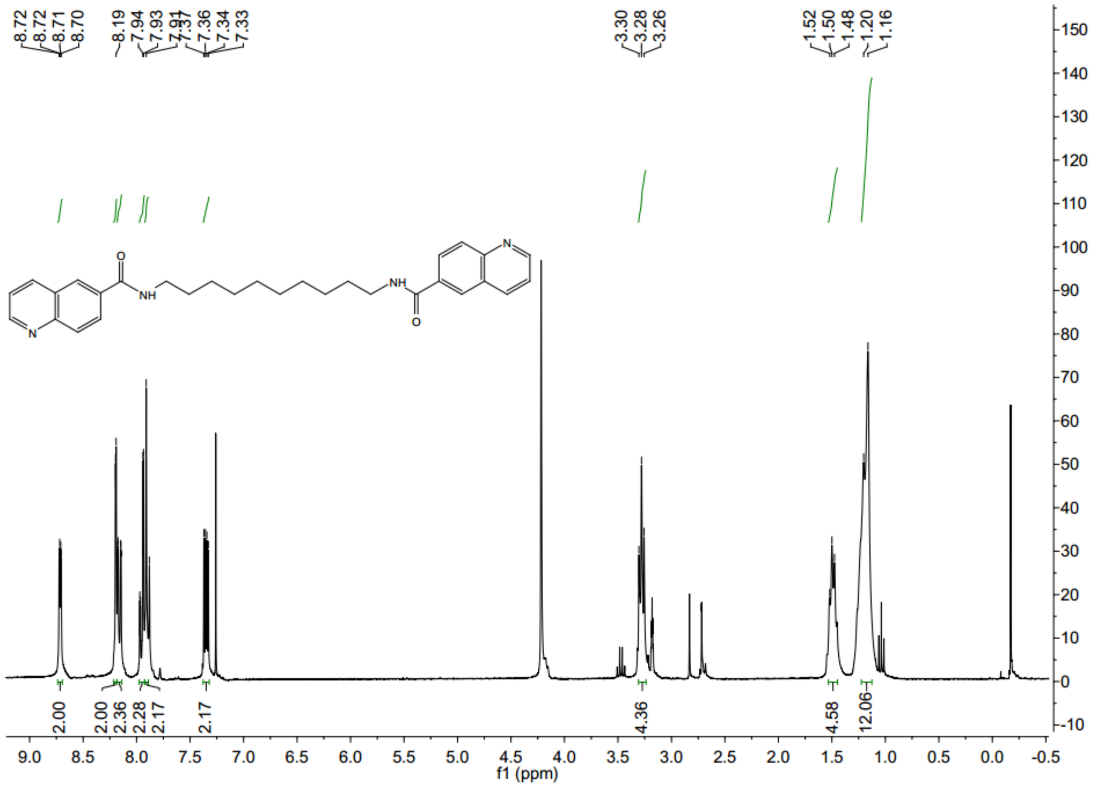

Figure S76 ^{1}H NMR spectrum of compound **18e.**

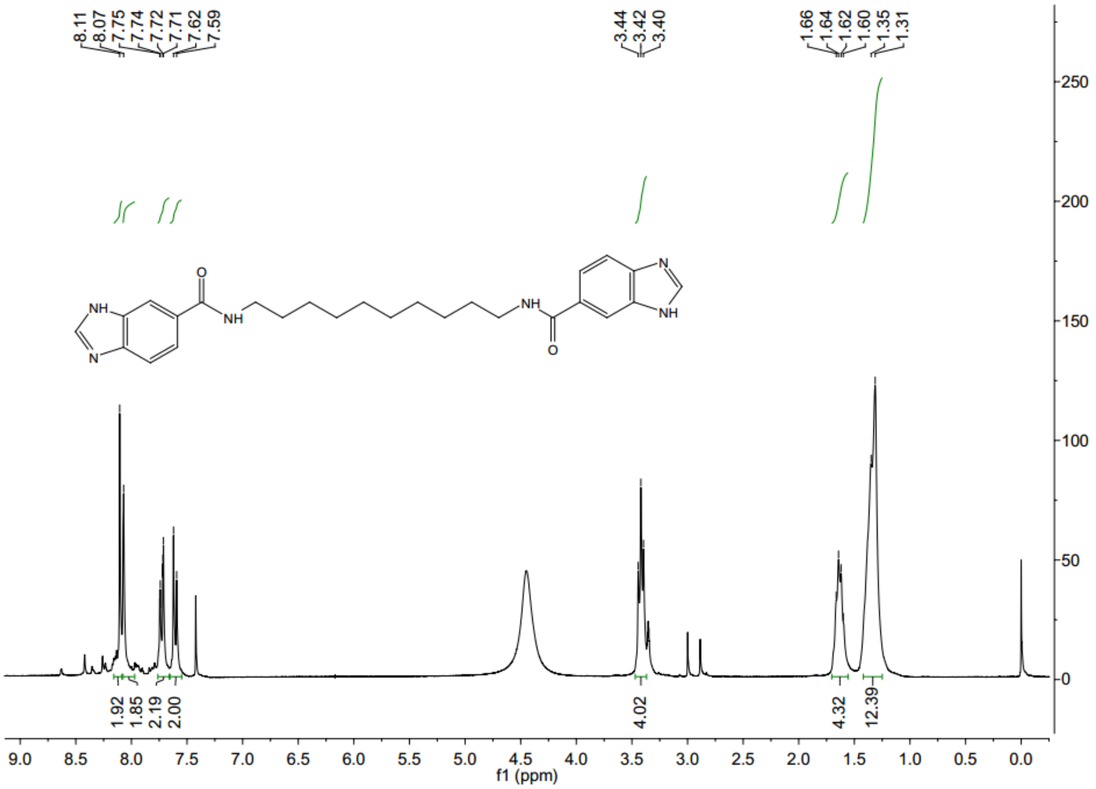

Figure S77 ^{1}H NMR spectrum of compound **19e.**

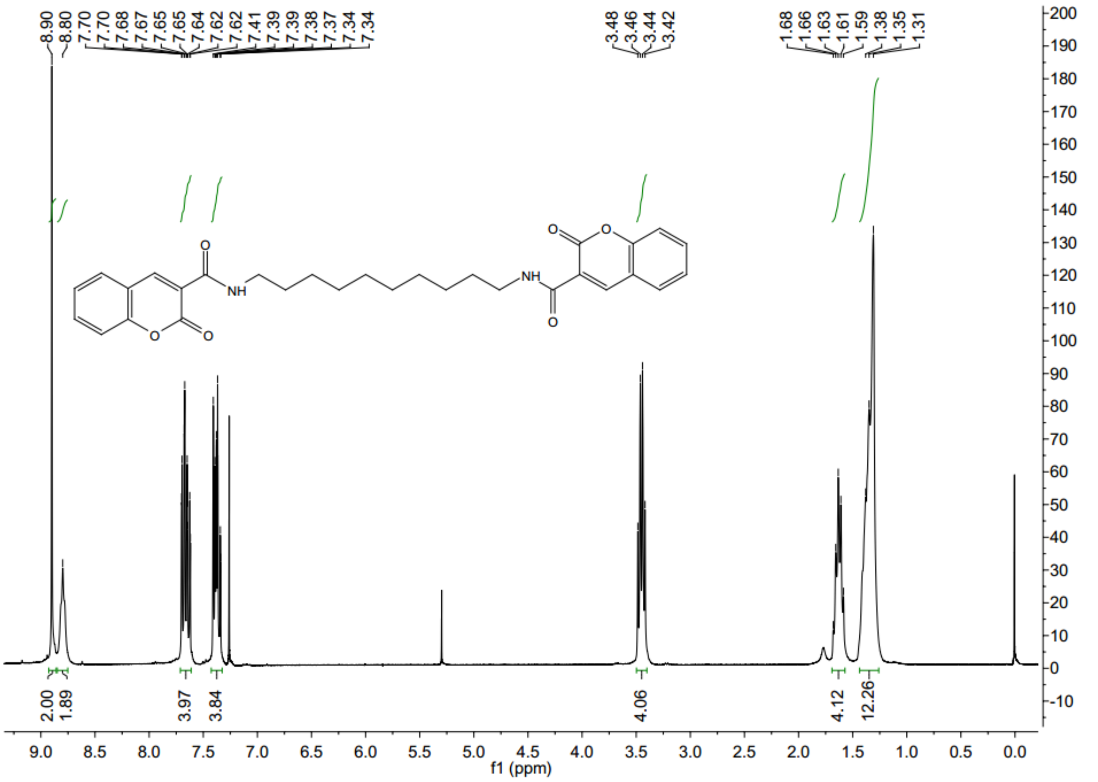

Figure S78 ^{1}H NMR spectrum of compound **20e.**

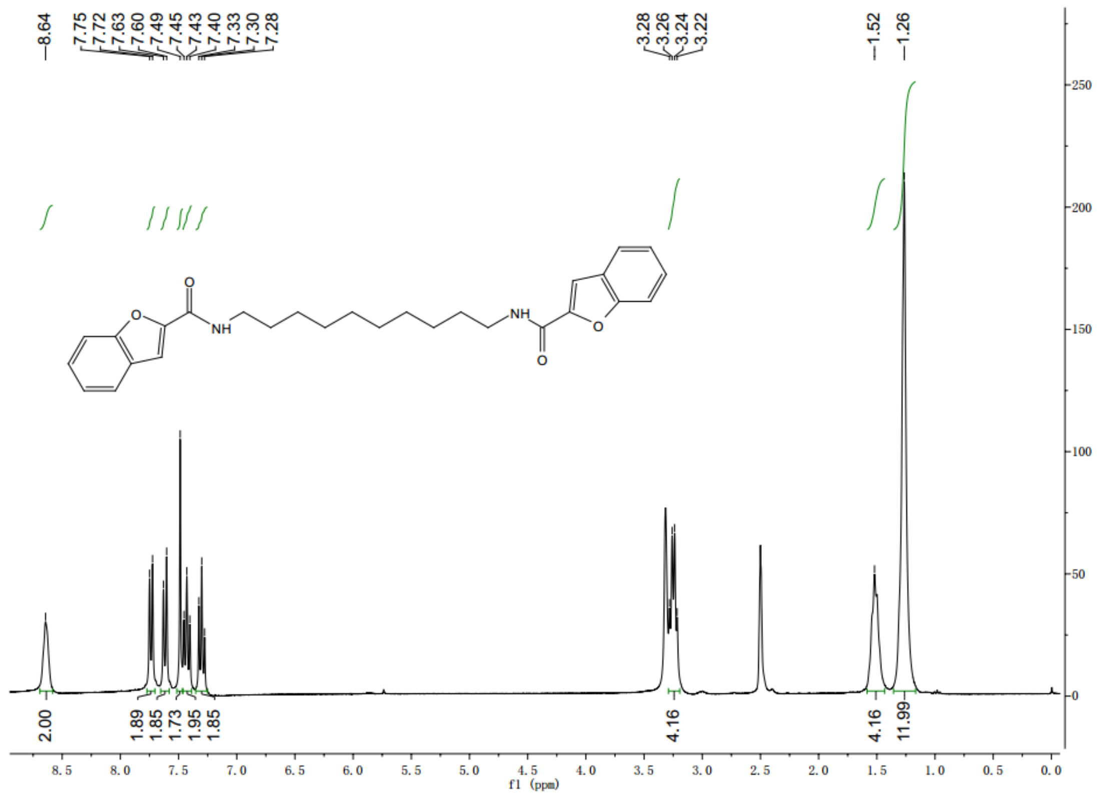

Figure S79 ^{1}H NMR spectrum of compound **21e.**

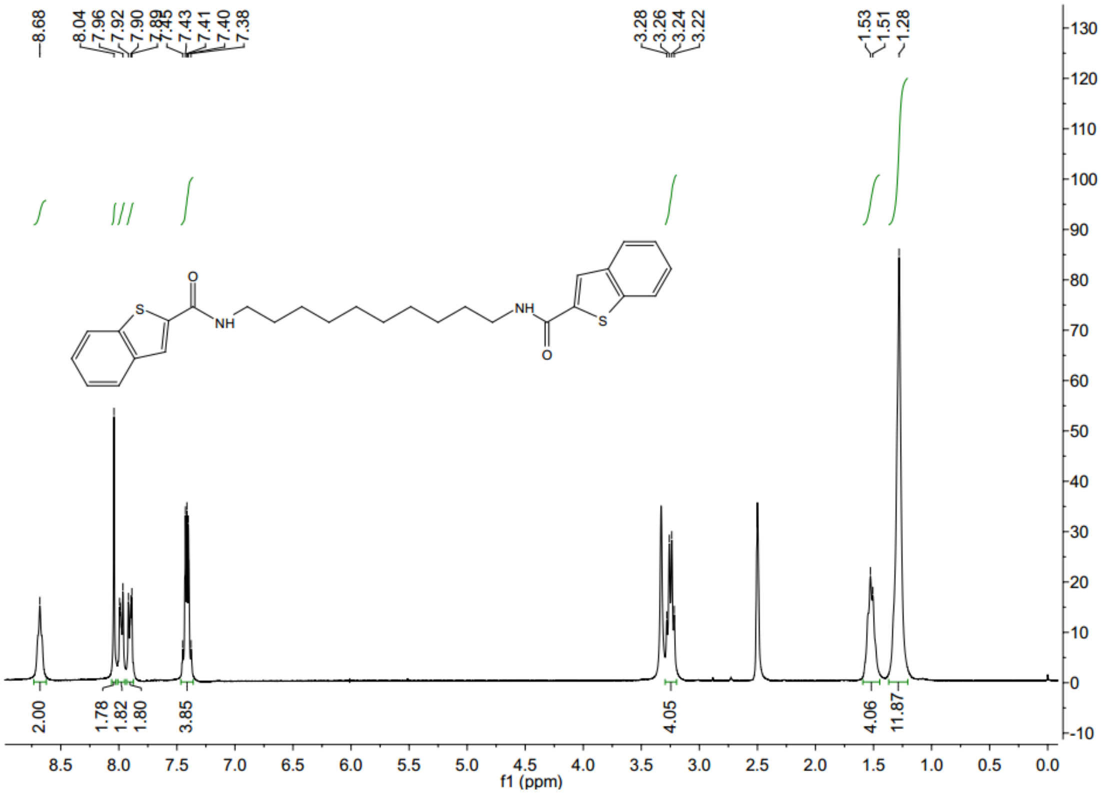

Figure S80 ^{1}H NMR spectrum of compound **22e.**

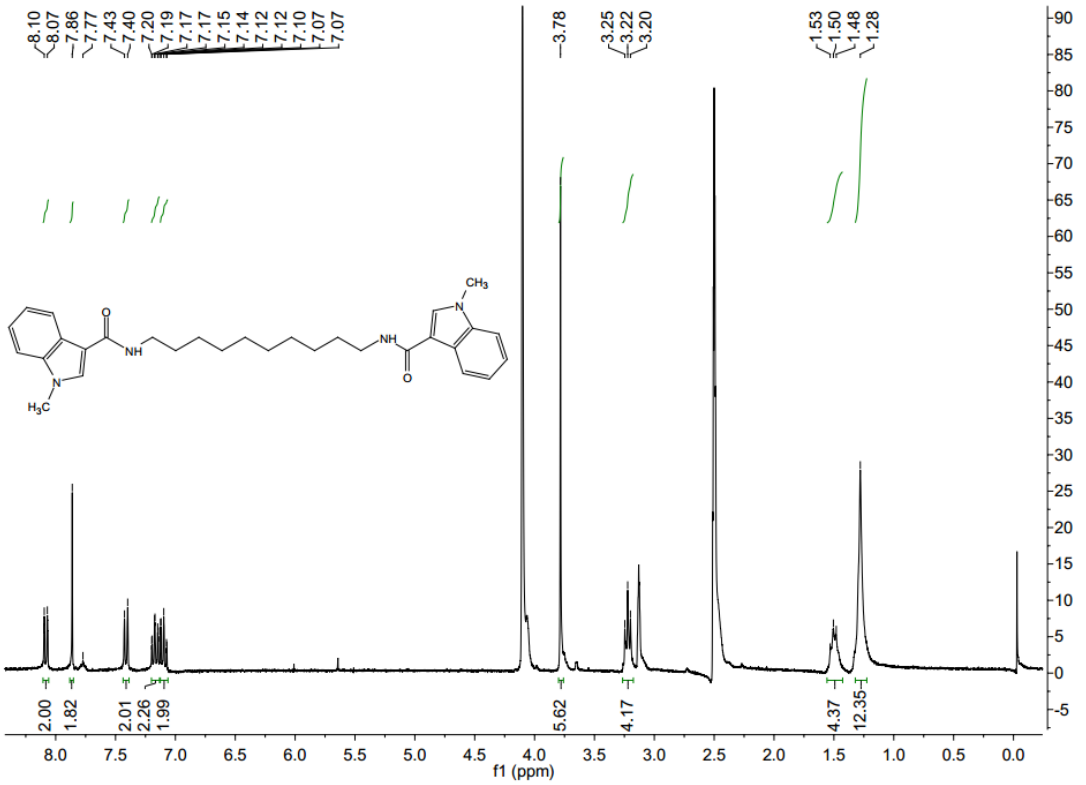

Figure S81 ^{1}H NMR spectrum of compound **23e.**

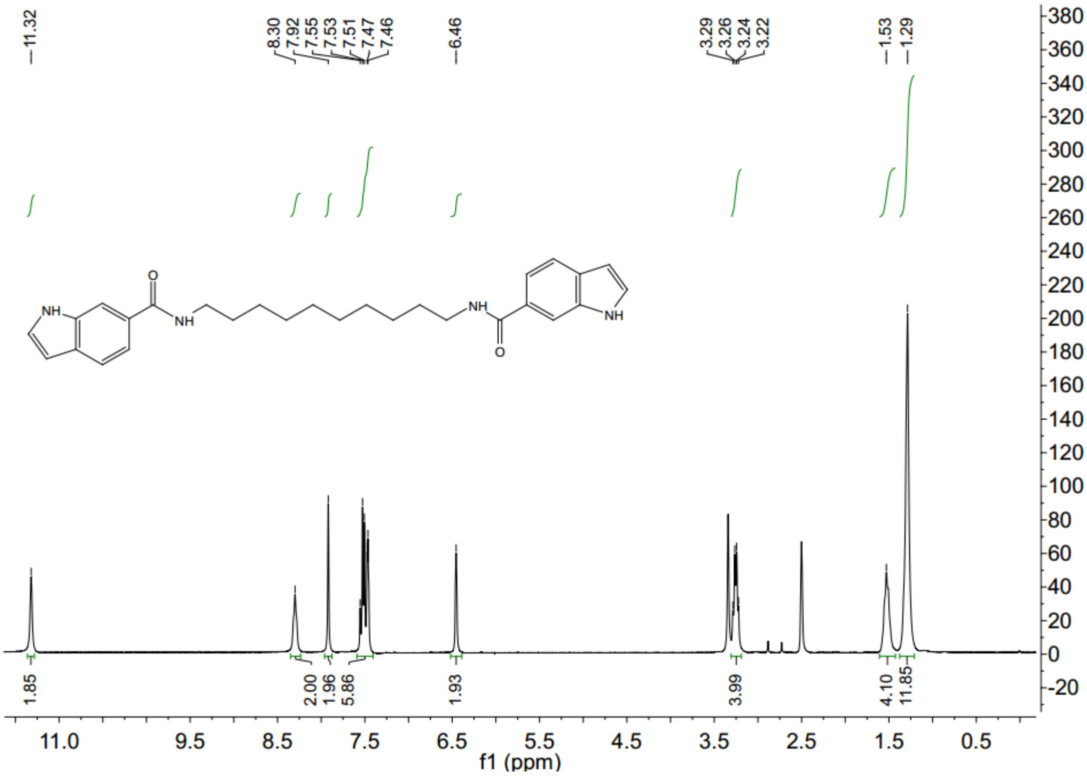

Figure S82 ^{1}H NMR spectrum of compound **24e.**

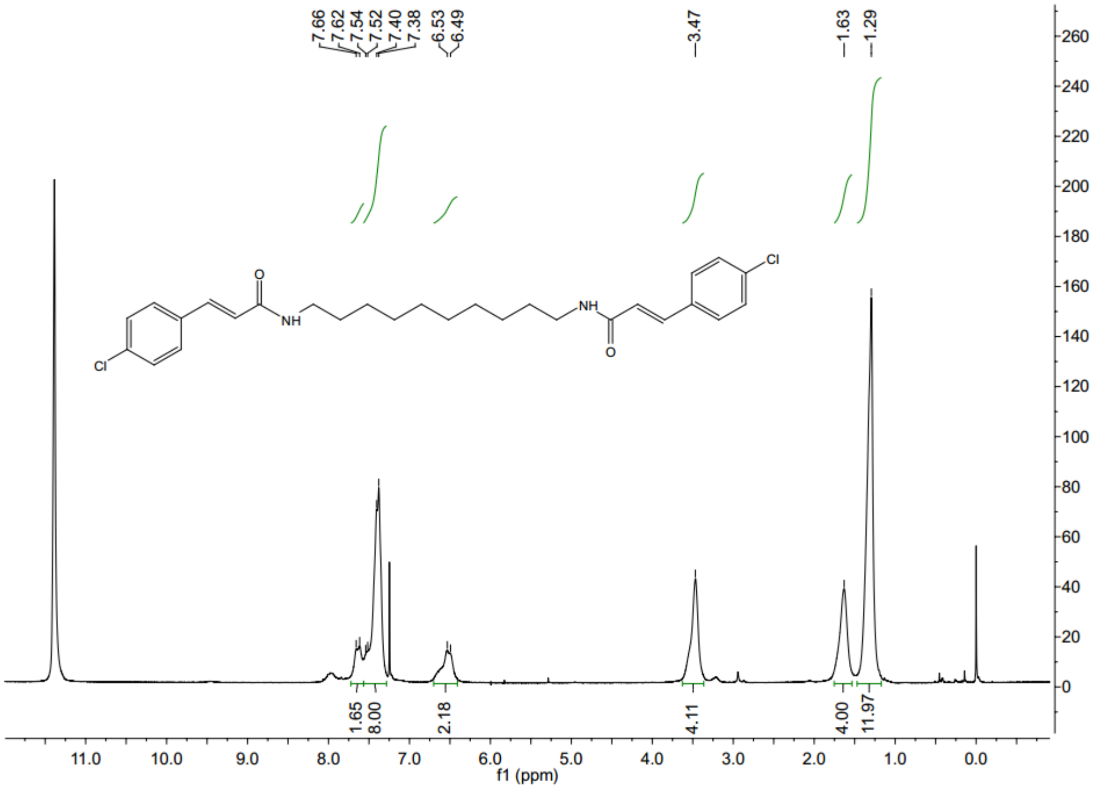

Figure S83 ^{1}H NMR spectrum of compound **25e.**

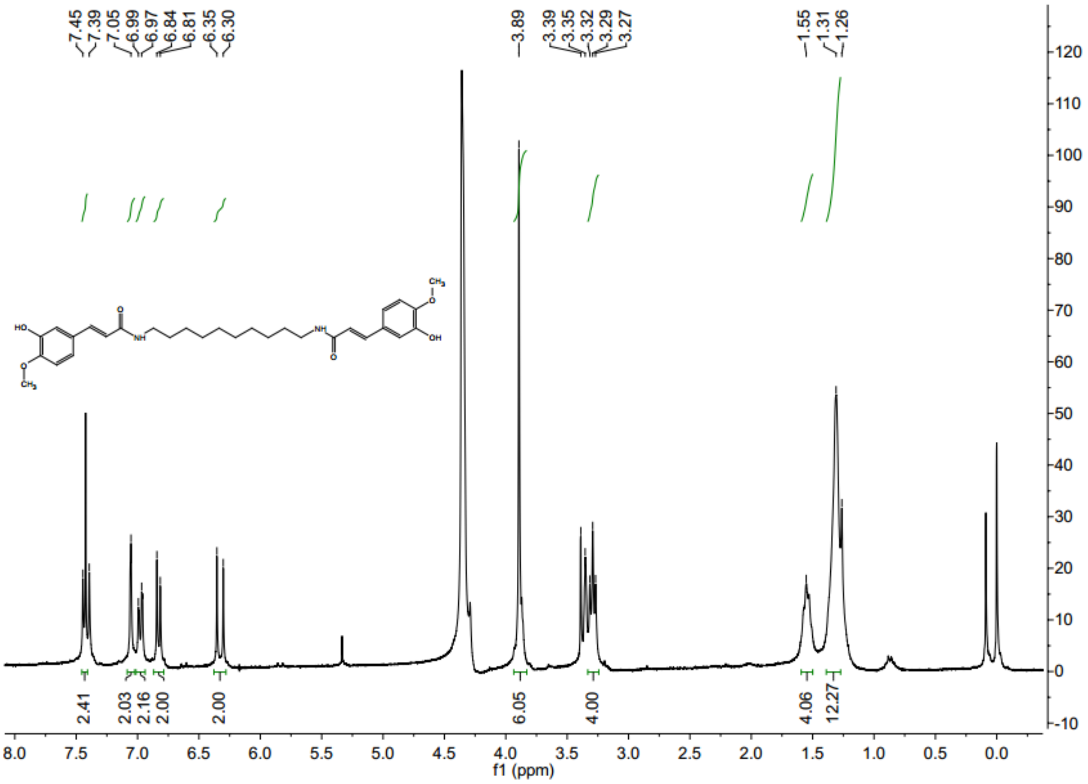

Figure S84 ^{1}H NMR spectrum of compound **26e.**

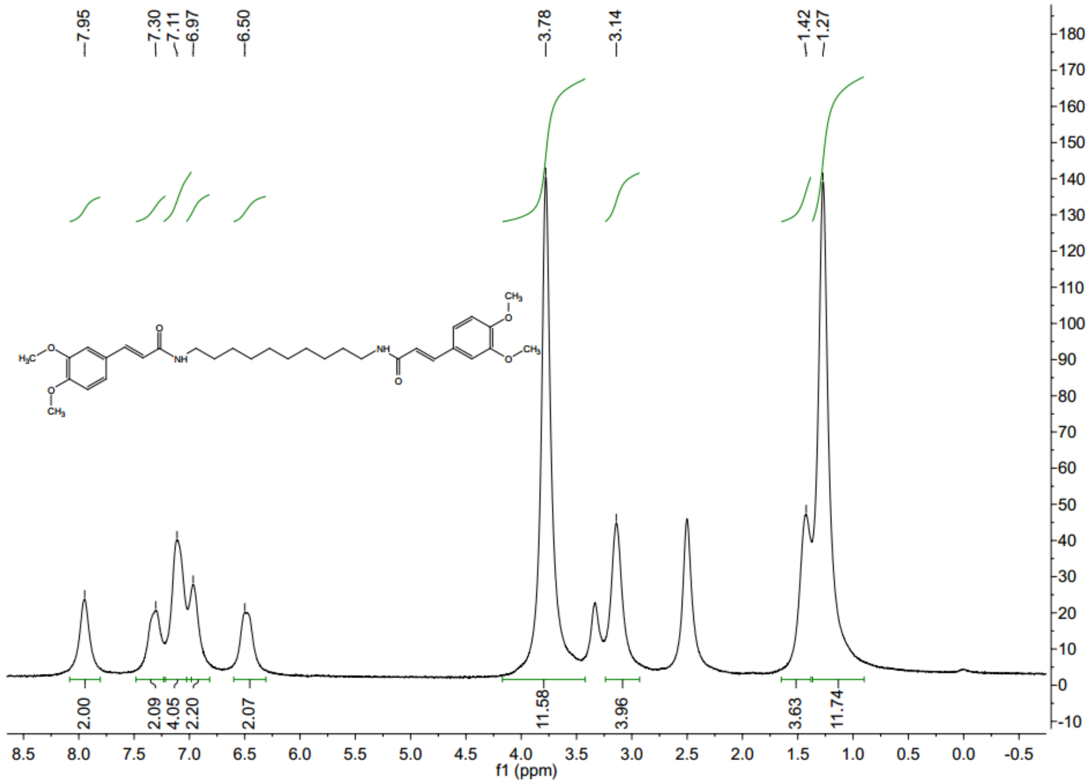

Figure S85 ^{1}H NMR spectrum of compound **27e.**

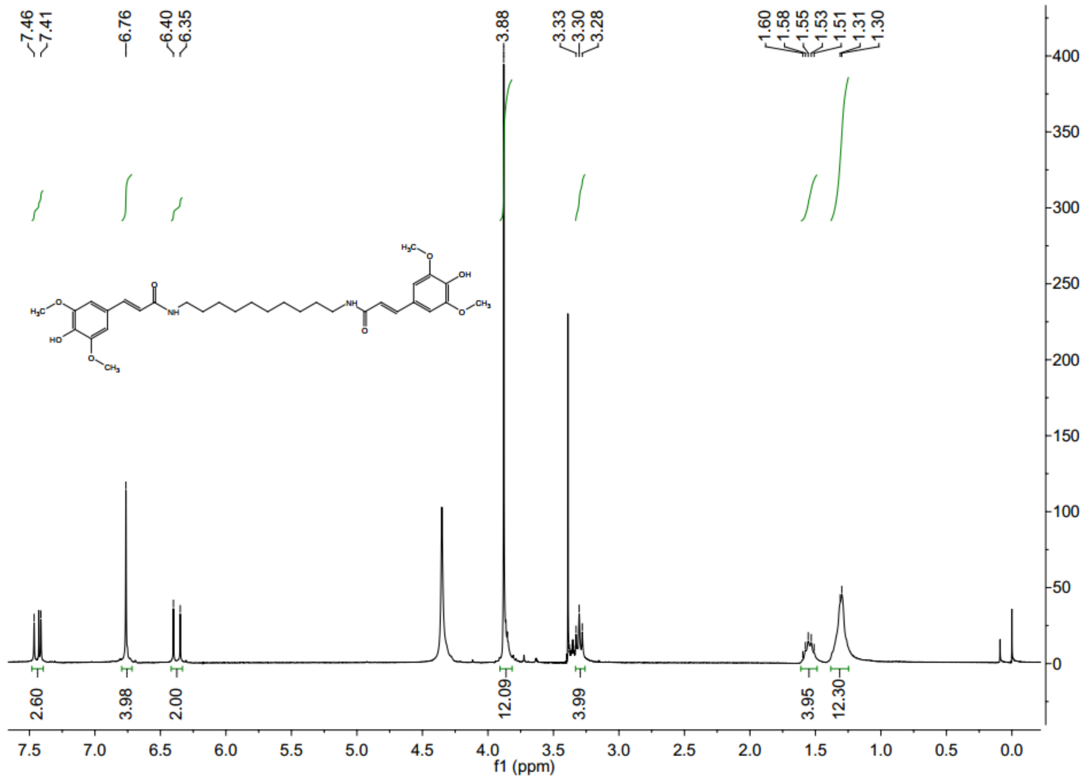

Figure S86 ^{1}H NMR spectrum of compound **28e.**

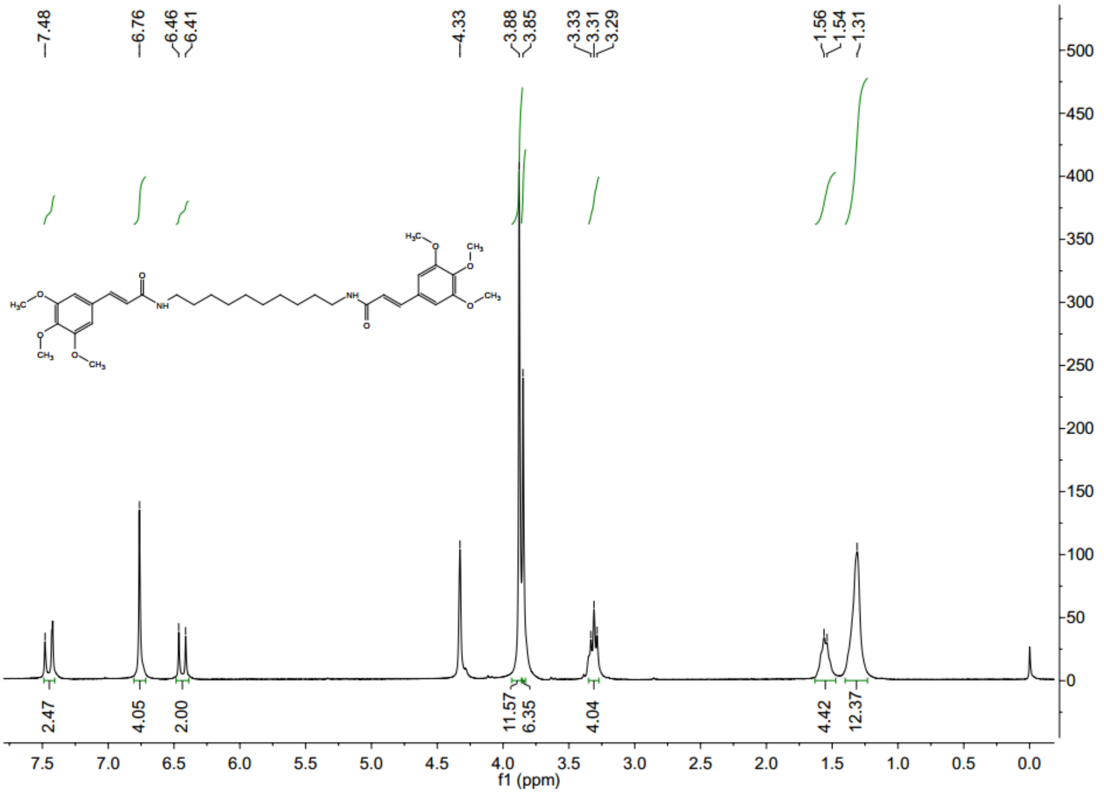

Figure S87 ^{1}H NMR spectrum of compound **29e.**

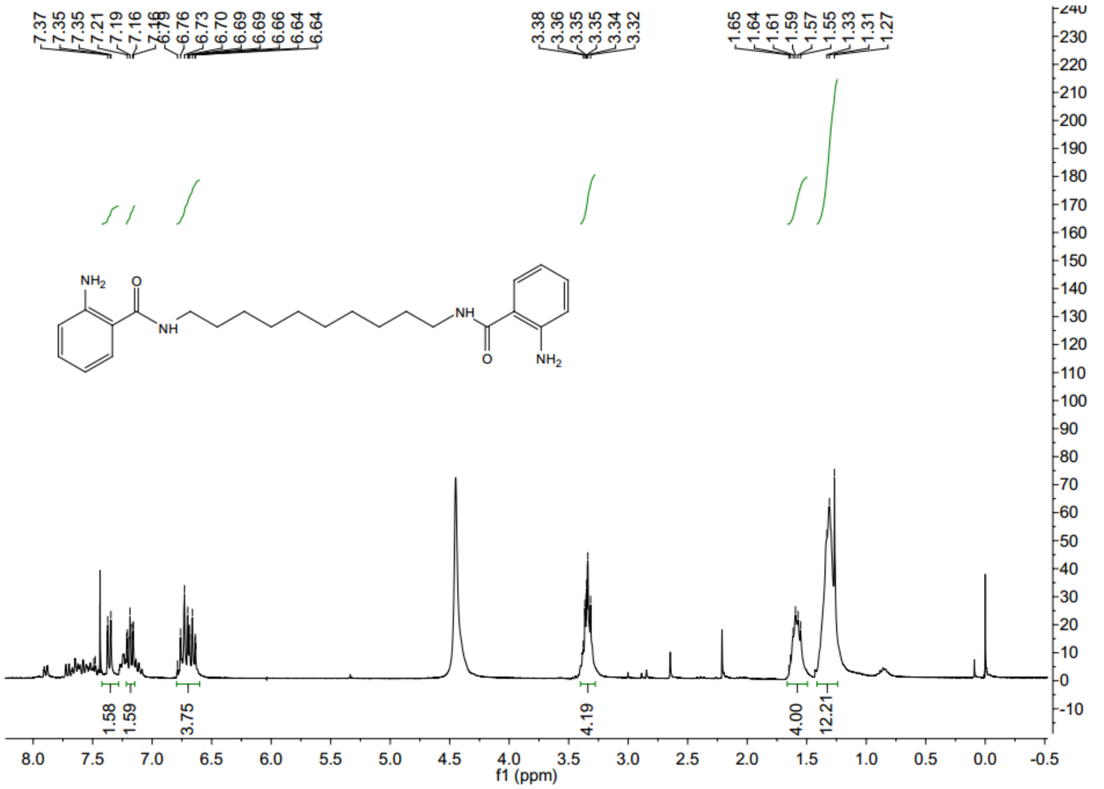

Figure S88 ^{1}H NMR spectrum of compound **30e.**

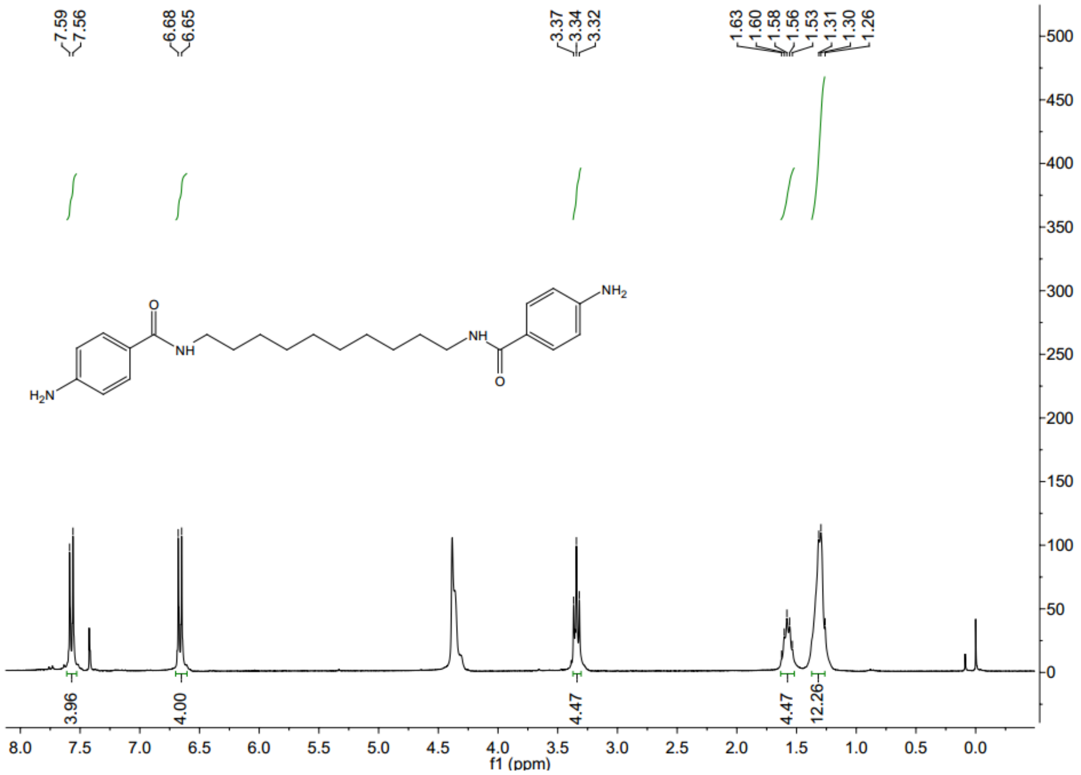

Figure S89 ^{1}H NMR spectrum of compound **31e.**

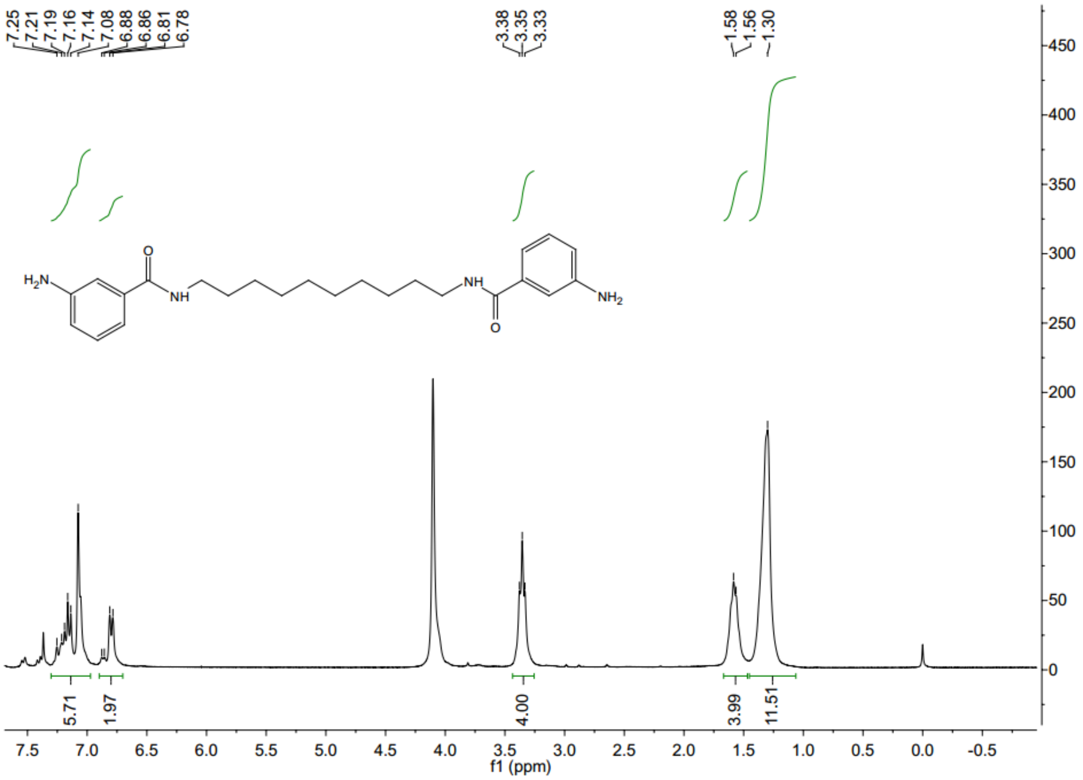

Figure S90 ^{1}H NMR spectrum of compound **32e.**

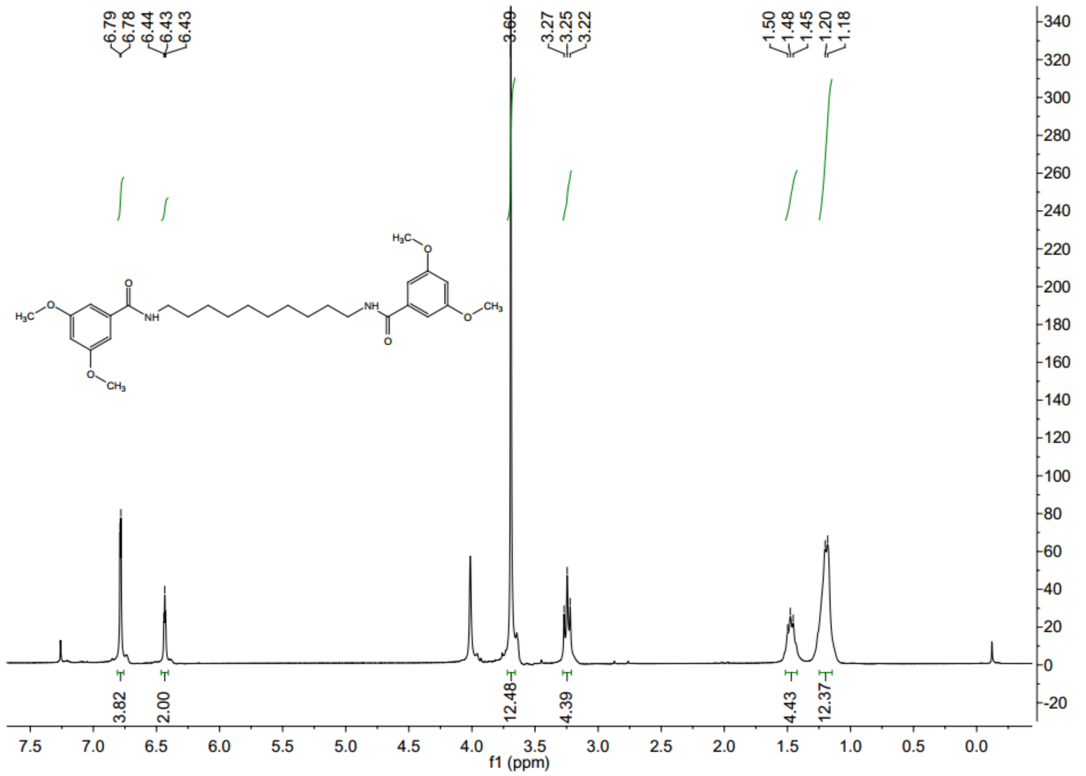

Figure S91 ^{1}H NMR spectrum of compound **33e.**

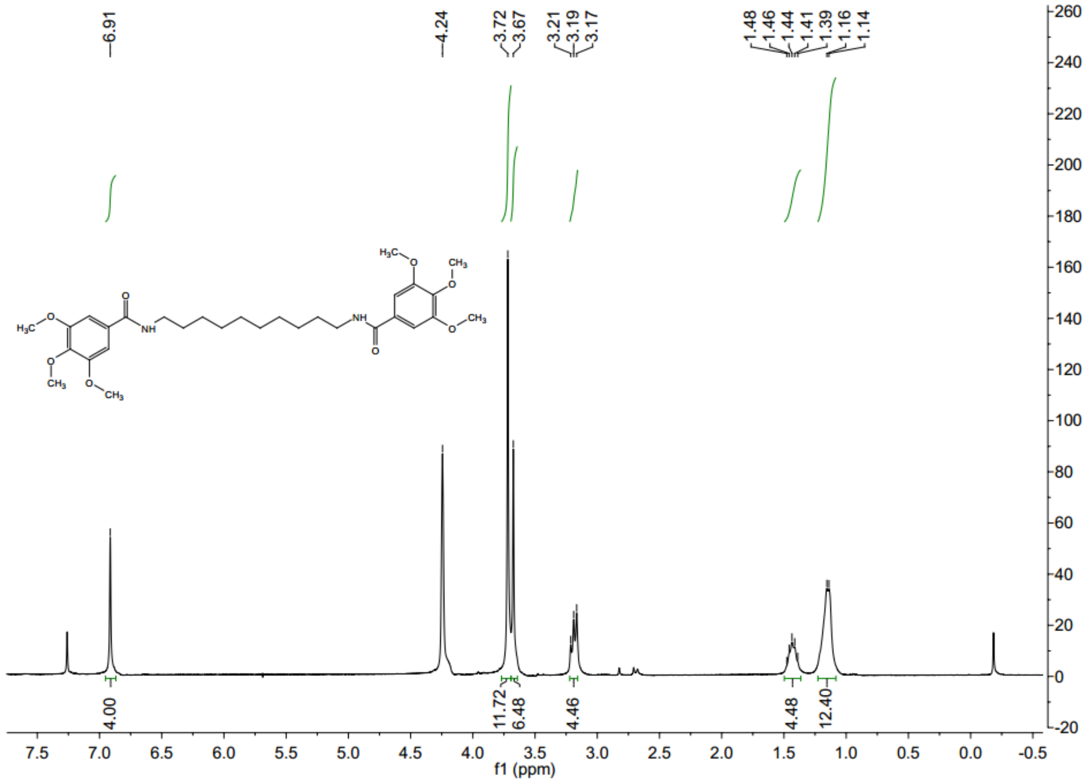

Figure S92 ^{1}H NMR spectrum of compound **34e.**

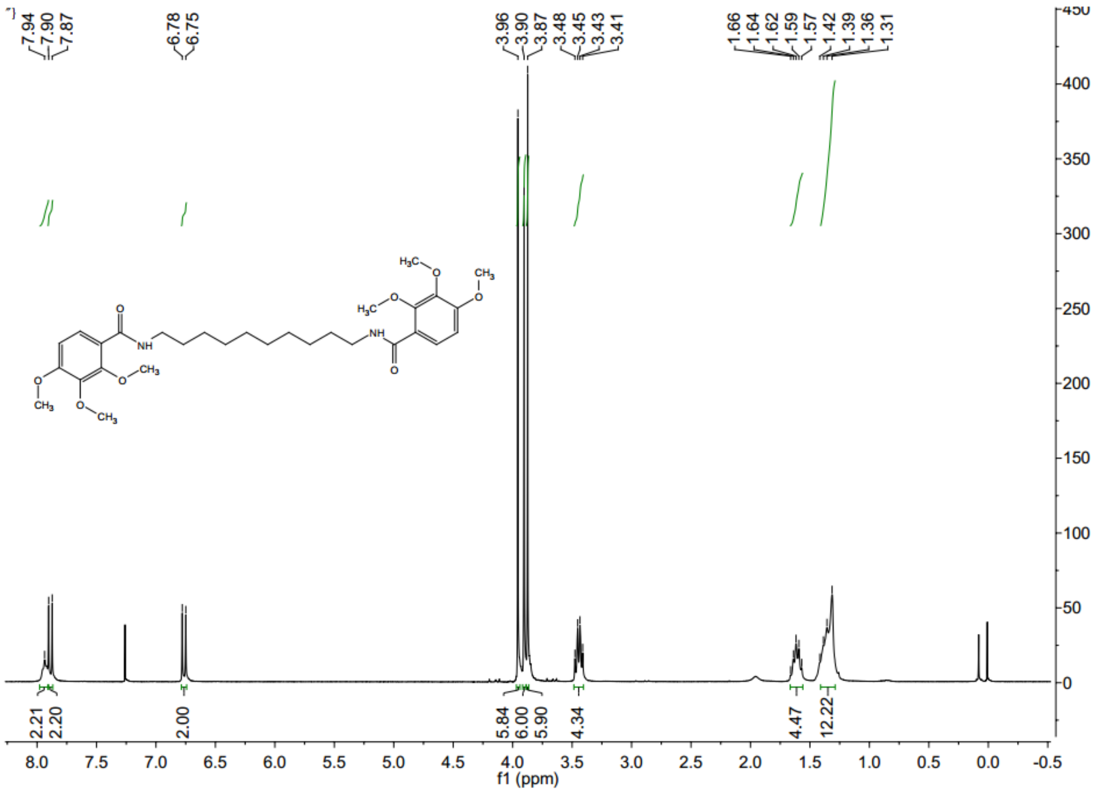

Figure S93 ^{1}H NMR spectrum of compound **35e.**

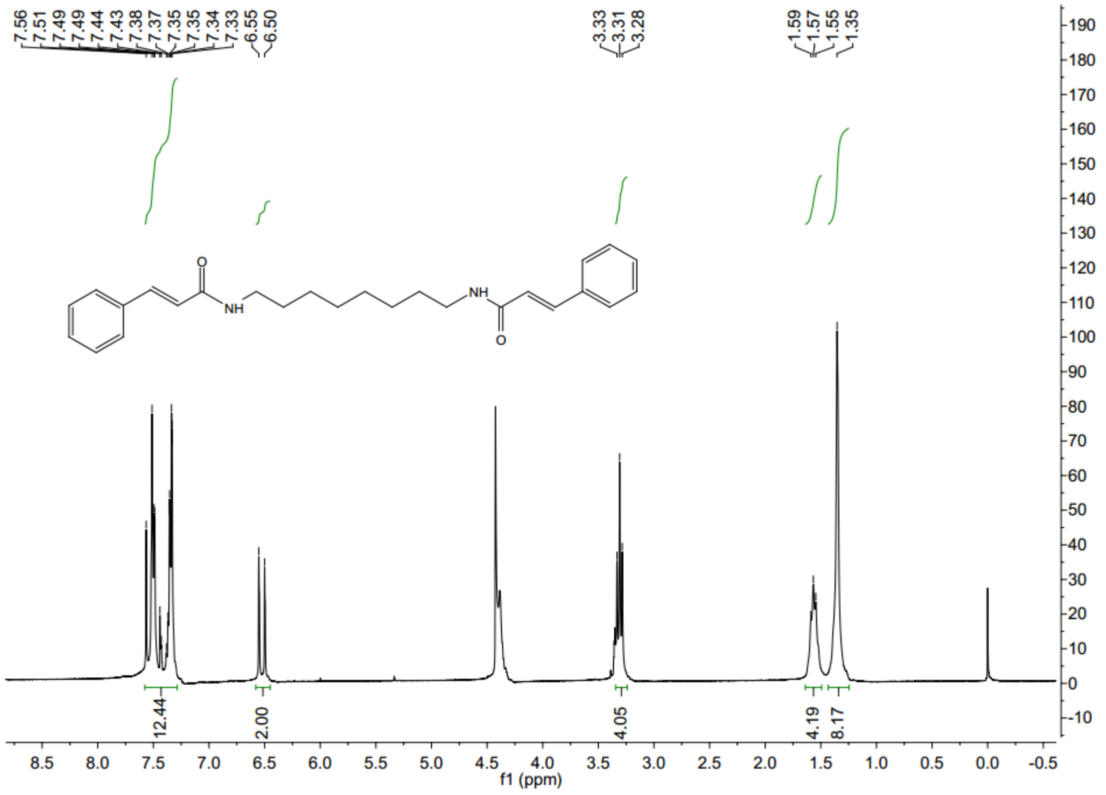

Figure S94 ^{1}H NMR spectrum of compound **9d.**

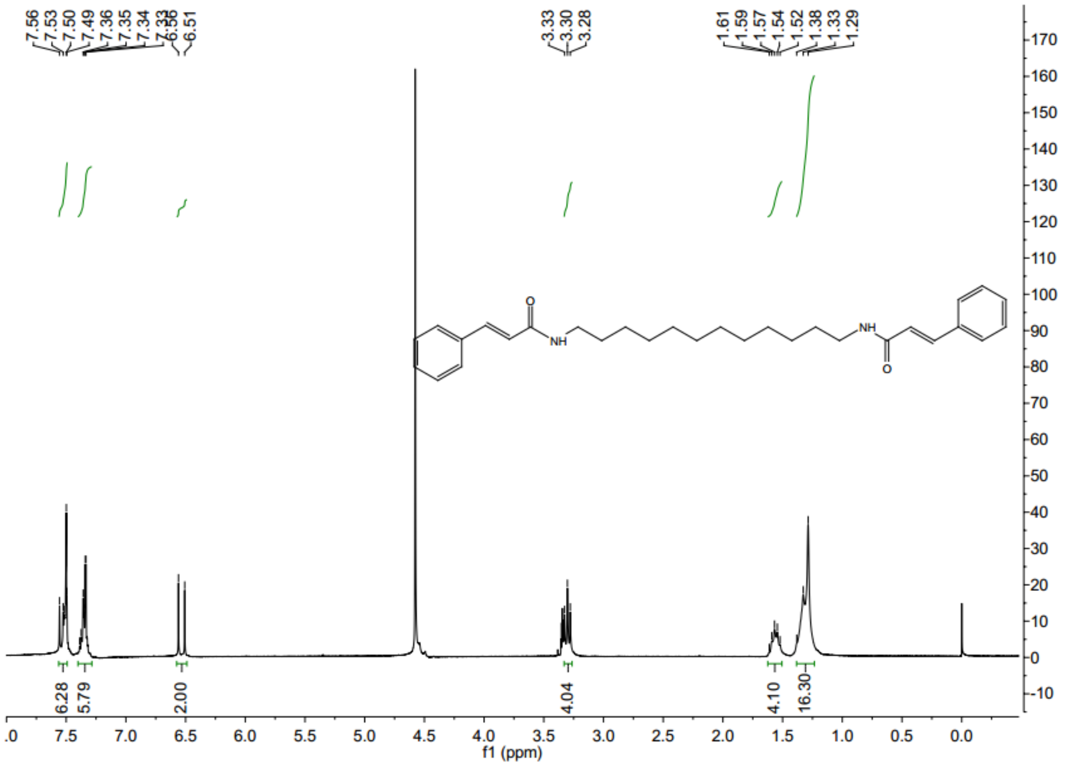

Figure S95 ^{1}H NMR spectrum of compound **9f.**

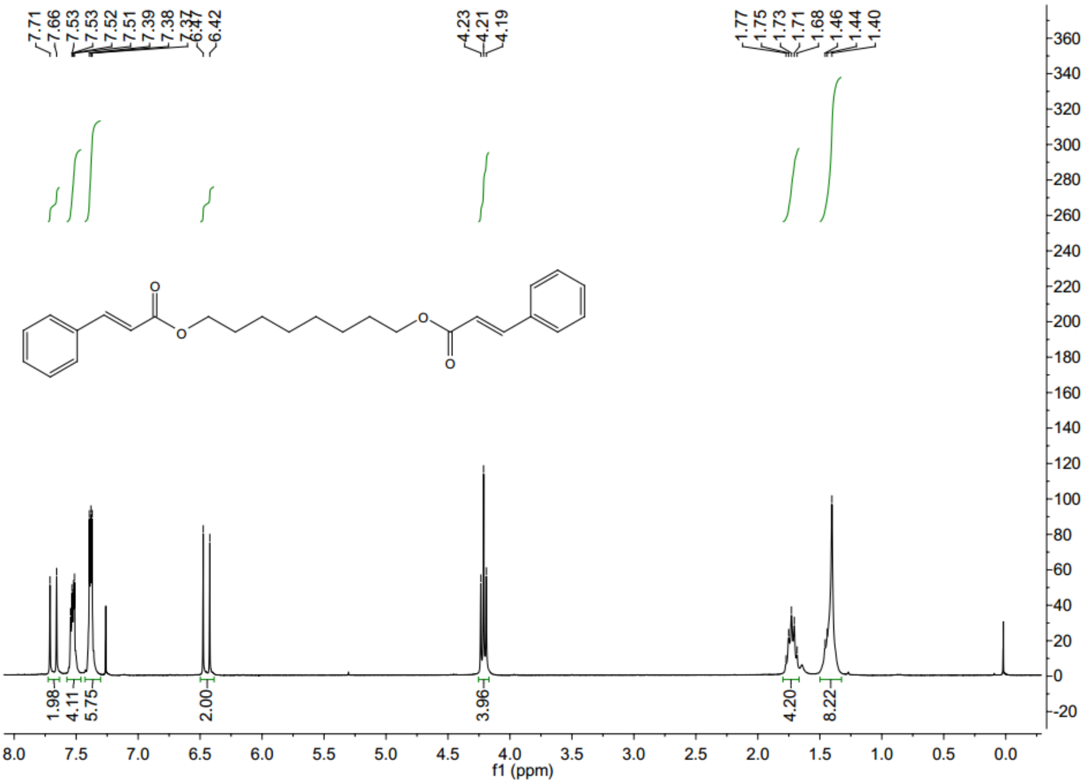

Figure S96 ^{1}H NMR spectrum of compound **9g.**

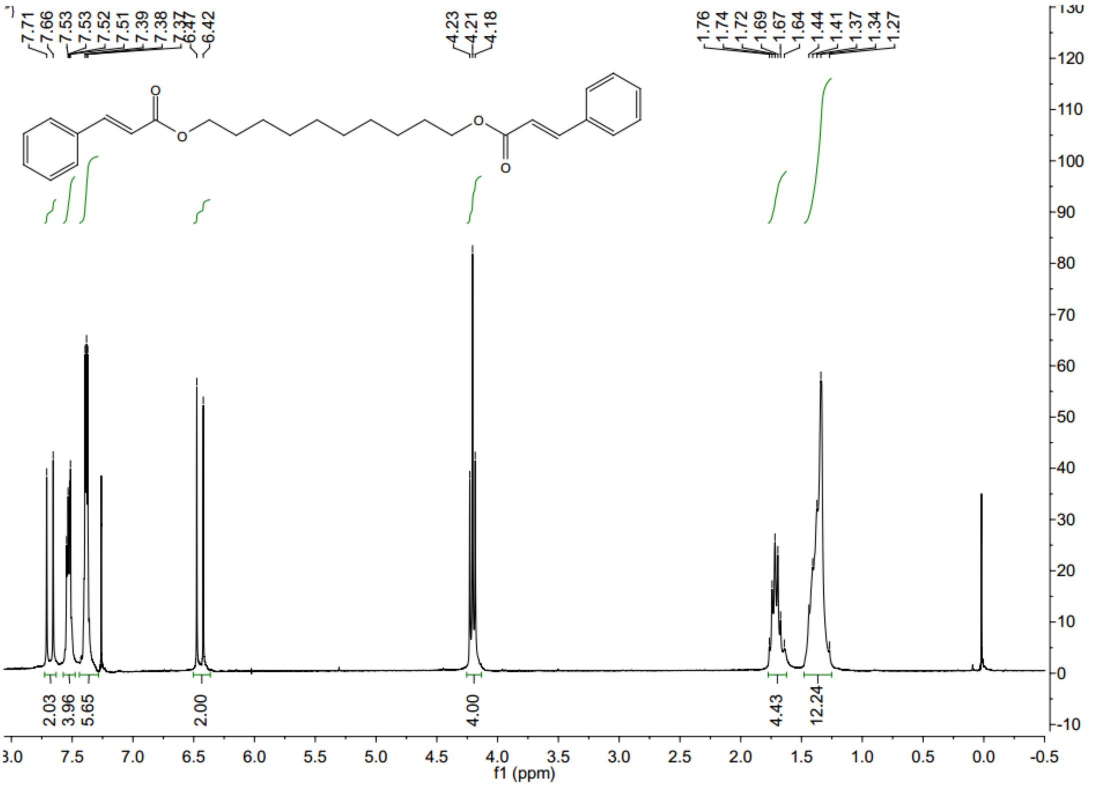

Figure S97 ^{1}H NMR spectrum of compound **9h.**

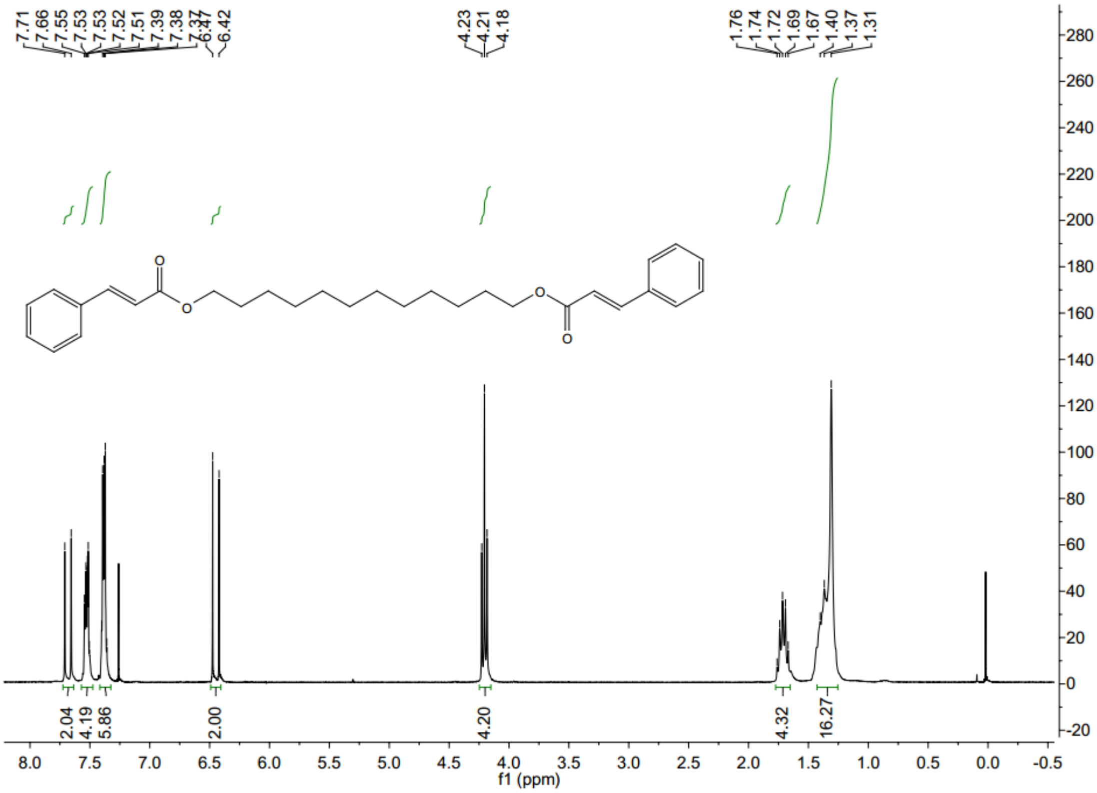

Figure S98 ^{1}H NMR spectrum of compound **9i.**

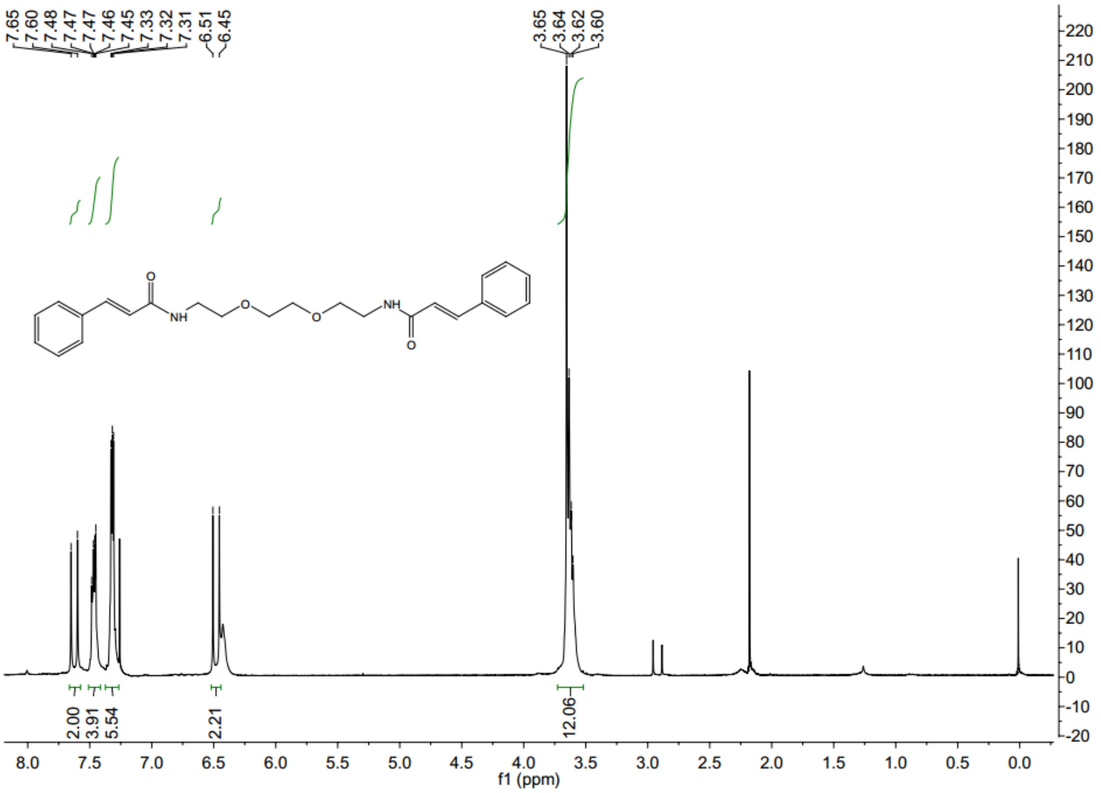

Figure S99 ^{1}H NMR spectrum of compound **9j.**

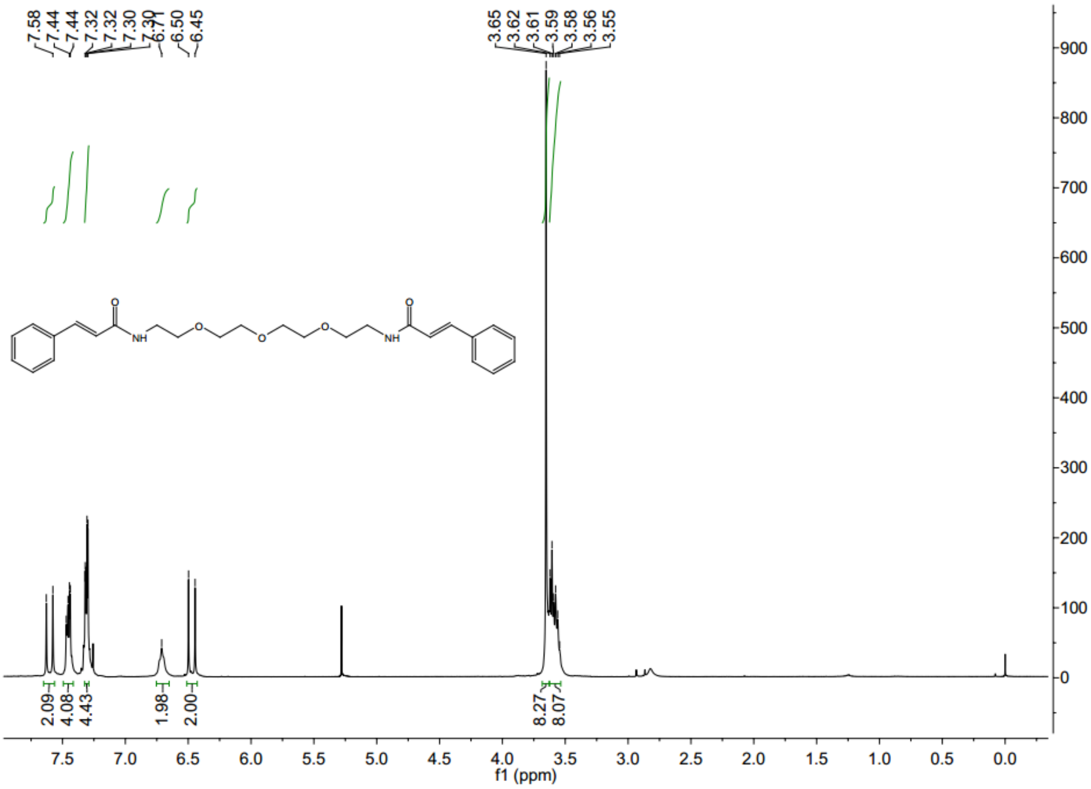

Figure S100 ^{1}H NMR spectrum of compound **9k.**

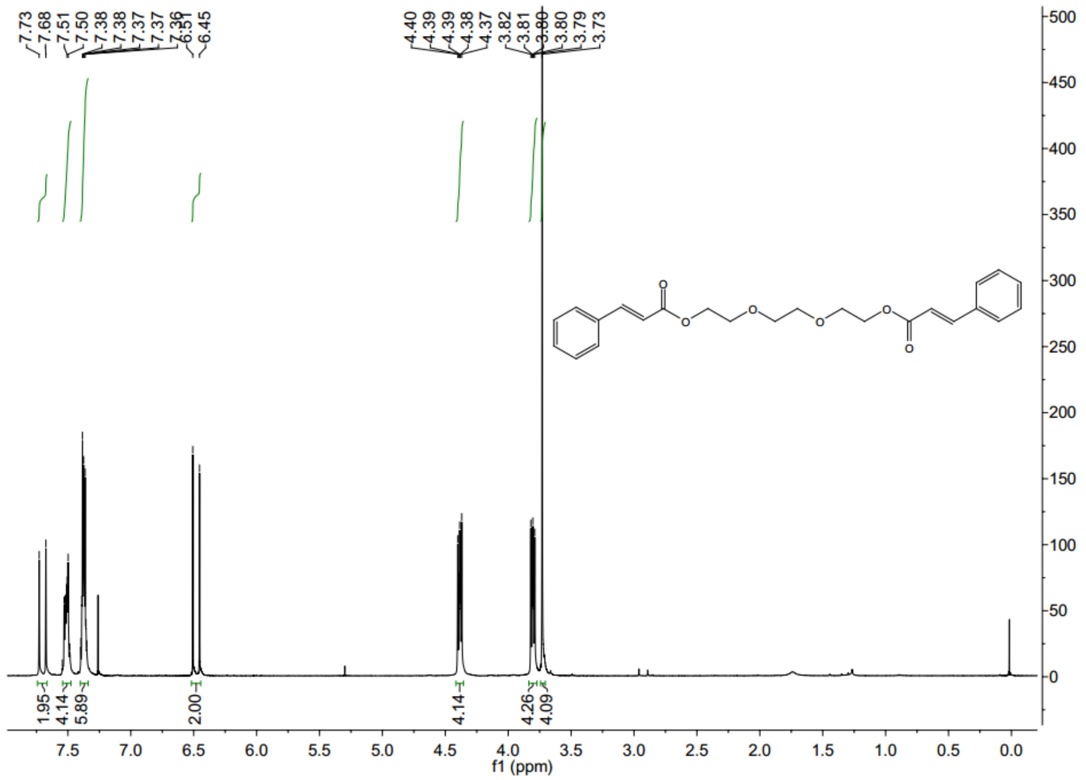

Figure S101 ^{1}H NMR spectrum of compound **9l.**

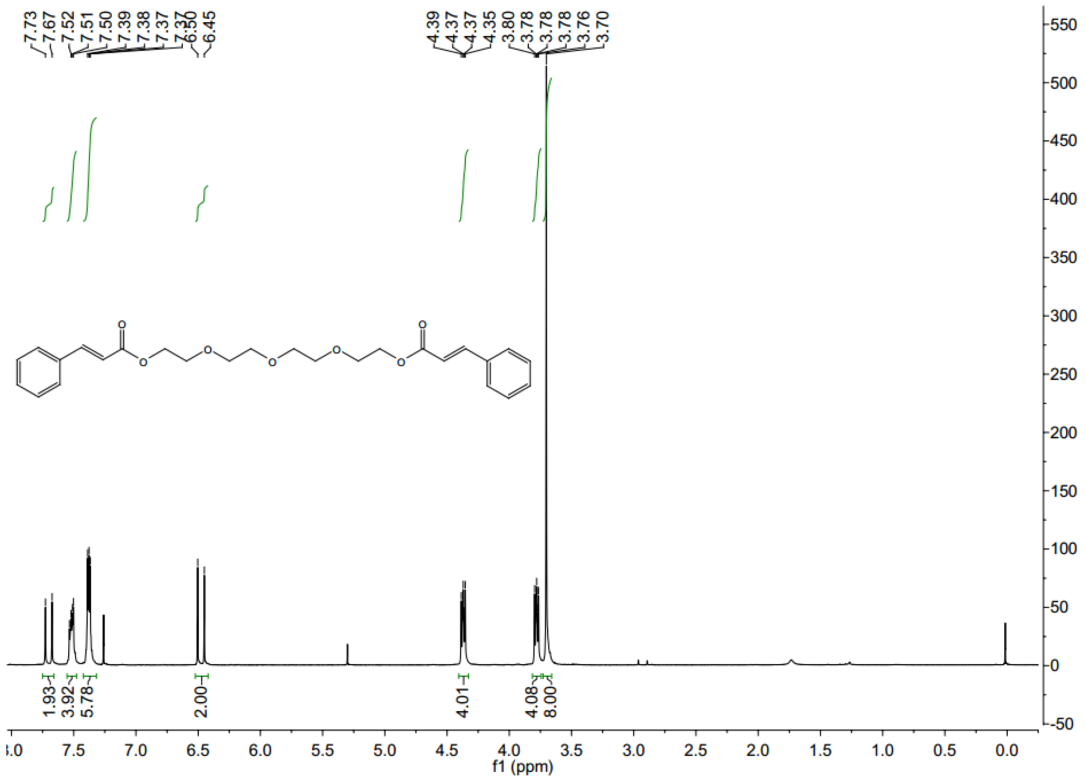

Figure S102 ^{1}H NMR spectrum of compound **9m.**

13C-NMR

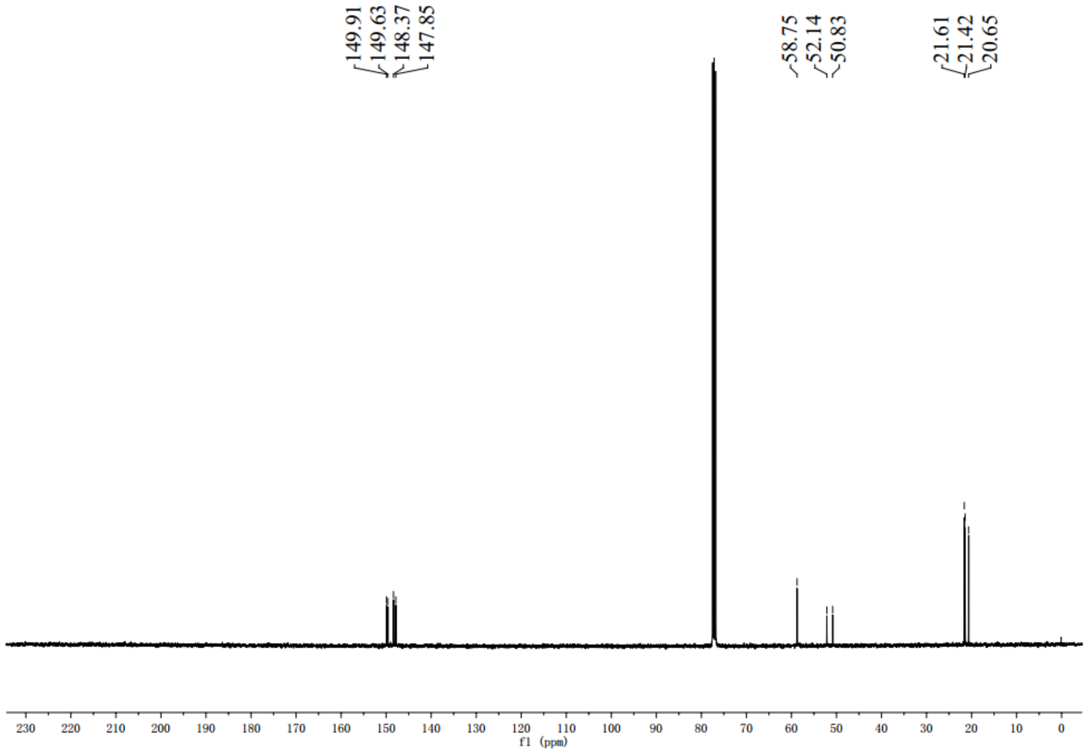

Figure S103 ^{13}C NMR spectrum of compound **6a.**

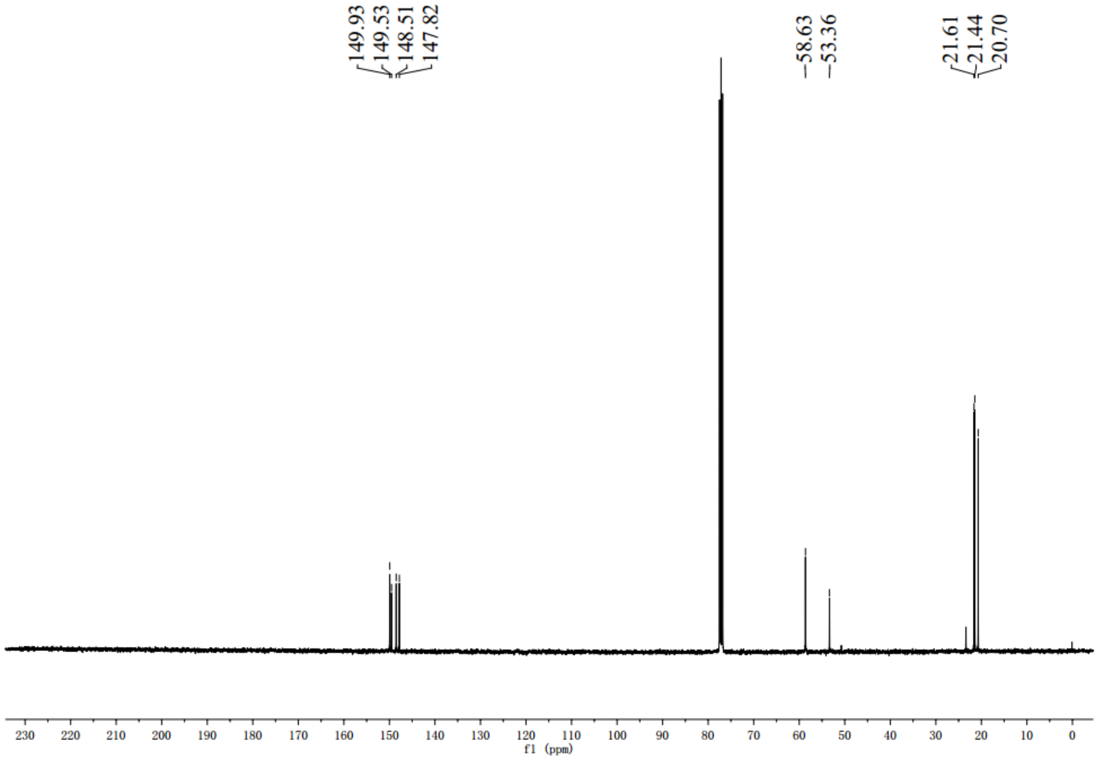

Figure S104 ^{13}C NMR spectrum of compound **6b.**

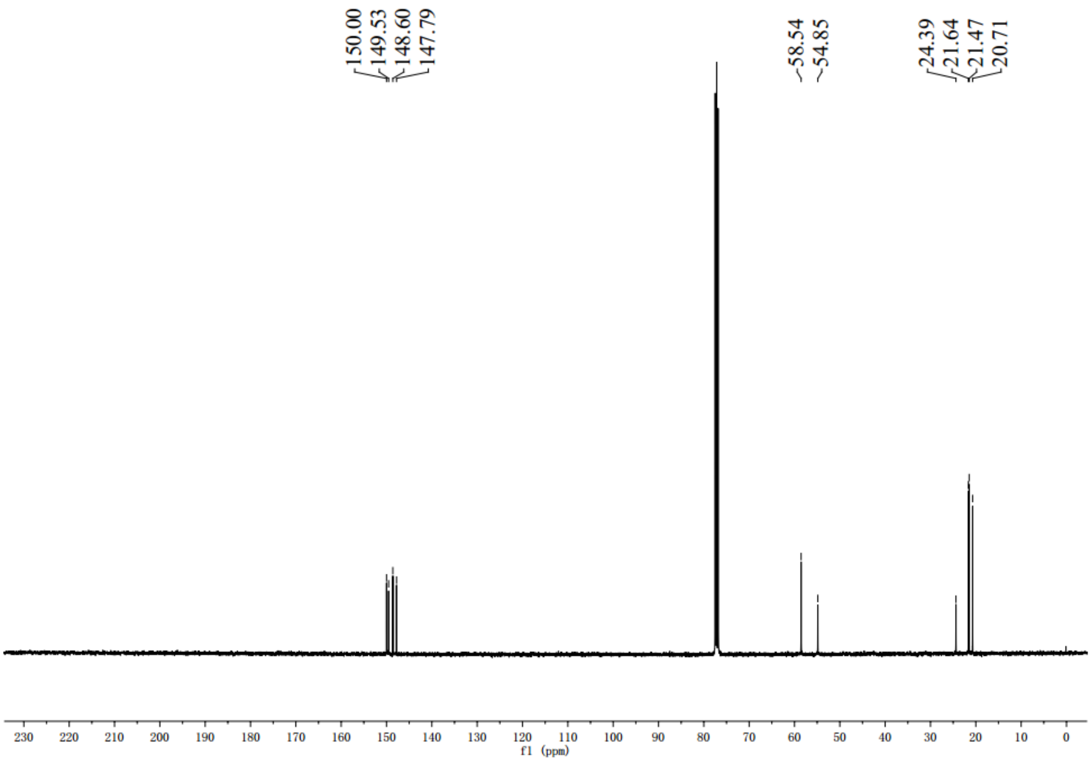

Figure S105 ^{13}C NMR spectrum of compound **6c.**

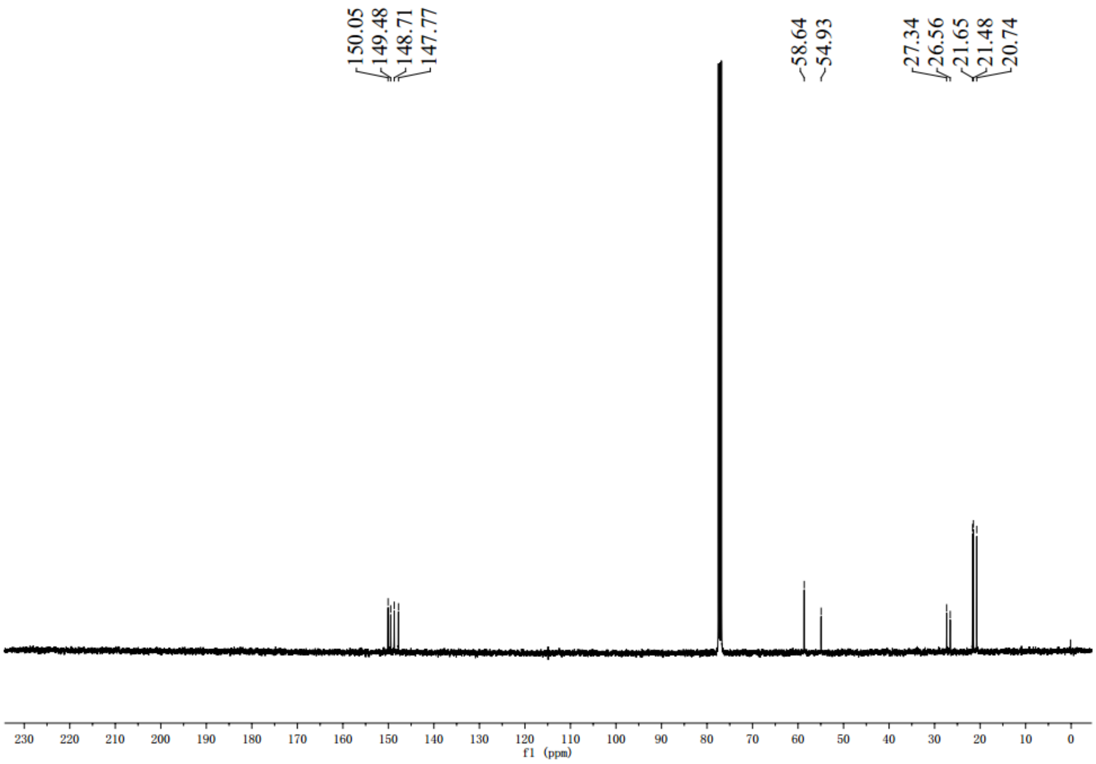

Figure S106 ^{13}C NMR spectrum of compound **6d.**

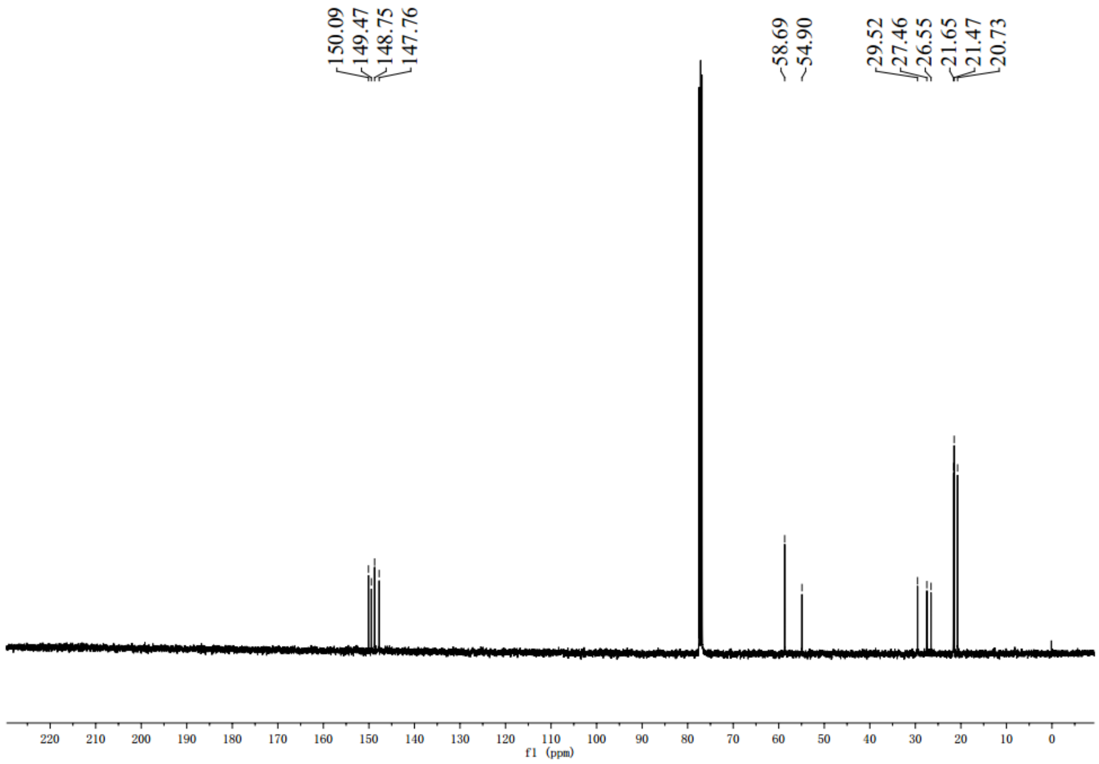

Figure S107 ^{13}C NMR spectrum of compound **6e.**

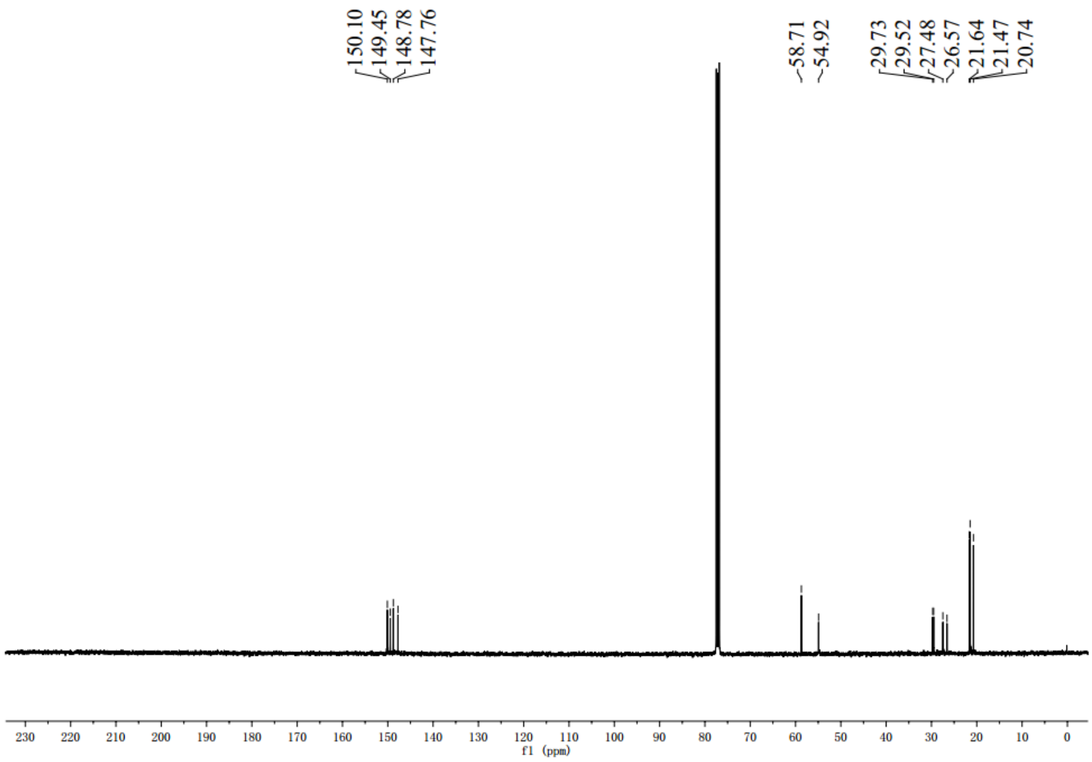

Figure S108 ^{13}C NMR spectrum of compound **6f.**

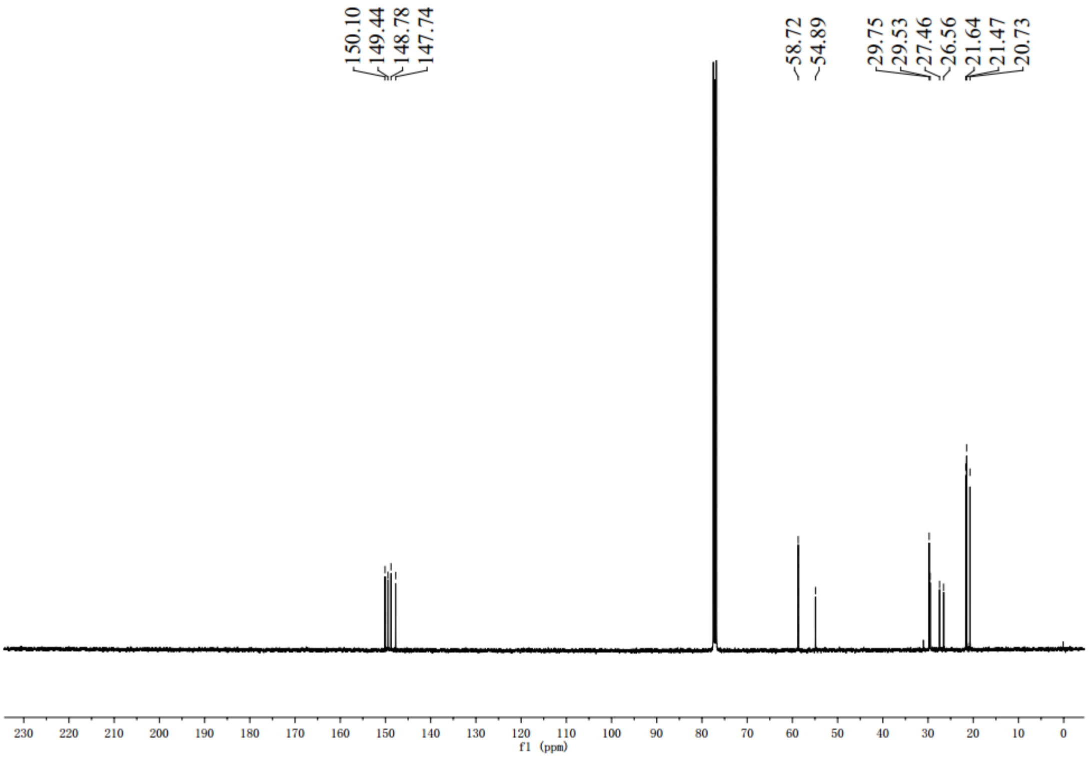

Figure S109 ^{13}C NMR spectrum of compound **6g.**

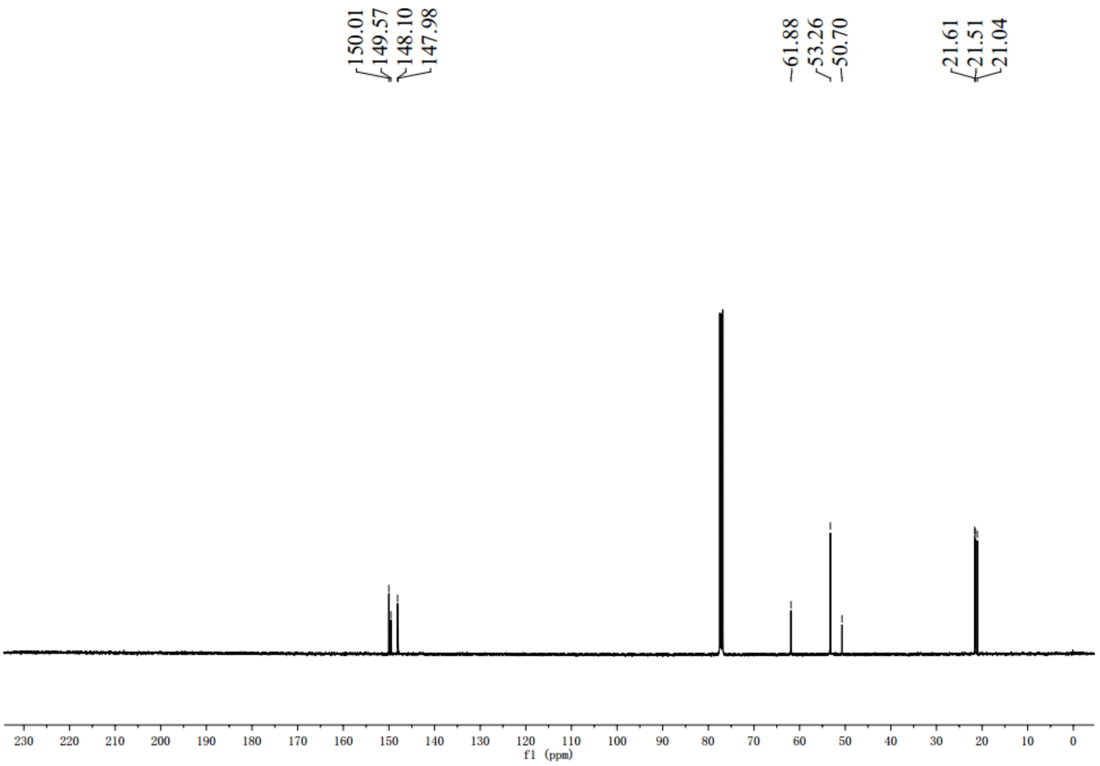

Figure S110 ^{13}C NMR spectrum of compound **6h.**

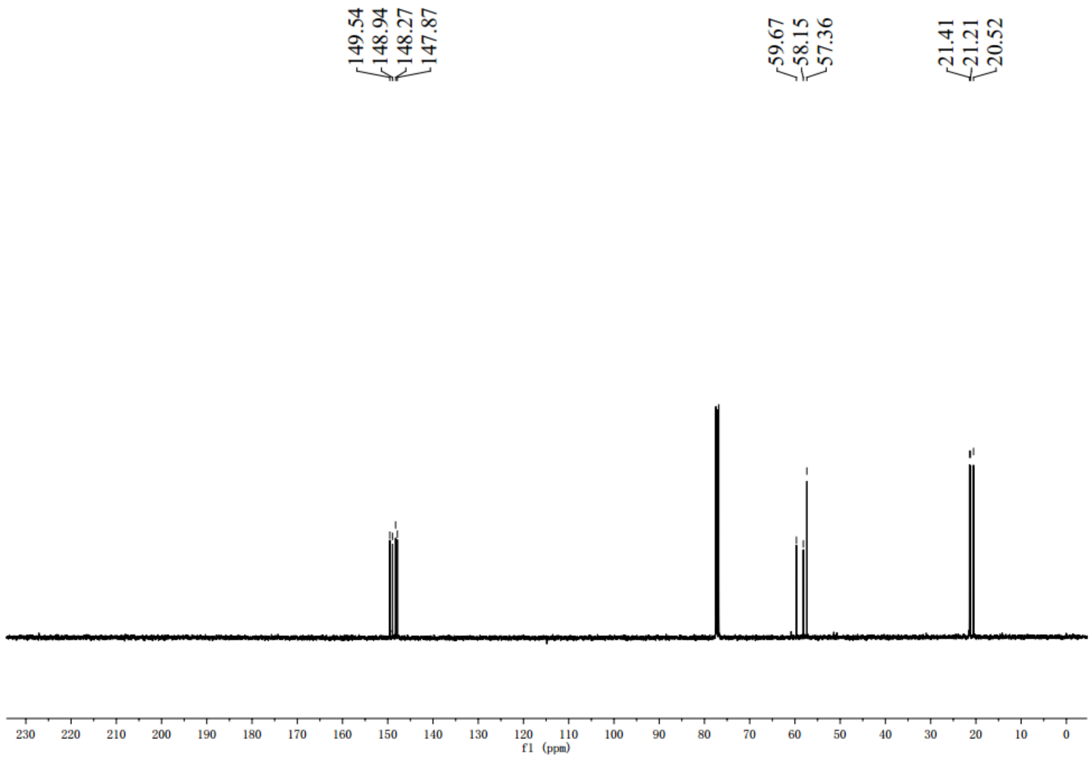

Figure S111 ^{13}C NMR spectrum of compound **6i.**

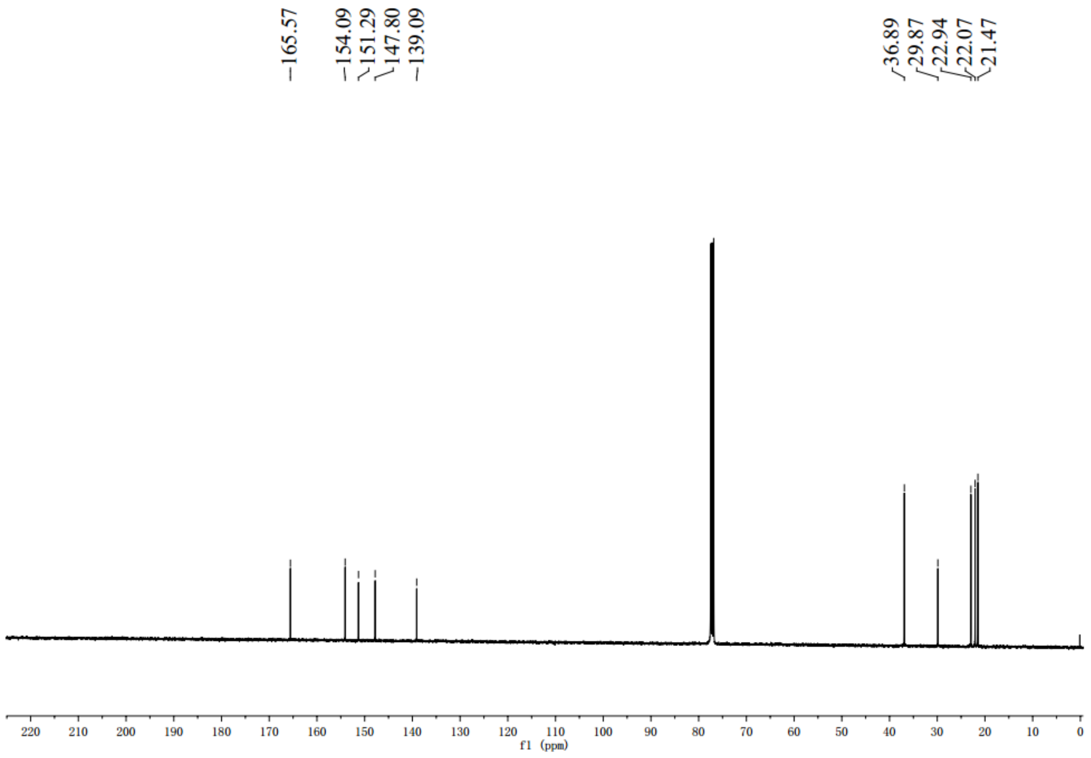

Figure S112 ^{13}C NMR spectrum of compound **8a.**

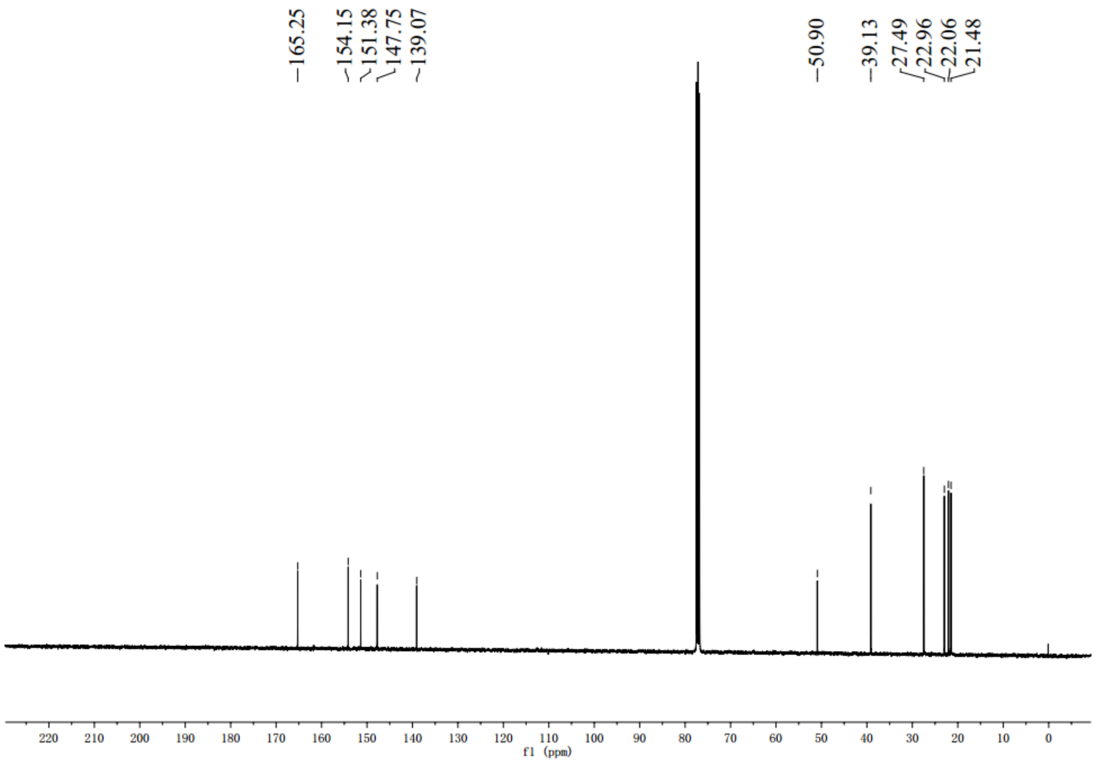

Figure S113 ^{13}C NMR spectrum of compound **8b.**

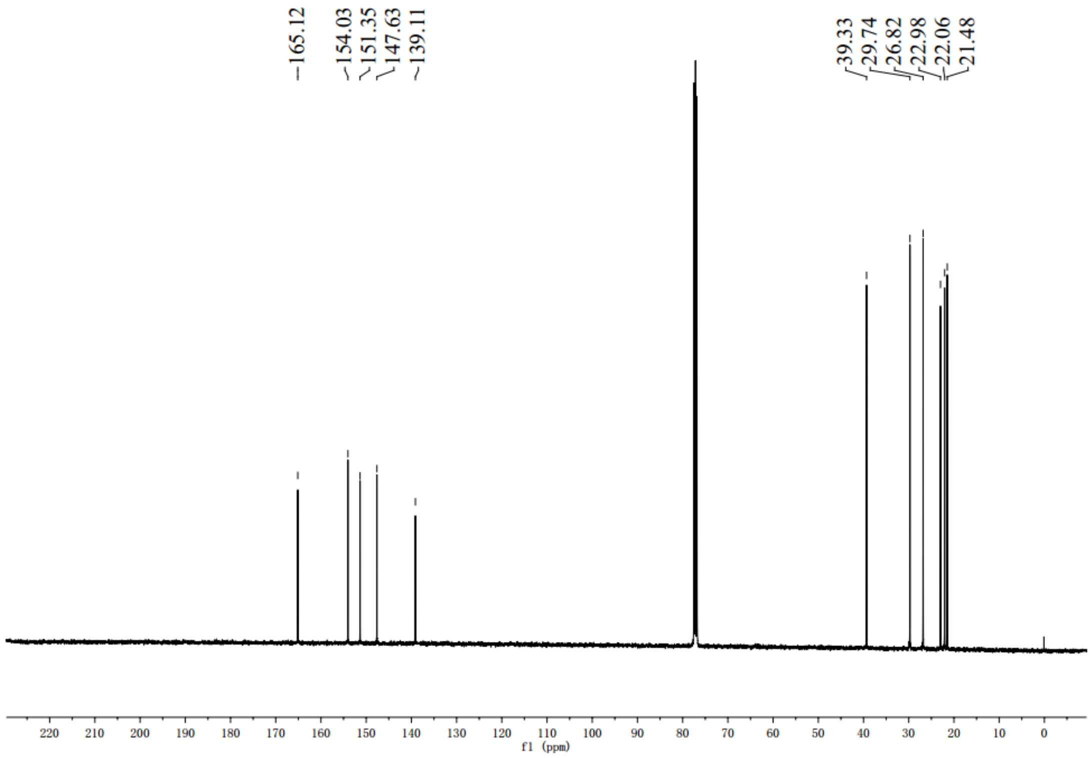

Figure S114 ^{13}C NMR spectrum of compound **8c.**

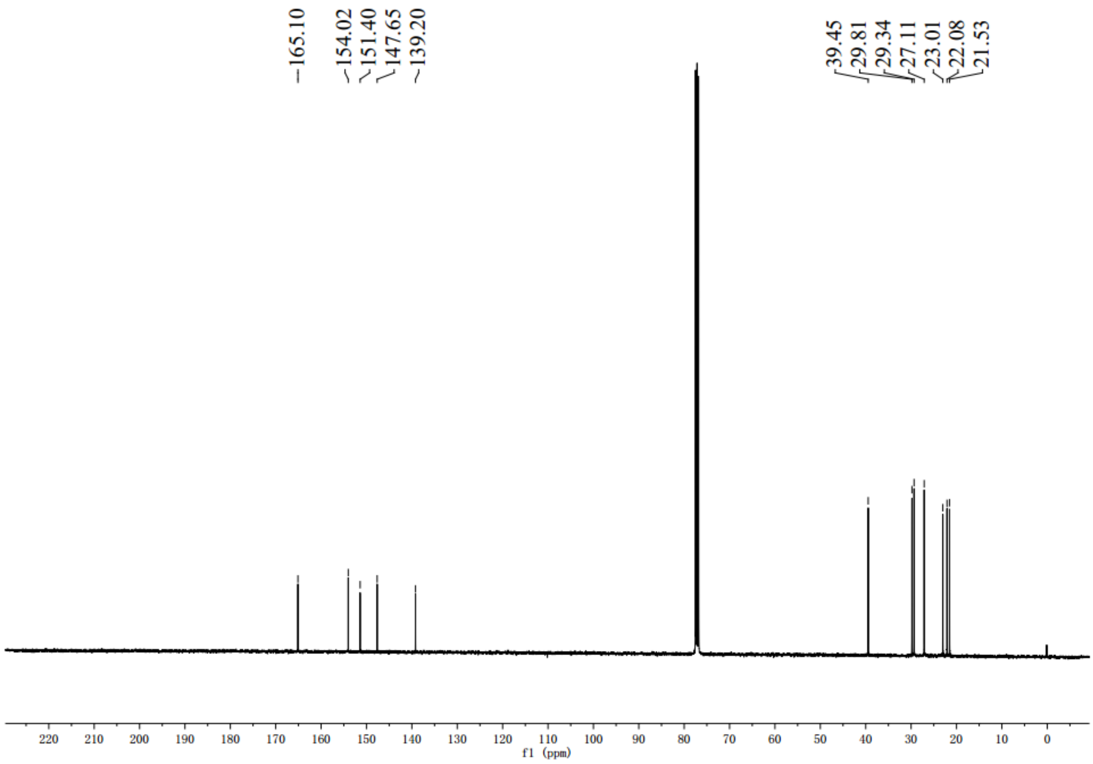

Figure S115 ^{13}C NMR spectrum of compound **8d.**

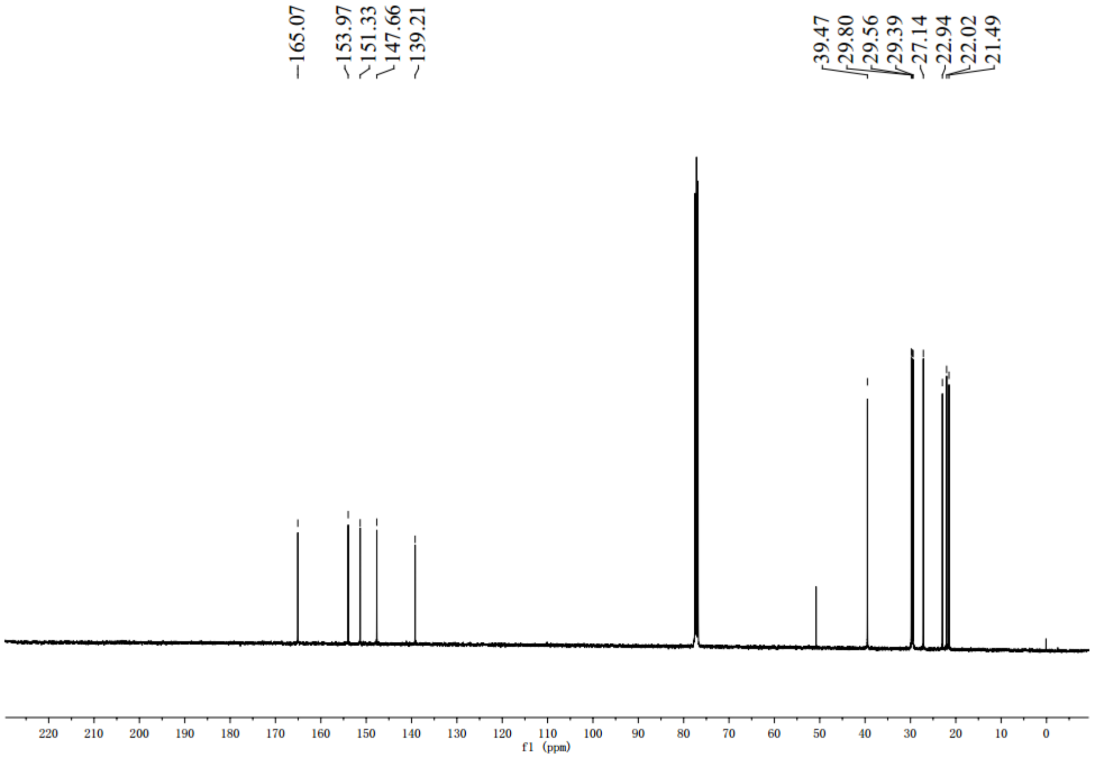

Figure S116 ^{13}C NMR spectrum of compound **8e.**

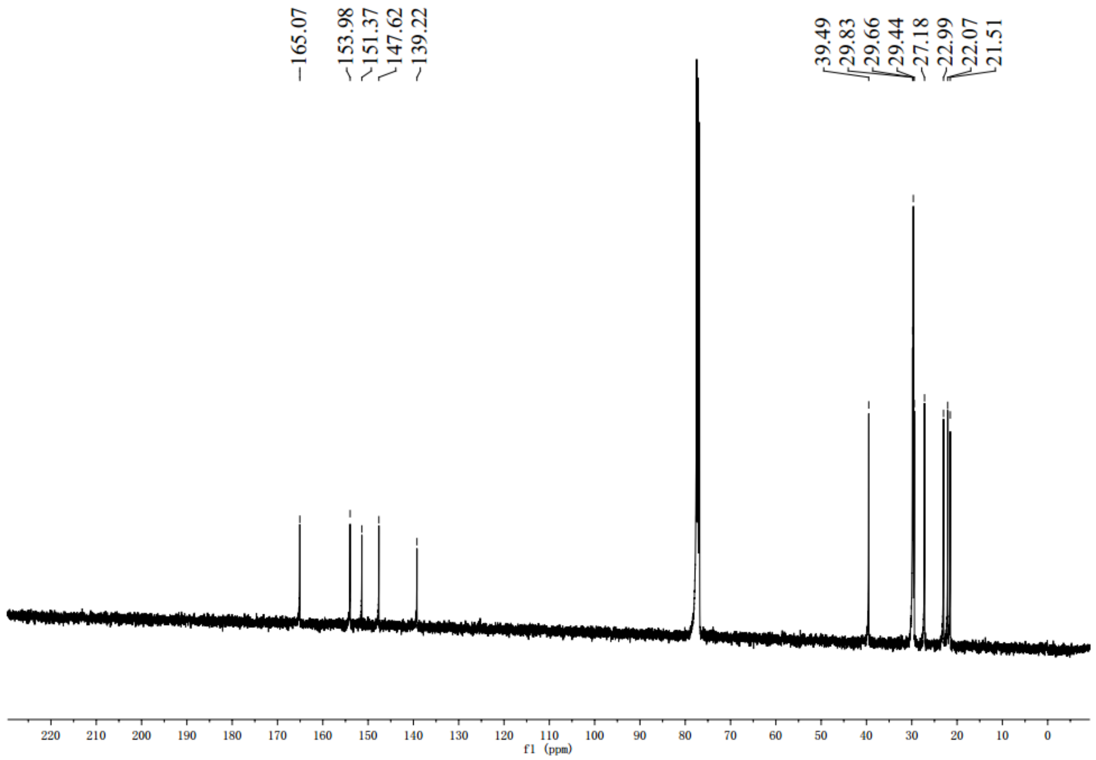

Figure S117 ^{13}C NMR spectrum of compound **8f.**

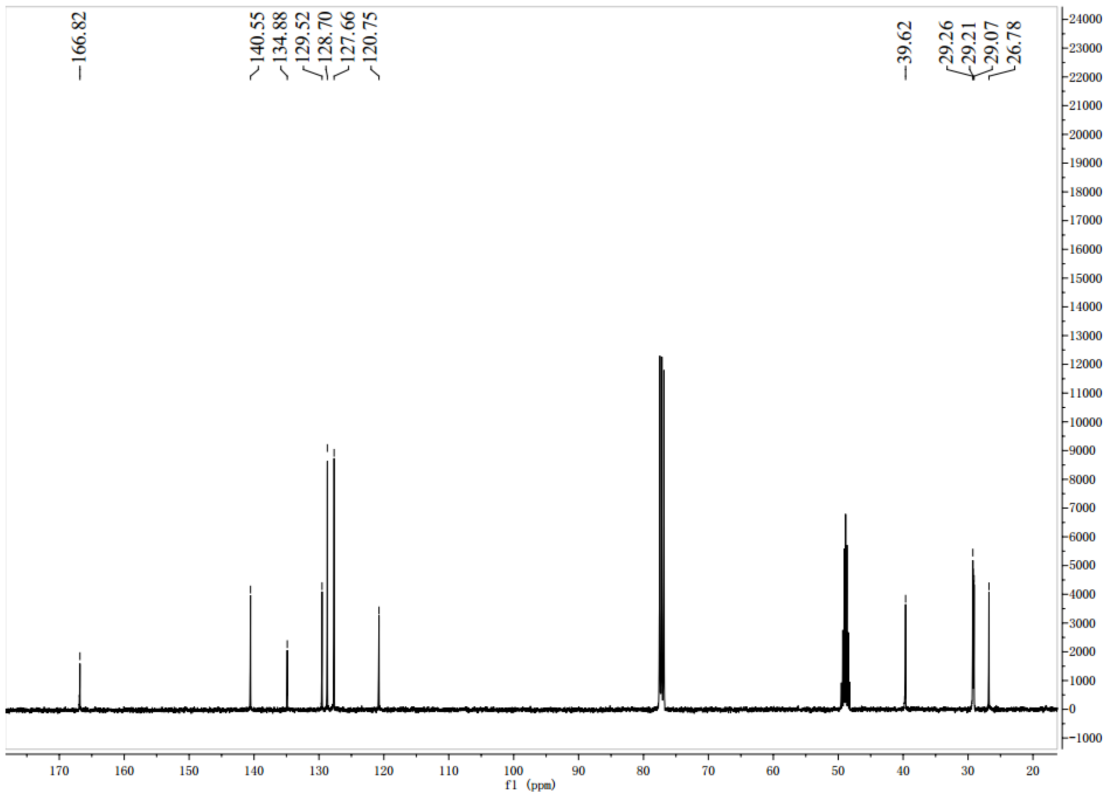

Figure S118 ^{13}C NMR spectrum of compound **9e.**

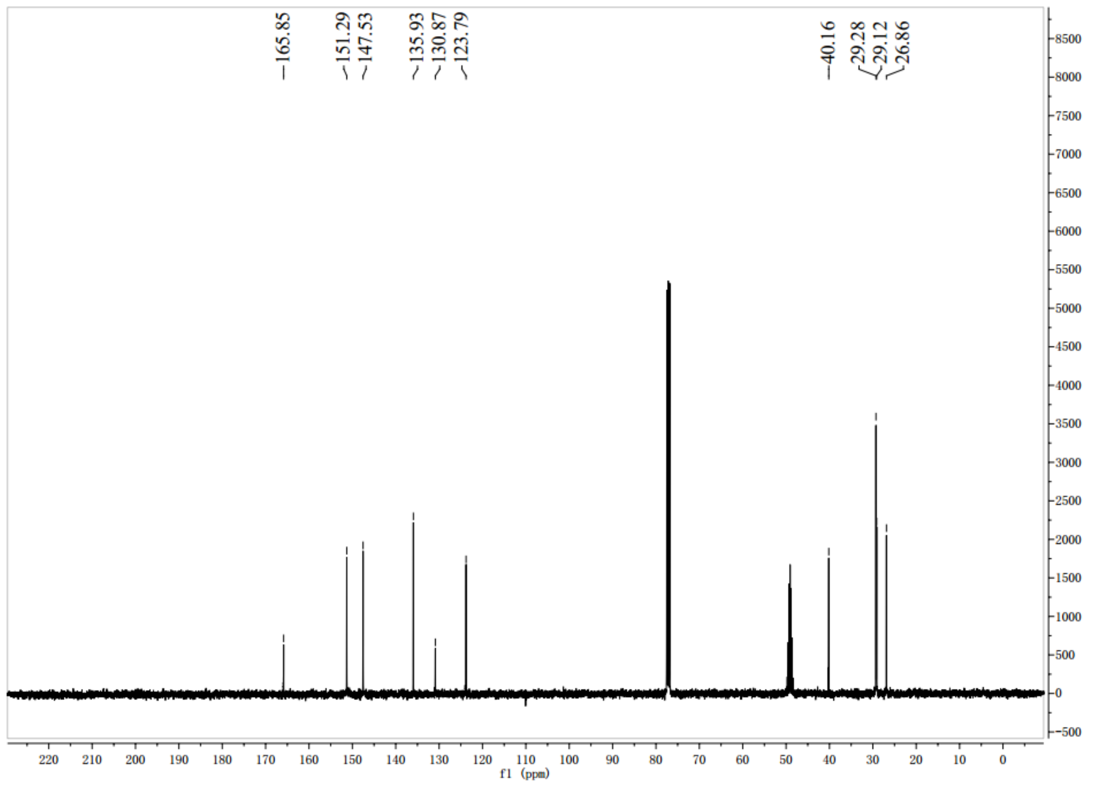

Figure S119 ^{13}C NMR spectrum of compound **10e.**

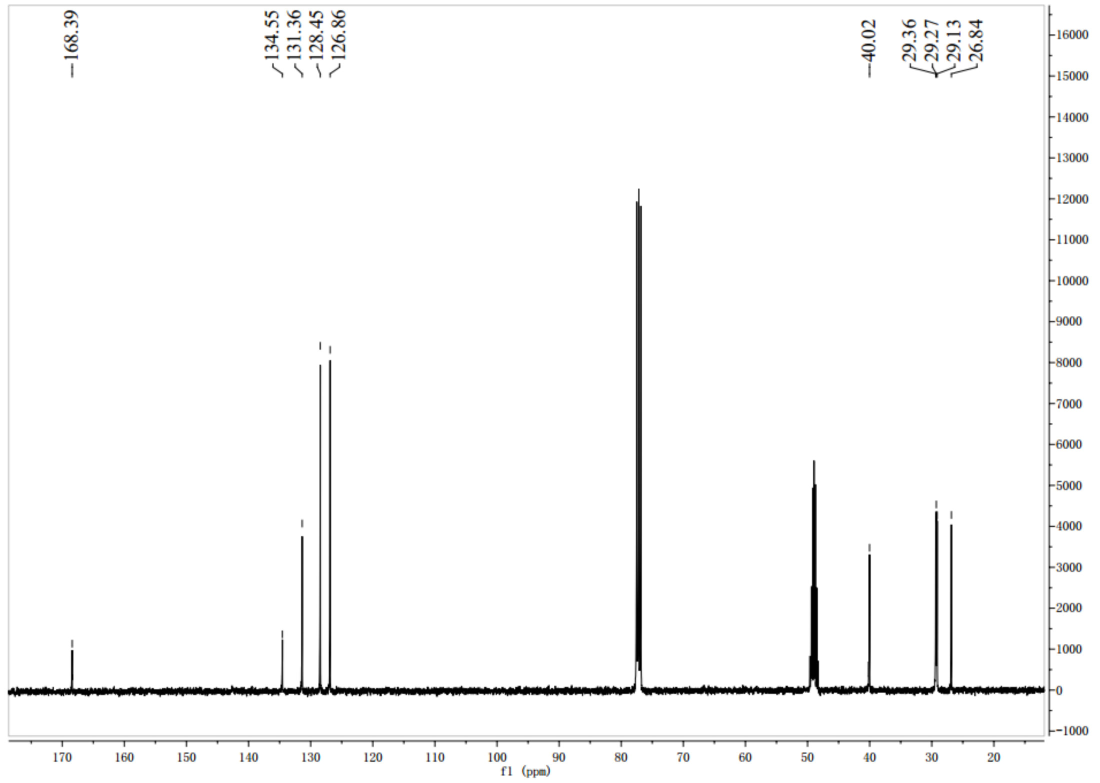

Figure S120 ^{13}C NMR spectrum of compound **11e.**

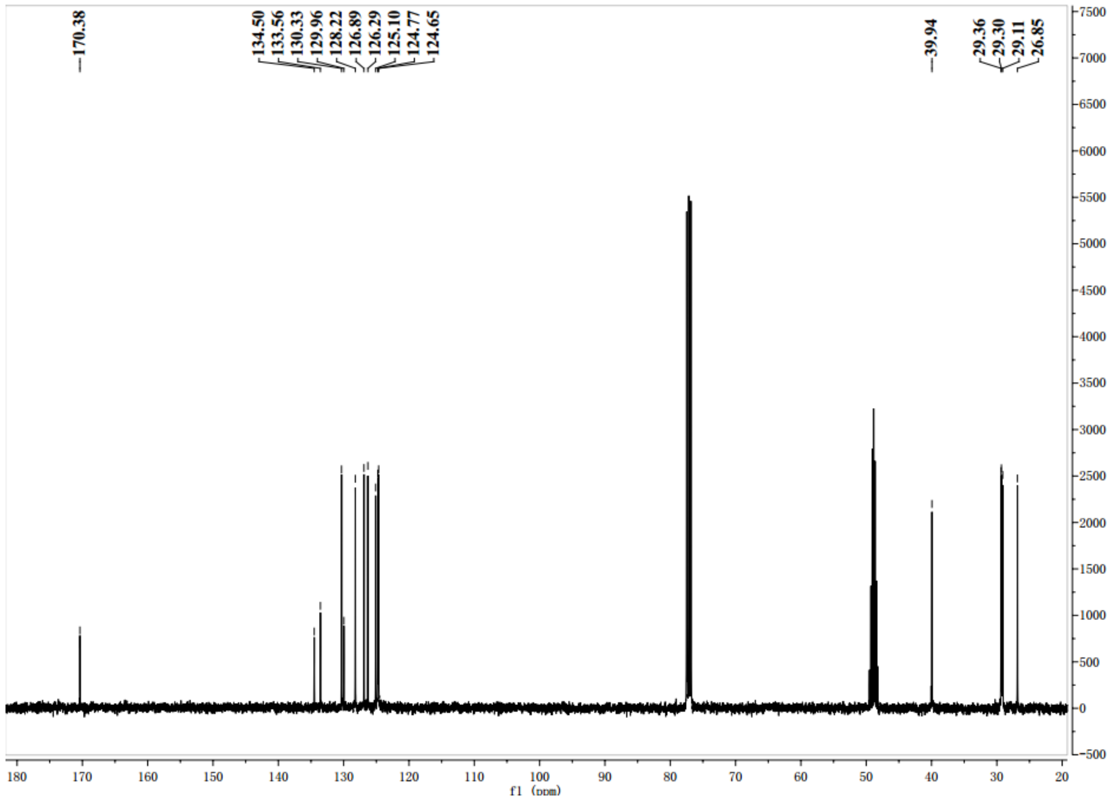

Figure S121 ^{13}C NMR spectrum of compound **12e.**

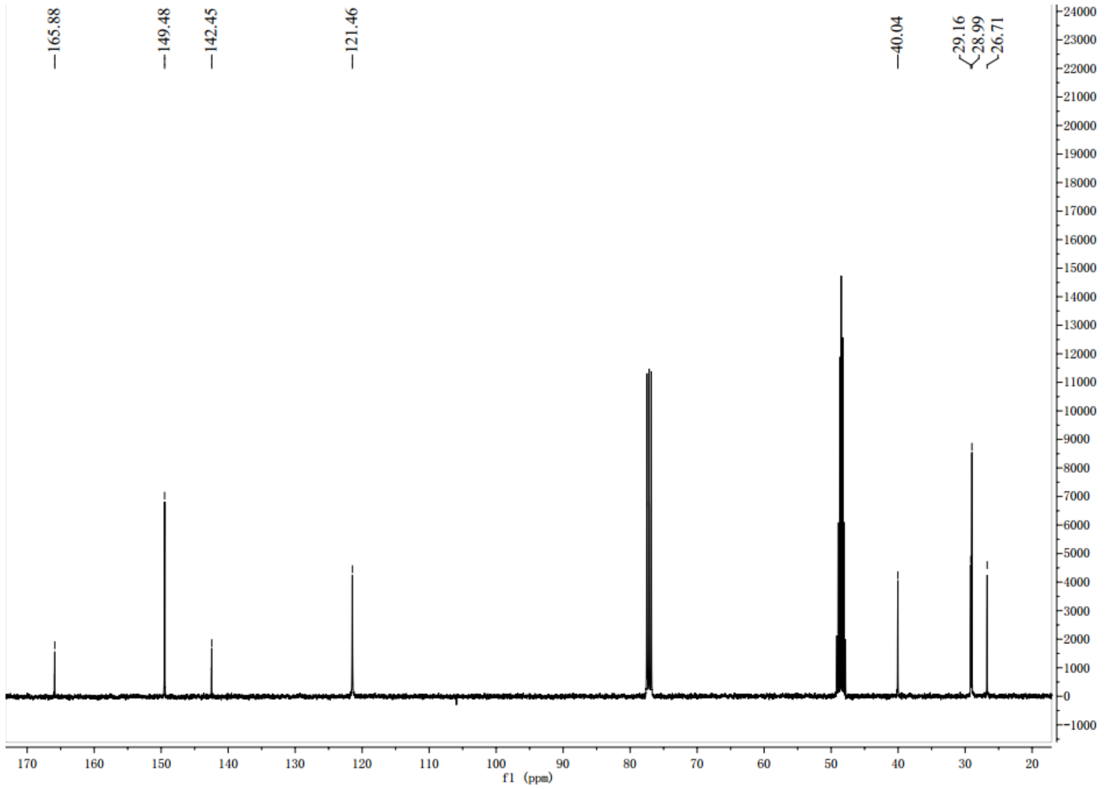

Figure S122 ^{13}C NMR spectrum of compound **13e.**

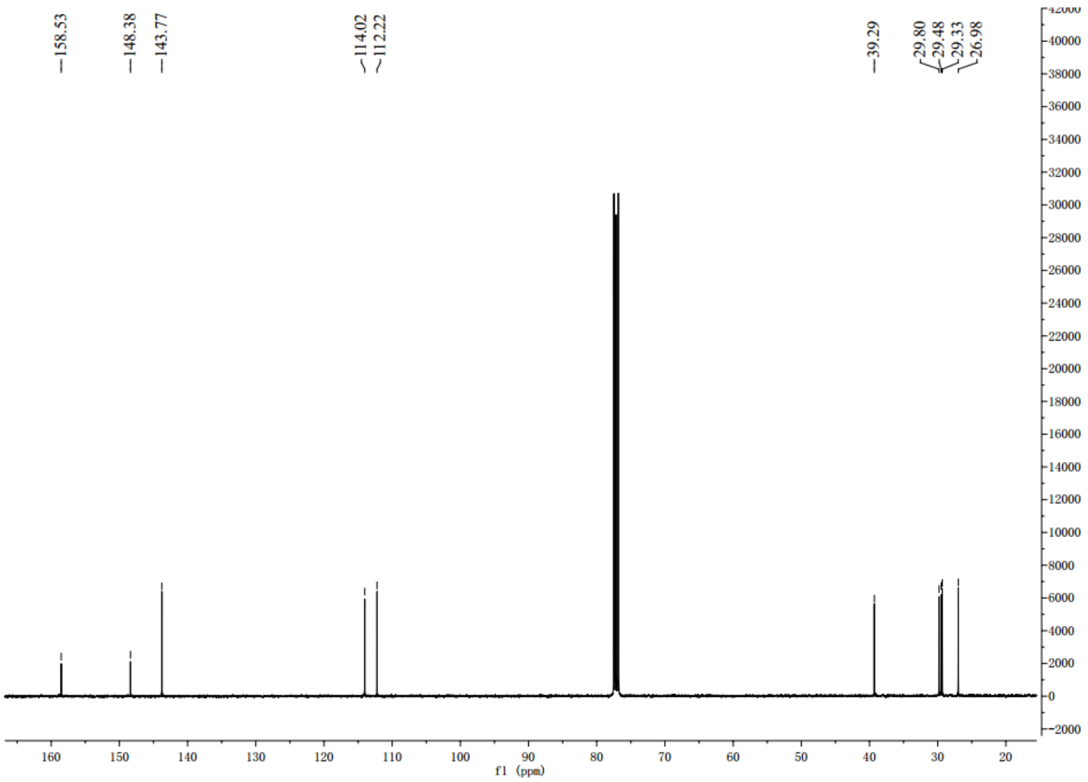

Figure S123 ^{13}C NMR spectrum of compound **14e.**

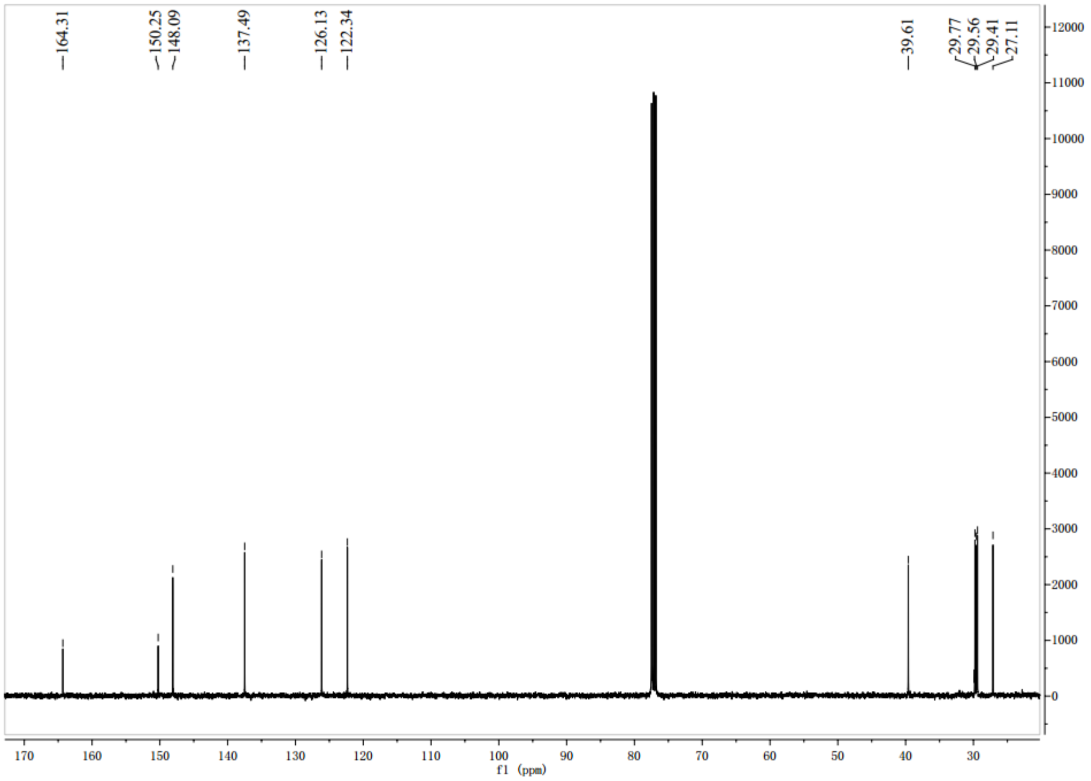

Figure S124 ^{13}C NMR spectrum of compound **15e.**

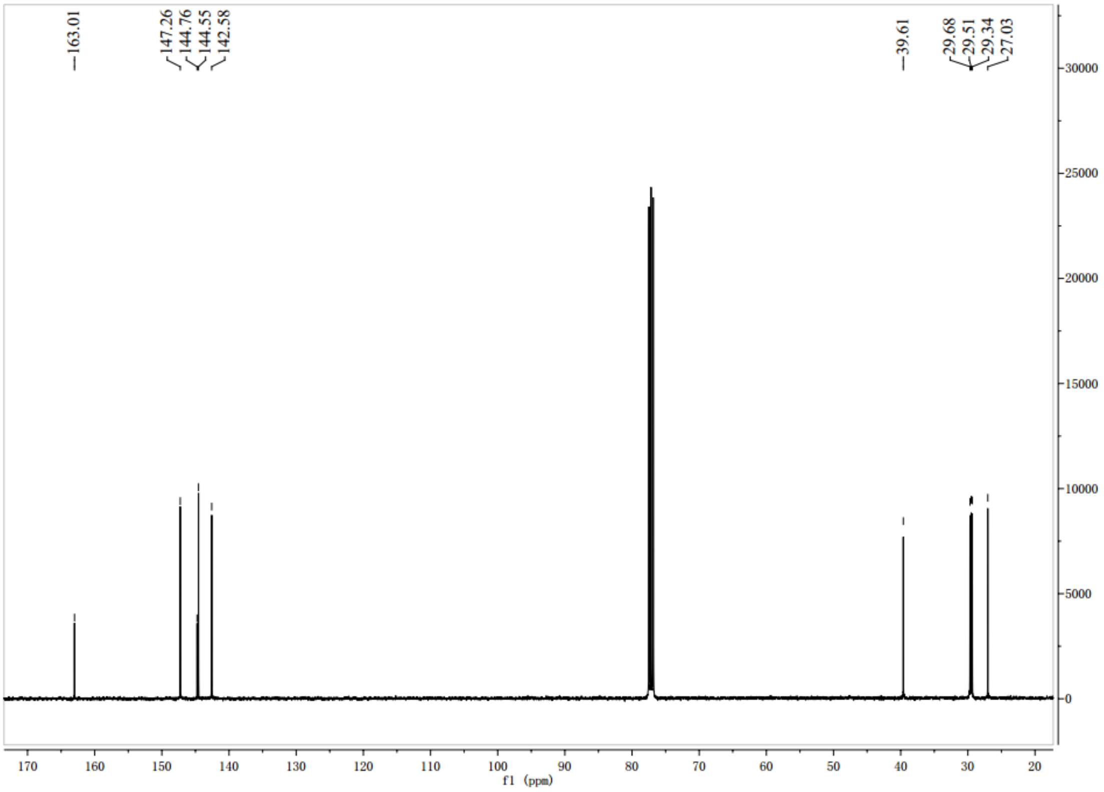

Figure S125 ^{13}C NMR spectrum of compound **16e.**

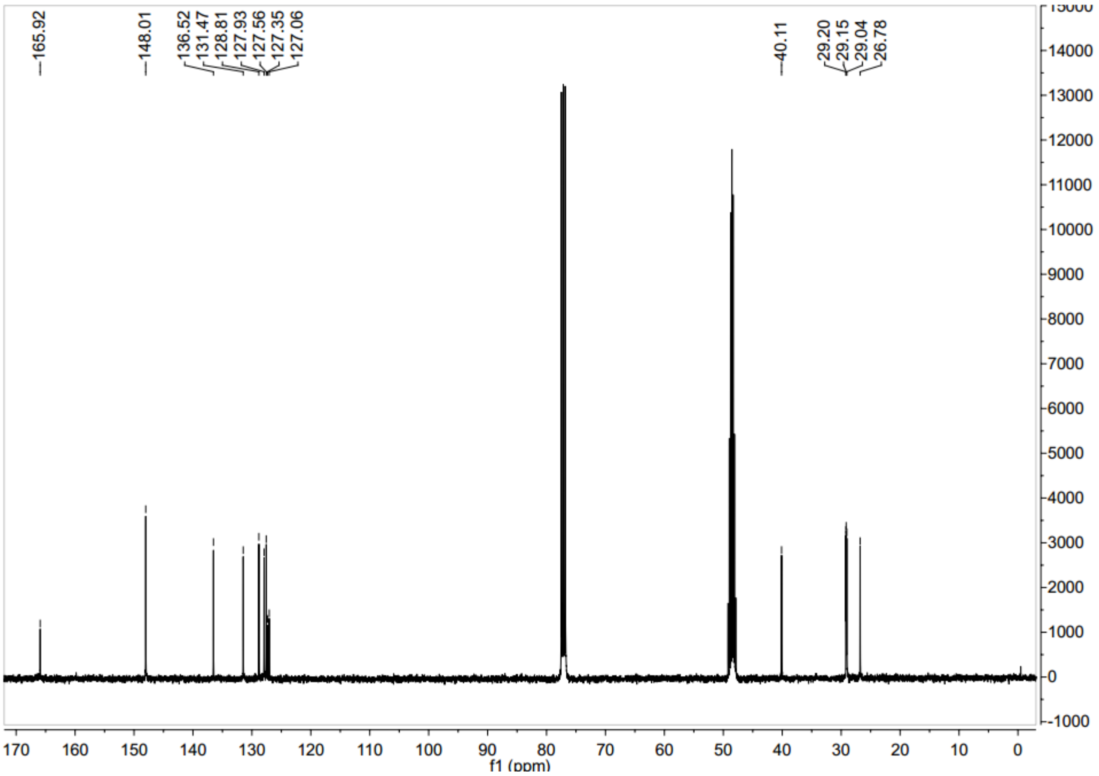

Figure S126 ^{13}C NMR spectrum of compound **17e.**

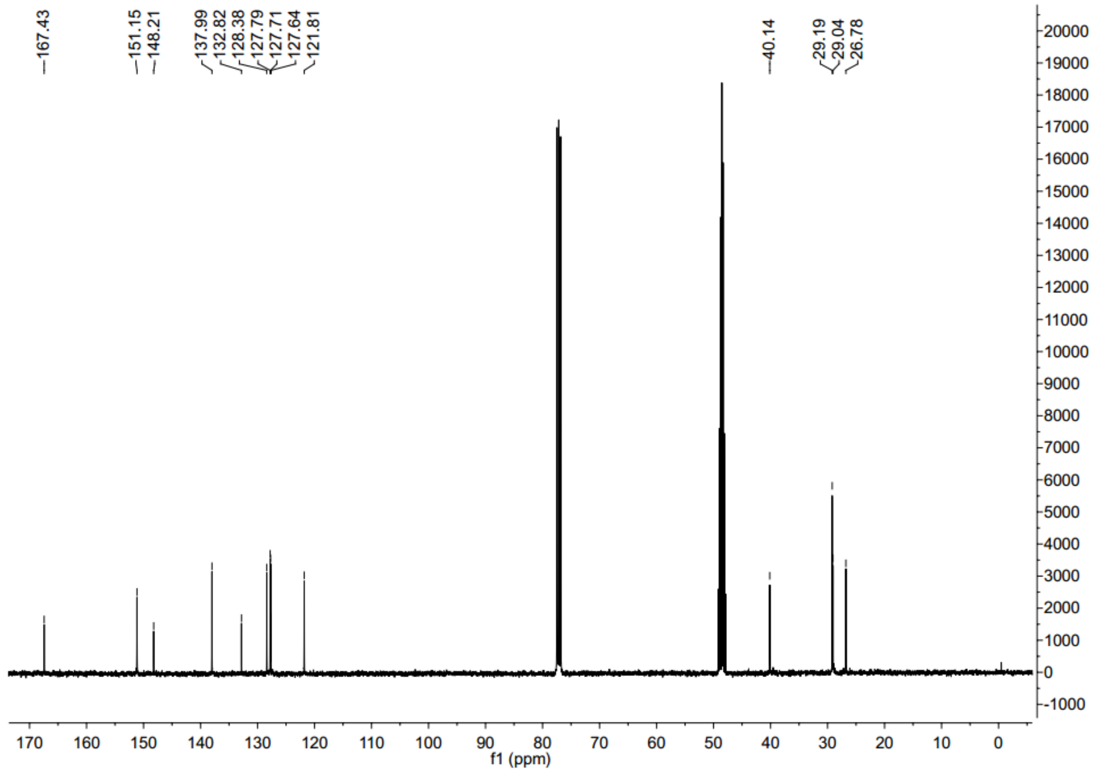

Figure S127 ^{13}C NMR spectrum of compound **18e.**

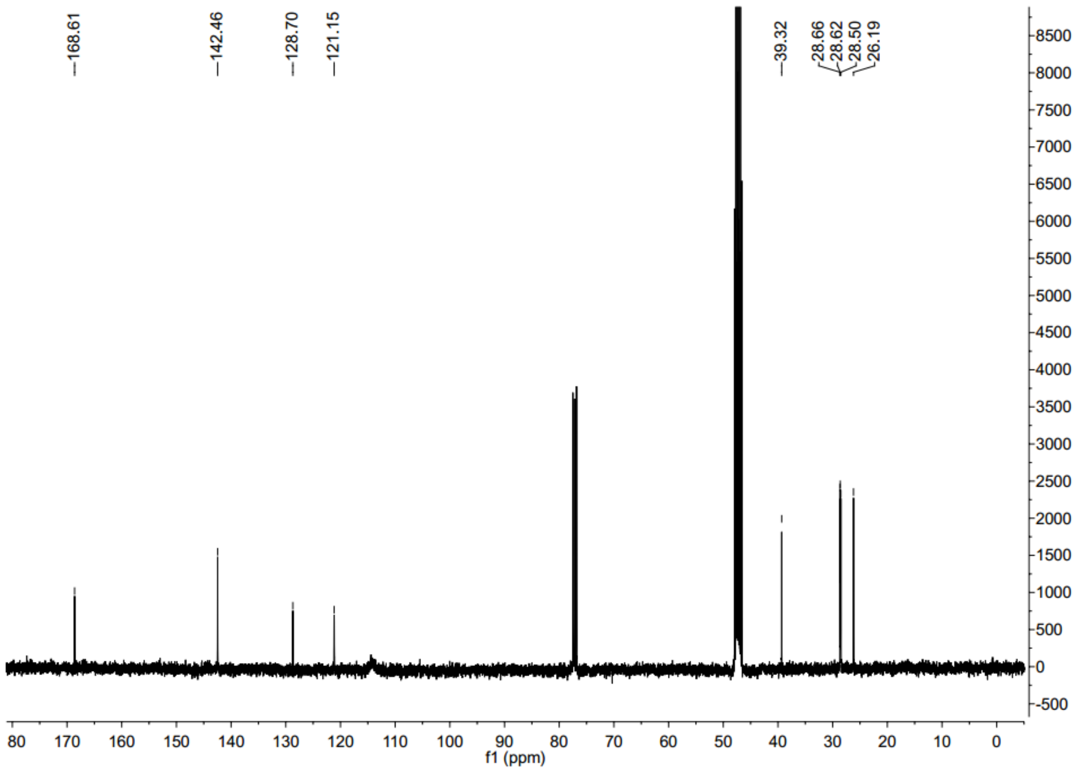

Figure S128 ^{13}C NMR spectrum of compound **19e.**

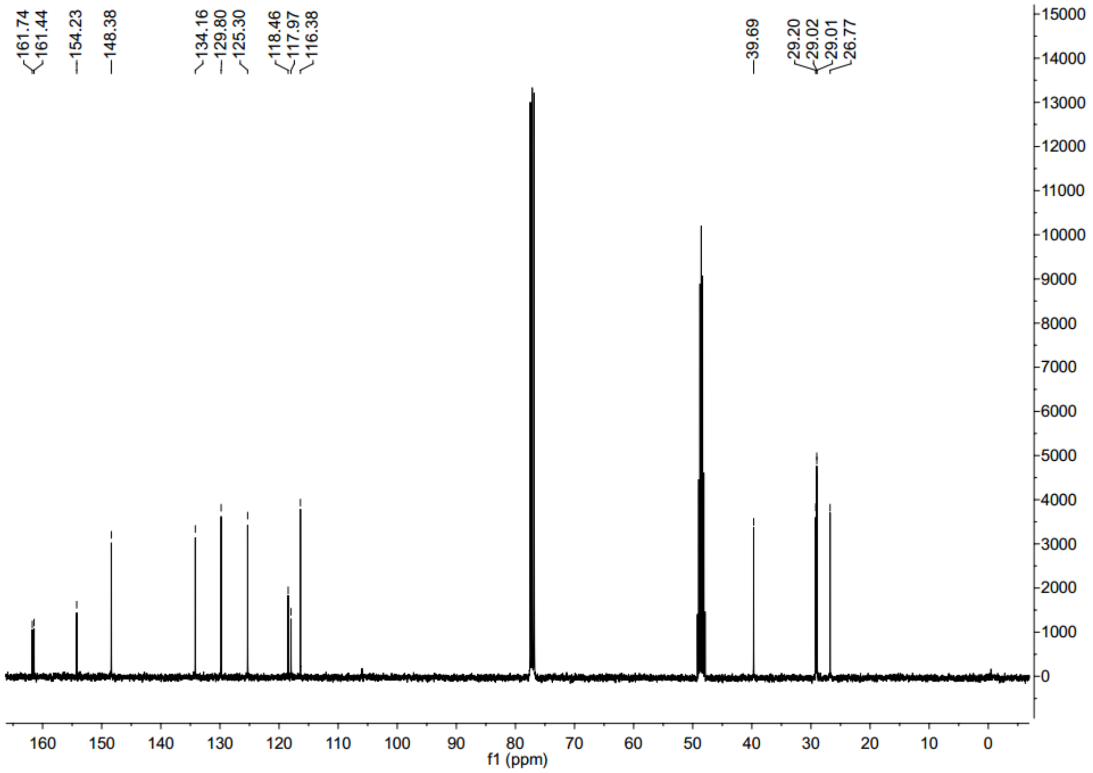

Figure S129 ^{13}C NMR spectrum of compound **20e.**

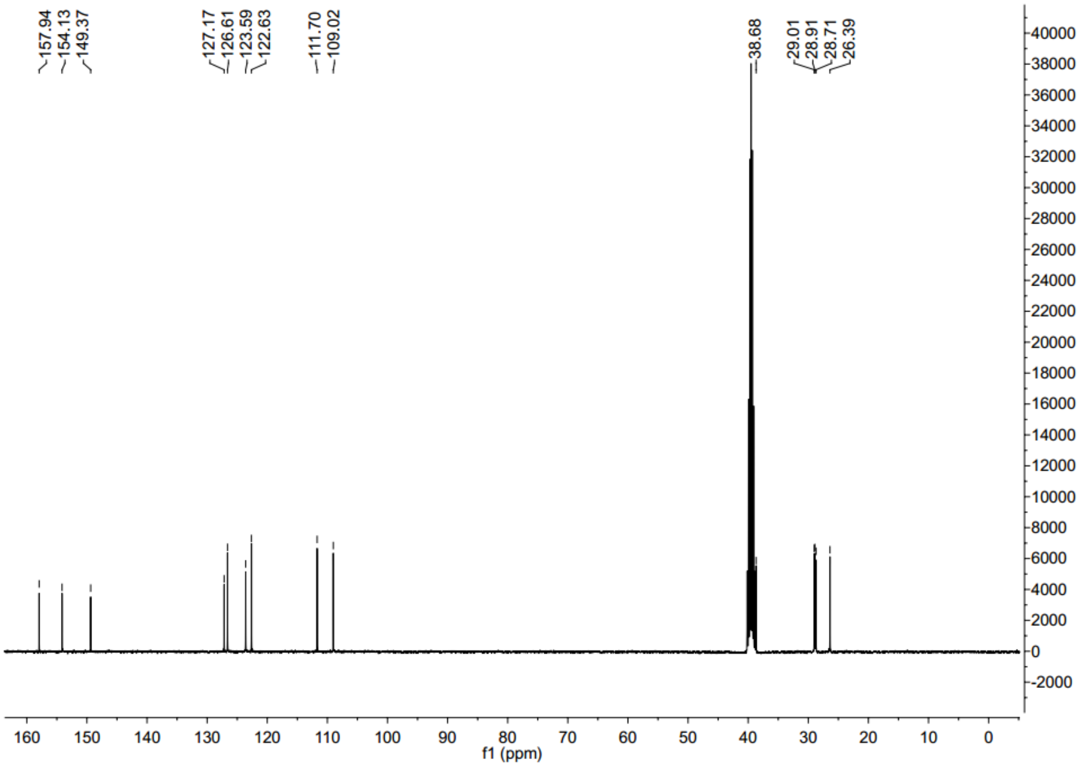

Figure S130 ^{13}C NMR spectrum of compound **21e.**

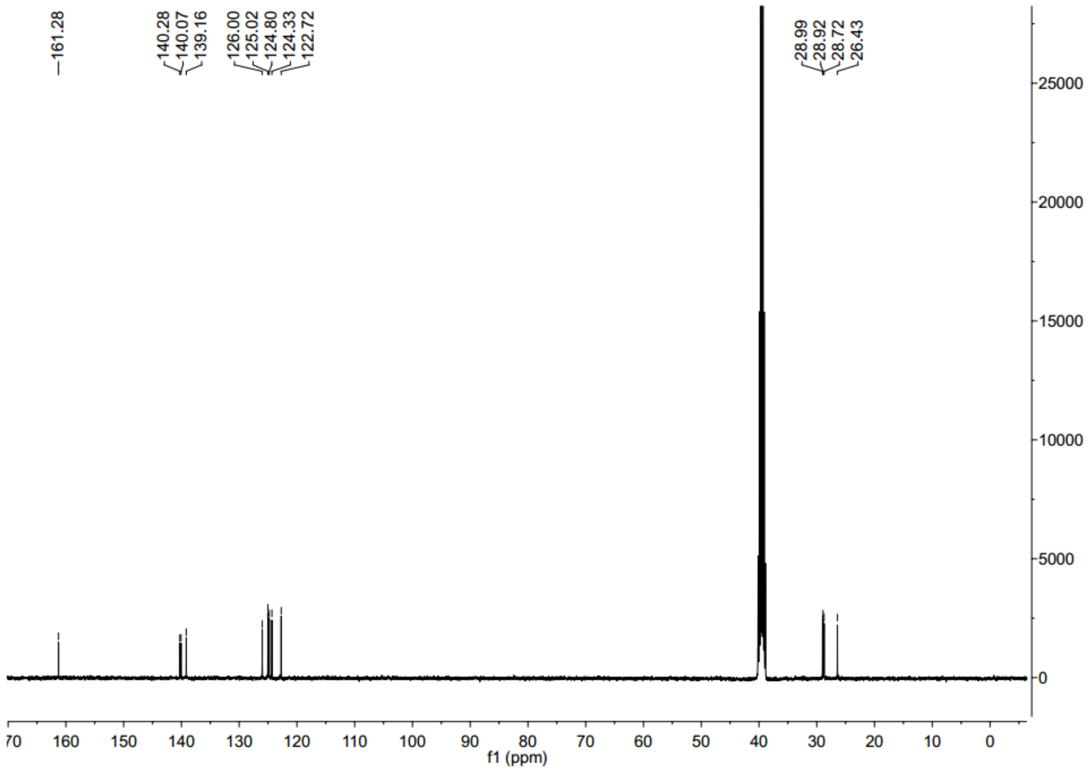

Figure S131 ^{13}C NMR spectrum of compound **22e.**

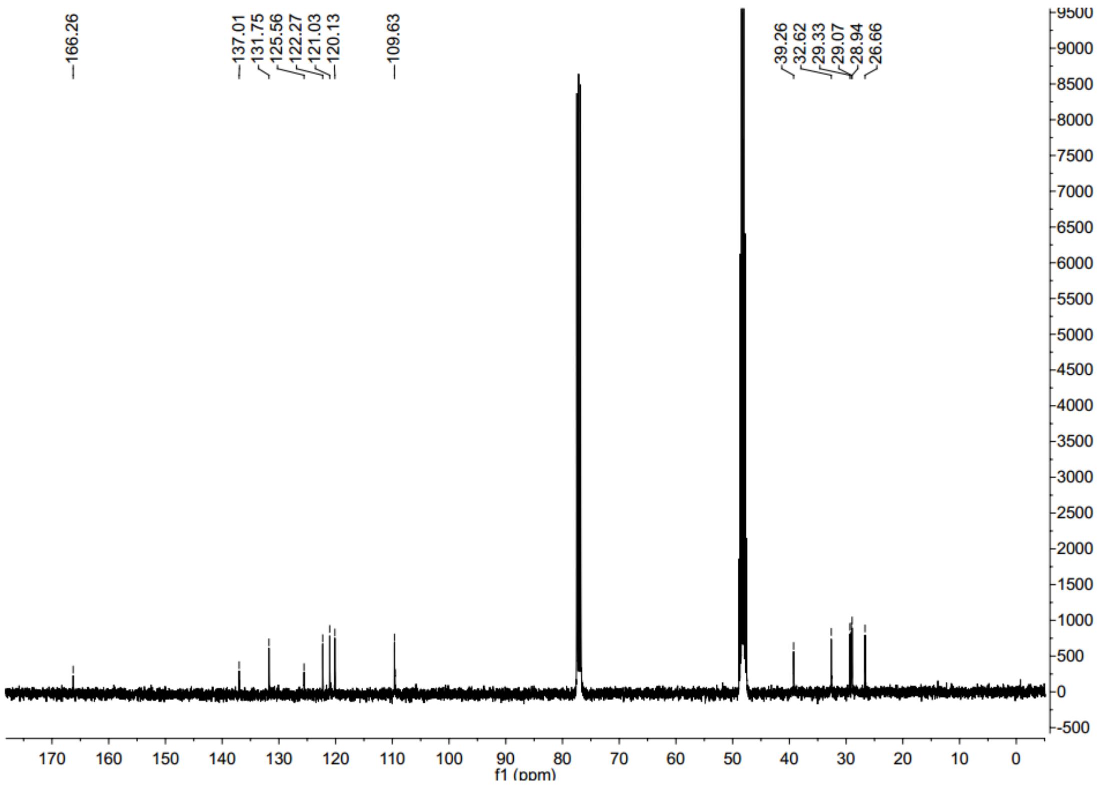

Figure S132 ^{13}C NMR spectrum of compound **23e.**

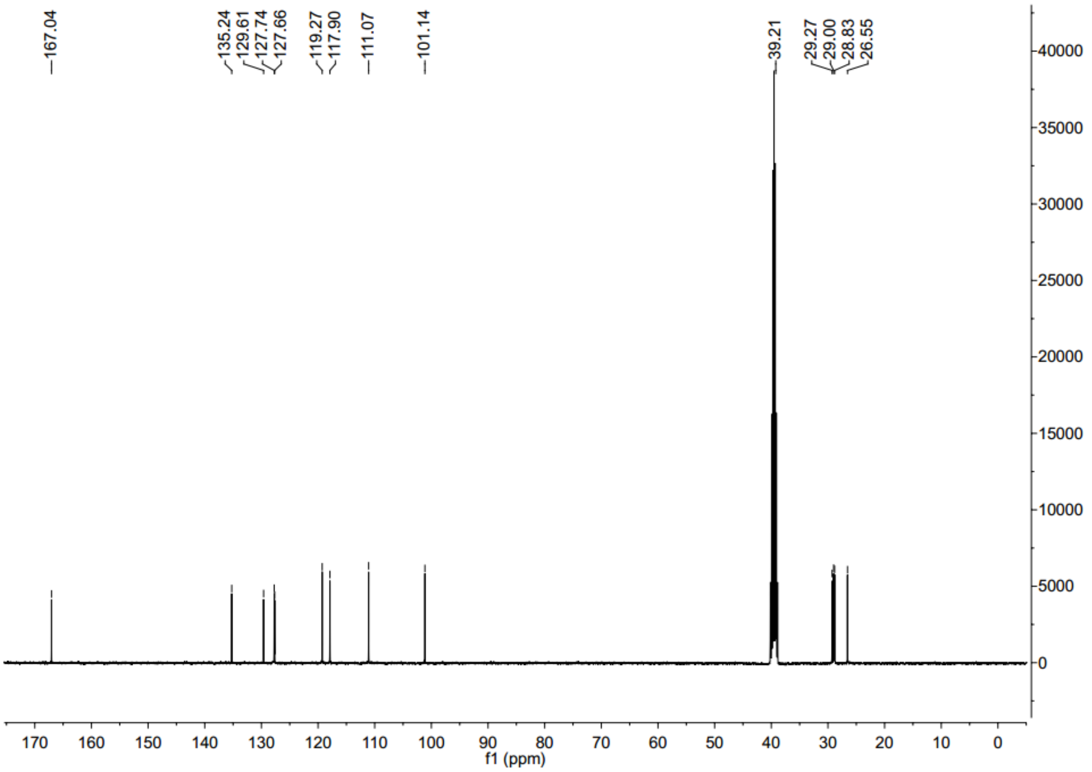

Figure S133 ^{13}C NMR spectrum of compound **24e.**

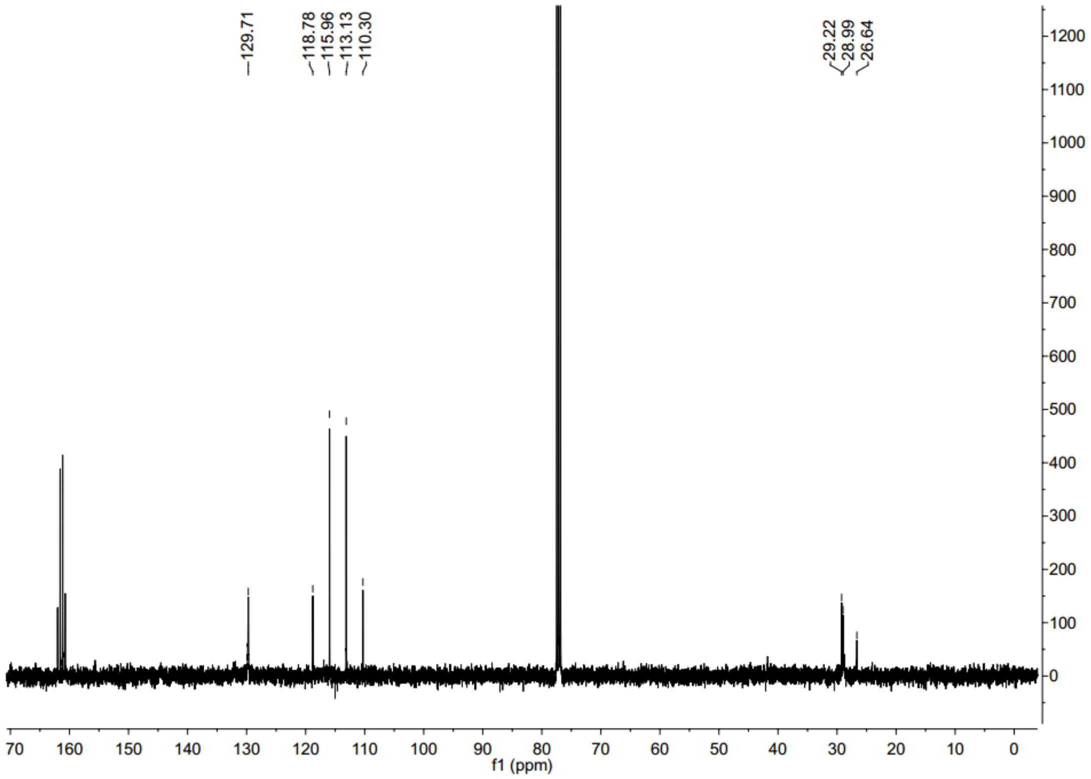

Figure S134 ^{13}C NMR spectrum of compound **25e.**

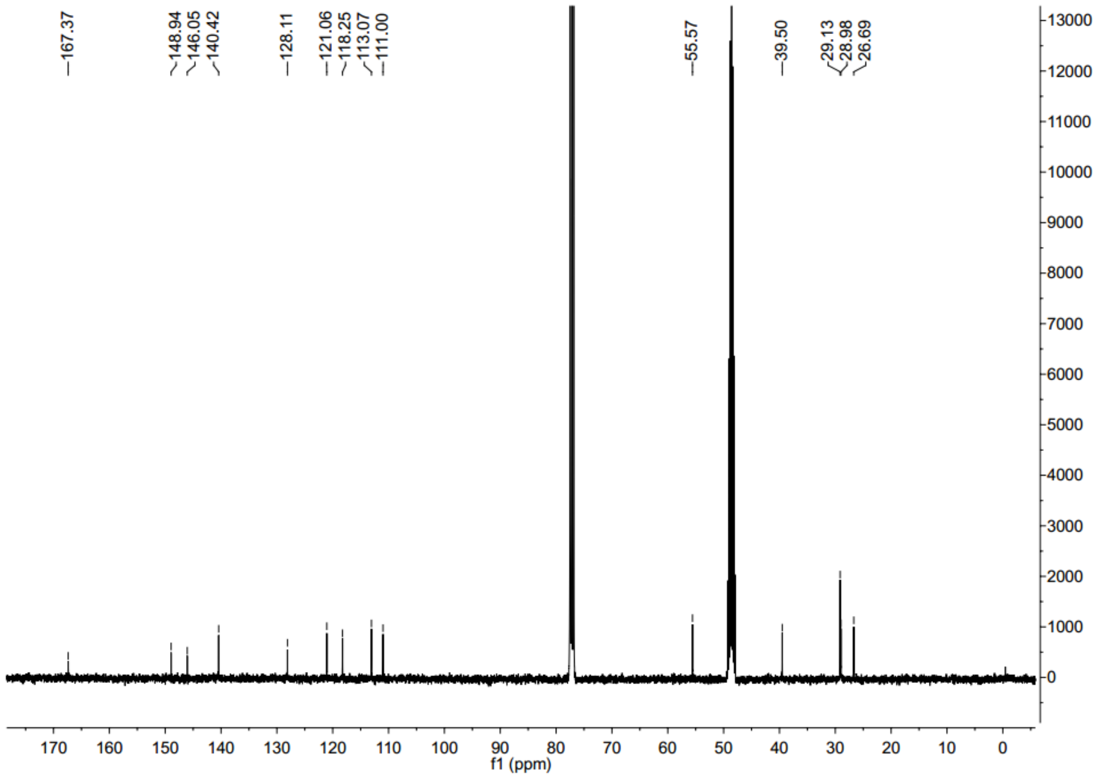

Figure S135 ^{13}C NMR spectrum of compound **26e.**

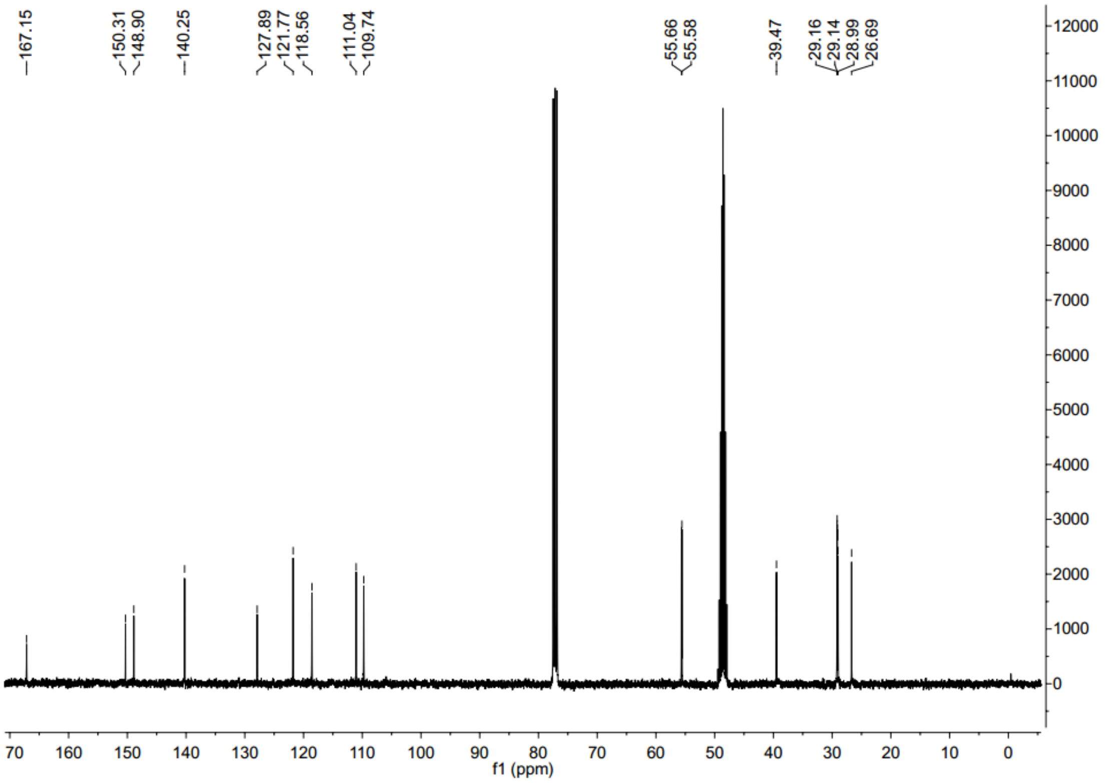

Figure S136 ^{13}C NMR spectrum of compound **27e.**

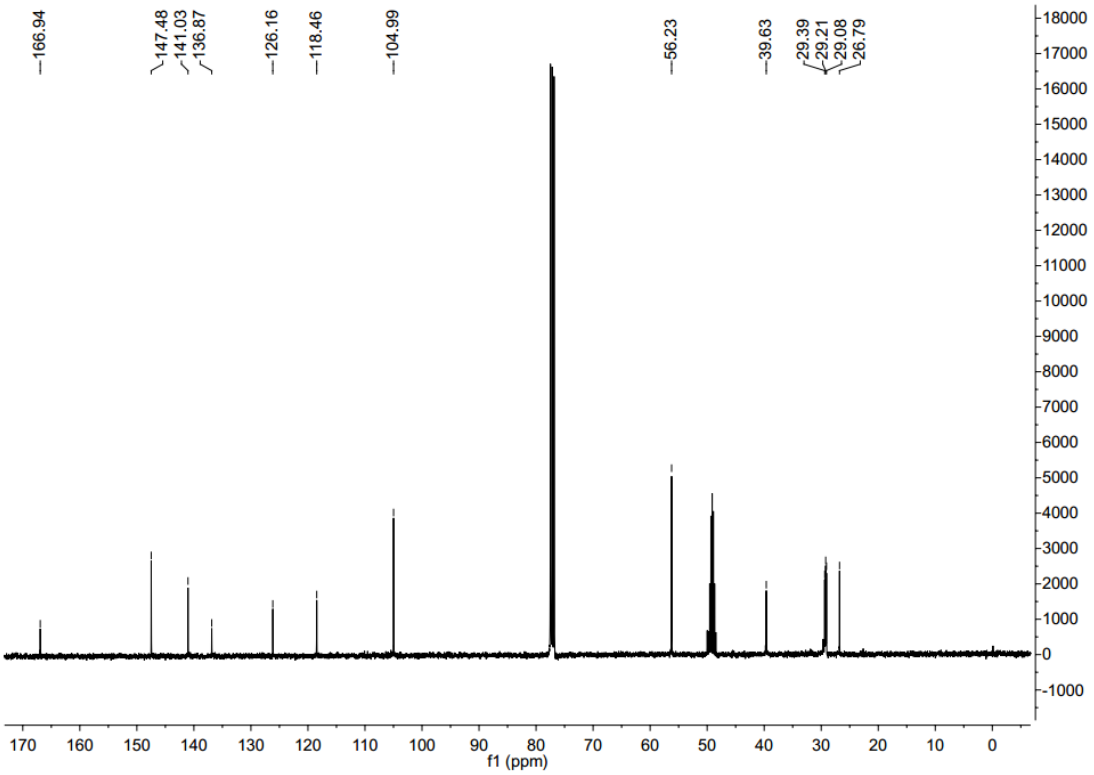

Figure S137 ^{13}C NMR spectrum of compound **28e.**

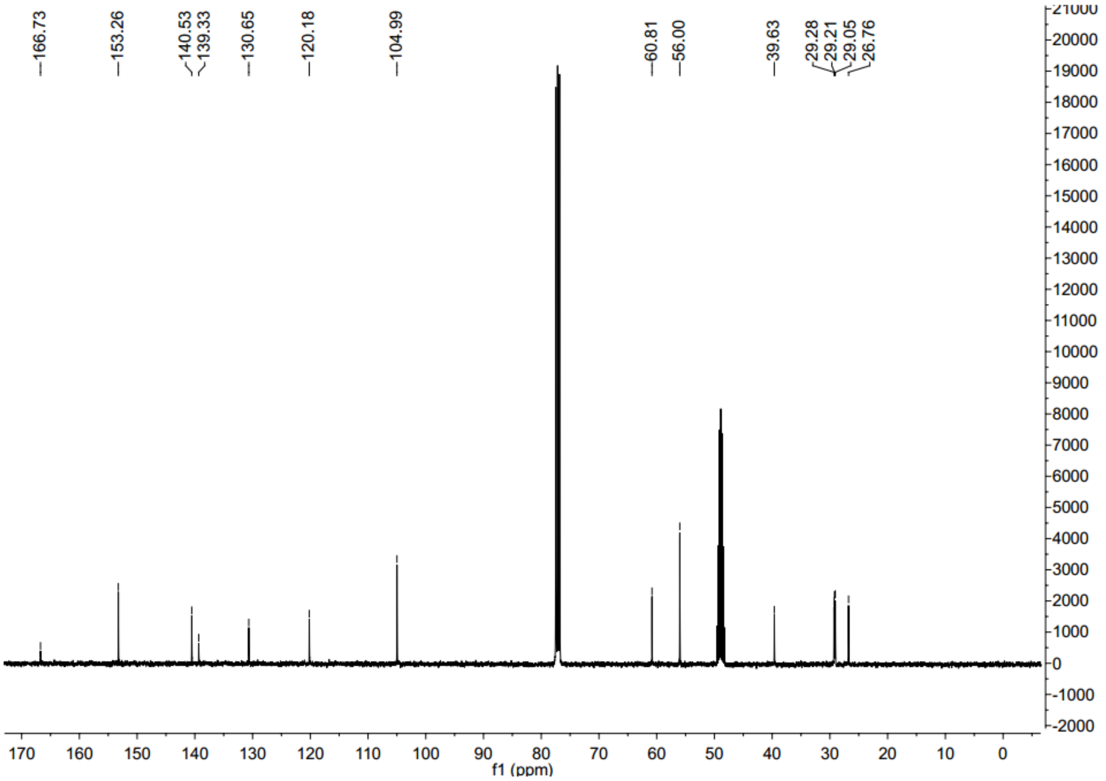

Figure S138 ^{13}C NMR spectrum of compound **29e.**

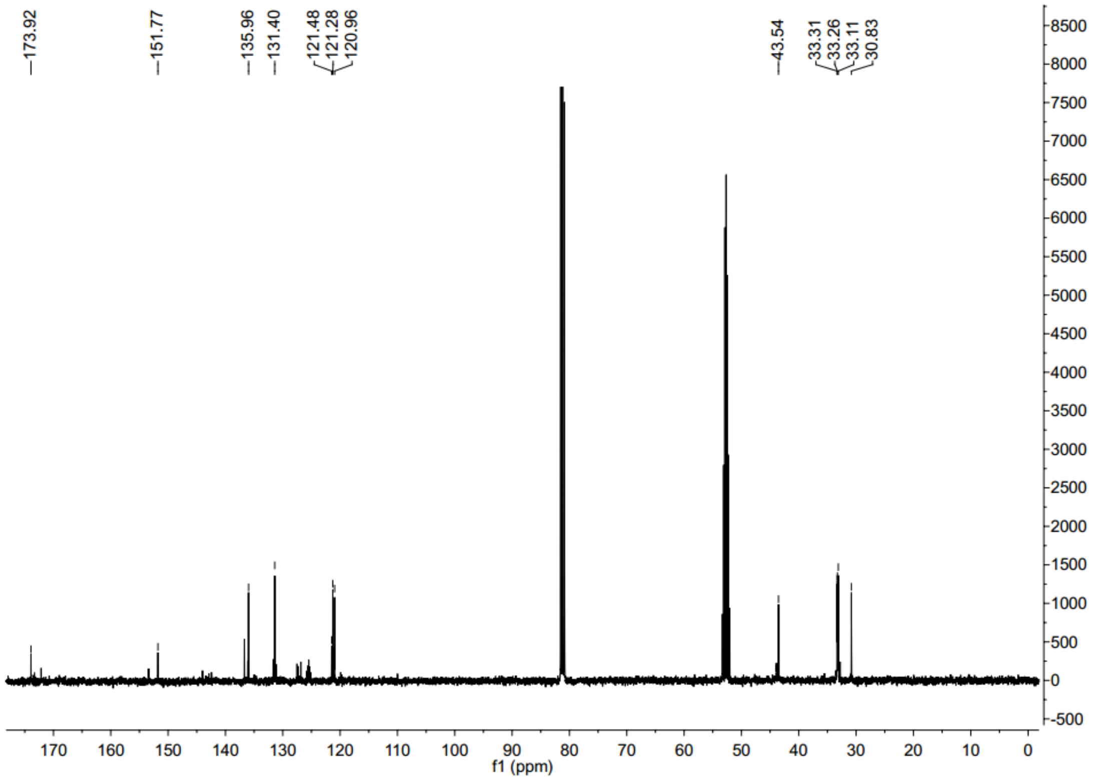

Figure S139 ^{13}C NMR spectrum of compound **30e.**

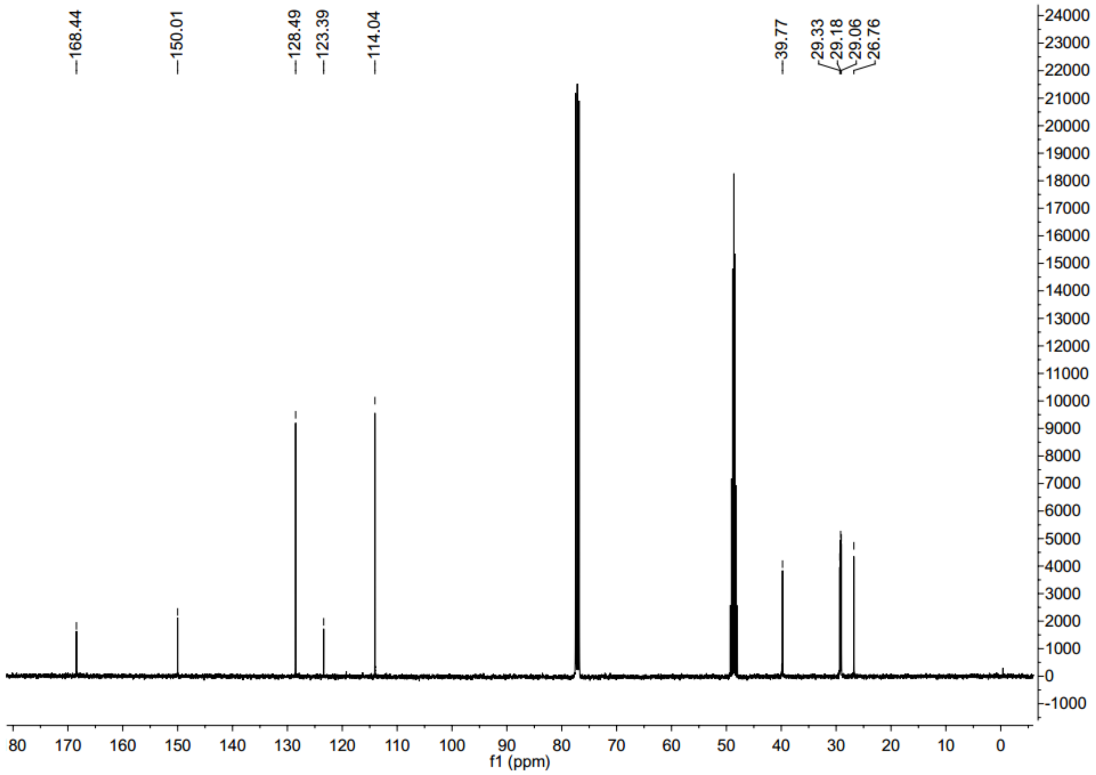

Figure S140 ^{13}C NMR spectrum of compound **31e.**

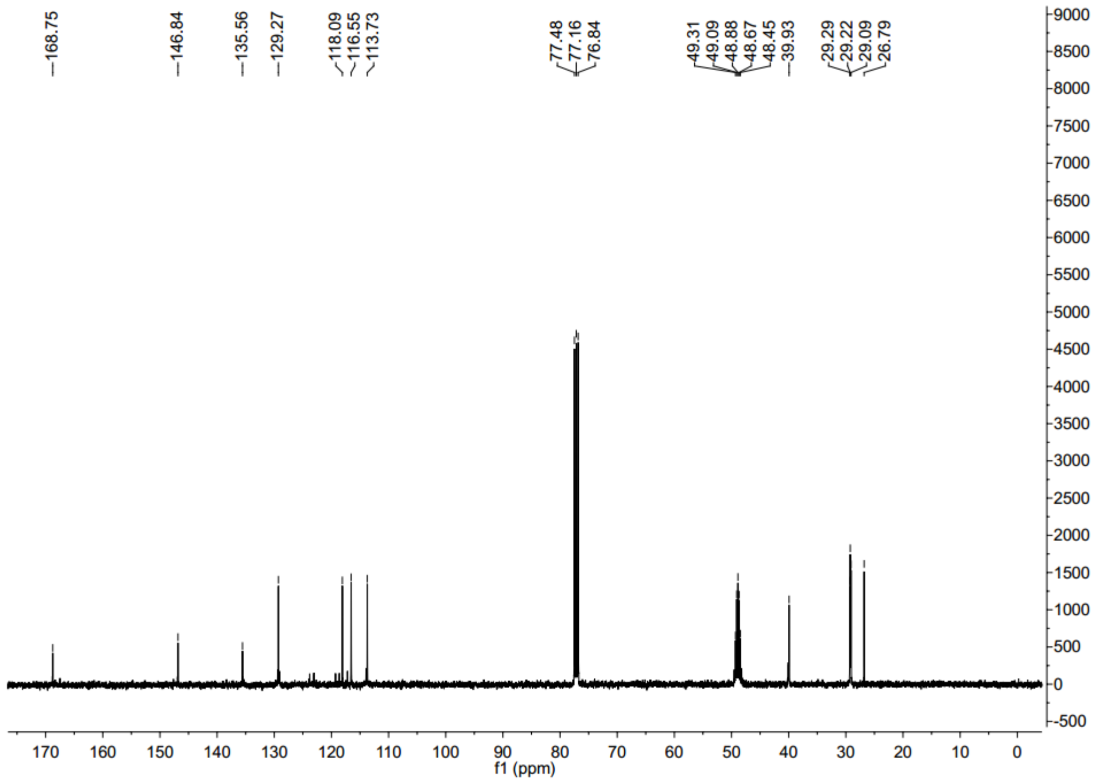

Figure S141 ^{13}C NMR spectrum of compound **32e.**

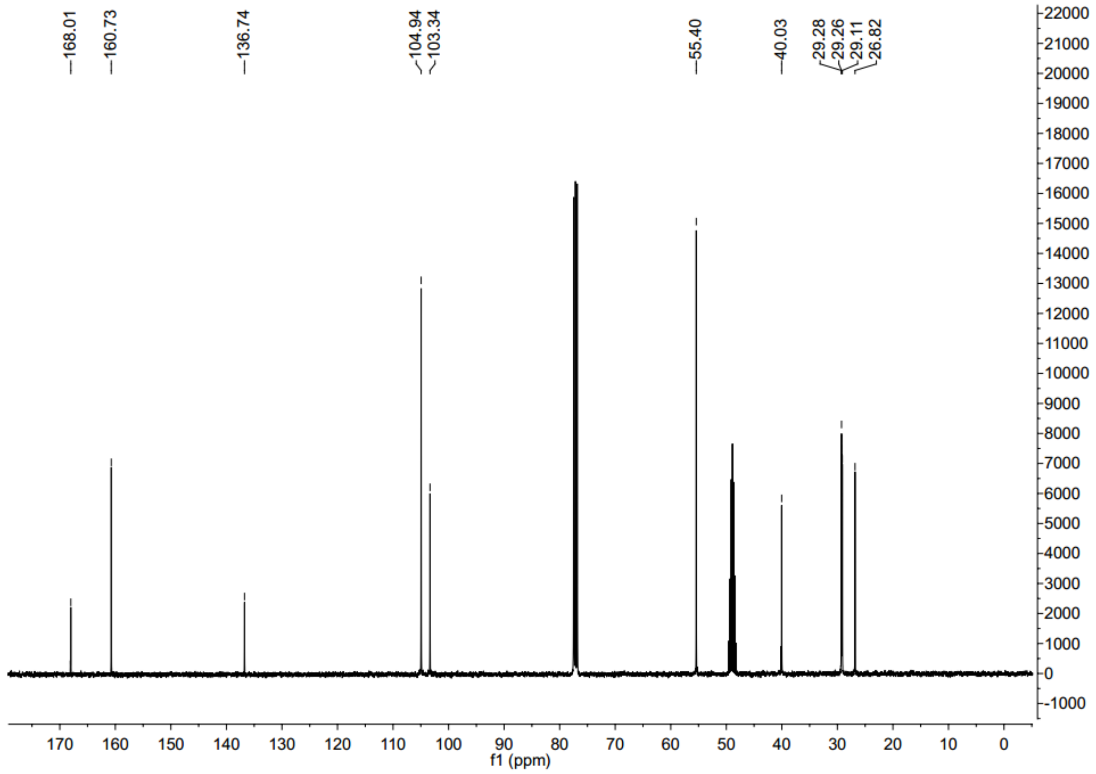

Figure S142 ^{13}C NMR spectrum of compound **33e.**

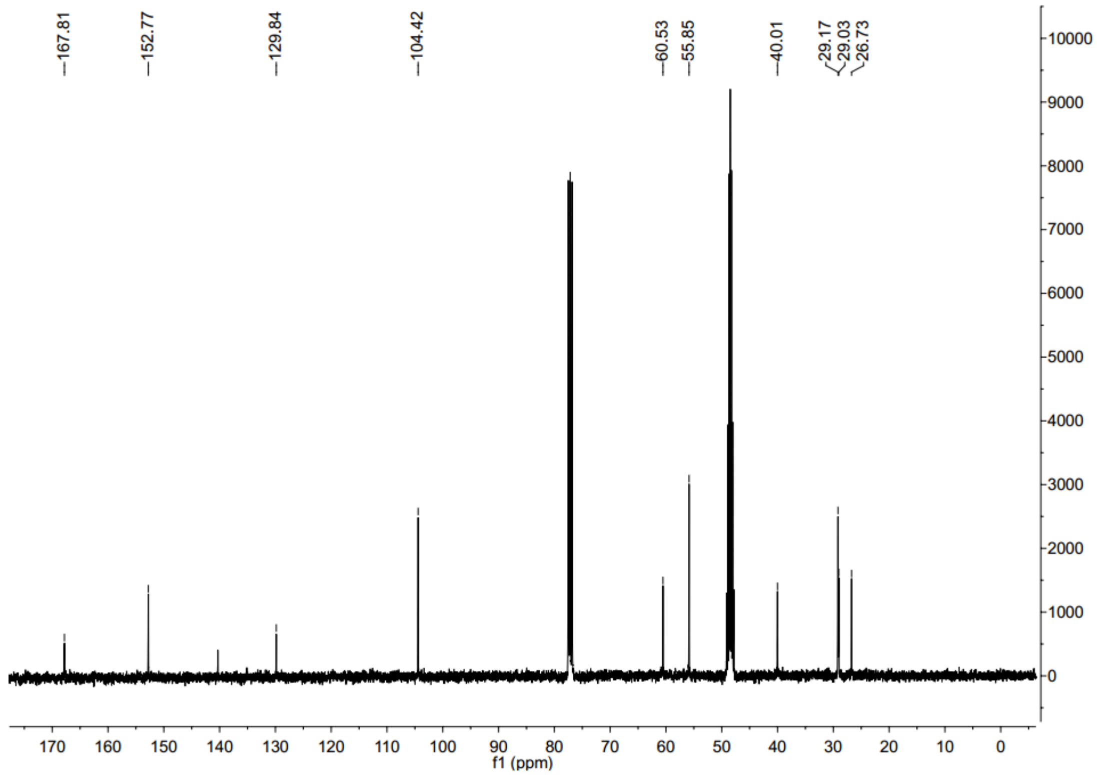

Figure S143 ^{13}C NMR spectrum of compound **34e.**

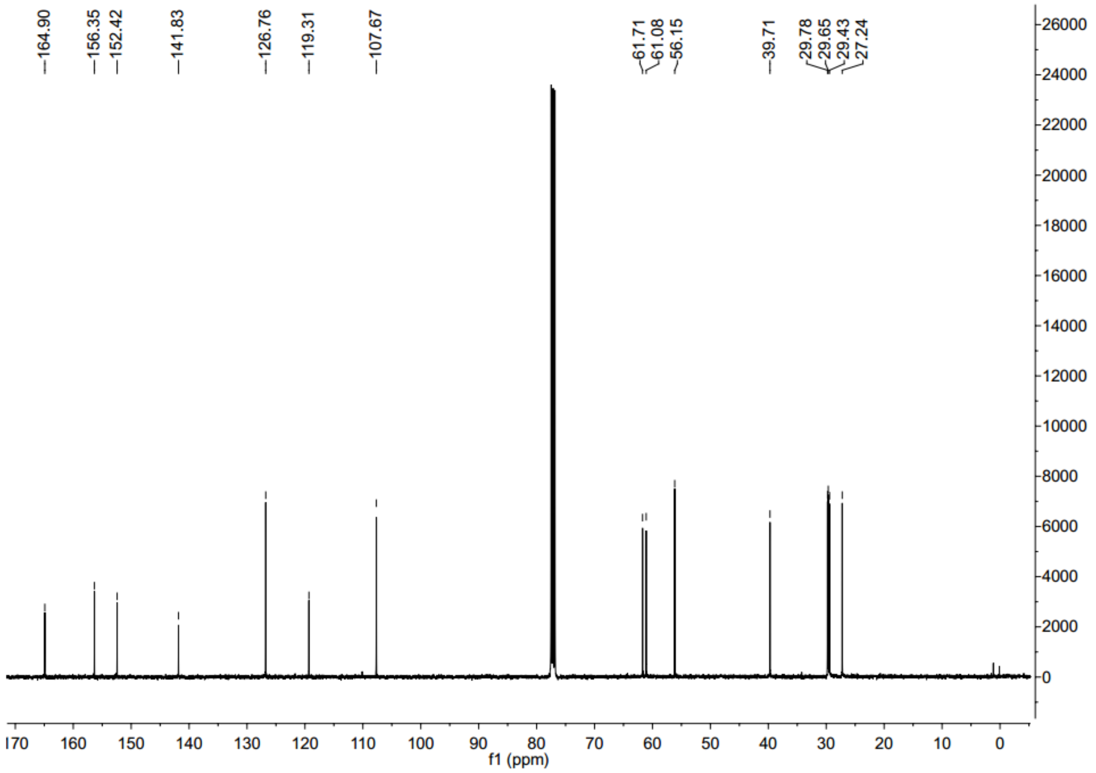

Figure S144 ^{13}C NMR spectrum of compound **35e.**

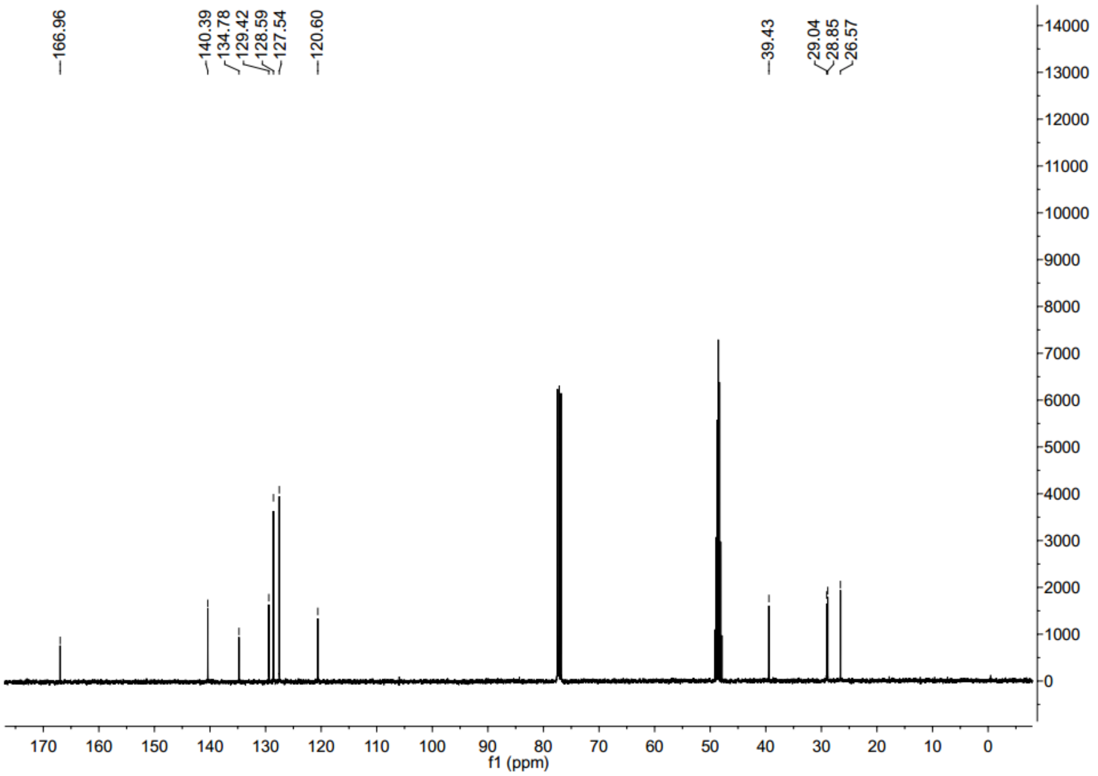

Figure S145 ^{13}C NMR spectrum of compound **9d.**

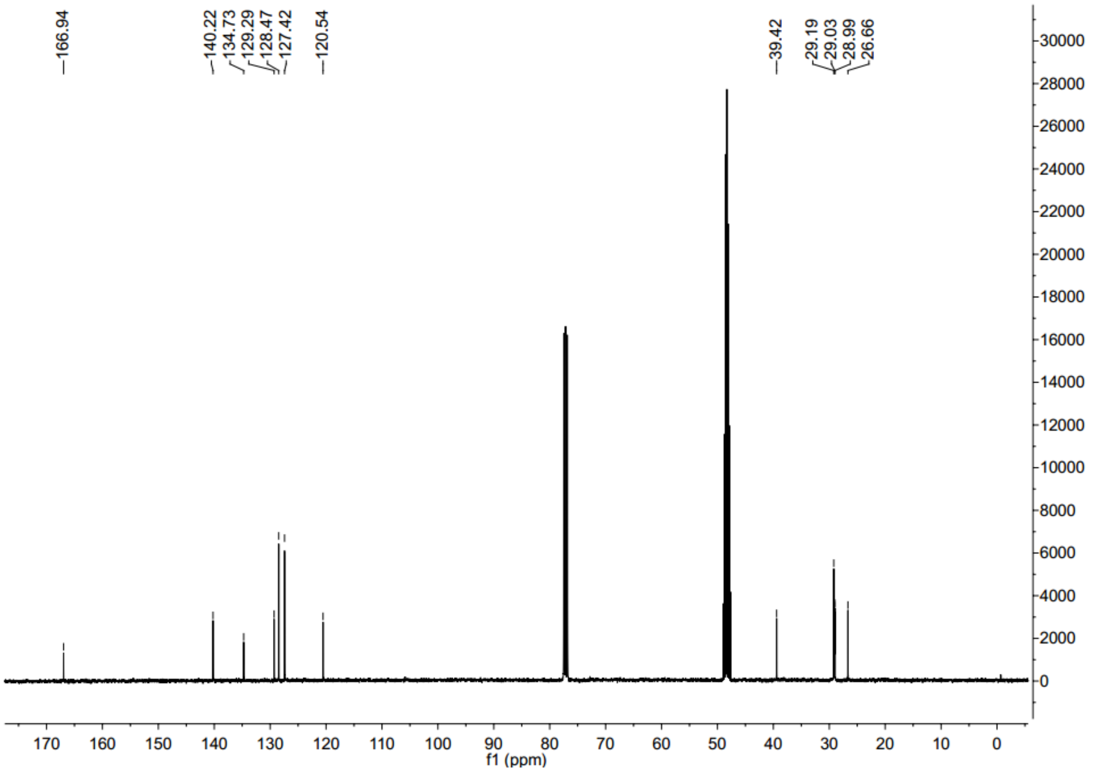

Figure S146 ^{13}C NMR spectrum of compound **9f.**

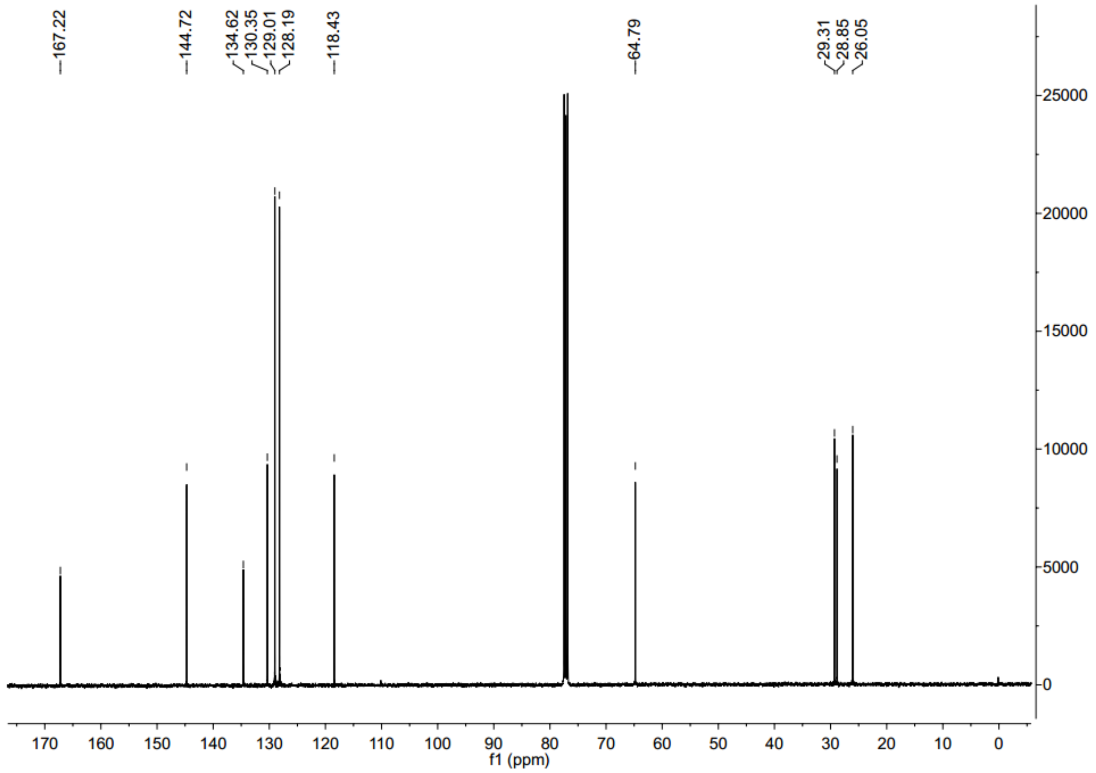

Figure S147 ^{13}C NMR spectrum of compound **9g.**

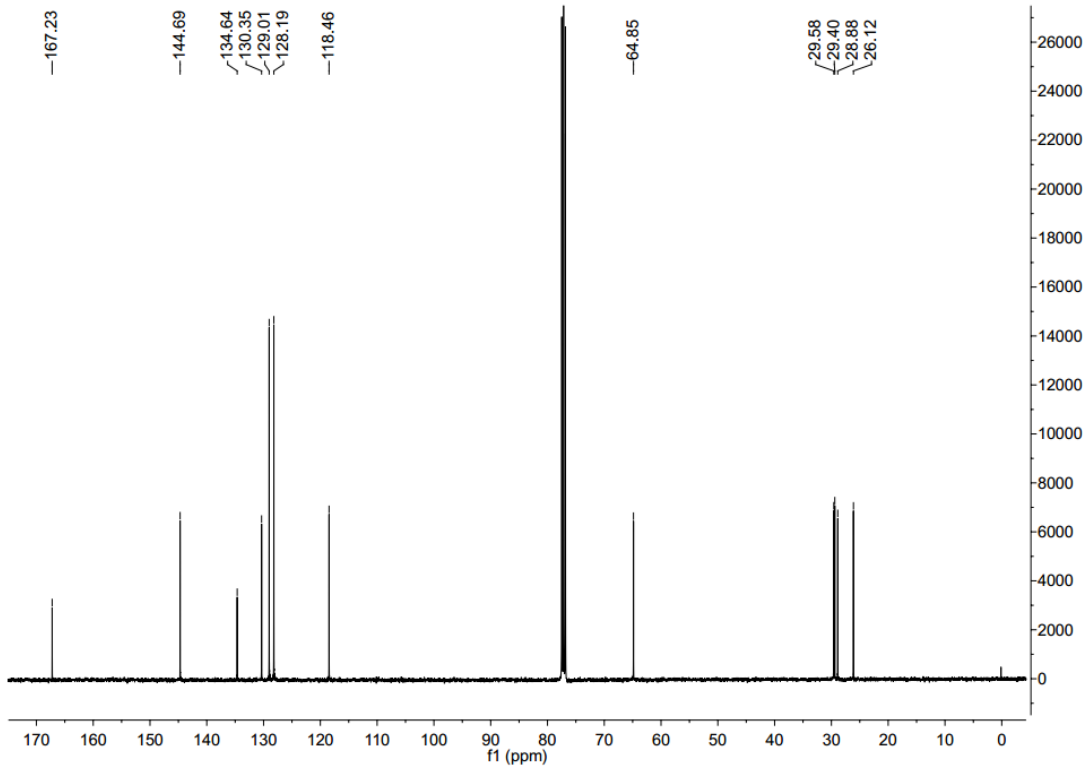

Figure S148 ^{13}C NMR spectrum of compound **9h.**

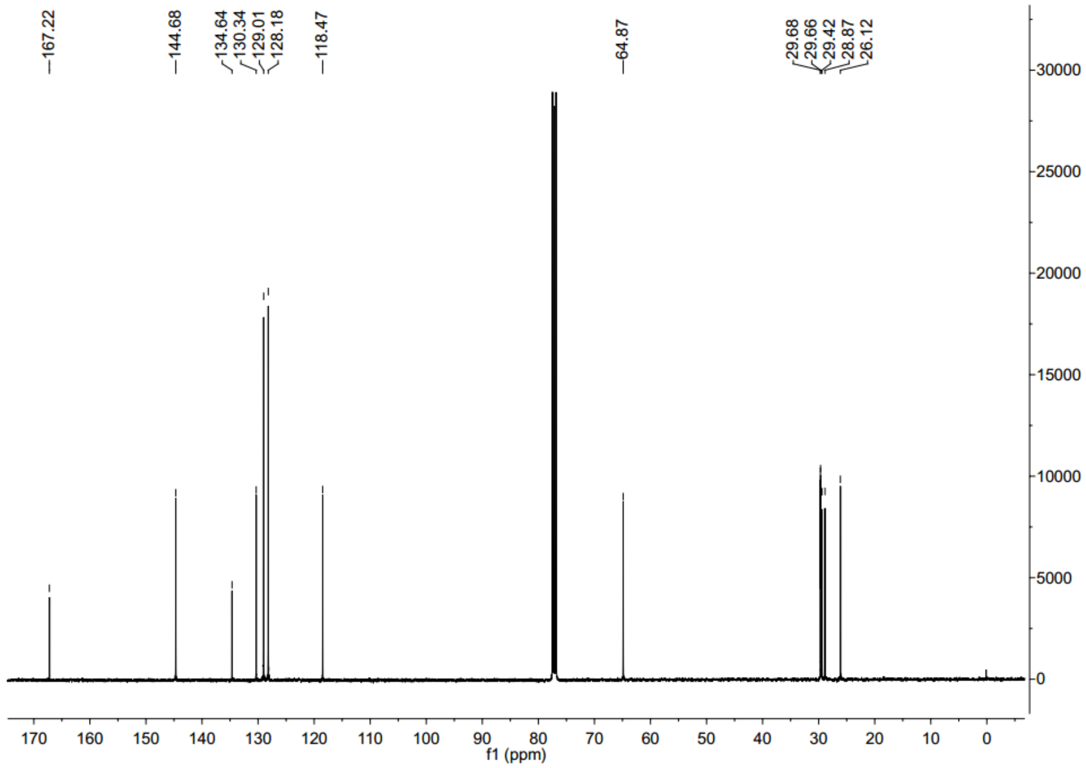

Figure S149 ^{13}C NMR spectrum of compound **9i.**

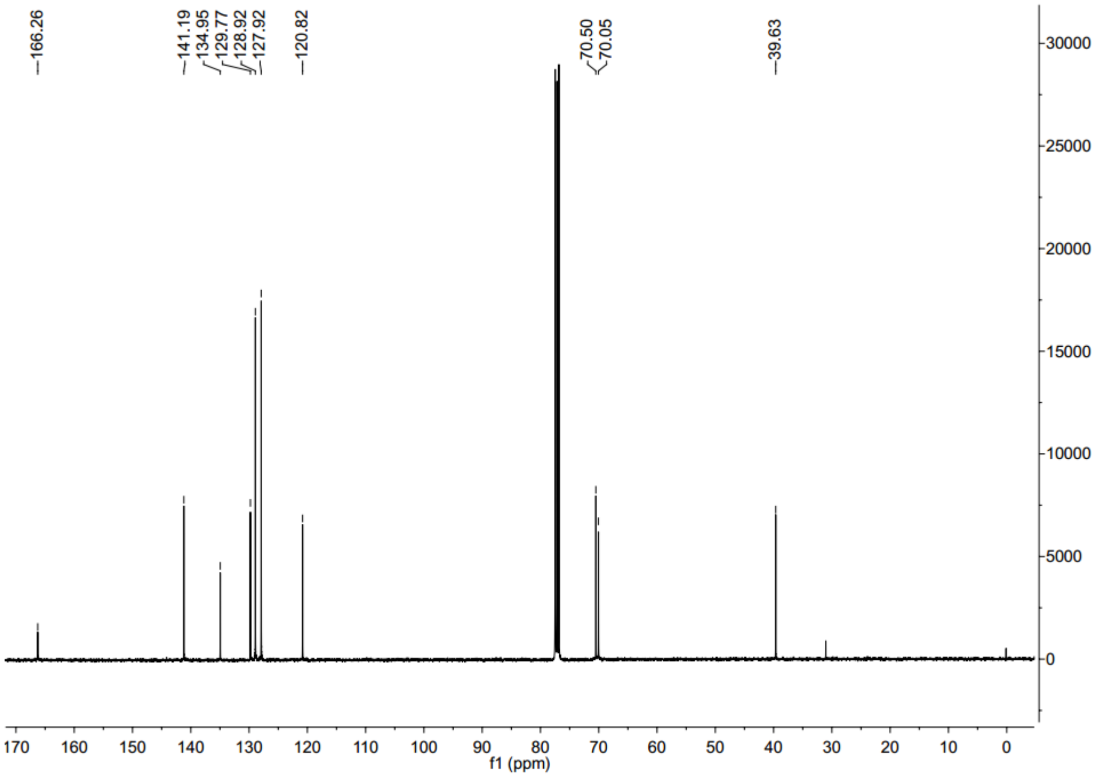

Figure S150 ^{13}C NMR spectrum of compound **9j.**

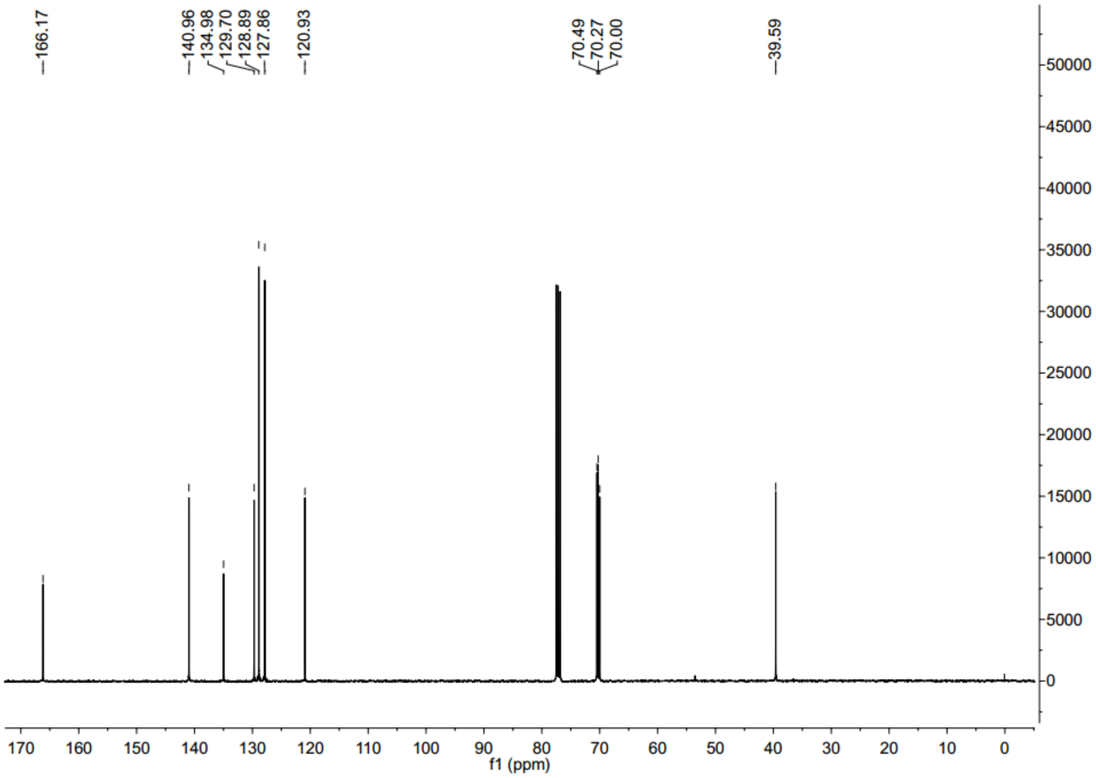

Figure S151 ^{13}C NMR spectrum of compound **9k.**

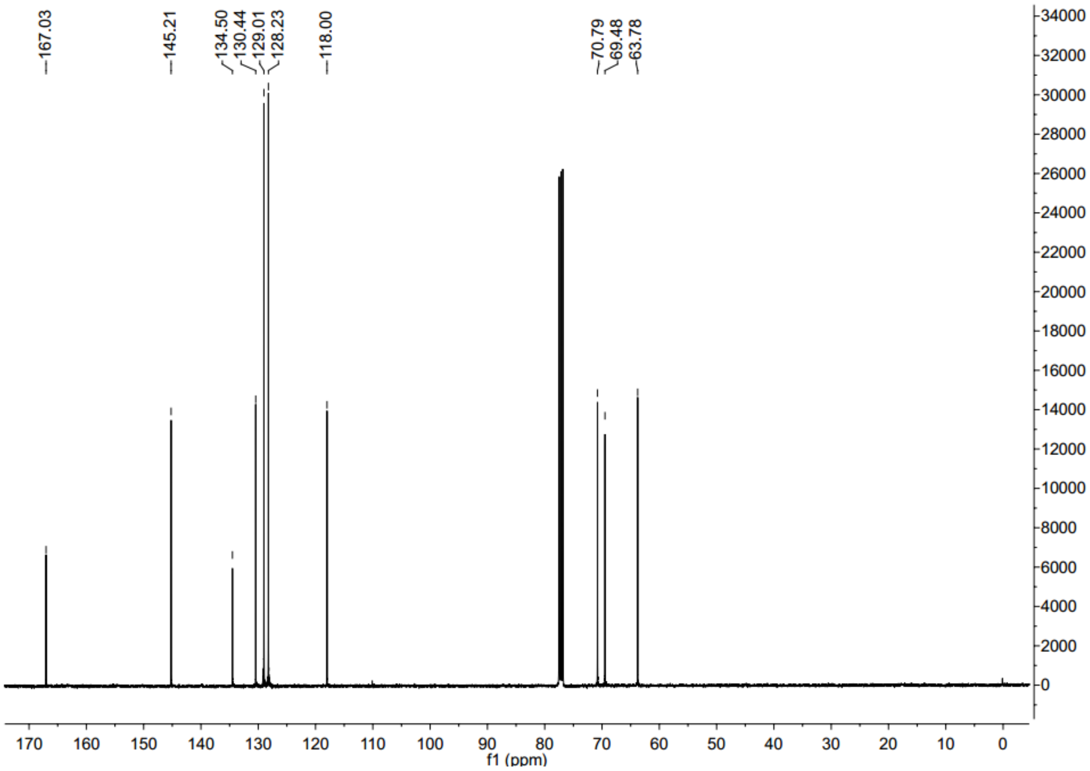

Figure S152 ^{13}C NMR spectrum of compound **9l.**

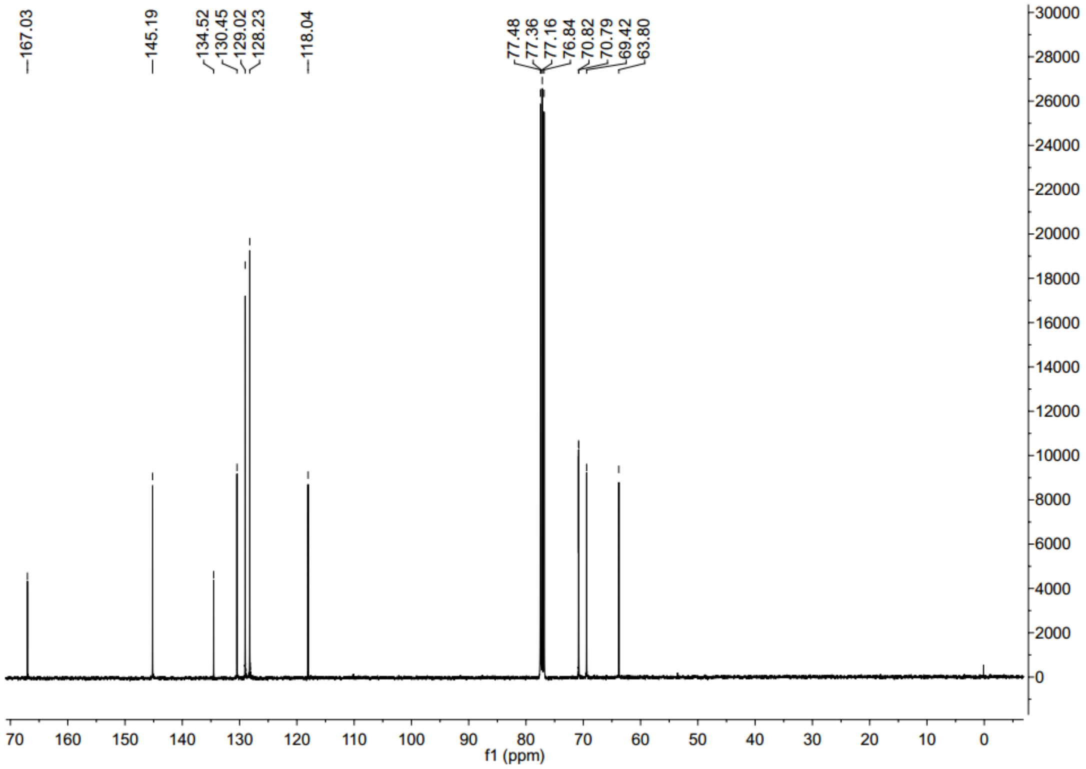

Figure S153 ^{13}C NMR spectrum of compound **9m.**
